# Pharmacy students’ perspective on remote flipped classrooms in Malaysia: a qualitative study

**DOI:** 10.3352/jeehp.2025.22.2

**Published:** 2025-01-14

**Authors:** Wei Jin Wong, Shaun Wen Huey Lee, Ronald Fook Seng Lee

**Affiliations:** School of Pharmacy, Monash University Malaysia, Bandar Sunway, Malaysia; Hallym University, Korea

**Keywords:** COVID-19, Communication, Curriculum, Focus groups, Pharmacy students, Malaysia

## Abstract

**Purpose:**

This study aimed to explore pharmacy students’ perceptions of remote flipped classrooms in Malaysia, focusing on their learning experiences and identifying areas for potential improvement to inform future educational strategies.

**Methods:**

A qualitative approach was employed, utilizing inductive thematic analysis. Twenty Bachelor of Pharmacy students (18 women, 2 men; age range, 19–24 years) from Monash University participated in 8 focus group discussions over 2 rounds during the coronavirus disease 2019 pandemic. Participants were recruited via convenience sampling. The focus group discussions, led by experienced academics, were conducted in English via Zoom, recorded, and transcribed for analysis using NVivo. Themes were identified through emergent coding and iterative discussions to ensure thematic saturation.

**Results:**

Five major themes emerged: flexibility, communication, technological challenges, skill-based learning challenges, and time-based effects. Students appreciated the flexibility of accessing and reviewing pre-class materials at their convenience. Increased engagement through anonymous question submission was noted, yet communication difficulties and lack of non-verbal cues in remote workshops were significant drawbacks. Technological issues, such as internet connectivity problems, hindered learning, especially during assessments. Skill-based learning faced challenges in remote settings, including lab activities and clinical examinations. Additionally, prolonged remote learning led to feelings of isolation, fatigue, and a desire to return to in-person interactions.

**Conclusion:**

Remote flipped classrooms offer flexibility and engagement benefits but present notable challenges related to communication, technology, and skill-based learning. To improve remote education, institutions should integrate robust technological support, enhance communication strategies, and incorporate virtual simulations for practical skills. Balancing asynchronous and synchronous methods while addressing academic success and socioemotional wellness is essential for effective remote learning environments.

## Graphical abstract

[Fig f2-jeehp-22-02]

## Introduction

### Background

The flipped classroom has been reported to be a useful and innovative teaching style for student development. In recent years, there has been a growing adoption of the flipped classroom model, particularly in higher education. In a flipped classroom model, traditional teaching methods are reversed. Students learn new content outside of a classroom setting instead of the traditional in-class setting. Some of the reported benefits of flipped classrooms include improved learning and engagement across different settings, such as in health professions programs [1] and higher education [2]. Other reported benefits include the development of useful life skills, such as communication and time management [3]. Considering the importance of life skills development in helping healthcare graduates become work-ready [4], opportunities for enhancement or improvement of the flipped classroom are worth exploring.

In the flipped classroom, students go through pre-class learning materials, independently learning new content. This could be in the form of pre-recorded short lectures with formative assessments and relevant reading materials. Students then come together for in-class sessions where there are opportunities to solidify and build on knowledge. These in-class sessions usually occur in person and at a suitable venue. Activities can include group work and hence require appropriate classroom setups. In-person interaction can help support the development of soft skills like communication and teamwork, which has been said to be an additional benefit of the pedagogical design of the flipped classroom [3]. The required physical space and classroom setup for in-person sessions can sometimes be a challenge, as not all teaching institutions may be able to support this environment.

In an evaluation study, investigators examined students’ experiences of remote learning, and students reported a preference for hybrid learning [5]. However, as the study was limited to pharmacy schools in the United States, the authors identified the need for further studies in different regions of the globe considering the influence of unique cultural learning environments to further explore this relationship.

### Objectives

We sought responses from university students who went through an undergraduate program with a predominantly flipped classroom that was delivered purely remotely in Malaysia, where such data are lacking. This study aimed to gain insights into how students perceive their learning experiences of a remote flipped classroom, which can be useful to future educators to identify areas for potential improvement.

## Methods

### Ethics statement

The study was approved by Monash University Human Research Ethics Committee (project #21922). Informed consent was obtained from all participants.

### Personal characteristics of research team

WJW, BPharm, MIPH/MHM and RFSL, BPharm, PhD, and SWHL MPharm, PhD are male academics at the School of Pharmacy, Monash University. All 3 have experience in educational research.

### Relationship with participants

Research team members had previously taught the interviewed students, which may have introduced potential bias. Students were informed about the objective of the research, the benefits to them, and response anonymity, clarifying that their opinions solely served research purposes and would not impact their academic standing.

### Context

The Bachelor of Pharmacy at Monash University Malaysia is a 4-year program and adopts a primarily flipped classroom model. Teaching in the program was delivered remotely through a mix of pre-class materials and in-class learning activities, which included formative self-assessment quizzes and interactions with pre-recorded clips. This was followed by face-to-face on-campus in-class learning activities. Details about the program have been described elsewhere [6]. In 2020, this model was adapted to be delivered remotely in response to the coronavirus disease 2019 (COVID-19) pandemic (Table 1). Examinations were conducted remotely and were evaluated accordingly [7]. Specialized teaching activities such as extemporaneous compounding labs were also conducted remotely and feedback was sought [8].

### Study design

#### Theoretical framework

We utilized a qualitative methodology underpinned by inductive thematic analysis for this study, and the approach is presented in accordance with the Consolidated Criteria for Reporting Qualitative Research available at https://www.equator-network.org/reporting-guidelines/coreq/.

#### Study participants

Students enrolled in the Bachelor of Pharmacy program were invited to participate in the focus group discussion. A total of 8 focus groups were conducted, each comprising 2 to 4 students. In total, 20 students, comprising 2 men and 18 women were recruited via convenience sampling via emails and face-to-face announcements. Each participant received a token of 50 Malaysian ringgit (approximately US$12). The age range of the participants ranged from 19 to 24 years. There were 2 students from year 1, 9 students from year 2, 4 students from year 3, and 5 students from year 4.

#### Data collection

Students from different year levels were grouped into cohorts and participated in 2 focus group sessions throughout the academic year, as illustrated in Table 2. This approach allowed us to understand students’ attitudes and behaviors over time, providing a deeper understanding of the impact of remote learning styles.

We developed an open-ended and flexible question guide to assess students’ perspectives, understanding, experiences, and improvement, which we sought to explore via qualitative methods. The focus groups were led by WJW and RFSL with video and audio recorded via Zoom (Zoom Video Communications Inc.) (Dataset 1). The focus group sessions were held in English, with each session lasting up to 120 minutes. Field notes were also taken during the interview process by one of the authors to aid subsequent data analysis (Dataset 2). Audio recordings were transcribed using the REV speech-to-text service (REV; https://www.rev.com/). Participants were not given access to the transcripts or subsequent codes and results.

#### Data analysis

Inductive thematic analysis was applied to the interview text using emergent coding. Initially, the 2 authors established categories following a preliminary examination of the data. A comparison of these category lists led to discussions on discrepancies, resulting in the adjustment and consolidation of categories. Subsequently, a consolidated list (codebook) was independently applied to the data corpus by both study authors (Dataset 3). To increase the trustworthiness of interpretations, multiple discussions were conducted between the 2 authors, fostering reflective refinement in the process until the team agreed that thematic saturation was achieved. Analyses were carried out using NVivo (Release 1.5.2; QSR International).

## Results

**Fig. 1.**
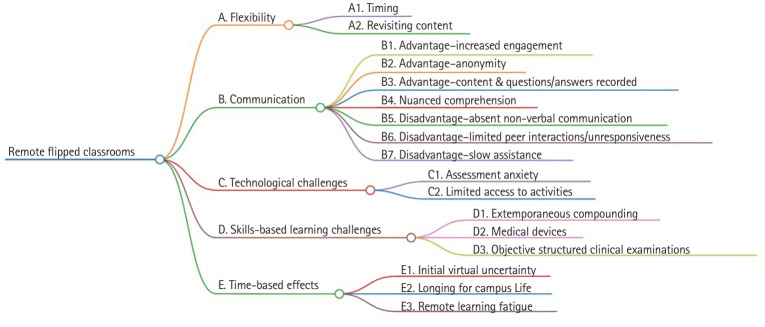
Summary of themes found in this study.

We identified 5 major themes in this study: flexibility, communication, technological challenges, skill-based learning challenges, and time-based effects. The major themes and subthemes are summarized in Fig. 1 and a list of themes and subthemes are available in Supplement 1.

### Theme A. Flexibility

Two aspects of flexibility were reported by students in the context of interactive lectures. First, there was a time flexibility to engage with lectures at their own convenience since the lectures were recorded (Supplement 1, A1). Another aspect of flexibility was revisiting content, where students appreciated the convenience of reviewing material through playback, enabling a thorough grasp of concepts (Supplement 1, A2)

### Theme B. Communication

Communication-wise, there was a mix of advantages and disadvantages from remote flipped classrooms. First, in remote lectures, there was an increase in engagement in terms of the volume of questions asked due to increased confidence in asking questions and the ease of formulating questions in written form (Supplement 1, B1). This increase in confidence could be related to being able to post questions anonymously while remote (Supplement 1, B2). In addition, remote sessions eased the documentation of content, as questions and answers, lectures and even slides could be recorded in high resolution (Supplement 1, B3). Nevertheless, certain nuances of content comprehension were acknowledged to be better suited for in-person interactions, particularly for clarifying complex concepts or intricate diagrams (Supplement 1, B4).

For remote workshops, disadvantages include the absence of non-verbal cues from peers complicating communication (Supplement 1, B5). In addition, remote workshops had reduced interactivity due to a lack of physical presence, inability to see peers due to the absence of video or being away from the keyboard and being limited to interacting with specific group members in breakout rooms. (Supplement 1, B6). Remote classes were also not conducive to organic post-class discussions (Supplement 1, B6). Finally, for workshops, the breakout room systems slowed down facilitation by academics as they needed to transition to different breakout groups and could only speak to one group at a time. (Supplement 1, B7).

### Theme C. Technological challenges

Technological challenges were very much related to internet connectivity, which is imperative for remote learning. Students expressed concerns about missing important information during key learning activities due to connection drops, or outright disconnections from sessions (Supplement 1, C1). If these happened in the context of high-stakes activities like assessments, these anxieties were compounded (Supplement 1, C2).

### Theme D. Skill-based learning challenges

Students cited challenges in skill-based learning domains such as objective structured clinical examinations (OSCEs), extemporaneous compounding, and medical device counselling. For lab-based activities such as extemporaneous compounding, Monash Malaysia implemented a home-based compounding lab activity via video conferencing which had challenges in terms of validation of quality of compounded products (Supplement 1, D1). Similarly, medical device counselling faced limitations as students lacked access to necessary devices, thereby hindering hands-on familiarity and comprehensive learning (Supplement 1, D2).

For OSCEs, students cited increased confidence doing these remotely due to the modality of delivery. However, they recognized the disparity between remote and in-person assessment, revealing that while remote assessments elicited reduced nervousness, they also underscored a dependence on references; thus, some felt they would be less equipped for real-life counselling situations (Supplement 1, D3).

### Theme E. Time based effects

Students who began university education using remote flipped classrooms expressed a sense of helplessness and disorientation due to unfamiliarity and inability to find peers to seek counsel from due to the remote environment (Supplement 1, E1). Once they adapted, many students found value in the flexibility of remote learning. However, upon long exposure students cited a longing to return to campus due to the importance of human interactions and inconducive study environments out of campus (Supplement 1, E2). In addition, students cited fatigue of remote learning, which caused loss of focus and a feeling of tiredness and boredom due to the remote modality (Supplement 1, E3).

## Discussion

### Key results

This study aimed to gain insights into how students perceive their learning experiences of a remote flipped classroom during the COVID-19 pandemic. Our study reveals key advantages in flexibility and engagement from remote lectures, which could be implemented for in-person flipped classrooms, but also challenges in communication, technological barriers, skill-based learning, and time-based effects. These issues need to be addressed if remote approaches are to be adopted moving forward.

### Interpretation/comparison with previous studies

In remote classrooms, the ability to interact with content at their own pace and review materials is key for reinforcing understanding [9]. We observed similar benefits of remote flexibility in our study, though our practice of combining asynchronous and synchronous lectures afforded benefits of facilitating understanding whilst allowing students to ask questions.

In remote learning settings, there is a preference for anonymity, which boosts participation due to reduced fear of negative evaluation by peers [10]. In addition, when comparing modes of engagement online using video conferencing, chat is preferred to using mics, screen sharing, and video sharing [11]. We observed similar preferences for anonymity and chat, which boosted engagement in remote lectures, although students noted nuances regarding types of content that are easier explained in person versus online. For remote workshops, communication challenges and technical hurdles were significant. Participants noted the lack of visual cues and limited peer responsiveness, echoing another study by Tiene [12]. These issues often led to miscommunication and negatively impacted group dynamics. Hence, workshops conducted remotely may not be ideal and additional scaffolding would be needed for effective implementation when remote modalities are inevitable.

Technical problems such as internet connectivity causing issues with learning equity and assessment anxiety have been echoed by other studies [13], and environmental factors such as distractions and power cuts can also compound these problems. Addressing these multifactorial issues can be challenging, but educational institutions can address these by providing robust tech support and asynchronous assessments or flexible scheduling of assessments.

Skills-based teaching in pharmacy such as OSCEs, medical devices, and compounding are challenging to deliver and assess remotely. For remote OSCEs though comfortable and convenient, limitations related to development of non-verbal skills have been found [7]. In this study, students additionally highlighted fears of the transferability of OSCE skills learned virtually to real-life practice. For equipment-dependent skills, such as compounding and the use of medical devices, logistical and skill validation issues need addressing [8]. Finally, assessments for skills-based learning have integrity-related concerns, as is the case for all remote assessments [14].

We found that remote flipped classrooms presented initial adaptation difficulties and challenges upon longer-term exposure. Similar challenges have been observed during the initiation of flipped classrooms [2], and it has been noted that long exposure to remote classrooms is unsustainable due to lack of self-regulation [15]. Overall, educational institutions need to refine remote education strategies to bridge the initial adaptation and address the negative longer-term consequences of prolonged remote teaching. Students’ desire to return to campus highlights the potential of remote learning to contribute to feelings of isolation and loneliness. This may have negative effects on overall mental and emotional well-being, thereby exacerbating disengagement and education.

### Limitations

Our study was designed to track time-based effects but had limitations involving sample size and diversity, response bias, and researcher bias. The study was conducted at a university in Asia, which may be influenced by certain regional educational practices and perspectives. However, its findings align with global trends during the COVID-19 pandemic, indicating that these challenges are representative rather than unique. Response bias could have occurred since the academics conducting focus groups also taught the pharmacy course. Steps were taken to minimize these issues by conducting briefings before the focus groups about response anonymity and informing students that their responses would not affect their academic standing.

### Generalizability

For remote lectures, a mix of asynchronous and synchronous sessions is ideal to balance flexibility with crucial real-time interactions. In remote workshops, faculty should ensure clear instructions, encourage switching on of webcams, provide ample resources, and facilitate technical support to ease implementation. To deliver practical skill training remotely for compounding, OSCEs, and medical devices, the use of video demonstrations, virtual simulations, remote practicums and if possible, exposure to real-life scenarios remotely or in person (e.g., compounding labs or community pharmacies) is important. For lectures, the blended approach suggested above can be considered for institutions that do not usually deliver flipped classrooms remotely. The findings of our study though done in a flipped classroom setting are broadly applicable to remote higher education in general, since traditional classrooms when implemented remotely have many similar elements.

### Conclusion

The study highlights the benefits and challenges of remote flipped classrooms. For remote lectures, the benefits include flexibility and increased engagement due to anonymity. For remote workshops, attention must be given to address inherent communication challenges within this setting. The broader remote learning experience unveils skill-based learning barriers and specific time-based effects from prolonged exposure. As education evolves, these findings emphasize the necessity of a balanced approach that melds technological enhancements with proactive support, fostering both academic success and socioemotional wellness. This study stands as a roadmap for educators, administrators, and policymakers, guiding them to navigate the complexities of modern education holistically.

## Figures and Tables

**Figure f2-jeehp-22-02:**
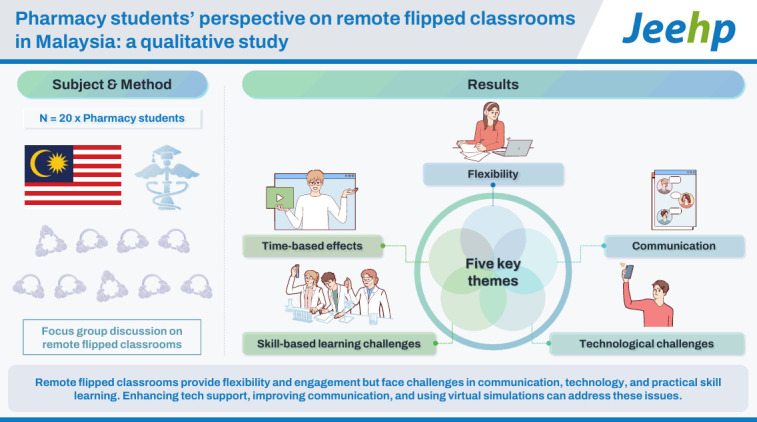


**Table 1.** Differences between in-person and remote flipped classrooms

In-person flipped classrooms	Settings	Remote flipped classrooms
Reading material included text, videos, links to external sites, activities, tasks and multiple-choice questions to gauge understanding	Pre-class materials	No change
In-person	In-class lectures	Mixture of asynchronous and synchronous depending on respective units
On campus in active learning classrooms	In-class workshops	Using Zoom breakout rooms
On campus in specially designed rooms	Skills-based learning such as objective structured clinical examinations	Using Zoom breakout rooms with an examiner
On campus in laboratory	Extemporaneous compounding	Using Zoom breakout rooms with a facilitator
On campus in simulated rooms with the use of demo kits	Medical device teaching	Using Zoom breakout rooms with a demonstrator going through device and students watching demonstration online

**Table 2.** Focus group participant composition

	FG participant composition
Round 1–2020	
FG1	2×Y1 (2020)
FG2	4×Y2 (2020)
FG3	4×Y2 (2020)
FG4	4×Y3 (2020)
FG5	4×Y4 (2020)
Round 2–2021	
FG6	3×Y2 (2021)–2 from FG1 and 1 new participant
FG7	3×Y3 (2021)–all previously part of FG2 and FG3
FG8	3×Y4 (2021)–2 were from FG4 (1 of whom was repeating the third year) and 1 new student.

FG, focus group; Y, year.

## Footnotes

**Data availability**
Data files are available from Harvard Dataverse: https://doi.org/10.7910/DVN/UZVAZR Dataset 1. All video and audio recording scripts.

Dataset 2. Field note from the focus group.

Dataset 3. Subsequent codes, emergent themes in the study.

## Supplementary materials

Supplementary files are available from Harvard Dataverse: https://doi.org/10.7910/DVN/UZVAZR

Supplement 1. Themes, sub-themes, and example quotes from focus groups with pharmacy students.

Supplement 2. Audio recording of the abstract.
