# Supplementary material for: Pharmacy students’ perspective on remote flipped classrooms in Malaysia: a qualitative study
Source: J Educ Eval Health Prof. 2025 Jan 14;22:2. doi: 10.3352/jeehp.2025.22.2 (PMC12055608; doi:10.3352/jeehp.2025.22.2)
Supplement: Supplementary file 1 [file jeehp-22-2-dataset1.pdf]

Dataset 1

Deidentified FG1Y2 - Sept 2020

Lecturer A:

Yeah, so I've just started the recording. So, we won't go longer than two hours, but we want this to be a very honest and frank discussion with the four of you. And again, feel free to share your thoughts and just chip in, and just imagine that you're having a conversation, ideally, we would like to have done this face-to-face in a room here on campus or somewhere, but obviously, because of what we are going through right now, this will be the next best thing. So, the first part of our questions, so we would like to ask you really is just targeted on the online learning experiences that you guys have gone through so far. So, because of COVID and all that has been happening in the world from the first quarter of this year, we got you all to just flip suddenly to an online learning environment. So, the first question there really, that I just have to ask you, how has the online learning experience been so far? Anybody.

Student B:

I think it has been okay so far because it has the element of flexibility. So we are allowed to complete our interactive lecture, anytime, anywhere, according to our own pace. And we can review back the video. That's a good aspect of it. Flexibility.

Lecturer A:

Okay. Anybody else from the three of you?

Student A:

Okay. So, in my point of view, yes, it has been accessible for us. We can reach different areas easily. Like for example, we can find recordings easily. We can record the lectures by ourselves or whether they are recorded by the lecturers themselves. The discovery materials are constantly updated. However, sometimes it's not that easy to reach lecturers themselves. So like, there is a problem. I emailed the lecturer about it and that was last week, until now I haven't received any reply. So maybe they're still accustomed to the new online system, but at the same time, we're not getting immediate responses. In addition to that, we're also not getting access to things like, for example, the exams. So you get the exam, whether your answer is right. Okay. It's right. If it's wrong, it's wrong. You don't even get to know which answer is wrong and why is it wrong? And you just find out that you got this mark. Previously, we were able to talk to lecturers but now, because of online thing, we can't talk to anyone.

Lecturer A:

Okay. Thanks so much. So you brought up two interesting points, which I hope to do talk about further after this, but really just centering on the overall online learning experiences so far. I just curious as well for [Student B 00:03:27] and for Student C, do you all have a similar thoughts as to what has been shared so far by two of your other friends? Or do you have anything different?

Student C:

For me I feel like learning through online saves more time for me. I have class at 09:00 and I can just wake up at 08:55 and go to the school, like for public transport, for like one hours and [inaudible 00:03:55]. I think it is very good for me. Like what all you say flexible. And even through online learning, because previously we learning at the lecture hall and then the slides at the projector. And then if we want, we need to get picture and it kinds of a bit blur and something and [inaudible

00:04:16]. The lectures skipped doing next flight. But if it's online, I can just keep on screenshot and my note is what I need from there.

Lecturer A:

Thank you. And Student C, anything else similar or anything different?

Student A:

Yeah, I'm not sure whether my opinion is a bit different from them or not. But for me, I think that if we are to go back to a physical class next year, I think that lectures conducted online is quite a cool thing. But workshop help online is not at all. Because workshop, most of them are live workshops. And for example if my discussion is a bit difficult to, I presented thing it's quite difficult virtually to discuss a topic from workshop like that. And also asking questions is a bit troublesome because you have to invite the lecturer in and out from the Zoom Room. And also sometimes in my line drop [inaudible 00:05:30] if a session is recorded never mind, but not most of the workshop sessions are recorded. So if maybe for example the lecturers are seeing something very important and my line just happens to not be working that time.

Student A:

I will miss that information. And it's quite hard to ask that actually, to repeat that point again, because you are not certain which part she's describing and she doesn't know which part you missed out. Yeah. So for my opinion lectures conducting online is fine and quite cool. We can capture every notes without using our phone to take a blurry vision like what Student B had mentioned just now, but the workshop part is really a bit hard for us to do it online.

Student B:

I would like to add onto what Student C had mentioned. So workshop could be a bit awkward when it is conducted online because some members might be unresponsive. They might be like completely MIA. They can just leave their computers on. So it has happened before and it's a bit troublesome.

Student A:

Even when actually talking like right now, I'm just talking as if I'm talking to myself. I'm not actually experiencing the actual teammates here, like physical communication instead of just saying words. For example, just point out something like use verbal communication, and using hands and things like that. No, we can't do this here.

Student B:

Connection problem is another thing as well like Student C mentioned.

Lecturer A:

Okay. Thank you for that. The four of you have given us quite a good start to the discussion and you have mentioned some points which we actually were planning to also to ask you later on as part of the questions that we have, but you've really mentioned it. So I thought that's a good start. So I would just try to take the time to filter them and to explore them. Okay. The reality is that what you've had gone through so far this year is slightly different than last year because of the nature of being in a physical class and this year being in an online environment. Again, maybe in a few words or very simply, can you just illustrate or share personally how you've felt the two different experiences has been so far? 'Cause your year one as you were pharmacy student, you were you're obviously on campus and essentially the bulk of your year two this year already will be an online. So maybe just

share some, any comparisons or any contrast that you've really felt so far, obviously apart from the obvious being online and offline experience.

Lecturer A:

So I think Student A did mention earlier that, so Student A, you felt that in an online, in a physical environment, you felt that you receive responses from the academics quicker or you're more comfortable asking questions in the physical setting versus in an online environment, or...

Student A:

Basically this is the point. So I don't have to worry about the late response or like a 72 hour response, they can just reply on pronto which doesn't happen actually here.

Lecturer A:

Here, meaning in a...

Student A:

In the online setting.

Lecturer A:

Okay. So Student A says, if I can just summarize to confirm what you're saying is that you feel that you get a more timely response in the physical environment versus in an online environment.

Student A:

Yeah, exactly.

Lecturer A:

Okay. All right. Thank you. Anything else that you want to add Student A, or even the other the three of you?

Student C:

I think the main thing I don't about online activities the most is how we, I know this is like this. Everyone doesn't want that to be conducted online, but I really don't like online exams, especially OSCE. I really think that's not really examining how OSCE should be conducted for me personally.

Student C:

I know this semester OSCE is being recorded, but I don't understand why last semester OSCE is not being recorded. Like normally OSCE exams are recorded. That's just one part of it. And also other than OSCE, maybe for example, in semester examinations or other examination, I don't... Examination are meant to evaluate how well we learned throughout our year or the course, but the exams kind of open book. So I don't really think that the results being released to us in this online exam, really represents how well we did. I'm not sure whether I'm interpreting what I'm thinking in the right or not. But I really think that online exam is not really a thing that I like. It doesn't really evaluate how well we learn throughout the year. Because it's open book the online exam, they will just say "this is an open book test". And then this learning material that we are learning, if it is open book. A big part of challenges are already overcome by the study part.

Lecturer A:

Okay. Yeah. Thank you for that Student C. I actually have some questions about assessments later on, but you've already brought up some points today which I'll just summarize those. At least one thing for you, compared to last year in the physical environment was that you feel that the assessments are not very fair in an online setting. If I was just to summarize what you were saying, is that correct?

Student C:

Yeah.

Lecturer A:

All right, thank you.

Student A:

Yeah, because think of it actually, what are we learning when we are in an online setting? Basically the whole answers are in front of us. We can literally just look at the computer and just do. Even at the OSCE, which we are supposed to be doing it on our own. It's not actually happening. This is not the way it works. What happens is we have a script written right beside us and we look at the script and just talk , and the patient cannot see us. Like you cannot even see if I'm talking to you or reading from scripts. So even with the invigilators just trying to monitor, but it's still not effective. It's not effective for us. So we're not actually benefiting anything. That's the point.

Student B:

Well, the obsession on part, I do think that it can be unfair because some people might look together to do the exam together, but also it depends on the individual itself. If you already tripled yourself and you want to optimize the benefit out of the exam, you wouldn't try to cheat or do any sort of things. But then again, it could be unfair to other people as well because if, let's say for example, a student who cheats compared to other students who did not cheat, the person who cheat got a higher score or whatever, and this can really inaccurately represent the GPA or when that test results are in. Yeah. So it depends on the individual. For me the OSCE one is not so good. I don't have a good experience on the previous [inaudible 00:14:45] and the OSCE. During my OSCE and the first batch that morning, 8 o'clock one and then I wake up in six [inaudible 00:14:58] myself.

Student B:

And during OSCE, my patients suddenly dropped off on the Zoom. I'm alone in a Breakout Room for like eight minutes. And then after that, I go back to the main room and then the lecturer's is like "why are you ready? You shouldn't come on there. I think I already finished, but I come up to the main, I should lift the Zoom and that came on line because they are assigned to them, to them for the next session. So I like I'm, don't know what to do and a bit confused. And after that, when I go back into the break room with my patient, again, I might cut off half. And I don't know how all my thing is [inaudible 00:15:39] already. For the assessment one like the online, because it's open book I asked what time you said that's not what our actual maps, because like, compared to the last year, the physical test that my, this year, the assessment test result is like better or improve more compared to the previous one. But not really what I get because I can just on all the tab or this other thing. And I eat one patient button that I can get all of them, just people. What we need to do is familiarize the discovery, like what section but not like understand whole thing.

Student A:

I'm not going to lie. But most of the things I did in the discovery, I had a tab for the exam and like 50 other tabs just for the information I know the exam was coming from. There was this quiz we had, I had the table summarizing the whole thing. I just, 15 out of 20... It was a multiple choice question. 15

out of 20 questions out of this quiz I got from the table I made. Yes, the table was my hard work, but still, I don't feel like this is the way it should work. I feel like there should be some effort to do.

Student B:

I would say online that assessment is assessing you on how well you can find your answers, how quickly you can find your answers within a time range and how accurate it is. Just finding the answers. As long as you are able to find the answer, then it's fine. You don't even have to understand the concept. That's what I think.

Lecturer A:

Okay. All right. Thank you. So just stay within the question again, it feels the general sense that I get from the four of you at least the main thing that you feel is the big difference between your online learning experience and your offline experience is this whole aspect of assessments. And I guess under assessments then you'll have to describe to us the different points and the different issues that you feel strongly about. Is that correct? Can I say that?

Student A:

Yeah. Something like that

Lecturer A:

Yeah. It's just from a general sense, just to contrast the online learning experience and the offline learning experience. Okay. So thank you for those really good points. We're going to start breaking down carefully, the different layers. The first one that I want to ask first. So I want to break down your interactive lectures, your workshops, and your close the loop sessions. So obviously you have no experience, your online one this year and also your offline one last year. I know Student C just now mentioned briefly about your workshops specifically, that you felt that it's just hard or you feel that it doesn't work. And then I think Poyi also did mention about group work, your workshops as well. You felt that your group mates may not be behind the screen as well. So you don't know whether they're actually doing the work that you need to do. So let's just carefully go through this first over the next few minutes, let's stick first with the interactive lectures that you've had so far. Any thoughts on the comparison between when you compare the online one and the offline one? Just your interactive lectures first.

Student B:

For online interactive lectures the biggest advantage is that you can ask questions quickly. You can just search and then the question is there. You can go anonymous. You can go non-anonymous, either way the lecturer will respond, especially when... That way it's more efficient compared to, if you're in a lecture hall, you can't just raise your hand and ask them. You can do that but if the lecturer might not see you know, so that's good.

Student A:

The joke is sometimes you can actually type of question anonymous, supposedly, and then the lecture just says your name.

Lecturer A:

Okay.

Student A:

Apparently, no reason.

Student B:

I think that's not a huge problem because if you were to do it in real life, it's not anonymous anyways.

Lecturer A:

Okay. From the other two Student C, Student B or even Student A if you have anything else to add. Just looking at your interactive lectures first, anything else to add?

Student B:

What [inaudible 00:20:27] say just now it's the flexible timeline. I can do it whenever I want. But one thing not so good it's the prerecording interactive lectures you need to answer one question before you move down to that that one. But if in actual class the lecturer ask most of the student, or some of the students, and then they got different answers. So then we can get more different answers from that. But from the lecturer you only have one answer if the lecturer answer.

Student B:

For prerecorded interactive lecture, there's also lectures that is using the previous ones. Like they are using the Australians or prerecorded ones from last year or the previous year. I didn't think that's a good thing. I prefer if you record it like this year. [inaudible 00:21:27] Instead of you hearing randomly lecturers saying "like quiet down please." Or "Can you keep it down". I know it's a bit distracting.

Lecturer A:

Okay. Understood. Thanks. Student C you have anything else to add? Anything similar or different to add?

Student C:

No. I still prefer online lectures.

Lecturer A:

Okay. All right. Thank you. So now we move now from your interactive lectures to your workshops. So..

Student A:

One last point about the lectures. You cannot record them. No matter how hard you try, you have to literally record yourself doing the attempt. I tried that once, it was boring. It was literally so boring. I don't even attempt to finish a lecture in one go. I just get lazy and just like, I'll do it later. But in the class you can just simply put your phone somewhere or your laptop and record. I used to do this for the whole last year. I used to record all the lectures, but now it's like, "yeah, it's just here." The prerecorded ones at least.

Lecturer A:

So do you see that as a benefit or a disadvantage?

Student A:

More of a disadvantage.

Lecturer A:

Sorry.

Student A:

More of a disadvantage.

Lecturer A:

So you see as a negative thing towards for you?

Student A:

Yeah.

Lecturer A:

Okay. All right. Thank you. So now going on to the workshops of your design of your course. We've had some points already from two of you. Are there any other points to add about your workshops.

Student A:

The fact that there are multiple rooms, and sometimes you go into the wrong room you just like panic. That's why I come like an hour early or 30 minutes early. Because what if I'm in the wrong room? I just keep checking for like five, six times. And usually look at the timetable, and yeah it's this room. You cannot go wrong in that room number. But in here it's all links. And sometimes the lecturer decides to change the link for no reason, for apparently no reason. "Yeah, I'm going to change the links and there you go." Why? No one knows. I just did it.

Lecturer A:

Yeah.

Student B:

When the workshops are in the breakout room, I always do them inside there. Most of us do not prefer to open the camera or open mic. We even prefer sites like Google Drive. That one is not so easy for us. We need to discuss something, but all of us not prefer to on the mic, even without the camera, even the mic they also don't want. We don't know how to discuss on that. Even type at the place, our question below, they like talking at there, but not really like my [inaudible 00:24:34], but in physical then we can discuss on it. Like the MRP there is something very important to discuss. And then for the workshop, I don't like that the lecture usually will be overtime. We finished at one to three and then the lecture one, two, three, [inaudible 00:24:53] something like this. And actually we have something to do after that but you need to like sit and wait.

Lecturer A:

So Student B can I ask a bit further? So the fact that you said about how you felt that communication was affected in the online setting, your workshops, that you seem to struggle to talk personally for you. Do you find it as a positive thing or a negative thing?

Student B:

I think it's not so preferable because it's kind of difficult to discuss like in MRP. I think our opinion Confederate, but no one in the Zoom preferred to open on mic, even if you ask in the Zoom [inaudible 00:25:45] and they will like pretend not hearing the thing or like they already leave from in front the laptop. That's why when you ask no one answer, that's the communication problem there.

Student A:

Personally speaking, sometimes during the workshop I have to go get something. So I'll leave the laptop. Sometimes I don't give notices, I just like leave the laptop just focused on something. Sometimes there's something dragging for like a good 15 minutes or something.

Lecturer A:

Okay. All right. Thank you.

Student A:

There should be something like a focus monitor. So if you're focusing it looks at your screen and sees if you're looking at the screen or not. If you're not looking at automatically disconnects you.

Lecturer A:

Okay. Student C do you have anything else to add?

Student C:

So for workshop specifically, like for example, PP [inaudible 00:26:52]. Some of them shots and then for us to get to know what, for example, the inhaler, how are we actually using it or touching it. We don't have the chance to touch it. That's fine. But even when we go to our placement in the community pharmacy. So I find that a bit unfair because some of the pharmacies that my friend went to, they have everything that they need, like they have inhaler over there. They can touch the in-haler. Or for example, they have a special blood glucose monitor [inaudible 00:27:34] plays. They have a lot of different types of patients that happens to relate to our course. For example, they encounter asthma patients or patients with high blood pressure. For me personally, the pharmacy that I was being assigned, they don't have inhaler and then they don't have spacer. And then they don't even have a lot of customers. The only customers that I encounter during the hallway is just diabetes patients and also patients with hypertension, that's all.

Student C:

And listening for my friends, describing how they learn the inhaler technique and all those things. I'm like, I can't even touch it and I don't even know how does it look like, and then I'm going to have my OSCE exam. How am I supposed to counsel a patient without really knowing how the thing looks like? I know we can just memorize the script [inaudible 00:28:34] or what kind of specific counseling appointments come in. Yes. We know that, but do we really know how to like counsel them with the real thing? Like that's quite a hard part of the workshop. For specific workshops. Yeah.

Student B:

I want to add on what Student C say the specifically. For the last time you have something made to do the actual one [inaudible 00:29:02] and therefore one of the SS

PART 1 OF 4 ENDS [00:29:04]

Speaker 1:

Oh, the actual at the lab one and then for our one of the assessment, at the hall [inaudible 00:29:06] what they want until now our previous sem haven't considered complete because of that assessment. Because of, we didn't, we can't attend the workshop and can't do the assessment, when the result out they're written that, I didn't finish. My parents is asking me, "Are you doing something wrong with your class? Or you skip the class or you fail from this sem or something like this?" I need to keep on explain to them something that because of we can't attend the actual workshop like this.

Lecturer A:

Okay. All right. Thank you for those comments. With regards to the close the loop, can I assume that your comments or your feelings about it is the same as all your interactive lectures? Just because of the, again, coming back to the question it's just comparing the offline and the online experience. Are there anything else to add for the close the loop sessions?

Student A:

I never knew they existed, actually. Did they exist last year?

Lecturer A:

Yeah. So close the loop sessions always happens at the end of each topic.

Student A:

But last year we didn't have anything like that. I don't remember. Did we?

Speaker 1:

Yeah, because during our first year, we weren't exposed to close the loop. So this is our first time actually experiencing how it is.

Lecturer A:

I see. Okay. All right.

Speaker 1:

So far, I think it's almost similar as normal interactive lectures in the lecture hall.

Lecturer A:

Mm-hmm (affirmative). So when you said-

Student C:

But I think we haven't experienced close the loop online last year and we, for this year, the start of this semester, I don't really know what's the purpose of close the loop, because we doesn't have it last year. But throughout the year, I really think that close the loop is the best part for all of them, because close the loop part is the part that we can really ask questions and the lecturer can answer us. But I'm not sure whether physically close the loop is conducted in a big lecture hall. So I don't think that's very convenient for us to ask question because like what [inaudible 00:31:22] mentioned just now, if you ask in a very soft tone or you raise your hand in a very [inaudible 00:31:30] posture, the lecturer might not notice you and you did not get the answer to it. Like ask the question. Close the loop is the best part overall for me.

Lecturer A:

Okay. All right. Thank you for that. So now moving on now, can I ask now, what do you feel about the organization of your online learning experience in semester one? So this was the first half of the year. And then whether was there any changes across when you started semester two? So some of the things that you can consider would be how the course is, how the syllabus are organized, how your timetabling is, how efficient the Moodle content is delivered? I think, Student A mentioned earlier a little bit about your communication with your lecturers already earlier, but was there anything else that you noticed? So, and this is really, we're just contrasting with semester one this year and semester two this year.

Student A:

Yeah. Why do we have some of the lectures, like suddenly we find out that we have a lecture week. Since when, how? No reason or explanation, which I find that pretty annoying. Suddenly out of nowhere someone tells you, "Yeah, we have a lecture now." Like just today, I was on my way. I was walking and then someone tells me, "We have a lecture today." So I was like, "What?" I didn't even plan for this. Yes, we might have heard about it or not. That's not the point. Still.

Lecturer A:

So are you saying that the timetable is not clear?

Student A:

Yeah. Sometimes they just change for no reason. And the change is not actually documented anywhere. We don't have something to rely on.

Lecturer A:

Okay.

Speaker 1:

Because that one [inaudible 00:33:30]. Student A, I think the timetable didn't change. Today having lecture because Wednesday is public holiday. [crosstalk 00:33:40] They just switched the discovery day to Wednesday only. But the sudden lecture, you mean, I think that maybe the Saturday, the extra class that is-

Student C:

The extra class. That one is recorded. You actually can look back through the recording.

Student A:

Yes, yes it is recorded. Yeah. I know that's okay. If everything is recorded, I could record in the lecture hall. I could record anything. We could record. The point is not about the recording. The point is about why is it so sudden? Why suddenly we have to know that there is something in the middle of the weekend, something in the middle of... something that's happening now.

Speaker 1:

Today's class, it's not surprising to me. Today's class is clearly written at the timetable, but because we used to, like Monday is discovery, they still be assume today don't have class.

Student A:

I didn't. Okay. Okay. Okay. Okay. I didn't assume that we don't have, there's a difference. We actually did... Okay. Okay. [crosstalk 00:05:34]

Lecturer A:

No. Yeah. Thank you. Thank you for that. I think I'm also asking the overall experience. So I want you all to have an overall picture comparing semester one and semester two. I think some of the examples that you all have shared so far were just a very, it could be a one or two off. I'm not sure because it's specific to your unit, but maybe if you all just take a step back and have a bigger picture of the whole semester one this year and semester two this year so far, what do you think? What do you think about the organization of it?

Student B:

I will say semester two is more structured compared to a semester one because during semester one, it was new to us, everyone. This happened, the pandemic happened so suddenly. So everyone has no system structured to help to conduct it online fully. So semester two is definitely way better, but there are some improvements to be made definitely. It's more easier for us because the timetable, the structure is all given to us way before the semester started compared to sem one. Before it's like bits and pieces of what you're going to do the next week, instead of just giving us all the details. So before the semester two started we were given the timetable, the structure, everything. So it's better.

Lecturer A:

Okay.

Speaker 1:

And even the [inaudible 00:36:03] are already written clearly and they're not like previous semester we cannot refer to the email a lot of things. But this year, most of that is already fixed.

Lecturer A:

Okay. Thank you. I think I'll move on to the next question for now. So when you all first heard about that we're going to switch to an online learning experience, so now I'm asking you to reflect back, back in March, April, when we just switched over from an offline to an online environment. Were there any concerns that you all had? That was the first question. The second question is that now that you have gone through it, do you all feel that those concerns were justified or are those concerns are still there and that we've not done anything about it? So two parts to this question, when you all had the first, when we transitioned to online, what were your initial feelings about it? Were you... Yeah. And then now that you've gone through like a good four to five months already are those problems still there, or do you think that those have been addressed?

Student A:

Personally my most concern that's still actually active, it's mostly about the internet issue. What if someone just like, suddenly his internet cuts off just for no reason. This is a problem that's actually we can't just take it lightly.

Lecturer A:

Okay. Thank you. Anything else from the three, from the rest?

Student B:

The main concern was how lab is going to be conducted like our [inaudible 00:37:50] training. And right now, I think it has shifted to the end of the year. I'm not sure.

Lecturer A:

Yeah. So I believe some of your lab sessions, which we cannot replace that will be, we are still having it, but at a later time of the year when things are possible. Are there anything else?

Speaker 1:

At this teach through online, we don't even know what they teach you and we don't know how to use, we scared about how to access the thing, the IT thing. Now, already used to it. At the beginning of March, when we start the Zoom thing, the lecturers sometimes don't even know how to record or

share screen like something. A bit of a time waste thing, like find memes about things like this, but now the lecturer, most of them already used to it. And we just go in all the tools they send. Even they have the staff teaching us or the [inaudible 00:38:50] teaching us, something like that.

Lecturer A:

And [Student Cyi 00:10:02], you want to say something?

Student C:

I think the thing are already being addressed like the schedule, that Zoom thing and also timetables. Those are already being resolved. But the only thing that I think is too, is the exam thing. I still don't think that it's fair to count this whole online thing, the assessment marks to.... How can it affect our CGPA if it is conducted online and we are learning things like that, and we are being assessed in this way. That's a little bit unfair as compared to last year.

Lecturer A:

All right. So I think Student Cyi brought that, on that note, we are moving on to our next question now, which is actually all about assessments. So if I can summarize also some of your sentiments or what some of you have mentioned already, that the main thing that you feel there is a sense of unfairness. So as a sense of inequality or not fair for your assessments in an online learning environment and the reasons for, I think some of you all have already elaborated on that. So I don't think we need to go back there, but can I also ask that, was there anything else that you wanted to share about your assessments that you've had so far in online environment that has not been mentioned already?

Student C:

I've once tried or I experienced before, when I was typing a full long answer for the subjective part and it pops up a red thing saying that I was not being connected with my connection or what, and my answer may not be saved. I have actually screenshot that notice because maybe it's because when I was doing my exam and my lines maybe drop out. So I was still typing my answer, but maybe the answer is not updated to that. I don't know, because I was not able to review back my... This one I think maybe. This, I actually screenshot the thing last time, I think. So sometimes I'm worried, maybe the answer that I type might not be the last answer because due to the unstable connection or what.

Lecturer A:

Okay. All right. So summarizing it also, it's really you feel that because of the unstable, we cannot guarantee a secure, stable internet connection and you feel that that could affect your answers and your responses for your exams? Is that correct?

Student C:

Yeah.

Lecturer A:

Okay.

Speaker 1:

One benefit, I think like online exam or online tests, it's like, [crosstalk 00:42:27] Yeah, I used my own laptop and I'm in my room by myself like this. When I'm doing, I used to my own laptop compared to the exam, at the class, that one.

Lecturer A:

MDEC.

Speaker 1:

Yeah. The keyboard is a not so... The feeling I don't like, the typing, but my own laptop is preferable. In my home myself, I was only in our room it maybe more comfortable.

Student A:

Yeah. You can have a nice cup of coffee as well with it.

Speaker 1:

Yeah. Even you can drink bean milk, if you want to with your exam.

Lecturer A:

Okay. So I guess now we are contrasting about your experiences that you don't have in MDEC, so that's the big hall where you all, there were lots of computers and everyone was typing. You could hear everyone typing and all that. So obviously having the exams in your room, you feel much more comfortable. And do you feel that you're able to think better as well and to respond better?

Student C:

No. I actually prefer [inaudible 00:14:35].

Student A:

Sorry. Could you repeat?

Student C:

Exam is meant to be that way, right?

Speaker 1:

But I think that in the exam hall everyone is typing and you already stopped, you feel like, "Eh! Should I add on longer or something", but if you're in your own room, you don't have any distractions.

Student B:

I will say doing it in our own preferred environment is better, it can slightly reduce the stress and the tension. And of course the keyboard in university it's very bulky and big and very loud. Yeah. And sometimes the exam hall is very, very cold. So if I do it in my own room, I can adjust the temperature. So it's like setting the perfect theme for my exam so that I can do better.

Student A:

I also want to point out something. Doing the exam at home is actually a very dangerous idea because I can simply just, I have not only I can just look up the answers anywhere, I can use machines, I can use... Because I used to program machines when I was young use it for to do some searches. I used to look for some machines, we can look for some specific information and then they just look it up somewhere and then I'll just be focused with another question. So I ended up finishing and I also noticed that I ended up finishing in half the time other students take. And there is no rule that explicitly says I cannot do this. So, yeah.

Lecturer A:

Okay. All right. So thank you for your comments so far. Student Cyi-

Dr. Lecturer A:

Could I-

Lecturer A:

Okay.

Dr. Lecturer A:

Sorry. Could I add, because most of you have been talking about assessments in more or less a written form, basically these SAQ, MCQ type assessments. How about these non, could we talk a little bit about non exam type things? Like, I don't know, access to workshops or things like that. What are your thoughts on those during the online implementation?

Speaker 1:

Well, the workshop, the assessments, what [inaudible 00:45:37] is saying, some of your group mates just leave the laptop open and they leave. Then you don't know what he or she doing and you got to ask.

Student B:

Yeah you can see. Because we normally work in a Google document, we can see the cursor, that particular student is not moving at all during, from the start till the end it's not moving. It's not doing anything of that thing. So... [crosstalk 00:46:02]

Student A:

Okay. I just think that this is misleading. This is misleading because sometimes you might be researching something. And if you're researching the cursor will stay still. Other times, it can just simply make like a problem that just moves the cursor left and right. Moves it in random directions.

Student B:

From my experience, that person, the cursor was at the start of the document where he types his name, his or her name and the student name form thing. And it did not move at all. And during our discussion, he did, he or she did not participate. And when the lecturer called for their name, they did not respond because left the room. So basically that student did not contribute anything. If he was to do it in real life in the workshop that wouldn't happen at all. So that's concern.

Lecturer A:

Mm-hmm (affirmative). Yep. Thank you. Thank you both for your comments. Lecturer A, do you have anything else to ask?

Dr. Lecturer A:

No. I'm good.

Lecturer A:

Okay. Student Cyi, just know when you mentioned about the point about sitting in an environment, having the, answering the question in the exam environment, we lost a few words, so I didn't really hear what you said. Can you, do you mind repeating yourselves?

Student C:

So for me, maybe I'm a bit different from others. I actually, I really liked the environment of the exam [inaudible 00:18:31].

Student C:

Us to tell, like that environment tell us we are actually having an exam and we need to take this thing seriously. But having online exam at home, it kind of creates like a very, just now what they mentioned, a very comfortable environment making you feel like that's not like how an exam normally we would take part. So maybe some people might prefer because some people would perform better in a less stressful environment, but not everyone is like that. For me, I really don't. I still don't like online exams at all.

Lecturer A:

Okay. All right. Thank you for that.

Student C:

Like a quiz, if I do it at home myself, rather than, exam is really a big thing especially those your end final exam. Maybe we can change the way how we assess students doing online. [inaudible 00:48:32].

Student C:

Still having this kind of assessment really can't. Evaluate as better... I don't know. Just suggesting.

Lecturer A:

Yeah.

Dr. Lecturer A:

There will be a time for that later. That's one of the questions towards the end of the session. So you can keep your suggestion for that [crosstalk 00:48:54] and we will ask it.

Student C:

Okay.

Lecturer A:

So I guess this also leads to my next question, which I think may be some of you all have already alluded to. So really what we are asking, hoping now to ask is to find out from your perspective, do you all feel that the assessments in an online learning setting is a good measurement of your knowledge and skills? So I think I know what Student Cyi feels about it, and I believe she thinks it's not a fair and good measurement. What about the other three? Do you all feel the same way, or do you feel otherwise?

Student A:

To be honest, [crosstalk 00:49:35] I feel...

Student A:

Okay. Go ahead.

Speaker 1:

No, you go first. It's okay.

Student A:

So personally, I feel like this online learning has tested how, it actually taught me new ways to use technology, basically. But other than that, I didn't actually end up learning actual material and I don't feel I learned much from the semester.

Lecturer A:

Okay.

Speaker 1:

The online exam, good is like my marks getting higher of course, but it also teach me how to search question from Google, within 0.0011 second and analysis this is the answer we want but that is not the actual result.

Lecturer A:

Okay.

Student A:

Oh, yeah. It actually taught me how to not depend on drawing at all. Yeah. Because now we cannot draw, we cannot scribble in the paper, since the moment they started the e-assessments, we cannot actually write anything. So it's something to be addressed as well.

Lecturer A:

Okay. And Poh Yee, do you want?

Student B:

I will say it's not an accurate representation of our marks and it doesn't evaluate our skills and knowledge, but it also depends, like I say, on yourself, whether you do it truthfully or not. So for me I'll still treat it as a real life exam because eventually we'll be returning to the uni and take part in the real exam. So I can't be accustomed to this online thing where I try to not study, I think. Yeah. And it might affect what future employers think about our CGPA maybe because they might think, "Oh, you graduated from the era where online assessment and everything for you." They might have the mentality that you cheated or whatever. So it doesn't really... It might not be a good image maybe.

Lecturer A:

Okay. All right. Thank you all for that. I think that's essentially what we have so far on the online learning platform. Lecturer A, do you have anything else to ask or add?

Dr. Lecturer A:

Not at the moment. Please go ahead and later on I will go through and if there's some gaps, I may ask.

Lecturer A:

Yeah. Okay. Now we're going to ask you, for the next part of our session. So thank you really all for your honest responses so far. The next part of your session is really focused on the way your syllabus is laid out. So I think we can say that the fact that you have this pre-class activities, which is your discovery, your Moodle activities for you what we asked you to do online each week before the

interactive lectures. And then when you come and join for your interactive lectures then we have some other activities in that session whether offline or online. It is quite unique to Monash. It is quite unique to the pharmacy program as well. And so really the next stage of our questions are really linked to that. So I guess first off, can I just start off first, what do you all think about your pre-class activities or in other words, your discovery materials?

Lecturer A:

So this is, asking you now to think about the past two years of your studies so far, because this includes the offline and the online experience. So we're really asking you what you all think about your discovery materials or your pre-class activities.

Student B:

I would say that all for our discoveries as, because sometimes it's our first time being exposed to a new topic. We haven't get an idea of what it is. So the discovery might be very overwhelming and we might not be understanding anything at all, even though we've read about it. But at the same time, it's also good that we have the discovery before our proper lecture. So it gives us an idea of what to expect.

Lecturer A:

So can I-

Student B:

But sometimes it's like too much also.

Lecturer A:

Okay.-

Student B:

To understand.

Lecturer A:

Yeah. So we have comments before about the amount of it, that it's too much or sometimes, but generally if I were to ask you to choose whether do you like it or do you dislike it, what would you say?

Student B:

I will say I like it.

Lecturer A:

You like it. Okay.

Student B:

The benefits are more than the cons.

Lecturer A:

Okay. And the other, and I think your reasons for you've already said, you've already shared that as well. The other, from the rest of the group, do you all like it or do you all dislike it?

Speaker 1:

I think I like it because before the university we don't actually study before we go into the class. But now, because of this discovery thing it's like quite deep, we can't understand the new topic but as we study, we have a bit of baseline knowledge on it. And during the interactive lecture, we know more about what we will learn and get easy to link between it. And then after that you still got shocked base, so if we didn't study before that and we just interactive lecture and then go to the workshop, I think we will have so much understand on those all good on the workshop because we didn't learn before that.

Student B:

The thing for discovery is that if you don't do your discovery, if you don't read at all, you can't go in to the interactive lecture getting anything out of it. So if you miss it, you'll be completely lost.

Student A:

Not necessarily true, but generally, yeah. But sometimes the elective work is so easy, you don't actually need to even read the discovery. Generally too.

Student B:

I don't know. Maybe I still feel [inaudible 00:56:22], we need to read.

Lecturer A:

I think this again, we're just eliciting your personal experiences. So there's no one person who's correct over the... But thank you for your comments and your sharing so far. If there's nothing else to add or there's nothing different to share, I'll proceed onto the next question.

Student C:

I actually have quite a different opinion.

Lecturer A:

Okay. Yeah. Thanks. Okay, Student Cyi, thank you.

Student C:

Not really related to the online thing. Since last year, I already think the discovery thing, or okay, maybe different person have different learning styles. Actually discovery, every time the discovery doesn't have the same content like the, but they gave us the same, they just gave us one day meant to be the discovery day, the whole day to read everything else. But every time the content is different. Sometimes it's four page, sometimes it's six page. [inaudible 00:57:33] Same timeframe to digest everything. And also I, you get what I mean?

Lecturer A:

Yes.

Student C:

If you are answering three questions during an exam, they will give you the amount of time to answer that three questions. But for discovery, I don't get why, they gave us extra stuff, but they don't give us extra time to read. And also I know that depends on our own capability to read in a short amount of time that. Yeah. Yeah, I know that, but I also...

PART 2 OF 4 ENDS [00:58:04]

Student C:

... to read in a short amount of times. Yeah, I know that. But I was focus on anything that most of the time, when I read discovery, I don't really benefit in. I don't really know what is the content really want to tell us. Until I attend interactive lecture, then the lecture tell us what we should focus and what we should take note of. Then why don't the interactive lecture happens before the discovery? So that we know which point is the important one and we don't have to refer it back to discovery again and study everything all over again. And realizing that the part that I am memorizing during the discovery day, that part is not important at all. Maybe not really not important, it's just not that significant as compared to the others. And I didn't know that I'm focusing on the wrong thing. But I cannot tell which part is the important part if the lecture is not telling me, right? Because the font size are the same. They don't really highlight anything on there.

Student C:

So I'm not sure, but that's for me. I really prefer to have interactive lectures or whatever things before the discovery happens. And then after the lecture, we know what's the important point. And then we start dive deeper into our discovery content, then we can benefit better.

Student B:

Perhaps in the discovery, there should be a-

Student C:

That's my opinion [inaudible 00:01:35].

Lecturer A:

Okay. Sorry. I think, Kayi, there's a big glitch, the last, but I think I know what you said. Poyi, you wanted to say something now?

Student B:

Yeah. On top of what Kayi has said, maybe in the discovery there should be sections. If there's extra information, there should be a type of heading saying, "optional reading," but we do have those. However, sometimes in the discovery, there's more in depth stuff, more optional reading, they did not specify at all. So this cause us... Sorry.

Lecturer A:

It's okay. Go ahead. Yeah.

Student B:

This might cause us to continue reading the discovery and further being confused by it. Because me, I'm not comprehending and understanding the really detail part.

Lecturer A:

Okay.

Speaker 1:

I also want to add on one thing in discovery there. Sometimes they have link for us to click in, and sometimes the link maybe [inaudible 01:00:40] and it's a lots of information and they just put the

link that. We don't know if we need to study it all in depth in the information in the link, or we just look through it, or what. Or even sometimes for some topic on some disease or something like that, the back office already, they put a video like yesterday, and that is consider what we should learn. Why not they just make the video as the content of the discovery, then by word and feature so we can study? And additional as a video or instead of just the video, we need to study the video? But then when we let me click in, we got lots of thing to read and don't know which is the important one.

Lecturer A:

Okay. All right, thank you for the comments so far. If I can summarize again, the general sense is that you'll have a more... From a general point of view, you feel a bit more positive towards the discovery materials. But obviously, you feel that if you were more clear about what you need to do for your discovery materials, you'll feel much better. Is that okay to say that? And again, this is a very general statement without going through all the details that you have shared so far.

Lecturer A:

Okay. So Kayi, so you mentioned an interesting point, that you felt that you would prefer having interactive lectures before the discovery materials. And from what I understand is purely because you feel that the discovery materials is too much sometimes, and you don't know how much time to spend on which area. If we were more clear on that, would you still be in preference of having the interactive lecture before the discovery material, or you will be fine having the interactive lecture after your discovery materials?

Student C:

Before.

Lecturer A:

Okay. All right. Thank you. Lecturer A, you wanted to ask something? Yeah.

Dr. Lecturer A:

I was going to agree with your summary of it. That's kind of the gist of it.

Lecturer A:

Okay. All right. Thank you. So, now moving on from the pre-class materials to your in-class activities now. So your in-class activities will obviously include whatever happens in the class, so your interactive lectures, your workshops, and your close-the-loop sessions. What do you all like about it? Or what do you feel about it? Where do you like it, or do you dislike it? And why? Some of you did allude earlier already that you like the whole interaction part in the interactive lectures, but we just wanted to hear a bit more from your... What do you think about it?

Student A:

Again, again, again? [inaudible 00:05:47].

Lecturer A:

So do you want me to repeat the question?

Student A:

Yeah.

Lecturer A:

Yeah. Okay. So now I want to know how do you feel about your in-class activities. So this would include your interactive lectures, your workshops, and your close-the-loop sessions. I think some of you have really alluded to how do you feel to some of these specific sessions, but I just like to know a bit more. So earlier-

Student A:

Actually, I like the close-the-loop. I like that close-the-loop sessions because they tend to... They just is a different way of actually understanding what's happening and having a recap of the... What is it called? The lecture. And having recap of the materials, making it easier to digest.

Lecturer A:

Okay. And I think one or two of you also alluded earlier that you like the opportunity to ask questions in the close-the-loop sessions, so that's good. What about your interactive lectures and your workshops? Do you enjoy the style of it, the design of it?

Speaker 1:

Well, the workshop we have short brief... The lecture would brief us on how to do like five minutes, but can the lecture brief more of about it? Because sometimes the briefing is just like, tell us all we can assess in some angle in [inaudible 01:05:20]. Some of the lecture don't even go through the case scenario, or some of them go through, and then with certain stuff to do. Then by finding the [inaudible 01:05:29] is quite difficult for us, suddenly this lesson. If the lecture can brief more a bit on it. Well, I will prefer after the workshop, we have a discussion on the workshop compared to after that during the closing the loop, because we will a bit forget, or if we discuss after the workshop, like everyone will submit, then I think I would have more to learn what I wrong just now and what is the lecture answer.

Lecturer A:

Okay. Thank you.

Student B:

Yeah, I would like to add on to what [Student B 01:06:10] had mentioned. It's that if the lecturer have a confusion after the workshop, this gives us more a bit of a closure instead of dragging it to the next day during the close-the-loop. Then sometimes I think during closing the loop, the lecturer doesn't address the workshop pieces sometimes, but sometimes they do, sometimes they don't. And especially if during the workshop, there's not enough time, the lecturer would just call us back into the main room and then be like, "Time's up. Okay, time to leave." And then everyone just [inaudible 01:06:47]. So it's a bit [inaudible 01:06:51] hanging, keep hanging.

Lecturer A:

Yeah. Okay. And what about your interactive lectures? Because as you, I think, alluded to earlier as well, your interactive lectures, you come in, we straight away go through case studies or talk to you about real world examples, and all of that. What do you think about it? Are there linkages to you? Is it linked to your discovery? Do you find that it's useful, or you don't like it at all as well?

Student B:

I feel like the interactive lecture is quite good because from what you learned in discovery, the interactive lecture put us the real case scenario, like think at the different way. I think that is very

good. I can think in different concept and something like this. But can the interactive lecture more a bit example, like the real case scenario so we know what actually happened once we do something?

Lecturer A:

Okay. All right. Kayi, do you have anything else to add?

Student C:

No.

Lecturer A:

No? Okay. All right. Thank you. I'm going to move on now to our next question. Again, this is a very general question. What do you think of this overall style of learning? And we compare this to a traditional classroom because here, again, you'll have your pre-class activities or your discovery, and then you come to your class and then you have all these examples or activities that you do, versus in a traditional learning environment where you just come to your class and you just learn, and then you go from there. What do you feel about these two different styles? Do you all-

Dr. Lecturer A:

Perhaps a good contrast would be comparing your friends in other universities, like in the local unis or other private unis. They probably don't have a similar thing that you do, right? They probably don't have the discovery plus interactive lecture. They probably just have maybe notes. And then after that, a normal lecture where the lecturer is basically speaking for one or two hours, but you have it differently. So could you compare and contrast this, your thoughts about this?

Student A:

I mean, you can always look at the bright side in the learning experience, but at the same time, we have to be real. So is it actually what's happened? Are we like saying, "No, we have the better one." Or is it actually good? We could be the best of the worst. So, right.

Dr. Lecturer A:

Yeah. What do you think personally? What's your thoughts about it?

Student A:

I think it's okay. I feel like we are given some stuff. At the same time, we are not being given everything. We're not given the more important things though. For example, about the point I mentioned about the lecturers not answering. What's the point if you're giving me good discovery materials, but you're not answering my questions? I'll still have doubts. That's the point.

Student B:

Based on my experience, when I first came to Monash and experienced this type of learning style, it's very new to me because during my pre-university years, I was taking A-Levels, and everything was study by the book. We go into a traditional classroom. And then every day it's just like that. So transitioning from that type of learning style to this, it's a very huge change. At first, I couldn't adapt to it because there's so many varieties, like workshops, interactive lectures. It's like everything is all over the place. But slowly, I was able to adapt to it, like working in groups and everything. So I will say it's way better than the traditional learning style, because there's more variation and we get to work in groups. There's more discussions going on and you get to work out your communication with other teammates.

Lecturer A:

Okay.

Speaker 1:

Yeah, [crosstalk 01:11:13]. Poyi, you continue. Sorry.

Student B:

I will say I would prefer if our syllabus has more Malaysian context, even though I understand that it's more Australian-based.

Lecturer A:

Thank you. And [Student B 01:11:31], yep?

Speaker 1:

Add on to what Poyi is saying, for our uni life, we've got more workshop and something like this. The one is more on presentation or we need to do out of worksheet, like we need to submit the homework on the spot like this. This is quite different from before that. And I think this will encourage more interactive and communication and something. I feel it's not only study and hearing the lecture or something, it's more interactive. I like this.

Lecturer A:

Okay. So you like it. Yeah. Okay. All right. Does anybody not like it?

Student A:

I think it's okay. Yeah.

Lecturer A:

It's okay. All right. Kayi, do you like it or not like it or it's okay?

Student C:

I don't have any opinion, because my pre-U is very different. My pre-U is like secondary school-

Dr. Lecturer A:

I think we lost her.

Student C:

... the first time I'm exposed into this kind of teaching styles, and I'm okay with it. It's just that it's has something that need to be properly, something only. But overall, it's very fine. It's very good.

Lecturer A:

Yeah. Now, we missed you after you said your secondary school.

Student C:

I mean, my pre-U is just like my secondary school, like the super traditional way of learning.

Lecturer A:

I see.

Student C:

In classroom, we cannot bring computer, anything else like that. My pre-U is like that.

Lecturer A:

Okay. So we also wanted to find out how your initial feelings about it in your first year, and whether that has changed over now that you're in the second year. So I think Poyi did alluded to earlier that you felt that initially it was quite messy. You didn't know what was going on and all that when you first started, and that was because of your previous education experience. But then now in your second year, you feel a bit much better and you kind of know what you're doing already. And is that the same sentiments for the rest of the three of you in the group?

Speaker 1:

At the beginning of the uni life, we don't have the actual text books or like this, and we just click into them, click but that we don't know what is actually at the beginning and we need to use to it and know how to search, how to get our materials, something like this. It's like at the beginning, I don't like to get reading thing, like clicking, clicking, next, next, next, next, next. And then I think now it's really used to it. It's okay now, because like this [inaudible 01:14:13] and you can have more diagram or even video.

Lecturer A:

Okay. Okay. All right. So now, I'm going to ask this because obviously the... We also want to know what caused you to suddenly maybe change how you feel about towards this style. I think the two of you have suggested that the fact that after you've gone through it a few times, then you'll get more familiarized and more comfortable with it, so that's when your feeling towards it became more positive. Apart from that was because we know that sometimes happens in life when we were given something new. Apart from that, was there anything else that triggered the change in your feeling towards it from a more negative to a more positive feeling?

Student B:

The fact that I cannot change anything, so I just have to adapt to it because I'm already here. So it's not that I can change how the module was, how the official activities are conducted. So my answer, just adapt to it. You can't change what you can't control.

Lecturer A:

Okay.

Dr. Lecturer A:

Sorry. I have a bit of a follow-up question specifically for Omah and Kayi, because both of you on the other hand were quite indifferent, right? You don't really like it now, nor do you hate it. And did it change? Actually from the beginning, was it the same till now? Or did it get better? Did it get worse?

Student A:

Personally speaking, I was very pessimistic about it. I was like, "It's not going to work." I had a feeling like... Because we actually did implement the system before back in my hometown, and it didn't work. It was like, it ended up having the same problems we were seeing right now. So I guess this is like even when this happened, I was like, "Yes, it is going to happen again." And yeah, it did happen. So I'm not actually... It's not that I'm surprised. I expected this to happen, but then what can I do?

Student C:

For me, Dr. Lecturer A, can you please repeat your question again? Because I think my line is kind of lagging just now.

Dr. Lecturer A:

Because I think for you, you are quite indifferent to what's split learning. You don't... Did she disconnect again?

Student C:

No, no, no, she's there. Yeah.

Dr. Lecturer A:

Yeah, sorry. Yeah, sorry. So you don't like all this split learning, right? Sorry, the way that it's done here, right?

Student C:

Yeah, neutral.

Dr. Lecturer A:

And so from the beginning, was it always like this or did it change? Do you actually like it even less in the beginning? Or do you find it good in the beginning, and now there's no difference or it was same throughout?

Student C:

It was same throughout. Actually, I just adapt because I cannot change anything, right? So I don't force myself to like it or dislike it. I just stay neutral, but maybe there's still... I sometimes I still find there's improvement for some different parts, but overall, I'm okay with it.

Dr. Lecturer A:

Okay. Quite similar sentiments with Poyi. All right.

Student C:

Yeah.

Lecturer A:

Okay. So I'll just continue on for now. Again, now, I'll just ask for your frank opinions. This style of learning, do you think it is useful across all the units that you have experienced so far? So the reality is that as pharmacy students, as science graduates, you get exposed to a whole heap of different things. You get exposed to fundamental science. You get exposed to physiology and biology. Then you also get exposed to clinical cases. You also get exposed to lab sessions or molecular chemistry. And then you also get exposed to things like gastroenterology, and cardiology, respiratory diseases. This style of learning, do you think it is useful across all these different types of topics?

Student A:

About the lab sessions, I realized I'm the only... Compared to other unis, I was talking with other people, other pharmacy students from other unis, I realized I'm the only one who didn't go to a laboratory now. I can't imagine... I mean, and they're still on year one. They're on year one and they went to labs. And I'm in year two, [inaudible 00:20:50], and I'm not even in the lab yet. I was like, "You guys go into labs? Wow."

Lecturer A:

Okay. So I take it that you feel that it is not useful for teaching the lab parts of pharmacy. Is that correct, Omah?

Student A:

Yeah, pharmacy is more for practical... When I joined pharmacy, I thought it's a practical subject. It turns out it's more of the theory and being with patients, which is also practical, but we don't get that practical exposure as much.

Lecturer A:

Okay. From the rest, do you all feel otherwise?

Student B:

Overall, I think it's okay because we get exposure through, let's say the pharmaceutical side, the industry part, and the clinical part as well. So it's like we can venture into different career pathway in the future. We get to know what we like and don't like as the fundamental learning.

Lecturer A:

Okay. If there's nothing else to add from the rest of the group, now I'm going to ask another more sensitive question. Given a choice, would you choose to learn in this style of learning? And again, it's a yes or no answer. And obviously, if yes, which you would like. And if no, also why, why no? So across the four of you, if you had a choice... I know a lot of you are saying that, yeah, you're already in here, there's no choice, continue on, but if there was a choice, would you choose this style of learning or not, and why?

Student C:

Do you mean by choose, to still choose Monash or not? Is that what you mean or do you mean by that?

Lecturer A:

Well, I guess-

Student C:

Or the study system?

Lecturer A:

The study system, most of the study system, because there are other unis that also do what we do here. So I wouldn't say that just to Monash. I would say specific to the style. I know we give you discovery materials, then come for interactive lectures and then have workshops, versus the traditional style of learning where you come to a lecture, you listen. And then after you go to a workshop, and you apply.

Student C:

Actually, I haven't experienced any of that traditional one before so I think I cannot give any opinion for that.

Lecturer A:

That's a very honest answer, Kayi. Thank you for that. Anything else to add from the rest?

Student B:

Based on my experience, I would prefer it this way than to traditional learning. Just by doing this type of learning style, I get to really enhance my other skills, presentation skills, teamwork, et cetera. And I couldn't think of any other way to really conduct the degree.

Lecturer A:

And so-

Speaker 1:

Mine is same as Kayi is saying. I didn't go through the another way of learning how to do a degree life. Actually, we just have Monash.

Student B:

Yeah, I don't know how other unis are conducting the pharmacy program as well, so this is just my opinion on how Monash is doing.

Lecturer A:

Yeah, sure. Okay. All right. Thank you. If there's nothing else to add, I'm going to the final section of questions that we have for the four of you for this afternoon. Again, a very general statement, we just like to know now how the learning experience has been for you compared to your pre-U or to your high school.

Student C:

To me, the experience for year one is great, but for year two is not that great.

Lecturer A:

No, comparing with your pre-university and your high school?

Student C:

Yeah, comparing it really like... Because year one and year two-

Lecturer A:

Oh, I see. Okay. Sorry, go ahead. Go ahead.

Student C:

Year one and year two is really different. They're really different. So year one is great, but year two is not.

Lecturer A:

Okay. Thank you. The same for the three of you, the other three?

Student A:

I think it is more interesting than year one, to be honest. Year one was more biology-related. Yeah, I don't like biology. Personally speaking, to be fair, I don't like biology, so my opinion can be a little bit biased. But yeah, I don't like biology, so I'm not a fan of actually dealing with the science behind everything. Just like chemistry and how the medication works, that's easy for me. That's easy for me.

Student B:

So sorry. Are we comparing to high school or comparing year one and year two of our university life?

Lecturer A:

So comparing your university life so far with pre-university or high school. Yeah.

Student A:

In that case, I think pre-uni is easier and it was more actually, I want to say more chill because I have to take four years into two years, so that's not [inaudible 01:24:40]. I think it's more manageable. I was able to manage everything, but now, no.

Lecturer A:

So more manageable than pre-university, is it?

Student A:

Yeah.

Lecturer A:

Okay. Yep. Sorry, Lecturer A?

Dr. Lecturer A:

Now, what kind of pre-university did you do?

Student A:

A-Levels.

Dr. Lecturer A:

A-Levels. All right. Just have to check.

Speaker 1:

For me, because my secondary school is [Malaysial 01:25:12], the normal SPM Secondary School, which is all paper-based, for me, I'm learning Malay Science. The science is language of Malay. Then during my pre-U and degree, I got to relearn all the thing again. And then from the book one to the online style, that one is quite different, but I think online is okay because more convenient. And for me, my word is quite horrible, like can be [inaudible 01:25:50] being rushed. During the uni, we are mostly typing. So I think it's okay for me, and we have something called correcting the spelling of the word.

Lecturer A:

Okay. Anything else to add from anyone?

Student B:

I was taking A-Levels as well. And like I said, everything is all by the books. You have to study everyday just looking at the book. As long as you follow everything from the book, you are able to ace your exam. So that was how it is. That is what A-Level was. I'm not sure about other pre-Us. So for degree, it's more on application. And because of the learning style, the [inaudible 01:26:41]

interactive lecture, so it's more fun and less boring to the traditional way of learning. So if we could incorporate this into, let's say high school, I believe it would be more beneficial.

Lecturer A:

Okay. All right. Thank you.

PART 3 OF 4 ENDS [01:27:04]

Lecturer A:

Okay. All right. Thank you. And then now I'm going to ask you as well about your discovery materials, because some, I think a few of you just now mentioned that sometimes you have a lot, sometimes you have a little, or sometimes you don't have enough time. Sometimes you just don't know what you're supposed to be reading. In your personal opinion of your experiences so far, how many hours do you think should be allocated to these discovery materials or to this pre-class activities? Just an average, give us a range of what you feel.

Student B:

Yeah. I'll talk about my own pace. So to stick like fully at full discovery material, I usually take it in three rounds. The first round, I just quickly glance through the discovery and this approximate takes one hour to read both 1.1 and 1.2, et cetera. Another one, I just take the discovery materials and paraphrase it in my own way and copy it into a PowerPoint slide show. This also takes two hours. Then the third time, I take the discovery materials and I write them on a copybook, that takes an hour, hour and a half. So I think four to five hours is actually, for each one is good. So overall we should be getting at least eight hours for the whole day for only two subjects.

Lecturer A:

Do the rest of you feel the same, or you think less or more?

Student B:

I think it really depends on how the person study, like the way they study. We cannot really give a specific time how much is really required for that. Different topic really have different content inside, we cannot... Sometimes I finish my discovery quite fast, maybe three hours. Sometimes it takes more than that because they have extra link and extra resources that we need to click into. So, if that occurs in our discovery, of course we need to devote more time. Yeah. It really depends. I don't [inaudible 01:29:30] of how much I need to study my discovery. It varies every week, sometimes, maybe two days are not enough. Sometimes, sometime, usually you need to delve deeper into what they really, and then apply it on to your own works and that's the hardest part.

Student B:

Yeah, I agree with [Student C 01:30:37], it depends on the materials. Let's say for professional practice, there are some sections where it's common, basic knowledge. Like how to communicate with patients though. So what did discovery you just browse through it, it's very fast. Sometimes the content is more detailed and more in depth and you have completely no idea what this is all about, is new to you. So you might need to slowly read word by word on what they are trying to convey. I can't really give an estimate on how much time I need. It depends on my attention span as well.

Lecturer A:

Okay, thank you. If there's nothing else to add, I'm going to proceed on to the next question. We want you now to give also an honest answer for yourself. This style of learning that you have right

now, this discovery and coming to class and then workshops and all that. Do you think this can, should be introduced later in your uni or do you think we should start it like this way from year one itself? So right now we, you obviously get exposed to it straight away from day one year one. Do you feel that you think that getting it introduced to it in like year three, for example, do you think that will be better? I know it's a hypothetical question because you've not gone through a different style of learning or to compare or to contrast, but really we just ask, we want you to answer based on what you have gone through already and what you know about your program. Are you happy being exposed to it from day one, for example? Or do you wish that you were only introduced to it later in your program?

Student B:

I don't actually like being exposed to it day one, because when somebody talks so much that you're going to University, they're like, "yes, I'm excited to see what's near the university." Then you tell them, no it's online. So it's a let down actually, especially like there's nothing you can do. Can just watch...

Student B:

I think stuff from year one, I'm using my Vista on discovery or something like, this method is quite good, because when you go into the degree, you already tell yourself that there will be different compared to your secondary school. So you know what you're going to face. Then if you study at your three or maybe later, then you need to spend time to used to it from the old model to the new model. The one is like... and then you need to spend some, maybe one cent or one month to used to it again and all those summit things, somewhat summit things more or something like that. You start from year one, I think it's okay, because year one is not so strict, yet the lecture or something like, they will teach you more about your three. The lecture will spend more time on how the class content or something, compared with teaching you how to use the project or something like that.

Student B:

Okay, sorry. I think being introduced during year one is okay, but it can be a bit overwhelming for year one students. I remember being confused using Moodle, navigating through Moodle. So I can imagine the year ones now being really confused, because it's online. Plus they have to navigate through Moodle. So it's really confusing for them. If the university can provide a crash course on how to use the Moodle, that will be great. Specifically for pharmacy degree.

Lecturer A:

Okay. All right. If there's nothing else to add from anybody else, I'll move on to the next question as well. Now this is reflecting what you have gone through so far and reflecting on yourself as a person. Do you think this style of learning has helped you to adapt to new situations better? I guess, I don't know if any of y'all have a lot of friends from the other universities around, just contrasting yourself. Then the... because, we know that our learning experiences help us unconsciously shape us, in terms of who we become. Do you think that this, going to this style of learning has helped you to adapt or to be more adaptable as a person?

Student B:

For me it's a yes.

Lecturer A:

So I see Student C nodding your head also, so I think that's a yes.

Student B:

[inaudible 00:08:29].

Lecturer A:

Student A, neutral, yes, no?

Student B:

Neutral.

Lecturer A:

Okay, thank you. All right, thank you.

Student B:

Could I follow up? Why, why do you think it makes you, if yes, why does it make you more adaptable? If no, also why could you just briefly explain?

Student B:

Yes, because we like the same statement. We cannot change anything. So we must adapt to what we receive, [inaudible 01:36:00] what we have to do now, so it's more like a growing up. You learn how to adapt. Rather than changing it because we don't like it. I don't know.

Student B:

Yeah, I would say being that the fact that we cannot change anything. So initially I was quite upset or angry because why, why is it like this. I'm so not used to it. I don't like the unfamiliarity, so it's very out of my comfort zone. That [inaudible 01:36:37], right now if anything that occurs that is new to me, I couldn't do anything. So I would just adapt. So it shifts my mindset to think that I can't change anything. Just adapt, look at the positive side of it. Yeah. Try to get the most out of it. So it shifts my mindset. So that's a good thing.

Lecturer A:

Okay. Student A, yours was a different answer than the other three. Why?

Student B:

Yeah. I don't think it actually changed my... it's an obstacle, just go past it easily. It's not that troubling for me, to be honest.

Lecturer A:

Okay. All right. Thank you for that. Lecturer A, anything else to add or ask?

Student B:

Not from this one, no.

Lecturer A:

Okay. Now we're going to ask a few more questions. We're going to end it already. I know some of y'all have recommended some recommendations already, but was there anything else that we missed out that you said that if there's anything we asked you today, if there's anything that you'd like to suggest to improve the overall curriculum, and that would benefit you as a student. Or

something that you would prefer? Did we miss out anything, because I know some of y'all have mentioned some points already in the past two hours. Was there anything else? So some of the things... [crosstalk 01:38:09] Oh, sorry.

Student B:

No, you could....

Lecturer A:

Okay. So some of the things that we shared already was that, maybe the discovery materials a bit more clearer in terms of how deep that we want you to go through. For starters, number one. The other thing that I think one of y'all did mention that you prefer having interactive lecture before discovery materials, that's number two. Number three, I think some of y'all said that you all like to close the loop session. So that should be that we can continue that. I think some of y'all said that the lab sessions doesn't really suit the style of learning. Maybe we need to think of a different way to run our lab sessions. Was there anything else?

Student B:

Will our opinion would be taken into consideration?

Lecturer A:

Well again, the purpose of this focus group is within a bigger project. So whatever you share, we will just put them together and compare that together with the other focus groups they will have as well. To we cannot promise you that it will be something that can happen next year, for example. At least this is your, if there's anything you want to say, this is one of the chance of your platforms to share. I know a lot of y'all will feel that, okay, we are already in it, we can change it. Yeah, so this is just one platform that you could express your thoughts and then we'll see what can happen from there. Again, we're not promising anything, so if you don't have anything else to say, that's fine as well.

Student B:

May I suggest that for our placements, we get allowance perhaps for our transportations fee. Or maybe... because some of us are placed at placement sites that are really far away from our home. So it's a bit inconvenient, especially if I'm staying in a hostel. To get to my placement site, I have to pick the [tram 01:40:27] because going there by public transport is impossible, and the tram is very expensive. Like hearing it, without hearing it, plus you in peak hours. There is no reimbursement or allowance for it. So I have to spend, let's say $70 a day just for transportation fee.

Student B:

Personally, I agree with her point. Some of us are placed in places, it's far away. Some others are placed in others, which is fairly new. It's like a site...

Student B:

We couldn't find a swap. Someone who's change our destination, then it can be very troublesome.

Lecturer A:

Okay.

Student B:

Another thing is, can we have the hard copy of APM lecture one.

Student B:

Compare the library on if you have that, and during the SS model of them, most of them, I think for the APM thing, as the library, but compared to the real hard copy.

Lecturer A:

We think as projectory researchers, we have to think a more neutral view towards these things. If I were to summarize it, can I say that, because of this style of learning, you would like to have more better access to resources, for example, your APF, and then you also liked to have resources to help support you in this style of learning. For example, travel allowances for your steps, activities. Okay. So I'll just say that for as a summary point. On that note as well, can I also just, I just thought of this as well, because, due to the design of our program, you guys actually have a lot more placements and in this place, meaning steps, compared to the other uni programs in other universities. You also get introduced to this placements much earlier in your program compared to other universities as well, deal like it, or deal don't like it.

Student B:

Yeah, I like it. Right. It really depends on the pharmacy that we are being assigned.

Lecturer A:

Yeah, agreed.

Student B:

I'm not repeating the location thing again, but I'm referring to the pharmacy from that branch.

Lecturer A:

Okay. There are some things we cannot control, but we take that into consideration and I believe that those points, you're not the first person to have shared those points, and that has been reflected towards the steps coordinator already, but we asking more of a general sense. I guess Student C is more of a positive feeling towards it. From the rest of the other three, y'all feel more positive, negative, or just neutral?

Student B:

Yeah, it's just neutral.

Lecturer A:

Okay. You're the neutral man, Student A. It's okay, again we're all entitled to our different opinions. The other two of your deal, what do you all feel about it?

Student B:

I like it, the fact that we are exposed more early on, because it gives us an idea of what to expect and how the pharmacy role plays in the real life setting.

Student B:

I agree as well, is like we get normally more earlier than... We maybe we know after our, what we know, this section, we actually really interesting like community pharmacy, or hospitals and things like that. We know about what we learn in the class or that not actual applicable at the real life. We know what we should more eventually, and even the off key question is not going to happen during the real life.

Lecturer A:

All right. So we have arrived to the last one for today, we've got about 11 more minutes. We've talked a lot of things for the past two hours, and I really do thank you for your honest responses and your feedback. I just really wanted to ask, maybe now we take turns at each of yours, just share. What do you feel was the most important thing that was discussed today. Or what's the one thing that has strikes you or surprised you the most from the things that we've talked about today, and it could be anything?

Student B:

The [inaudible 01:45:13] are actually happening a long time ago.

Lecturer A:

Okay. The other three, what struck you or what surprised you?

Student B:

I just suggest all the recommendation, like the interactive lectures. I should think something, the idea is quite good.

Lecturer A:

You think it's quite good. Okay. So having the interactive, lectures before discovery materials.

Student B:

No, no, no, all the recommendation, based here is very good.

Lecturer A:

Oh, sorry, okay.

Student B:

That's fine.

Lecturer A:

Okay. All right. For [Poyee 00:18:58], anything that struck you, or surprised you?

Student B:

Not really.

Lecturer A:

Yeah, that's fine, that okay also. All right, thank you. Student C?

Student B:

Nothing surprised me.

Lecturer A:

All right, thank you. Again, we recognize, I think one of y'all did mention, all of us learn differently and we all have different prior education experiences as well, because we all come from different parts of the place. What we are trying to do is just trying to listen from you guys and then compared

that with what we are offering you here in the wellness unit pharmacy program, and obviously to see how we can improve from that. Lecturer A, do you have anything to ask or to add?

Dr. Lecturer A:

Yeah. Okay. There was one question, so I was actually taking down notes whilst you all were telling me what kind of talking in, and there's one question I think. I'm not sure whether, based on the answers, I feel that maybe you didn't really get the question. It's on the one where we asked on, would you think that this system of learning is useful for all types of subjects. So, because we have some subjects that are very basic science types, how body works, how medicine works. We also have some subjects that are quite clinical, critical care. I mean, comprehensive care and things like that. Do you feel that this system works for all types of subjects? Or do you think that it's better for some types more than others?

Student B:

I feel that sometimes, some types, more than others. That's what I feel.

Dr. Lecturer A:

Would you think which types it's more suitable for, or for which it's less?

Student B:

The ones who are already accustomed to the online learning, the ones who were born in a more electronic friendly in the environment.

Dr. Lecturer A:

So you're talking more in terms of tech savviness, like someone who's already familiar with it. This is most suitable for those who are more tech savvy. Okay. Right, which is a fair opinion. Yes.

Student B:

I don't think any other types would actually benefit.

Dr. Lecturer A:

Okay. For the rest of you, do you feel the same that it's equal across? It doesn't matter what type of subject it would still more or less work or it's equally good or equally bad.

Student B:

Are you talking about whether we're comparing online learning and traditional learning or the style of learning?

Dr. Lecturer A:

Yeah. [crosstalk 01:48:53] I mean, you have your discovery plus your, IL thing like that. Do you think this will work well, whether it's on a very science heavy subject, like your, how body works. Would it work just as well if it were a comprehensive care topic? Do you think it's equal across or is it better for one kind of subject more than the other?

Student B:

I will say for our comprehensive care units, the clinical workshops are very helpful compared to our year one, more on the biology chemistry. The Workshops are okay, or maybe there's a better way to conduct it.

Student B:

I think for compare for comprehensive care and keeping the professional practice, not necessarily sensory, like meaning discovery, than intellective lectures than workshop and CPL. Comparative care, the topic is quite heavy. Well maybe we can have more the intellective lecture, but for the professional practice, the workshop, I feel like meaningless or like not necessary or compared to that might not be changed and workshop to something like the Zoom IBS. Then some of the synchronous one the same. We have done the workshop that not the Zoom one and just combined it. I feel after I've found that after I do it, I actually don't know what I learned. Compared with it, the lecture, which he knows what he's talking to us is better.

Dr. Lecturer A:

Right. Okay. Yeah. Anyone else has anything to add on this topic on suitability of the learning style? Two subject. Okay. If not, then back to your [Wayjin 00:24:01].

Lecturer A:

All right. It has been a long afternoon, but definitely very thought-provoking and we really do appreciate your thoughts and your feedback. As you'll be aware, we also be running focus groups with the other groups of students as well. Then we will compile this data together and to submit this to our school education board. So thank you again for your time. Do wish you all the best for the rest of your studies and your semester. I think I'll be seeing some of you a bit more frequently in some of the other units, as in the weeks to come. We will also get in touch with you and how we will pass to you, your 15 Ringgit gift voucher. So it's obviously be, we have to plan that properly to when we can see you the past year, this vouchers as well.

Dr. Lecturer A:

I guess your next live session is during HMW three labs right, so that's the most likely time that we can pass you what needs to be done at that time.

Student B:

Sir, I'm already on campus now. I'm already on campus [crosstalk 01:52:15] ..

Dr. Lecturer A:

Well if you want to, pass by and you just need to sign off and we'll pass you the voucher, no problem. If any of you come to campus, you can also contact us and tell us, that would also work. Actually, I need time.

Lecturer A:

Yeah. So just let us know. Okay.

Student B:

Can I add on something?

Dr. Lecturer A:

Yes, please, please.

Student B:

I noticed that we have not touched on PLP. Our reflective sessions. The concern that I have is that I will, our PRP sessions are assessed, and we are given max for it. The bright side is that it can be

considered as free max to us, but at the same time, I think it's a bit weird that it is assessed. For my lecturer, for my mentor, we are given marks based on how well we write our reflective practice, our reflection. He's the one assessing whether it's good or not, but I think based on what I feel, my reflection should be a reflective of what I think. If I cannot convey what I'm trying to say, he might... [crosstalk 01:53:56].

Dr. Lecturer A:

Are we in a Bowling Alley?

Student B:

Well there are people playing pool. If I cannot convey properly what my reflection is, he might misinterpret it and then I might not be given marks for it. That's the only concern.

Lecturer A:

There was a survey that was sent out by Dr. Juman on specifically about the BLP, have you answer that?

Student B:

Yes. I did.

Lecturer A:

Yes. Okay. All right. Thank you. Whatever you said right now, also will put that in consideration as well. I'm conscious about the time. Student C just have to leave already. I didn't want to take too much of your time already. If there's nothing else to add. Yeah. We'll end here and I'll stop the recording.

PART 4 OF 4 ENDS [01:54:55]

Deidentified FG2Y2 - Sept 2020

Lecturer A:

Okay. So, hopefully we won't go longer than 4:00 PM and we really hope to cover just main two topics today. So, first topic really will be focusing on this whole aspect of online learning and then the second part of the session, we will focus on the design of the program. So, you have your discovery, your workshops, your interactive lectures, that's all will be parked under the second part of our discussion. So for now, we will focus on the whole online learning part. Nice and easy, I just wanted to ask you all a general question. How has the online learning experience been for you so far? Anybody who wants to start? So, just feel free to unmute yourself and yeah.

Student A:

So I'll start first. So, definitely I prefer physical classes more than online classes. I think everyone agrees that because we get to ask questions on the spot and then, it's easier to clarify what we don't understand instead of expressing it in words. And then, it's always better to can see the person you're talking to face-to-face instead of looking at camera. And then, I can understand that for the lecturers, it will feel very awkward if the students don't want to switch on their webcams or if no one answers when the lecturer is asking a question. So, I feel awkward for them as well so I definitely prefer the physical classes. But the academic part, I think it's the same because we study the same discovery and the same lectures and the same content. Yeah.

Lecturer A:

Thanks. Thanks Student A. Anybody else?

Student B:

So, I do agree with what Student A have just mentioned, but I would like to add on. So, I feel that online classes have one benefit over physical classes because last time what we used to do is during lectures, the lecturer would actually show the slides on the big screen and we have to take pictures. And if your camera quality isn't that good, you often miss out a lot of important information, especially if the diagram is really small and really specific.

Student B:

So, I find it easier because through interactive lecture online, we can actually screenshot the important images that we need and that really helps me a lot in my studying because I can make sure that I didn't miss out any important information and I can refer to each and every single page because it's separated by pages instead of a whole single lecture recording, which I always find it very overwhelming to actually listen to it. Yeah. But I honestly believe that it is easier to ask face-to-face questions for me. Yeah.

Lecturer A:

Okay. Thank you. The other two, have you all got anything else different or similar?

Student C:

I do agree with both of them that asking face-to-face is easier but I still have something to add on, it's like, I do feel that online, I have more time. Yeah.

Lecturer A:

Can I ask, what do you mean by more time?

Student C:

Because there's no need to... The travel time and for example, if lecture times, normally if you have [inaudible 00:03:30] and then maybe example [inaudible 00:03:35]. The 30 minutes break in between is like somehow hanging there, we don't really have time to do something but then yeah, it's due time but then at home it's like the 30 minutes, that you can just leave the Zoom and then do something and then just come into the Zoom again. Yeah, so something like that.

Lecturer A:

Okay. Thank you. And-

Student D:

For me, it's quite similar to [inaudible 00:04:02] because like from [inaudible 00:04:07] is quite far, I have to wake up early do to the traffic jam. It's quite convenient for me to go online classes. And I'm not sure if it's better [inaudible 00:04:21] internet, it's easier to type up the questions, I feel like online classes, just like encourage us to ask more questions because it's like a when we really face some problems when we're reading discovery, we can just immediately go to email and type out. We don't need to like wait during the interactive lecture classes only we have to ask the lecturers. I feel like the lectures have more active in replying emails to our students.

Lecturer A:

Okay. So I didn't hear you very well, but just to confirm, did you say that you prefer the online learning because you felt that you could ask questions whenever? You're more flexible in asking questions, is that what you mean? Or

Student D:

No, I don't... I prefer physical class, but that I feel [inaudible 00:05:21]. I'm not sure it's better because it's [inaudible 00:05:24] or because it's more convenient to email lectures, I feel like we are more encouraged to ask more questions using email or using the forum in the discovery, in the Moodle, sorry.

Lecturer A:

Okay. Okay. All right. Thank you for that opening one. So then the next question really was to ask, how does this compare to the offline experience? So meaning when you were in your physical classes, but a lot of you all have really made some contrast and some comparisons there. And I just like to maybe to zone in and just explore that just a little bit further. So, do the four of you all feel that you can ask questions better in a physical class over online learning? Is that the sentiment? Yeah.

Student C:

Actually it depends because I think for the physical time, it's just to explain that when you want to point to a diagram to point at that position and then seek clarification for that part. And maybe if the lecturer explain, you can add on to what the lecture explain and [inaudible 00:06:49], it's just to do it that way. But then for online, I think the easier part is that when a lecturer ask questions, if you are shy, you can like [inaudible 00:07:00] and answer.

Lecturer A:

Mm-hmm (affirmative)

Student A:

For me, I feel that physical classes are definitely easier to ask questions, because for example, the CC units, especially CC4 and CC4, a lot of the lectures are recorded. So if I have a question, I'll have to wait until I finished the whole IL or like during the interactive lecture sessions, then I can ask on the spot, but I am the person that likes to ask the question on the spot, when the lecturer is talking and I interrupt them, say excuse me sir, I have a question. So I feel that it's easier and I will not forget the thing that I want to ask. Yeah.

Student B:

For me, it's somehow similar to [inaudible 00:07:51] and Student A, but because I always take time to think through my questions before asking. So to ensure that I'm actually asking something that I really don't know.

Student B:

So what I feel that by sending messages, I could actually formulate my question before I actually send it up so I could read through it that I think is advantage of using online, but to clarify concepts, especially how a process or a specific diagram, I prefer physical. Yeah. But for small questions, it's easier if it's typing out. And if it's a huge diagram and concept to understand then I prefer physical.

Student D:

For me is during the workshop. Like if, for example, I'm discussing with my workshop group mates. And if we reach a point where we don't really understand the question, it's very convenient to ask the lecturers at the spot. Like, how should we answer this question? Is there any way to deal with this? Because... Online normally it's harder because the lecturer had to go to another Zoom for another breakout group and then they are like very busy, with different, different breakout group. So it's like a bit messy in that case. For online one I prefer is, yeah, it's very convenient in the sense that if you have any questions in the discovery, or after you read the interactive lecture, you can just copy paste into the email so that the lecturers can know what to answer.

Lecturer A:

Okay. Thank you. Student A and Student D brought up two interesting points, which I would like to explore a bit further, but I will start off first with Student D's point, which is also linking to my next question. My next question really is to ask, how do you all feel about the online delivery of your interactive lectures, your poster loops and your workshops, contrast this to your offline experience and why? So if I can confirm what Student D briefly mentioned earlier, you particularly didn't like how in your online workshops, the lectures had to move from one breakout room to another breakout room, and there's a time constraint on that. So it doesn't give you enough time to ask the lecturer more question if you had, is that correct?

Student D:

Yes, it's correct.

Lecturer A:

Okay. So contrast this to a physical workshop that you had last year, I could just be standing there on the table facing three tables, and I still be able to address the questions if you have any questions, is that correct? Okay, we lost Student D for a bit there, but nevermind. Yes. Okay. All right. But anyway, from the other three, any thoughts, any opinions to that? So really contrasting now your physical, [crosstalk 00:11:20] sorry, go on. Yeah. Student D?

Student D:

Is it me?

Lecturer A:

Yeah.

Student D:

Oh, okay. I feel like the breakout room, sometimes the students keep the lecturer inside their breakout room because they have a lot of questions, then the lecturers will stay in that room for quite a long time and everyone like... Because they don't know whether the other students in the breakout rooms will have any questions. It's a bit, yeah. That's problem, I think.

Lecturer A:

So you feel it's a bit unfair. It can be a bit unfair.

Student D:

It's not unfair. It's like we have questions, but we don't know when to ask because the lecturer is not yet in our room.

Lecturer A:

Understood. Thanks. Thank you for that. Anybody else? Yeah.

Student A:

Okay. I just want to add on like, one thing that I observed during physical classes is almost after every lecture and after every workshop, some of the students will not rush home or rush back to other places, but they will stay back and ask questions until like maybe extend an hour or something. And then the lecturers would stay back and explain. But in Zoom, I understand that actually we have a lot of classes, like compact classes and then the lecturers have to do other stuff. So usually we will end as soon as possible. So I think this is another disadvantage of online class. And not everyone gets to ask questions because just like what Student D said, we are not sure when to ask and yeah, but that's not anyone's fault, it's just the limitation of online class.

Student B:

I think onto Student D's point of online workshop, it's not always the case, but sometimes when your teammates are a bit less responsive and you can't see them face-to-face and actually talk to them and interact with them, it's quite hard to discuss, especially in sharing of information where you can't just say, here, so look at this info. And secondly is the line problem. I think it is my site, but sometimes I drop off the workshop groups and I have to wait and do the lecturer add me in and if it's an assessment workshop is quite nerve-wracking. For online CTL, this semester, I think we end quite on time because we usually have another class coming up. But last semester, we have a lot of dragging off time for lectures where people actually stay back and ask questions. Sometimes I don't want to miss out on the question so I would stay in, but I feel like sometimes it drag on a bit too long and some other people will not be able to get the information needed. So there's pros and cons on both sides.

Student C:

I think for me [inaudible 00:14:28] the prerecorded ILs because I find that some prerecorded Ils, actually it's very short, there's actually nothing much in them. Maybe three minutes and then just one diagram and then the prerecording is done and then I'm like, was this it? Yeah, so it's just yeah,

because [inaudible 00:14:55]. Some of them are very informative, but some is really really short and really there's nothing in it. So that is, I think one of the disadvantages.

Lecturer A:

Okay. On that note, can I just now ask also so, because I know you've had of different types of interactive lectures so far, you've had a live, a strict, standard one hour, ILs versus you have prerecorded ILS, which are broken down into maybe four by five minutes or five by 20 minutes, depending on the units. Am I correct? Yep. Okay. Which do you prefer?

Student A:

I definitely prefer the live one.

Lecturer A:

Okay.

Student A:

Yeah.

Lecturer A:

The other three?

Student C:

Me too.

Lecturer A:

Okay. And [inaudible 00:15:52] and Student D?

Student B:

Oh, I prefer pre-recorded IL but I definitely need one live lecture. So if IL is prerecorded, I need a live lecture so I could ask questions. So such as CTL.

Lecturer A:

Okay. The prerequisite lecture, wait Student D sorry. [inaudible 00:16:14], your prerecorded lecture. Do you mind having it in the whole hour or you prefer like a four by 10 minutes or five by 15 minutes, for example?

Student B:

I prefer five by 15 minutes.

Lecturer A:

So broken down into small chunks? Okay.

Student B:

Yes. So, I can refer be feel back to each session.

Lecturer A:

And Student D, sorry?

Student D:

I think I prefer, if chapter 4.1 and chapter 4.2, I prefer chapter 4.1 is the prerecorded one. Then the chapter 4.2 is the live session. So that any questions in the 4.1 can be asked in the live Zoom in the 4.2.

Lecturer A:

I see. Okay. So you're a bit like [inaudible 00:17:11] style? Can I say that?

Student D:

Yeah.

Lecturer A:

Okay. All right. Thank you. So, thanks for that. I'm just going to proceed to the next one now. Now contrasting, just thinking about semester one this year, and this was from March onwards when we flipped everything online and compare to, and semester two, what you've had so far, what do you feel about the organization of the whole online learning experience so far? So when I asked you about your organization, I'm referring to things like your timetable, like your announcements, like your communication, generic announcements from the course coordinators or from the UCs. And so comparing semester one and semester two this year, are there any improvements in semester two? So, yeah.

Student C:

I think that some things are more synchronized and systematic for this time because for last time, an email was [inaudible 00:18:18]. And so it was like, people get confused, which Zoom link belongs to which, because it was just randomly sent email, but this time they put it all in a Google doc, so it's very easy to search for it and apply it.

Student A:

Yeah. I also agree with what [inaudible 00:18:40] said, there is a significant improvement in the organization for central units compared to SEM one where the lecturers don't email us the Zoom links and announcements very last minute, like for example, midnight or 1:00 AM. So yeah, I think this is better now.

Student B:

Yeah. I also agree on that and I realized that more and more lectures are encouraging forum discussion, which I feel really useful.

Student D:

I agree with all the answers and I feel like the lecturers, they know how to meddle around the Zoom, so it's like more [inaudible 00:19:30] classes.

Lecturer A:

Okay. All right. Thank you for that. So generally, to summarize, definitely there has been an improvement in semester two and you all felt semester one, it was just a bit messier. Just, yeah, but definitely improved in semester two though. Thank you for that. The next question is a bit interesting one. What were your... So when March happened, when we told you all we're all going to flip online and all that stuff, were there any concerns that you all felt personally? That's the first part of the question. The second part of question, are those concerns justified, as in are those concerns still

there today, now that you've gone a few months of online learning? So two parts to this question, when we flipped the online, one with your initial feeling about it. And then the second part of the question, today in September, do you still feel the same way about it, or? Yeah, so.

Student B:

I could start first. So, some concerns I have when we first go online is, first how our classes going to be carried out, such as lab and also our usual lectures. Secondly, is more personal, is like costs, how is the cost going to be calculated? And lastly, I'm worried because I am a person who actually use physical notes a lot. So I'll not be able to go print my notes and NMT. So regarding your second question, is the problems addressed for the classes. I can see that we actually postponed the net classes and we carry this up online through Zoom. Costs, I don't think there's a reduction. Well, notes, I now opt for electronic notes, which I find very organized and systematic even more than my physical notes. So yeah, most of my concerns are addressed.

Student A:

For me, at first, when the university announced that they are going online, I was very disappointed, but I'm also very happy because I'm not from Solano so it means that I have to fly there to study. So I can study home, of course, it's a very big plus for me, but then I feel very disappointed because of course I prefer physical class or why else I was flying to that place to study. And also, yes, the cost, because online classes are not as interactive or as immersive as physical classes. So I also worried about the cost.

Student A:

And then I also worried about the hostel accommodation and stuff, because I've already checked in quite early and yeah. And also worry about how will the workshops be carried out because usually the workshops require a lot of physical interaction and a lot of discussions, so I understand that actually a lot of students will be very shy to switch on their webcams or microphones to interact. So I already have the feeling that the workshop is going to fail or something, but then later as the course progresses, I feel that actually we are doing a very good job by studying online. And it's not as bad as I expected but I will always choose physical classes. Yeah.

Student C:

I think my concern was the same. Some of them are, [inaudible 00:23:18], about the labs about the workshops and about [inaudible 00:23:24] too. Like how is it going to carry out? But then as time progresses, like they say, after doing a lot of workshops, you find that somehow is to able to interact. It's just that it's not as easy as physical classes, but it still can be carried out. And then the extra sessions, like medical devices for the labs and then [inaudible 00:23:48] being carried out. So yeah, I still think is not as bad as expected.

Student D:

For me, it's firstly, the costs because there a lot of online classes that you can just pay online, then you can just get whatever knowledge and information you want. So I feel that it's very unfair to pay the same price but then it's also due to school's decision. You need a state decision. It's not [inaudible 00:24:32]. And the second thing is the lab sessions and Oskie. So like, yeah. And they [inaudible 00:24:39] ended up sessions in semester one, like for example, the formulations and [inaudible 00:24:46] . So it may be a disadvantage for us in semester two. And then we go for placement in the hospital.

Student D:

So because we are not very familiarized with the devices in the labs and the Oskie session is for me, it's quite difficult to do it on Zoom because face-to-face Oskie, I think is the best way. And the last one is the workshops, because not all of us are very familiar with everyone and sometimes we get shy when we face pictures, so it's a bit awkward in semester one, like not everyone is responding to the questions and that is the problem. And it kind of affects our teamwork and communication, it affects our product in the [inaudible 00:25:50].

Lecturer A:

Okay. Thank you for that.

Student C:

I like to add on.

Lecturer A:

Yeah.

Student C:

With regards to Oskie, what happened for me was that because they actually asked us to check if our house, are there any renovation or anything before the Oskie session, if you are worried about that. But the problem is I checked already, there really wasn't anything, but then during my session, like five minutes before my session, [inaudible 00:26:18] came to the road there and started wanting to do everything. And then luckily, somehow, a few minutes before my turn, it rain, so they stopped and then I could come into the Oskie or else it was [inaudible 00:26:37], really sudden thing. So I think that was like once an issue with online Oskie, because sometimes it's really hard to, even if you prepare for those [inaudible 00:00:26:51], sometimes really some emergencies will just come out and you're not sure what to do then because, for what they say, if you have any problems, you need to get an evidence.

PART 1 OF 4 ENDS [00:27:04]

Speaker 4:

Any problems, you need to get an evidence. It depends on your evidence and then see what happens, but it can be very nerve wracking because at the time you not sure whether what evidence, it, what had happened to you and others.

Speaker 5:

Yeah, also for OSCE, so, we do have a very strict time that we have to adhere to, and that didn't, sometimes that didn't really accounts for the lagging time of internet and also possible distractions. So it would be good if we have a buffer time, just a really short one, 30 seconds instead of a usual, really strict time. So that we can adjust for it, if it's zoom online, if it's OSCE online, yeah.

Student D:

I would like to add also that they require us to stay in sight with the camera. So our eyes focused on the camera so that I'm not reading, reading a script by my eyes is just, I can't always keep looking at the camera, right? It's so small. Yeah, so, that's one of the problem. And the second thing is I was writing in, on the paper because the patient wants, is talking to me about their problems. And then, I don't know whether the lecturer was suspect me of cheating or not, because I'm not cheating. It's just that I'm writing down some information, because I just want to look at it, to make sure that I get it correct. Yeah, that's all.

Interviewer:

Okay, and I like how this conversation is going, because really my next question for this is quite a sensitive question and I want you to be as honest and frank as possible. How do you feel about assessments so far, in an online learning environment? So obviously this includes your OSCEs, your workshops assessments, even your written, supposedly your written final assessments. So, that's all about it. And together with that question, do you feel that the assessments is a good measurement of your knowledge and your skills, or is a good form of tests or measurement? So two parts again, how do you feel about that? So I think the three of y'all have really shared briefly how you felt about your online OSCEs, but I want to explore that further, also about your other assessments. And then also then the second part, do y'all think that it is a true measurement or are good measurement of your knowledge and skills and the way we test you?

Speaker 6:

I think I will start first, because for me, I have quite a major issue, which is, I don't have an specific or an isolated that space for me to do my exams and assessments because my house is quite small and then I don't have specifically built room for me to do exams and assessments or OSCEs. So usually I have to ask my family members to keep quiet or something while I do assessments or OSCEs. So I understand that not only me have this issue. So I, I believe that there are students who have this issue and also the wifi, it will suddenly cut off and then I will drop off the OSCE, or I will not be able to complete my assessments on time, but the time is still ticking. So, it makes me even more nervous and anxious every time I do assessments, because I will worry about, Oh, what, will happen to the wifi or when someone suddenly speak and affect my OSCE or something like that.

Student A:

And then, I believe that it is not a really good way to measure our studies because we can always refer to discovery and stuff, but another way to see, look at it, if it's a good way to see if we can refer to a reference very fast and find out the answers that we want. So it's more applicable to like daily life, how we refer to references or sources to get answers, instead of like our real knowledge, because in some situations, for example, emergency situation or Acute care situations, we have to immediately have the knowledge in our brain. We can't refer to it or do a one man research for that topic, right?. So it depends on how you look at it. So I think there's good and bad for this kind of assessment, but Monash has already turned to eAssessments since last term. So I think I'm already quite used to eAssessments, but of course I will always prefer written assessments because written assessment is, because it's faster for me to write instead of typing.

Student A:

So, I prefer written assessment and then of course I also prefer face to face OSCEs because it is more real and more realistic. And then the zoom OSCE is less intimidating. And then, yeah, but it's also good for us because less intimidating means that more relaxed and better performance and higher of the marks. That's all.

Interviewer:

Thank you.

Speaker 4:

I agree with Student A, because in terms of marks, it's a good thing because it carries[inaudible 00:05:40]. Yeah, it carries [inaudible 00:32:43] but then in terms those disturbance then is a disadvantage because, for me, although I stay, I have my own place, my own room, but then because my parents are working so in the morning brother, because he studies in the afternoon, he'll be at

home in the morning. So when I doing assessment, once I remember towards the end of assessment, he somehow broke the pipe and the pipe was splashing water and he just ran into my room and told me, but I'm doing assessment that yeah, the pipe broke and stuff like that.

Speaker 4:

So, so I think the good thing is we can refer. At least with the disturbance around us, which you can't control and the wifi issues. In terms of whether it's a good thing, to measure our performance. I will say that, I will agree with Student A, that, it's not so good to measure our knowledge and what we know, but then maybe it's a good thing to measure whether we know where to find our resources, if we need them. Yeah, that's what I will say.

Speaker 5:

I feel both [inaudible 00:33:57] and Student A has covered most of my points. Regarding eAssessments, I think we are quite used to the platform by now. So, it shouldn't be a problem. Yeah, I think they covered most of the points for mine, yeah.

Student D:

For me, the quiz, it's good that you can refer, but then actually there's a time limit, for example, if you're in campus, the questioning are for one hour, but it's now about 20 to 40 minutes. So it's [inaudible 00:00:34:41], but you can still refer if you're not really sure about the answers, but at the end of the day you also have to study. So it's a good in a way that it helps you [inaudible 00:34:55] understand what you are missing out after you study Discovery, you think that you understand what you study but actually there's something you are missing out. So,you know, what you need to fill in and so [inaudible 00:35:11] already mention. Yeah, And then distraction as well at home. So there'll be lots of patients that, and I prefer discussing with my friends about certain topics before that assessment so that I would have the clearer understanding, but that's also my weakness because I don't really know how to study independently, but because I prefer more groups study.

Interviewer:

Okay. So yes, we introduced eAssessments last year. But the difference between last year, and this year was that this year now it's because of this environment, we have now an open book exam, in the other sessions that we've had so far, some of your friends express, a strong level of unfairness of you. That is a very, it is just very unfair to have an open book exam. Do y'all feel the same way as well or not?. The general feel so far I sense is that, no, you're okay with an open book exam, but I'm not sure. So what do you think about that?

Student A:

Why is it unfair? Because I thought everyone gets to open their book. It's not like only a certain group of students are able to do the test in an open book manner. I thought it's the same for everyone.

Interviewer:

Yeah, but the unfairness came that because not everybody knows how to use these references. Well, number one, and then number two is not actually a measurement of, as you said, it's not a measurement. It can be not a measurement of your personal skills and your knowledge, but really it's a measurement of how good you are at looking out for answers. Again, there's no right or wrong, but I just wanted to know what this group feels about it, but really that, those are the groups that shared those, were some of the sentiments that they felt [crosstalk 00:37:17]

Speaker 4:

We actually, we don't really have that much time to refer. I think the only thing we can refer is that, maybe in addition to EMH, will be ETG. So because in the past year we only had EMH , so yeah, I'd say you still need to know, because I know for me, I don't really have read but, [inaudible 00:37:45] except for the addition in ETG part, for some specific questions where maybe I'm a bit doubting my myself, let me just quickly go to ETG and roughly look through it, I'll get to confirm is correct, right, okay. Then I just come back. Yeah. But I'd say that's really not much time to do that.

Student A:

Yeah, I agree. But I think the only unfairness that I can really think of is just the environmental issue. Whether the, they don't have a space or they don't have a very good wifi connection, that's it? Because , what is inside, we don't really have time to ref refer, but even if we have time to refer, like pharmacists also have to be competent to be able to find resources effectively. So I think it should be included inside as part of the assessment to see if we are able to look for an answer very quickly via different resources. So I think it's not unfair.

Student D:

I think unfair in terms of if they're really studied, but then the question came up, it's a different type of question that they can't even find it in discovery, have to search it online. So it depends on how fast they can search the information and what resources you use. [inaudible 00:39:14]

Speaker 5:

Oh, I remember for you to some of my seniors actually use APF as a reference. They see that they can actually get the book and actually bring that book into the exam hall. This is a disadvantage for us because we don't really have APF with us because it requires access and I think a subscription to pay. So instead of referring to APF, what we opt for is online and [inaudible 00:12:39] , which I feel covers up the unfairness in this sense. However, I'm not sure if the exam questions are totally the same for past years, previous years and our year. But if that is the case, it might be unfair for our seniors as in, they might not be able to check out on ETG. I think, you know, the flow, but in terms of drop reference, I think it should be the same.

Interviewer:

Okay. Thank you for that. And greatly, this is all just our last question for the first part of our focus group. And really, I think some of you don't have covered disagree, but I just wanted to see whether there's anything that's missed out. You would just really wanted to know. Was there any thing, roads changes, challenges, sorry, during the transition. So some of you has mentioned about your differences in internet connection and all that, but any, did anybody as many details to add to this?

Speaker 4:

Because in our physical class or during eAssessments, actually spare laptops laying around. Yeah. Just in case you have problem with a laptop because like for my house, I think the only, that when my parents are working out, When my parents are working, the only really working laptop in my house is my laptop. So yeah, so the problem would be that. So if anything happens to it, I will need to get an iPad and just type using the iPad everyday, so I think that's what I mean.

Interviewer:

So did you actually faced it during the, over the course in the past three months?

Speaker 4:

uhm [crosstalk 00:41:23]

Interviewer:

So this is not just limited to assessment. It could just be also be part of your online going for my ILS or online workshops

Speaker 4:

Right now. Not yet. Hopefully. No.

Interviewer:

Yeah. And

Speaker 7:

Another issue that I observed during interactive lectures and workshops, and CTLs, is students will find that their webcams are broken or their mikes are not functioning, and I'm not sure if it's real or if it's just an excuse, to not open, switch on their webcams or mic. I feel that this is a very big impediment for lecturers to carry out the, to carry out, continue the lectures and classes. Yeah, so, this is, this may be a technical issue for our course. Yeah.

Student D:

For me, it's during PP we are having a article, clinical article assessment, so it's a bit hard to discuss online it's, because it's a clinical article, you need to know the outline and then if you don't really understand, you have to ask the lecturer whether it's current or not, whether this article can be, can be acceptable or not. So it's, I feel it's a bit hard to ask online because the lecturer will have so many articles because so many students have so many articles to read through, so they're not going to read everything, they're just going to browse through. But then we are not really sure whether it's the correct one or not. Then some of the questions in the requirement is it's not a, we don't bring up whether it's ethical or ethics clinical article outline or not. So it's a bit confusing at times.

Speaker 8:

Yeah. So just to add onto [inaudible 00:43:24] point, using point on laptop does not because my laptop, it actually, it's actually not working for, I think right before semester two. And I was really worried because I only have one that taught and the rest of my laptop, isn't working at my house. So I could only opt for the desktop, which sometimes also go haywire. So yeah, I'm really worried about that. Second point is I feel like the attendance is a bit lower sometimes online because they say they actually can refer to recordings. Most of my friends actually attended, but I do know that attendance is quite low. So that means that you didn't get the full benefit of lectures before going to bookshop. And this might actually makes answering the workshop questions a bit tougher as a whole group. Some of them did not really properly understand. And the last part I would like to point out is that because we are doing it online, some questions that we are trying to ask didn't actually get across. So either the way we phrase it, isn't clear or the catcher didn't get what we're trying to say and has, they didn't answer the questions directly on, on point. So you're still lost on what to do. And there was a slight confusion in rubric for CC the other day, but it was terrified I think this morning. Yeah, I think, but I think there was a point that we can point out because just in case there's any future assessment that requires the same scenario. Yeah.

Interviewer:

Okay. Thank you. [crosstalk 00:44:57]

Lecturer B:

It's hard to put questions across, is it due to the strength of your connection or it's a mic issue. What do you think is the barrier here?

Student A:

It was actually the rubric where we have two person ones the patient and one is the pharmacist. So both of us did the role play. So I am the patient and I'm also the pharmacist. So the confusion was in which point of view, should I look at this rubric? Should I look at it in patient point of view or pharmacist point of view?. And then the second question was who should fill out the rubric? Me as the pharmacist or me or my friend who is a pharmacist. So there was a confusion there and it is really hard to say it out like this, or even share screen, unless we have a example of completely filled form. And who wrote here? Who wrote this? Yeah, that was the confusion. It's not about the line.

Lecturer B:

Okay.

Speaker 4:

Because for that one physical classes, we just fill it up as the six falls and then the hand up that is okay, but because it's a night, each of us into summit one individually. So they're not sure who shows that me a, which from,

Speaker 6:

It's a bit hard to ask questions. If you have a lot of questions,then you're going to write a lot in the email or in the forum.And sometimes you don't know how to phrase the question. Yeah.

Interviewer:

Okay. Thank you for that, Lecturer B, do you have anything else to ask or to add to this?

Interviewer:

[crosstalk 00:46:36] Okay. All right, thanks. All right. We're doing good timing. And I, we're going to proceed now to the next section of our focus group. And so this next section is really focusing on the design of the syllabus that you will have the reality, that the fact that how your are learning here at Monash powers, we give you discovery materials, we come to your ILS and then you have your workshops. And then after you close the group sessions, these are unique to, to Monash, particularly to Monash policy program. So the next few questions that we're going to ask you really in reference to that aspect, this the style or the design of the course. All right. So first off the bat, I just wanted to ask yourself this, what do you think about the, these, the discovery materials, the, this whole, and this is a very big area, this whole idea that we give you discovery materials to go through before coming in for your ILS. I want yáll to also elaborate of what do you like, what do you not like about it and why as well? So, Yeah.

Student A:

I like that we get to study beforehand before the lecture. So we can ask during the lectures, what we don't understand for the discovery, but I don't like that discovery is sometimes incomplete and sometimes inaccurate and very confusing because sometimes it will be different from what the lecturers are teaching, but then it will eventually not be clarified at the end. So for example, we are confused with the, something in discovery, and then we ask the question, but it will still not be really clarified. Yeah. So, but then we will still carry the confusion until the end of the unit. And then we will never look at it again. So, but for me, I think it's better to make it more clear. And then some of the

mistakes from our year, for example, my year 2019 year one is not correct until now for the new year ones. So I think it should be updated immediately after someone raises a question. Yeah, that's it?

Speaker 7:

Yeah. I think, for our year an example will be the bullet be the fiber part, for the indigestion IBS [inaudible 00:22:19] because according to the discovery, it was, it seems that fiber is a not good thing because from what they said, they keep say from the other fridges, it seems that fiber is not good. But then even though the tub criticize us and everything, so there was a suggestion last year, but it wasn't change for this year.

Speaker 4:

Yeah. I agree that discovery allows us to study beforehand so that we can have a better understanding and questions. What I feel that discovery could be improved on is basically we have a lot of links in certain in discovery. Sometimes it's useful as we can before and gain a better understanding. But most of the times it's confusing. Whether do they want us to memorize that 80 pages? Or are we just reading through it for a more understanding? I think it will be good if they actually stayed better. It's an optional read or a must read so that we could actually know to what extent we, because I know that healthcare there's like, no, then it, so it will be good if we can identify, not identify, we can define a barrier to, to which form it will be tested on two. And to which point we could continue to expand our interests on.

Speaker 4:

And I do also agree with Student A and eating that some discovery materials are quite misleading and some lecturers actually sit, they will update the discovery. But what I usually do is I download it the discovery beforehand. So it's in the form of PDF and I sometimes will not rectify the message unless I is, unless it is really confirm. So that is a confusion for me too. Thank you.

Student D:

For me is I like the format on how the classes are organized. Like for example, you have to Mondays is our session, then I L and STK, it's a very good schedule, but for discovery is for instance. So is that after hypotension by blah, blah, blah, then after they sentenced and they have this type thing that I always have to it in my notes, because it's quite disturbing to read that. I know it's like the reference to go, because it helps us to find what resources is used to for this information.

Student D:

But then when you want to really study on it, how to say it's pretty distracting. And for the, for example, like I feel a bit messy that I see the Malaysia when I reach, it's very organized. And when I read like, kind of talk to them, but I really can't understand systematically how I should like understand this topic. So after that I looked for gender, I have to idea what this is like this update. But when I read the discovery, like I have to like organize and make it. Oh, okay. It's supposed to be, say this eight. And then this be like, this it's a bit, yeah, not very systematically.

Speaker 5:

Oh yeah. I have a feedback from some of my friends that we actually discussed on because in discovery they often insert a video and that is actually our notes. So they did give us the PDF slides, but the way they, they discussed, it's more comprehensive and more detailed. And because it might be because of personal reasons or my friends, but we sometimes cannot get the accent or maybe they talk too fast and we miss the point. So we have to return to it again and again, but we still couldn't get it. And a possible reason, a possible mistake is that we get the wrong information. That's

the, in the worst case scenario. So if it is possible, it would be good if we have both the notes and also the transcript of what the person is actually talking about the video, just in case we miss out any important points.

PART 2 OF 4 ENDS [00:54:04]

Interviewer:

Okay, if I can just summarize some of the sentiments and I won't comment on the specific units or the specific modules, but from a general sense, more of you all do like the idea of having access to some knowledge or some material to study before your lectures workshops, but obviously, then you would prefer a more clearer presentation of this information. Is that correct?

Interviewer:

Yep. Okay. I see most people nodding heads. Okay, cool. Thank you. Next, I'm going to ask now it's about your in-class activity. What I mean by that is your daily sessions that you have in your interactive lectures and your cluster groups and your workshops.

Interviewer:

I think the reality is that these sessions are designed to have a lot of interaction in them between academy and student, and between student and student. I guess it was obviously emphasized more your workshops, as well. What do you all think about that? Is there anything that you like or you not like about it and why, as well?

Students:

Oh, I like how workshop has our understanding and how to apply it into real life scenarios. But what I feel could be improved is that, every time after we finish a workshop, there's usually a wrap-up. So, because we are in different workshop groups, the wrap-up session by different lecturers may be in different style and contain different information. So if we have a streamline wrap-up for workshop to ensure that everyone got the same information and explanation, it will be really, really beneficial.

Speaker 9:

Sorry, do you mean this in terms of different lecturers giving different summaries or you mean different subjects having a different manner of giving the summary?

Students:

All right. I'll just give an example. So for CC-3 what we actually do is we have a workshop, but we don't give a wrap-up on that day itself. So, the second day in CTL the lecturer will actually give a general wrap-up for the workshop before, and then they will open the floor for discussion so that we could ask question.

Students:

It is good to have a wrap-up right after the workshop, a small wrap-up for the important points. But to elaborate more, I think it will be good to do it in the session where everyone is in CTL so that we get the same information and questions. Because some people will ask questions about the workshop and it's beneficial for everyone to actually get the same access to the information. Does that answer your queries?

Speaker 9:

Yeah, more or less, that's more or less.

Students:

I think to add on to your answer about this, is because different sessions have different workshops. So, for example, if a session at 10:00 AM then I have to do scenario one, and then another session around 2:00 PM, where I will have to do workshop two, but we don't really have access for the second session to workshop answer. And the scenario, sometimes I press into Moodle... I can't even press into the link because it's for the second session and it's a different scenario.

Students:

So I would prefer, if it's possible that the lecturer would provide a scenario and the answer for the second session. It's not all the others that have the workshop answer, some scenarios are not provided. So it's a big [inaudible 00:04:02].

Students:

Yeah, I do agree because, for example in last SEM, we only had three different scenarios, so my group was carpal tunnel, and others had different scenarios. So during the finals, when I revised with my friend, we were trying to see if we have covered everything that we should know. Then I just realized that my friend, she said she have no idea what's carpal tunnel because they had no access to that scenario.

Student A:

Actually what we did for last year or year one is we have one representative from each session to upload a copy of our answers and questions into our own private Google drive, which is for the whole year one. But then it's not quite efficient because not everyone remembers to do it and not everyone is willing to do this because it takes a bit of time. So it's better if it's open straight on Moodle and we can just access it because we will share it anywhere. So yeah.

Students:

Yeah. In that spot, can I just add onto Student A's point? So it is in the Google drive and everyone has different answers. So you don't know which one is actually correct. So it would be good if the lectures could actually give us a guide on like... Okay so for this scenario, what are the treatment options that you could consider and what is considered the best and the reason behind, because everyone has different reasonings and it make us more confused when we look at others' answers and we are trying to figure it out ourselves. Yeah.

Students:

I think the Lecturers can use the Moodle platform to give the students the link, where we can place our workshops inside for different sessions. And the lecturer can choose three different workshops and give specific ideas of how we should improve our answers. So the lecturers, they shouldn't be the ones to give out the workshop answers for students. They can give us a feedback on how we should improve our answers.

Interviewer:

Okay. Thank you for that. Can I just highlight now focus also on your lectures, we call them interactive just because there is obviously a strong interaction component to it, contrast this with your prior experiences so far where the lectures have been given or delivered to you in a more one sided direction way. There's not some, the only interaction when he says from the Academy or from the teacher, the students. Whereas I think you all can visualize now, then you're interacting back to your ILs is like a two way interaction. Now that happens in the lecture environment, what do you all feel about it, do you all like it or do you all not like it and why?

Students:

I think it gives us a chance to try to answer, but then I think the only problem is, actually sometimes because the question is very hard, so everyone actually is done about what they're thinking about it. And I think because we can... Especially for now when it's not face to face, I think that we the one to answer the lecturer, but sometimes it's because I don't think anyone knows the answer. Then I'm still thinking or Googling or trying to find somebody to get the answer, but then somehow nobody answers. So yeah, I think that is one of the issue there.

Interviewer:

Okay, but you gave a very generic opinion about it. I want to know what do you like

Students:

At first, I wasn't quite used to this interactive, but after one year passed I think for now I'm starting to get used to it. So I think what I like about it is that it actually makes us ready, pass us, makes us to contribute and makes us to make sure we understand the thing. Instead of just listening, listening and listening, I really don't understand and I really don't care. So yeah, because now we have to answer, so we actually do make sure we know before we answer. And if you can't answer, you can say that, Oh yeah, we don't really know this topic well, so we need to revise more on it so yeah, I do like it more this way.

Students:

Yes. I also agree with ILs because most of my previous experiences study is more of a balmy type. And I think IL give us a place where we know where to focus our studies on what are the important points and for the life ILs, I feel that it prompts us to think more because the questions are quite challenging, while online, it gives us the opportunity to do more research on the topic that we don't understand about. Personally. I do like interactive lecture as compared to one vs one.

Students:

Yeah. I also prefer interactive lectures, but for now the online interactive lecture is not so interactive anymore because compared to physical classes where usually the students will fight to answer questions, or sometimes the lecturers will like give for you rewards something. But it's not more for the reward, it's the interaction for me. I like the interaction between the students and the lecturers, it's very interesting and very engaging. But compared to the online, we can't do anything, but yeah. And then the lecturers can't really see our faces as well, so they can really gauge, how is our understanding and how do we feel about the question? So they are not sure how to proceed. It's very awkward, when they ask the question and then there's a silence, and then the students can't answer, the lecturers can't say anything. So it's not so interactive anymore. Yeah.

Student D:

Personally I... There was a time when [Dr. Shwan 00:10:33] posted in the Zoom, I don't know how he does it, it's like a poor Arabic question. Then suddenly in the Zoom, there's a pop up box in there, and then the question would be there and then you'd have to press A-B-C-D. [crosstalk 01:04:52] okay. I really liked that format not because it's really... It's quite intriguing. I liked the format it's because the students are required press their answers. So it's very convenient. You just need to press for everything you think is correct. And then it encourages participation to answer questions.

Students:

Yeah. I actually agree with [Rossana 01:05:21] is actually a poll function on Zoom, where you can actually choose. It would be good if the questions can be provided afterwards, so we can think about

the question and still refer to it later. Because now what we are using is we are using Poll Ev. We are using the zoom chat function, or sometimes you are using WhatsApp to communicate, to send messages across. So I think it would be good if we just streamlined it to use zoom or Meet or something, so that just by fixing one, we won't get distracted because sometimes I look at my Poll Ev and I realize I missed out something when the lecturer was talking. Yeah. So it's just distraction. I agree with Rosanna.

Interviewer:

Okay. So again, I'm taking a step back to, again having a more broader and more generic view. Can I summarize that the style or the design of the Monash pharmacy program, it seems that you all have a more positive feeling towards it compared to your previous learning experiences in the way they were designed. Is that, can I summarize that for the four of you?

Students:

Yeah.

Interviewer:

Okay. All right, thank you. And again, it's fine to disagree. We just really wanted to know how you feel about that, but thank you. This sub question is really then to ask whether where you're now is to compare this with your... Or to reflect now a bit more about, when in your first year, when you started. Obviously when you first started, it was everything new and what's this? And all that, how am I coping? How my struggling, what were your initial feelings about it? And again referring to this design of the program. And how long did it take for you to change or to adapt?

Students:

I'll go first. So for me, because I joined a lot of activities in my first semester and I find it really hard to balance between my academics and also my curricular activities. I feel really stressed out when I first started, but it took me around one semester to completely adjust to it. And because I have this confusion very early on and I didn't clarify it, so it builds up and I kind of freak out during the first semester. But overall, I would say that I prefer the structure year two, which is you go for discovery, IL, workshop, CTL, and it is actually connected. So there's no breaks in between, unlike year one. So year one what we had was, I think we have discovery, IL and then break and then workshop, CTL. So in between that, I kind of like, I couldn't get the connection. So it took me a while to adjust to that. Yeah.

Student A:

For me, because I took [PU 01:08:26] foundations, So the foundation system is similar to university degree system, like the timetable arrangement, and how the course is delivered, like tutorials and stuff. It's actually similar to how a degree works. So I'm used to the system, but then Monash is using a very efficient system where students have to study on their own, and then it depends on the student's arrangement and stuff. And also the lectures are very interactive and the workshops are very based on real life scenarios. So I think it is very beneficial for us if we want to apply it to our future jobs and scenarios. Yeah.

Interviewer:

And the other two of you, anything else different or anything else to add?

Students:

Yeah. [crosstalk 01:09:28]

Interviewer:

Okay. That's good. Okay.

Student D:

The thing is just that during this year's semester, I think, all had a challenge of that not being online because of different system to use, but then after, during this semester [inaudible 01:09:58]

Interviewer:

Okay, all right. So you adapted. Okay, all right thank you. So now proceeding onto my next question, this, this method of learning, where we give you discovery materials that we use for our interactive lectures and then workshops and all that, do you think is useful, or it helps you in your learning for all the units that you've passed so far?

Interviewer:

To help give an example, so I think Student A talked a little bit about her CC units or at least the experiences in the CC units so far. And from what she said, I sense that she thinks she, it does help her, this style of learning. But I was just wondering, do the rest of you all feel the same as well? And again, I want to ask you to compile all the different units that we've had so far in the past two years ago, undergraduate program. If you don't understand what I'm asking, just let me know. If you don't understand my question, because I know it's a bit, wordy.

Students:

I don't really understand.

Interviewer:

Okay. All right-

Speaker 9:

Should I try and rephrase it?

Interviewer:

Yeah.

Speaker 9:

Well, perhaps the question is something like, so once you're doing a lot of basic science things, like how body works, how medicines work, and then after that subsequently now you have some units that are more practiced based on your comprehensive care. PP is also somewhat practice based, so do you feel that this style of discovery, IL, workshop, CTL, is it useful for all kinds of subjects? Or do you think it's really just not useful for CC units, but it's not really useful for how body works? Yeah, that's kind of the general idea of this question, suitability of this system for different types of subjects.

Students:

I think they are applicable for all, for all units, because since all the units we're taking are all related to our future careers, unless we have English essay writing then yeah, of course we don't have to relate it to our course. But then I think all the units we're taking will eventually be used in a future career, so it should be more integrated with our future scenario. In my opinion.

Students:

I think that it's applicable for all the units, because discovery actually gives a pre-knowledge of what to expect, and the basics before we enter IL where we get further clarification, and then you can ask questions to clarify. Then for workshop, we get to applicate what he know, so I think this structure is quite useful for all the units to get a hang of the stuff inside.

Students:

I agree both of them.

Student D:

I agree. But then the workshop for PP, I'm not really sure whether it's applicable or not, for example, like the systematic review, the case control corporate study on dose, we have to read an article and then we have to apply inside our workshop worksheet. I'm not really sure whether they are reading through the [inaudible 00:19:52] in a correct way or not, and how should really apply it, because I'm not really sure how it's applicable to the worksheet and the [inaudible 01:14:07] .

Students:

I think it's really applicable for some situation where maybe you get a patient who has a very rare case and then you have to research for the case. And then that is when you have to use... So maybe like a hospital pharmacies instead of community, I guess. But community maybe like new product or new herbs or medicines being marketed outside, and then you have to research, how is it beneficial for a specific patient and help the patient with the research? I'm not sure, but that's how I think-

Student D:

I think it's the extraction of the answer for the article. Sometimes we don't really know where to extract it from. Even though after the lecturer tell us, this is what we're extracting, and this is where you can get the answer from. Sometimes it's like you are doing your own research, then you if sit by your own article then you are all by your own, there's no lecturer, there is no one to guide you. Yet you are only one who is doing the research, so I feel like that's a better way to help us talk to really extract information.

Students:

I think I understand what Rossana means, because in that workshop, my team actually confused primary research articles and secondary research articles. They actually asked us to prepare beforehand and we didn't know until we entered the workshop and we don't have any materials to work on. So yeah it is a good method to, but I think there was some confusion in between the communication on what the lecturer wants and what we actually did. Yeah.

Student D:

Yeah.

Interviewer:

Okay. Yeah, thank you. Now, given a choice, would you still like to learn or go through undergraduate program to this style of learning where we give you a discovery and we'll try to brighten your workshop and your cluster group.

Students:

Yeah.

Interviewer:

Yeah. All right. So that's all positive or give from the four you, okay thank you. So going on now to our last few questions, we're almost to the end of it.

Interviewer:

Now we didn't prepare for COVID. We didn't prepare for all these things would happen when we have to do physical distancing and all that now. And who knows what 2021 will bring, nobody knows the future, but let's say if this style of learning was to continued again for next year. What, what do you think can be improved or what do you feel that as a student do you think that will be the best suited design for you to help support you?

Interviewer:

And I'm leaving this question very open ended just to let you all be creative of how you imagine it. So we're asking you now, how do you like to learn knowing the current setup that we have? Let's say there's no vaccine, we still have to do physical distancing, governments still do not let us do face to face classes and all that. What do you all propose?

Interviewer:

You can take some time to think about it because it is a big question and I just kind of just dropped it on you. While you're thinking about it, I could maybe just give some examples of how you'd like that we can describe it. So say for example, you are happy with your interactive lectures online for now, so that could continue. But then some of you all have mentioned about the workshops, online workshops may actually not be really... Or maybe there are some things about the online workshop that you don't really like.

Interviewer:

So maybe what do you think can be improved by their own debt to do, to help you like it more? number one. Another example is that, some of you shared that, Zoom ask... So this is not coming to your assessment parts. You totally don't like how the Zoom asking is being run, but then knowing the current set up where the governments has not allowed us to have all the space available to banks that if Zoom asking has to continue, what do you think could be better? Some of you suggested, I think one did say that, or give us a bit more time or [inaudible 01:18:59] between each student or between each cases, so that in case something happens. So that could be one practical way that we could incorporate that. But was there anything else that you think we could help improve or change to help make it a more positive experience for you? Yeah, the idea is to really come up with an improved version of how the year would look like for you.

Student D:

I think that this ejection, because I believe if we have a question we will, if we're in campus, we will just go to the lecturer's office and just ask directly on any problems we have. But now, because there's only an option of email the teacher of our questions, and sometimes we don't know how to type all the questions that we have because it's quite limited, the lecturers replying us. Maybe we can request zoom a session, one to one with the lecturer Zoom session so that we can directly ask them our questions on certain issues. But not during class, because class is a little bit too general because the lecturer will be teaching on the specific topics. But if they have other questions or other topics, maybe there's a chance for us to directly Zoom the lecturers and ask them directly on what problems we are having with other topics. I'm not really sure whether it's possible yeah.

Students:

To build on Rossana's suggestion, I think if, because I heard that some, you need the actually.

PART 3 OF 4 ENDS [01:21:04]

Students:

I think if, because I heard that some uni's, they actually have something called office hours. So the lecturer allocate one time that, sorry, give me a second.

Interviewer:

Okay. Anybody else want to comment while we wait for... [Inaudible 01:21:22] coming back. All right.

Students:

If we have an office hour where we have the zoom question session, [inaudible 01:21:26], it would be easier for the lecturer and also the students. So it's a fixed timing at a certain time. So anyone has questions can just go into the zoom and ask questions.

Lecturer A :

Yeah. I think the time is quite okay, I suggest the students do not misuse the lecturers time.

Interviewer:

Okay. Before I asked the other two as well, but just to comment on this point first. So I get what you're saying, but the idea of the risk behind that is that I hope I give you a bit more attention to the two of you, and then I unconsciously forget to comment or to help support your 90 other friends. And then sometimes there's a danger of miscommunication. I'm not saying that that's happening now already, but that there's the risk of that happening. And so that's why we have this anonymous forum that we have popped under some of your units where you are allowed to write your questions where we can respond to you written on the forum, and that way all [inaudible 01:22:37] students have access to that. What do you all think about that? But unfortunately for some people they don't really utilize it. Some people like it. I'm not sure why, so what do you all think about it?

Students:

Well, personally, for me, anonymous forum is a bit messy to refer to for studying because it's not categorized in any sense. It's not categorized by a topic, for example. if it's like, copy one, two, three, and if you have specific questions for that topic, you put it in. So if I'm confused about that topic, I could look it up. It's a bit different in that sense, because it's an individual email, or I can compile them. So it's a mixture of CC and PP. And sometimes I don't really refer to it because it's just too messy. But if it's streamlined, I think I would actually look for anonymous forum. I would prefer anonymous forum more than the session that I mentioned earlier.

Interviewer:

Okay. Thank you. Student A or Lucy, if you have anything else to add or you're not sure what my question was, just tell me as well.

Lecturer A0:

I don't really have any suggestion to improve because we can't really do a lot, because the most we can do is zoom session. And it's not just like when, [inaudible 01:24:02] suggested. I think it's the best suggestion that we can provide, because that's what we can do most. But then for labs, I do have a suggestion, but we are going that for labs anyway. But, but then I saw in America, they send the lab kits and the pot, sorry, the whole pig to be dissected to their houses. So maybe we can do something

like this, but I'm not sure that because it's not within the budget, but then, maybe we can send some of the materials to the students' houses and then let them do. And then we can have a session where we practice the practical. But then we are going back anyway. So it's not really valid for the other non-lab session. I think there is not much we can suggest.

Lecturer A1:

Well, it's a bit off topic. Can I ask something about placements?

Interviewer:

Yeah.

Lecturer A1:

Because when I go to placement, my preceptor actually asked me, do you have a log book available? So what are you trying to learn when you're being here with me, studying about community pharmacies? And I find out that I actually have nothing to offer to her, except for the rubric that we got. I'm not sure if there's a guide or perhaps I could ask my friends, but I don't really see a proper guide, like a log book for me to fill in and to know what information I need to know for year two itself. I'm not sure about future placements yet. Does anyone has access to some log book that I didn't know of?

Lecturer A0:

Isn't there a guide like [crosstalk 01:25:36]. A stack of paper together with our, [crosstalk 01:25:40] that one.

Lecturer A1:

We learned about smoking cessation, diabetes care.

Students:

I think there's a page where the preceptor has to circle for us and that's what we have to learn, right?

Lecturer A0:

[crosstalk 01:25:51] [inaudible 01:25:54] the log book is from the [inaudible 00:04:56]. You can search it online. [crosstalk 01:26:01] There's a document. There's a PDF you can download.

Lecturer A1:

That's all right. [inaudible 01:26:09] for those that she don't have. So I'm not really sure how we should apply this in year two.

Lecturer A0:

Like Pfizer? I don't think that was necessary during our [inaudible 00:05:19].

Lecturer A1:

Oh, okay. It's fine then.

Interviewer:

Yeah. So just, just to comment. As you progress through the program, your future placements, particularly in your years three and years four, we will have more specific log books ready, because the activities will be more targeted. Your year one and your year two placements are a bit more if

you're [inaudible 01:26:46] and I see it more introductory and exploratory where we want you to just be exposed to all these different setups, but the preceptor sites should have already been breached of these. So maybe it was just a case of omission or that they were just not really clear, but we were expecting the preceptors. So, I think to be honest, if there was any particular assessments in your respective PB PD units, the coordinator would really have emphasized that.

Interviewer:

But coming back to our discussion really about things that we can improve or not, or want to be optimized.

Interviewer:

So I think the sense that I get from you guys is that you just want you just want your lecturer to be more available or more involved in answering your questions or in helping to support you, how that could look like. Whether, if it's a zoom, or a particular time where the lecturer just sits here and just take whatever questions you get, or a more clear presentation of your anonymous forum, that's up to well, whatever now. But those are some of the examples of what that could look like. Okay. So, that's just really summarizing this point. Having said all that now, do you think this style of learning-where we give you a discovery material and get you to study this [inaudible 01:28:30] for coming into our classes-do you think this has helped you to be more adaptable or do you think this whole style has helped develop some skills in you that you are not aware or you are not conscious of, for example? Has it helped you to grow as a person?

Lecturer A2:

To take responsibility for our studying. It makes us start to realize so much in lecture and at least we have basic knowledge and study first before we go to the lecture. So we aren't going in very blur, at least we have basic knowledge already.

Students:

Yeah. My time management has improved since the online classes start, because I have to force myself or else I will be behind the CTL and the workshop. And especially the workshop where you have to study the interactive lectures and discoveries first. And then I also learned to speak up more during lectures, because it's difficult to carry out and online zoom or workshops. So we always have to initiate the conversation first or speak up or answer the questions.

Lecturer A1:

I think they covered all of my points that I have.

Lecturer A0:

I feel like there's a time that is given for us to read our discovery. Makes me more detail and more focused, because I know that today, Monday, is the discovery day and I should focus on discovery and not on other stuff. So it's like an enforcement day that you will have to learn yourself and not rely on that.

Interviewer:

Okay. All right. Thank you. My last question for our session, actually. And after this, I'll ask Lecturer B also if he's got any other things that he may want to ask, but really, we've talked a lot about a lot of things the past hour. So, but maybe just not, we go one round. What do you all think was the most important thing that we talked about today? And you tend to be different, or something that struck

you the most or something that you were surprised with. For example, what was the most important thing that we talked about? If there's nothing also, you can say nothing.

Lecturer A0:

The most important thing is interaction and communication, because since everything, now, is online, physical interaction is already removed from the whole communication part. You still have to speak up more and interact through online more.

Lecturer A1:

I think that today's important message is to highlight what students are facing and what causes that. What measures that we can take to improve the betterment of our studies and the differences between online study and physical study method. There're any barriers that we can fix and how we can prepare for our next year three.

Students:

I think the important part about this session is that we can see both in the point of view of lectures and students in terms of our barriers and how we can improve from them.

Lecturer A2:

I think they've said everything.

Interviewer:

Okay. Thank you. Lecturer B, do you have anything else that you'd like to ask?

Lecturer B:

One question that was actually a follow up from [inaudible 01:33:17], just now [inaudible 01:33:18] asked a question on if we continuing online next year, what would be improved? But let's say we go back to offline, let's say everything returns to normal next year instead. Is there anything you would want to retain from the online part? That means to kind of bring some elements of the online thing into the offline experience? Is there anything you would keep instead? Or combine?

Lecturer A0:

[inaudible 00:13:05]. They sent us the slides, they are very handy for us because we don't need to take pictures of the site during our [inaudible 01:34:16] and then we face our laptop, because it's clearer. [inaudible 00:13:22].

Lecturer A2:

So if we intend to do that next year for offline, maybe they can continue doing the extra sessions online on zoom because it will be easier for both lectures and student. [inaudible 01:34:43] And then go, if it's planned to do one.

Students:

And the lectures and the sessions are recorded, I think it's a good thing because on year one, a lot of lectures are not recorded and especially on echo three 60, not a lot of the lectures are recorded. So if we managed to record all the lectures or CPL or workshop like how are you doing now then I think it's better.

Lecturer A1:

Yeah. They covered all my points.

Interviewer:

Okay, great. Thank you. Anything else, Lecturer B?

Lecturer B:

Oh wait, let me just quickly, Oh, we asked them already.

Interviewer:

So in the meantime, while Lecturer B Is going through some things, is there anything else you would like to ask us, the four of you? And this is really specific to what we've just been talking about over the past two hours. I need thoughts, questions, comments, feedback.

Lecturer A0:

Is there a chance that we are going to have zoom sessions in year three, semester one? Or is it physical class?

Interviewer:

We don't know. We're not sure yet. And I know it's not a very satisfactory answer for you, but we don't know, because the SOP's are changing every week. I think you can be confident that we are in dialogue-the university at least-is in dialogue with the ministry of the higher education. Once somebody makes a decision and we clear that and we speak, then we go ahead and plan. But, in the meantime, we also don't know.

Students:

I understand that it is a policy of the school to not reveal answers for assessments after we finish. But I would just like to ask, because for example, if I get three questions wrong and I don't know which three questions I did wrong, I would just kind of continue repeating the same mistakes until I finished the end of the course. But even in year one, because I said, I wasn't really sure about my basics. So a lot of things, until today, I still don't know what I did wrong. And that is actually hindering my studies because I don't really know what my mistakes is. And even if we have our own, how we usually get our test papers back after exams, just to review and know what we did wrong, it will be good. But because we don't even have the access to the questions and also the options available, we can't even review what we did wrong. Even if lecturer is not giving feedback. I think that is quite a hard topic for me to study because I don't have any exercise to work on except for IL and workshops.

Interviewer:

Yeah. point taken on that one, thank you. And you're not the first person with a voiced it out. And certainly, if it has not only come up this year, but in the years prior to that as well, my only comment to that, that I can say so far is that I think as your lecturers, we try our best to give you some sort of feedback, even though it is not very specific to your question after each assessments. And I know sometimes it's very generic, but unfortunately, this is the policy of the university. Yes. You're not the only person that has voiced it. It's not the only program that's voiced it, it's also voiced and expressed in the other programs that we offer here as well. I can't really say that.

Students:

I know what is the reason behind this policy. Why are students not allowed to know what they did wrong for their exams?

Interviewer:

I think the perspective is not really to tell you... Lecturer B, do you have anything to say to this question?

Lecturer B:

Question. We follow [crosstalk 00:18:31].

Interviewer:

Because to a degree sometimes doing that is almost like spoonfeed style of learning, which is not really what we do here in university. And I know from hearing from your four responses, so far you guys are not students who require spoonfeeding the four of you demonstrate that you are able to be independent on your studies. And it's a hard balance to be able to be someone who is here to not tell you, this is right, this is wrong. But at the same time, if I give it out to the specific details already, then you're not really... we promote this, this sense of almost dependency on it. That's why we have learning outcomes. And that's why we always go back to the learning outcomes. We don't go back to your question, your exam questions, for example. Because when you practice next time as pharmacists, whether you got question 67 wrong or not doesn't really matter if you have missed the learning outcome altogether.

Interviewer:

So I think on board your concerns, I understand your frustrations. We try our best to help support you and to tell you where we can. But yeah, we are also accountable to the education committee, the university, which also does govern how we teach or what we say and what we cannot say. So yeah, it's a fine balance, but point taken.

Lecturer B:

Yeah, I think [inaudible 01:41:16] probably he already more or less... explained it as best as you can, but the truth is we don't have a better explanation than that. It's not like we were given a document that stated the reason behind every policy in the university. Potentially, as [inaudible 01:41:34] said, it is true. It does reflect. If you think about it from a more positive perspective, it does reflect reality a little better. In the real world, often times the answers may not be so straight forward, neither do you actually get the answer in front of you. If you view it in that perspective, that could be one way to look at it.

Interviewer:

And just to give you all some encouragement, at least, that all is not lost, some of your units in your year three, the assessments are designed where you are allowed, after you complete it, you will be shown at least one, which was wrong, what was right. So, but again, it's not generic for all your year two units. It's just one of the few assessments. So where we can, where we have permission, we do, because obviously we want to support you and we understand where you're coming from as well. We were students once. Or no, we also still students today, we also learning. So, hopefully they're satisfactory enough for now.

Students:

Yeah. Thank you for explanation. And I could see where the university's point of view is coming from. May I just suggest, if there are some questions that everyone did wrong, for example, and no one knows the correct answer, would it be okay to just point out just the few questions that people always get wrong and discuss about it in detail, because that means that our understanding about that topic is lacking in the sense. I think that would be balanced if possible, it's just a suggestion.

Interviewer:

Yeah. Yeah. So again, this comes back to individual units, individual academics and their own style of teaching and learning. Personally, at least for the units that I'm involved in, I do do some analysis to see, okay, let's say 60% of you guys got question three wrong. Okay. Then it does show something, and I better revisit this and just highlight some points because I don't want you all to come out of this thinking 'oh, it's fine to give aspirin to everyone.' For example, 'it's fine to do this all the time.' So I think it comes down to individual academic style of teaching, really. So I can't really comment too much on that. But point taken, thank you. Coming back to our purpose for this session, for this focus group, really is this Lecturer B give any closing comments or anything else you want to ask or add?

Lecturer B:

I think it's fine. I think we've more or less covered all the questions we have. I think we covered most of them. That's fine. That's sorted, no worries.

Interviewer:

All right. So I think if there's nothing else to add from the four of you, I do thank you very much again, for your time. Again, your comments are very noted, particularly your feedbacks, where we think we can improve. So, point taken and thank for your honest sharing. As I said earlier, you will be entitled to a 50 [inaudible 01:44:51] coffee bean gift card. Whenever you are on campus, do send myself or Lecturer B an email and we can give that to you. If not, we will see you whenever the next physical sessions that you have on campus. And that's where we can give it to you as well. Because I know some of you are living close by to a campus and can come by whereas some of you are living far away. So totally up to you whenever that suits you. If there's nothing else, thank you and have a good the rest of the day and enjoy your semester break as well. Catch up on your rest and your necessary revision, if you feel the need to. Thank you.

Students:

Thank you for this [crosstalk 01:45:42].

Lecturer B:

Thanks for coming.

Lecturer A1:

Thank you Lecturer B and Mr. Wall.

Lecturer A2:

Thank you Lecturer B and Mr. Wall.

Lecturer B:

Okay, thanks a lot for coming.

Interviewer:

Let's stop the recording.

PART 4 OF 4 ENDS [01:45:48]

Deidentified FG3Y3 - Sept 2020

Lecturer A:

... [inaudible 00:00:01] that you mentioned as well, the comments will be kept confidential. Where any data will be reported we will just quote, "Student X says this. Student one, two, three said this." Rest assured that we would like you to be as frank and as honest as possible. We shouldn't go longer than two hours, but really we want to just really focus on two themes today. The first really being this whole aspect of online learning, which most of you all have been going through for the past few months. And then, the second part of our discussion will really revolve around the design of the syllabus of your undergraduate program. Just to get things started, first off around the four of you, how has the online learning experience been so far for you? Anyone to start?

Student A:

I'll start [inaudible 00:01:14].

Lecturer A:

Yeah. Thanks.

Student A:

I think it's good at first. [inaudible 00:01:19]. I think semester one is better, but I think semester two is blur for me. I think because of discipline, which is different compared to when we go to class and stuff. We know, "Okay. We got to go to class." [inaudible 00:01:38] requires us to wake up and do our own studies. I think in terms of the exam, we are doing online exams, I feel like there can be a lot of issues. For me, I actually experienced technical issues during my finals and it was very chaotic, but the only solution they gave me was to re-take my exam, which was doing the beginning of semester two. Semester two is already so hectic, so I shouldn't [inaudible 00:02:13]. I think that's the downside of it.

Lecturer A:

Okay. Thanks [Student A 00:02:19] as a start. You did mention a lot of things that we'd actually like to go into specifically after this, but really we just want some general feelings so far on how the online learning experience has been for you guys. For the other three, similar or any differences to what Student A has shared? Again, just a very general feeling, first words, before we go down into the specifics.

Student B:

[crosstalk 00:02:51] Yeah. I'll agree. Even I experienced technical issues when doing my [inaudible 00:03:01], and then I emailed my lecturer and he said that there's nothing that could be done about it. I actually scored a very, very, very low mark. When stuff like that happens, it really demotivates you and it makes you wonder about this whole process because it sometimes seems a bit unfair. It's not really your fault.

And then, it's also a bit of a problem with asking questions because sometimes when you type it down and then when the lecturer reads it, they may not get the question at that point and they may answer something that you don't really ... That's not the answer that you're looking for, and then you need to re-email them, wait for some time and then again they reply back to you whereas if it's face-to-face, you can just walk up to the lecturer after class and then you can just talk to him face-to-face. When you talk to the lecturer face-to-face, he'll almost immediately get your question then rather than typing it in an email. There's a time factor that if you have a test tomorrow and you really need a quick answer, this method isn't exactly the best.

Lecturer A:

[Student D 00:04:18] or Student C, any other thoughts or anything different?

Student D:

For me, I think that, I don't know if it's just me or other students think so, but I think that this time has a lot of unnecessary assessments, especially for 3042 for [inaudible 00:04:36]. I think they are doing the same thing, but it's just individual and group. I don't think it's necessary. I understand what the teacher wants, what the lecturer wants. They want us to work in teamwork to solve problems together. I think that [inaudible 00:04:55] is not really necessary for me.

Lecturer A:

Okay. [inaudible 00:05:03] is more specific to the design of the whole course. For now, we just really want to focus on this whole online learning aspect for now. I think if I can summarize some of your comments so far, the general sentiment is that you all don't really prefer this online learning experience compared to your offline experiences. Contrasting it with your first two years of your [inaudible 00:05:36] program, that's the general feeling that I'm getting so far. Student C, any thoughts or any different thoughts or anything extra?

Student C:

I would say that earlier in semester one, everything being online was implemented actually pretty great. I found it easier because the lectures were all recorded and whatnot. I would say generally my own experience is better in semester one compared to semester two because with more live sessions on the briefings and lectures and whatnot, sometimes it's a bit confusing as to when is class, which link is class and whatnot. I'd say it was better implemented in semester one opposed to semester two.

Lecturer A:

Okay. All right. Just to summarize again, we like online learning because of the seemingly convenience as what Student C has mentioned, but we do not like online learning as the three of you have more suggested too is because you felt that there's a distance with the lecturer and also getting comments from your questions and you just don't like the online learning experience. Is that correct?

Student A:

There are more cons than pros.

Lecturer A:

For the online learning experience for you.

Student A:

Yeah.

Lecturer A:

Can you elaborate more for you?

Student A:

Okay. Pros is very flexible timing, being with family and not have to go out and everything. We actually have more time. We don't have to travel to uni and stuff. The cons is there's a lot of confusion. Let's say we were taught that if we have any issues we can just email or put in a forum

and everyone can learn together. It's really easier said than done because sometimes it's really hard for us to phrase what our confusion is through text. Instead, we would be more prefer it if we can do it face-to-face.

Sometimes, in class we can just raise our hands and ask immediately and actually everyone can benefit from it. Forums, it can get too ... A lot of people ask all kinds of thing. We get floods of emails. It's not always that we will actually go and read it because there's just too many. I feel that sometimes we need to wait for the response. Let's say we need instant response, but then forums we will need to wait and it's just very confusing, very, very confusing. Sometimes we don't really know which path or which way to go. In the end, we're just like, "Okay. We'll just do this on our own".

Lecturer A:

All right. Thank you. Anything else that anybody else want to add? Okay. Really, the first two questions was really just to find out what the general feeling was about the online learning experiences so far. We wanted to contrast this with the offline experiences, meaning what you've experienced in years one and two compared to what you've had this year already. We've got some good comments. Now, we're going to go down specifically to some details.

Specifically now, how do you feel about the online Zoom ILs, CTLs and the workshops compared to the offline setting? I think Student D did mention specifically now to some units where you had [inaudible 00:09:29]. Unfortunately, you did not have the chance to do it physically in the physical setting, which may have affected how you feel about it. From the general sentiments, I can gauge that for Student D, she thinks it's messy or it's ... It's just messy. I really wanted just some more, again, without going through specific units, just think about [inaudible 00:09:55] lectures so far, meaning your ILs and your CPLs and then your workshops, and compare the online ones and the offline ones that you've had in your first two years. What do you feel about it and why?

I'll give maybe some time to think about it because it is a big question [inaudible 00:10:22]. If I can maybe just put a suggestion. This is not the first focus group we've ran. We've been running small focus groups with your other friends as well. One of the comments that we've got so far that some of them did not like the online workshops because in your online workshops where we go into our breakout rooms, a lecturer may be stuck with a particular breakout room and then they don't know when or how to get the lecturer's attention. Even though we tell you that you can use this function to raise your hand, they felt that that didn't happen very ... That didn't transition well, versus in the physical setting, during a workshop or lecture you could just be standing there facing three or four tables and it's easier to get the lecturer's attention and the lecturer is able to just answer any questions openly. Is that something that you all feel as well?

Student C:

Yeah.

Lecturer A:

Yeah? Okay.

Student C:

I think that's the only downside of the online workshop. The only one is when you need a question answered by a lecturer, but your lecturer is not available because another group is ... He or she is being held up by another group. That would be the only one. Other than that, the fact that you can just be with your own group online so the discussion is a bit more focused because it's just everyone, you don't really hear any other noises.

Lecturer A:

So that's an advantage.

Student C:

That's the one advantage because you only hear your group members and you know you guys can discuss among yourselves and everyone can hear the question quite clearly.

Lecturer A:

Okay. Just to stay on that point for now, if I could ask further, some of your friends have also shared that they were not happy because in the breakout rooms when they were instructed to have your cameras on, your mic on, not all the group members followed that, and then [inaudible 00:12:28] everyone was contributing equally, but then this was more apparent or more important for them when those workshops have assessments in there. They felt some kind of injustice or unfairness. Is that something that is echoed here as well? Do people feel the same way or not?

Student A:

Yeah. I agree for this one.

Lecturer A:

Okay.

Student C:

I agree with it to an extent because from what I had seen, one of my group members, usually she stays muted, but when it comes to ... In the document when we are actually doing everything, she actually does ... What is it? She does give her part, and if we just ask questions in the Google Doc, she actually answers. I would say that so long as you are doing your part, I'm quite okay if you stay muted.

Lecturer A:

Okay. So far, I've just given you two examples of some of your friends, what they felt. Any particular that you've experienced personally? Again, it's not just the workshop. It could be your ILs and your CPLs as well. Anyone?

Student A:

You mean offline, or are we comparing semester one and semester two?

Lecturer A:

Yeah. Comparing offline and online, not comparing sem one and sem two yet. After will be a related question, but right now just compare years one and two, meaning your face-to-face experiences and this year as a whole, the online experience.

Student A:

I think for the workshop one, I agree with the part where sometimes [inaudible 00:14:20] members they are there, but they're not there. They don't really contribute nor talk. Sometimes, we don't know how to say, so we just read like this. Also, the lecturer sometimes ... Our class is only two hours. You can actually be dragged and do three hours because the lecturer spends more time one by one. To be in the class, we see one goal for everyone. The timing is like, "Oh," because we have a next

class or we have to finish by two hours. I think that's one of the downsides that I saw online. The time management is not that good.

Lecturer A:

Thank you. Student D and [Student B 00:15:16]?

Student B:

I think the group workshops [inaudible 00:15:19], especially if you have what Student A and Student C mentioned, the quiet, shy ones, it's quite hard to get them to participate. Sometimes it doesn't feel like a group discussion, and like Student A says, even I don't exactly do much about it because I know that they actually contribute in the Google Doc. But when it comes to discussing MRPs and discussing counseling points, you just don't know if they're there. Sometimes, we just have to personally call them out and ask them, "Are you okay with this suggestion?" That's the only way we'll know if she's also okay or if she has some other idea because it's a group thing. We need to listen to all their ideas because even though a group member may be quiet or shy, she may have an idea that could potentially save the assessment.

Sometimes, they just don't know how to bring it out on an online platform because most of the time our screens are all blank because we're usually off it so it's like talking to a black wall literally sometimes. I think [inaudible 00:16:30] workshop class [inaudible 00:16:31] sitting in a round table and we all have to look at each other, at some point we will just talk. It's more forceful because the person is sitting right next to you. I feel like that interactive experience with your friends, your group mates, it's very, very, very low in this online setting. There's no interaction at all sometimes.

Lecturer B :

Sorry. Do you think it would be better if everyone was forced to switch their video on in these workshops?

Student B:

I think in the assess ones, I would appreciate it more if it was on, but non-assess I don't really mind. Even though it's on, the main goal is to get the people to talk to each other. That is my goal.

Lecturer A:

Student D, anything else to add?

Student D:

I agree with Student B's statement.

Lecturer A:

Okay. What about the online lectures that you've had so far? This would include your ILs and CPLs. I think that one or two of you had mentioned that you like the convenience. You don't have to travel. You can just dial in when you need to. Anything else about it? It can be positive, it can be negative. This is contrasting your physical lectures that you've had in the first two years. I think Student A also did mention that she felt that asking questions was easier in a physical environment versus an online environment. Was there anything else?

Student D:

For me, I think that IL is good for me for sem one. I like sem one's IL design. Sem two is not good for me.

Lecturer A:

What's different about the sem two ILs compared to your sem one?

Student D:

For sem one ILs, you need to just find a time to click in and listen to all the lectures and answer all the questions to get marks. For me, I think that you can choose the time that you are really free or that you want to study to do the ILs. That way you can focus more and to get more information you need or research anything. For this way, it's like you are forced to do IL on this day. For that, you need to ... You are forced to finish your lectures two days before that, and you need to squeeze your materials between these times. It's a bit hectic for me.

Student C:

It's also hectic, especially if there are public holidays because usually we have Monday CPL followed by Tuesday, our discovery day, and then Wednesday will be the ILs. Usually, we have the buffer day of Tuesday, but if a public holiday falls on Monday, immediately after your CPL is your ILs, which barely gives us any time to actually go through the materials properly to answer [inaudible 00:19:43] properly.

Lecturer A:

Okay. Was there anything else from anybody? No? Okay. This is leading to my next question as well. We're really now just focusing on what you've had this year. We want you to contrast semester one and semester two and ask you about the organization of it. When I say organization, and we're starting again very in a general sense to what's the timetabling, the communication with the academics, the flow of the units. I think a few of you have already suggested that you all felt semester one was much better than semester two. Is that correct?

Student D:

Not really. I think ILs in sem one is better than sem two.

Lecturer A:

Okay. Student D thinks the lectures were better in sem one. What about the timetabling, the way we planned the flows in the units? Same, better, no difference?

Student A:

I think it's very hard to say that because first sem we really had more units. To be honest, I do feel more hectic in sem one compared to sem two. The only difference I felt between the two is sem two at first it's very hectic because we feel like we don't really know where to go. It's very blur. That's the [inaudible 00:21:25]. In terms of the weight of the units, I don't really feel that it's that bad as compared to sem one. Sem one was very, very heavy for me. It's also self-regulating and everything. I felt more stressed out for sem one, but after sem two I think the first week was the most stressful, but following weeks then I feel like it gets better.

Lecturer A:

Okay. What about the rest? Any thoughts, comments?

Student C:

I would sem two's timetabling is more organized compared to sem one because usually I'm the one communicating with the UC's. I would always have to check between my two UCs to ensure that

classes don't clash, especially on days we have workshop because there's always a time where a 1:00 to 3:00 class can happen while we're having a 2:00 to 4:00 class. I have to communicate with both UC's and ask who wants to move their class when?

Lecturer A:

And Student B?

Student B:

Just about the [inaudible 00:22:47]. I feel like we should not have it the day after we do discovery because discovery is the first time we're exposed to newer content. It's probably going to be the first time you're actually reading it in your entire life, something like that. And then, the next day you immediately have to go and do a test in the morning, which is not teaching you anything. It's just making [inaudible 00:23:09] and be like, "Oh my God. I read it, but I don't know how to answer this. Does that mean I don't know what's going on?"

I feel like the [inaudible 00:23:15], maybe if you move it to CTL after we finish a new chapter, after we ask our questions and after we actually understand what's going on discovery, and then we do a test, it makes more sense. We're testing our knowledge of how much we know. We literally just read it and we don't know everything. Last sem, that was what IL was about. You do your discovery and then they have all these interactive questions where the lecturer actually explains stuff to you. And then, at the end only you have that question where you get graded. It's still better compared to doing the question at first. Also, the ILs now, they just only focus mostly on the seven questions that we did during the test. It doesn't focus on anything-

PART 1 OF 4 ENDS [00:24:04]

Student B:

During the test, it doesn't focus on anything a bit broader. Sometimes the answers to those questions don't really cover everything in discovery, so it's like not getting any extra information on other parts, we're just getting information on those seven questions. There's no other extra activities most of the time, and there's so much you can explain from just one question, because it's just MCQs it's not long SAQs also, it's just an MCQ. So there's not much a lecturer can explain based on just one MCQ. So I feel like they're not covering most of the content in discovery, and discovery for this module especially, is probably personally my hardest. So I feel like there should be a bit more explanation involved from the lecturers for better understanding.

Lecturer A:

Okay. Anything else from anyone?

Student A:

I want to add. So like in our discovery this sem, right, there's key concept tasks and stuff like that, but they're not actually discussed fully. So we are just like... So I think the iRET, tRET thing, yeah, I agree what you're saying Carrie, but I think if you really want to, if we want to know if we actually understand a topic, check your learning is actually more than enough to help us check where we are and also the key concept path. We can self answer first and then maybe during IL can ask the [crosstalk 00:25:37] lecturers to, yeah, to go through, because I feel like a lot, I actually really answer all the key concept tasks, but sometimes I actually don't really know if I'm correct, but the thing is IL they don't say, they don't really, I mean, they probably discuss one or two, but not really. Yeah. So I feel like there's kind of redundance since you put it into discovery, but we're not talking about it. Yeah.

Lecturer A:

All right. Thank you. And this is the next question, again similar, some of you all have already alluded with already, but we wanted to find out what were your initial concerns in March when everything all was happening and we told you all, we're going to flip everything online. Were there any initial concerns and just maybe another way to say it, how were your feeling towards it? That's the first part of the question. The second part of the question, now that you have gone through quite a few months of the online learning environment, are those concerns [inaudible 00:26:42] and again, we're asking a general feeling towards this whole aspect of online learning, not specifically just on your iRETs or your tRETs, but really this whole online, the new style, if you might. So yeah. So two parts again, what was the initial feeling? And then is that feeling still there or has it been addressed or yeah?

Student A:

I'll go first then. The first feeling I had was, is there any discount for our school fees? Because honestly speaking, this whole COVID thing did impact the economy as well as for all my family. So it really did take a toll on us as well. So I was kind of, because I was staying in KL and I was alone. So I was away from my family. It was very hard for me to manage everything. So I was worried about tons and tons of things, the economy being my main. But we did write petitions and stuff, but Monash couldn't. Yeah. They just... Yeah. So that was my first concern. And the second was that, was I able to cope with it, but throughout my first year and second year I always sleep in class.

So I know that maybe I can actually self regulate in some point, but I wasn't sure if I could do this, but after this COVID thing, I did realize that, okay, basically this is uni, right? All unis are the same. You go to UCL, you go to very famous unis, I heard people saying, "Well, at times you need to do it yourself, it's like a self-driven thing." Yeah. So I think coping thing is better but the financial thing, I think it's still on like, yeah, it's still a concern to me.

Lecturer A:

Thank you. Thank you for your honest sharing. The other three, any thoughts?

Student B:

My first concern when they said online was wifi, because I live in student accommodation and if you want to get something changed or fixed, it's a bit of a process. You need to call people and they don't come immediately. They'll take a few days and even if they come fix it, they'll be like, "Oh, I fixed it. It works fine." And then two days later, you'll see this notification says internet connection is unstable. So it's a bit frustrating that way.

And like I said, I personally experienced problems with that and I couldn't do my [iRET 00:29:39] at all, nothing literally. My answers didn't even get saved. I really don't know what happened, but my answers didn't get saved. So I feel like maybe the lecturer should have a backup plan or a solution for students if something like that happens. For example, since I couldn't do my quiz, maybe they should have at least a backup quiz with different questions so that I can still take the test and it still seems fair because it's different questions. So I think they should add in stuff like that to prepare for the worst, I guess.

Lecturer A:

Thank you. And the other two. Anyone?

Student D:

For me the first, I mean, not really the first, one of the concerns I have is like Student A say, financial problems. Yeah. And the second is, what about the classes? But it's okay. I'm really okay with Zoom

classes so far, because there's nothing that we can do. So why not we just adapt to it. Because I understand the COVID situation and as for the money, actually I communicated with a lot of people and they say that it's not just about the utilities fee or anything that concerns like, if we are not going to school, we don't need to pay for all these fees and all those. Yeah. We need to consider about the professors or anything, especially for private university. Yeah. So there's nothing that we can do about it actually. Yeah. So after that I know about this and I'm just like, "Okay", like that.

Lecturer A:

Okay. Thank you. Anything else that anybody wants to add or...?

Student C:

Initially for me in March, when they announced that the entire semester's going online, I immediately felt stressed because I knew I was the middle person between the UC's and the students. And because we didn't have a clear timetable as to when classes are like solid, fixed time. So it's always up to me like lecturers will announce in the forum, "Class will be from this time to this time." And then the next thing I know I'll be having messages, "Don't we have class from", lecturer announced a class from one to three and the next thing I know I'll be getting a message saying, "Don't we have a class two to four? Will it clash? Won't we clash?" Which comes to me having to go back to the UC's and rectifying all of this. So I think it was up until, almost end of the semester I had to keep doing this. Yeah. Then came semester two with a fixed time table and I was like, "Okay, I don't have to do this."

Lecturer A:

Okay. Thank you. Anybody else has anything else to add?

Student A:

I think I have one more thing that was a bit concerned was the thing that, because I'm always very active in uni. After class, I will always have activities. I'll join. Yeah. I'm very active person. So that was actually the only escape I had from stress and managing pharmacy because yeah... So when they announced online, I felt like a lot of my social activities were just stripped away and being alone in my room in KL and not being able to go out was very, it was mentally and physically torturing for me. Yeah. I only have one room and that was very hard for me. And because last time I would have all my the time they're out, because actually if I have more things to do, I will manage my time better. So if I know I'll have rock climbing that time, then I will need to finish this by then.

But under COVID situation and being in that room, I felt like all I had was time. So the moment I took time off to do something, I will feel like, "Oh no, I need to go and study because it's all self regulated. Am I doing enough?" So I was very, very, very stressed out during sem one. I would constantly study and study because there's nothing else I can do besides that. Yeah. Yeah. I think that was mentally challenging for me, but after this sem two, I realized that it wasn't worth it because it was just very hard. And it took some effect on my general health as well, because I will forget to eat and things like this being too busy, dealing with all online things.

Lecturer A:

Thank you, Student A, for your honesty. Can I just ask a bit more and if you don't feel comfortable answering it, that's okay. You can just decline.

Student A:

Okay.

Lecturer A:

So first part, do you feel you're in a better space now?

Student A:

Yeah.

Lecturer A:

What, why? What has changed because we are still learning in an online setting?

Student A:

Okay. So during sem one, I think I've never worked so hard in my life, to be honest, and the results I got did not reflect the hardwork that I put in. So I just felt that I sacrificed so much. I wouldn't even go out with my parents because I would think I need to study. So I actually, gave up all the quality time and I just felt like life is so much more than that. So this sem, I actually started doing a lot of things besides studying. And I felt like by putting more activities in my life, I can fully appreciate everything. And like I said, if I have more things to do, I will manage my time better. Yeah. So I feel like results isn't everything.

Lecturer A:

Mm-hmm (affirmative). All right. Thank you. Thanks for that. Okay. I think we can proceed to the next question. And this is going to be a bit sensitive. You have been introduced very gradually to this whole brick wall of research in your semester, this semester. Can I just ask again, how do you feel about it? And do you all feel that has been useful or do you feel... Do you do think it's useful or do you think anything else? And you can be as honest as you want and again the responses are kept confidential and will be shared as well.

Student B:

I think it's useful. At the longterm run when you think about it, it's useful. But if you're someone who just focus on, "Oh, I have finals. I need to study for finals." At that point, it becomes like a bit, is it really necessary? Something like that. But in the long term run if you want to carry your research, then yes, this is very useful. But I know we had a bit of a research thing, like professional practice earlier, but I feel like when they put research in this semester, it just came out of nowhere. It's literally just one research module that just came out of nowhere. Because last time we only had like CCU modules and then we're so used to that format.

So when this research module came in, initially when I first opened it, it was a bit of a, more of a panic thing. Because I'm always used to reading discovery and science stuff and management plans. And then now this is something completely opposite and different going on. And you have the CCU staff going on on one side and then you have these statistic tests and ethics and other stuff going on at the other side. So it's a very, a bit of a disconnect and you need to manage your time appropriately. So yeah, back then it was just CCU discovery. It's always management and plan, management plan, rationale management plan, called stand point. So you'd just have to like follow that order. But now it's like two different modules going in two separate ways. It's something like that. Yeah.

Lecturer A:

Okay. That's a very good point. Thank you for that. I appreciate that comment. Thank you. Anyone else has anything to add?

Student A:

At first, when I saw this research and I think at first everyone was very revved up. So among our friends, we will say like, "I think this research unit should only be given to those that are interested in research." Because I think most of us may not be interested. But after a while I started doing my own research and I actually, besides Monash, I do participate in other research and surveys. Yeah. So actually, I was just doing the surveys just for the tokens at first. And then I slowly realized that the importance of it after I started doing my placement in Caring pharmacy.

So Caring is a kind of pharmacy that they do have evidence and research to support their products. They are very patient centered, so let's say they support something, they will always, they have something to back them up, their evidence. So I feel like that's the importance of research. That's when research comes in. We need to know why it's important to know what is good and best for the patient and not just sell things off like that. So that's when I realized the research is important, but yeah, I think the main point I want to say is, I think not everyone is meant for that. And if I'm not meant for that and I'm to do this unit, obviously I will feel like it's unnecessary for my future. Yeah. But it's a good exposure.

Lecturer A:

Okay. But I also wanted to find out about this [inaudible 00:40:13] about learning research online. How do you all feel about it? Has it been okay or you don't like it or?

Student B:

It's very confusing. [crosstalk 00:40:25] Having to learn it online is very, very, very confusing.

Lecturer A:

Thank you. So that's why I was, so [Denny 00:40:31] feels that it's confusing. Okay.

Student B:

Yeah.

Lecturer A:

The [inaudible 00:40:33], you're nodding your heads as well. Anything else? And so really just focusing on the online aspects. Some of your reaction about just research, and I want to know research online. Anything else?

Student B:

Harder to ask questions, because like I said, it just gets miscommunicated along the way. And if you really want to get it done fast and you're waiting for an answer, you just get a bit impatient. Because it really, really gets miscommunicated along the way. Yeah. And sometimes that miscommunication may result in you having to redo everything [crosstalk 00:00:41:06].

Student D:

Further confusion.

Student B:

All over again.

Student D:

You get more confused. Yes.

Lecturer A:

Okay. All right. Thank you. I'll go to the next question that then now it gets more cardinal. How do you all feel about assessments in this online environment that we have so far. Some of you all have said some points already, but now we're going to give you a chance to say a bit more if you want to. Student A, you want to start? You were nodding your head already.

Student A:

Okay. So I think my problem with my assessment, it was just actually sem one. It was during my final, so like that whole [inaudible 00:41:45] with it. And the fact I was wronged for not calling the helpline. Okay. On separate note, after I finished my exam, I called the helpline and the helpline had like piano, hotel music and welcome to Monash. How am I supposed to still be calm during exam when I had to call the help line and they go, welcome to Monash. And I mean, I was panicking during exams. So it was so hard for me. I mean, I know that there's a helpline, but what I haven't called because the time was, it was time pressuring. So all I felt was, "Okay. I'll just keep going even with these technical errors." So my technical error was that when I type something it overrides the next letter. I switched keyboards during the exam.

And there was nothing. I didn't press anything like insert or anything. So it was only at that platform that, that happened, like other platforms I type it was okay. Yeah. So it was very nice for Ms, I forgot her name, I'm sorry, that she felt compassionate and then after that, they offered me to retake. So I feel like they should have a prepared questions, like prepare a set of questions. If I could have just done my exam again, the next day, I will still be able to have it fresh from my memory, but to have it during semester two at the beginning, it's just very stressful.

Student C:

I can vouch for that. I took it.

Student A:

You took it?

Student C:

Yeah. I think it probably was, I would say it was so stressful to the point where I ended up not passing the paper. So I am sitting here taking intermission. So it's like, I would say the amount of effort I put in in sem one to a point I failed the paper. And then I was so stressed. In sem two I had to find time to space out sem two's activities and studying back for that paper. I would say the effort was just not worth it, because in the end I didn't even pass. I got 849, which was very painful because I could not sit for the paper again or have it re-checked. So, I just had to take an intermission.

Lecturer A:

Okay. Thank you for your honest sharing, but I really wanted to find out, so to summarize both Student A and Denny's comments that you all felt stressed, very stressful in this online assessment. Why? And again, contrasting this to what you've had in the previous years. Why do you all feel this sense of stress?

Student B:

Because if something goes wrong, you literally cannot raise your hand and then invigilator will come to you. You're literally sitting in a room alone so the first thing you do is panic and panic.

Student C:

[crosstalk 00:44:49] And we have to make sure your wifi is completely stable.

Student B:

Exactly.

Student C:

Because in Monash everything is connected with the LAN cable, so you don't have to worry about your internet connection. You just have to worry about your PC dying. But even then, it's because happening in Monash, they can get it rectified almost immediately. But at home, if it's your laptop that's crashing or your wifi that's crashing, you just going to step up on it and you really can't answer your questions anymore.

Lecturer A:

Okay. Yeah. So that's what we are trying to find out a bit more.

Student B:

It's easier to find help if you're doing the computerized exam there, than doing it at home. Because if something goes wrong, there is a backup PC and plenty of wifi. But if something goes wrong, I live in student accommodation like I said, if something goes wrong in my room, I only have one laptop and one router. I have no way of going anywhere. And the exam is at eight in the morning. I'm pretty sure management's not even open for me to do anything. I just have to like sit and wait, which isn't going to help.

Lecturer A:

Okay. Yee [Huey 00:45:46], do you have any to add?

Student D:

I agree with what they say. Yeah.

Lecturer A:

Okay. Well personally, now to give you a second question to link to it, do you feel that the assessments in this online setting is actually a true measurement of your knowledge and skills? Okay.

Student B:

No.

Lecturer A:

Everyone is saying no. Can you all, can anybody want to expand? Say why do you all feel that way?

Student C:

So, because it's an online assessment, we don't really measure our understanding based on our discovery. They take more resources from other online things like those guidelines and ETG and all those. Yeah. So initially for people like me, who really just focus on discovery and whenever the assessment starts and it's all about not, it's not everything about discovery and it's about ETG and all the guidelines and panic like, "Did I really learn this before?" Something like, "Did I really miss this? Or I'm pretty sure I read everything about the discovery. So why something like this over here." Same goes to the final exam. It's really all about ETG and everything else.

Student A:

I want to ask if this is how it was carried in the previous years or is this the first time they try out? Because in last year we would have our AMH with us, like a physical book, but now it's like, we have to open up tabs and tabs and tabs and we only have one computer. So it's very hard to go back and forth, back and forth like that.

Lecturer A:

Yeah. So even though we tell you, that it is an open book exam, I think we do expect that students should be able to at least attempt some parts of the questions on their own and only need to read so when necessary. So I think where some of the students face some difficulties was they went in getting ready with 10 tabs open and then that gets a bit messy. And then they get lost in the resources. And they don't know how to answer the question. At least, when I was talking to some students, I was telling...

PART 2 OF 4 ENDS [00:48:04]

Lecturer A:

Right. When I was talking to some students, I was telling them, just treat it like a closed book exam, but you actually tend to check your references and study like you're preparing for an exam, so that's on that part. So coming back to the question, you feel that it's not a good assessment of your knowledge and your skill.

I think we don't need to ask...elaborate more on that, I guess the final question for this session when you were asked...with this first part was really to find out whether you all face challenges during the transition. And I think again, I would just say yes, as quite a few of you all already shared some very personal examples of the challenges that you all faced. Lecturer B, do you have anything else to ask or comment on this?

Lecturer B :

I guess just that a lot of you have focused more on assessments that are...MCQ, SAQ those type of written, I mean online e-assessment type. How about other types of assessments? Workshop based assessment...Do you have anything to say about those or standing issues?

Student C:

I do have something to say about the workshops. So I know we are all asked to do our own like pre workshops and things like this but then answers will not be given. So it's very...so sometimes for us in class, in Monash, sometimes the answers will be put on and then we can actually take a picture, but this one is a discussion. Then obviously they can be different answers to things like these.

So I remember I had an assessment last time. It was something like a workshop and it was individual and they were different...Obviously we have AMH, ETG a different kind of resources. And because I use one resource and the other resources say can, and one other say, no. I use the no one, I got my answers wrong and it was just really...To me, I just couldn't understand, there's so many resources, how are we supposed to decide?

Which is which, which is correct. And then when I justify my answer, the response I get was "you should check other resources as well, and then pick the major one." So, I mean we are asking you for new resources. So these are resources that were provided to us. How do we know how to say that? And it's not that I didn't justify that I used; I didn't use; I didn't write that. I used this resource, but still I got a zero mark from that question.

Speaker 1:

I think [inaudible 00:50:51]

Student A:

Yeah I think it was something you [crosstalk 00:02:54-58] and also the previous one, I think the anti-platelet thing . I just hate it.

Lecturer A:

Okay. Point taken. Thank you. If, there's nothing else Lecturer B?

Lecturer B :

I'm good, that's fine.

Lecturer A:

Yeah. So the next section of our focus group really is then focusing on the design of the program. The reality is that you guys come into the Monash Pharmacy program, you are given Discovery materials, your lectures are more interactive. You have quite a few more workshops than some people do and then, you finish the week with a cluster group.

So that is unique to how we teach Pharmacy here at Monash and it's slightly different to the other universities, or even some of the other programs out there. So really we want you to think about those aspects and in answering the next few questions. So just to start off the discussion, what do you guys feel about your Discovery materials? Is there anything you like about it or is there anything you don't like about it and why?

Student A:

Initially Discovery was very in depth last year, previous years and all. It had the information you needed to get to your final year assessments for your own knowledge. But now, Discovery is basically... Refer to this link to complete this task, refer to that link, to complete this...It's literally just a bunch of links and I'm okay with links. I'm okay with using ETG, but this term, the links literally go to all these PDFs, which are 60 pages long and even scrolling through 60 pages would take some time. So I feel like maybe if they would at least specify, go through pages like 20 to 30, or maybe 30 to 35.

It'll be a bit easier because [crosstalk 00:53:02] because you're really reading all this and then you're just like "Oh my God, do I have to know this? Do I have to know this? Is this part of my syllabus? Am I going to be assessed on this? Do I have to-"...You have all these questions running through your head. We don't know if we're supposed to literally know everything in that 60 page document, or is there a specific section that we're supposed to focus on because there's no information regarding that.

Lecturer A:

Okay, thank you. Student C. You said something just now, I missed it. What did you say?

Student C:

On Zachary's point is, I'm also completely fine with links, but if your entire Discovery is links, you kind of just question, what am I paying tuition fees for?. Also, it's very easy to get sidetracked because when I read a link. I tend to get very interested in other topics and from there, I just completely sidetracked off the Discovery and I'm like "Wait, I should come back". So I have all these extra knowledge, but it's kind of pointless because you don't have a chance to implement it unless you go out and work so to say, in terms studying and getting through your semester, it's kind of hard because it's very easy to get sidetracked.

Lecturer A:

Okay. [Lei 00:54:17] How do you feel about that?

Student D:

And they are not just writing you one link, they are providing a few links with different statements, as Student A said. You need to consider on which one you need to refer.It's quite hard.

Lecturer A:

Okay. Student C, do you want to [inaudible 00:54:39]?

Student C:

Okay. So I realized that there are a lot of optional activities. Obviously you skip it because you have too many things to do. So during last term exam, there was actually a question that came out there was under optional activity. So that kind of shook me because I knew that optional we obviously wouldn't get tested on it because it's optional. So there was... I thought that was really unfair. I do agree with the comments of things, especially up to date. So I remember, I think it's, I can't remember if it's chapter two or chapter three for this term, there were a few resources. I think it was stroke. So there was like the stroke. Why should we do this and that? And then there's up to date and then they give different things, different suggestions, but we need to go to...we need to use this resource and not up to date because it's more updated.

So are we supposed to go so in depth to know when was the publish date? When was the one, which one is the most updated. We are constantly, we don't...I do understand that we were exposed to so many resources to give us the idea that we cannot just do...We cannot be fixated to just one, but then during exam, are we guaranteed that if we were to quote this resource, we will still be given the marks or are we going to get a reply that, "but that resource is more updated you should use that."? That's what my concern is.

Lecturer A:

Okay. If I can just summarize again, just trying to be consistent. The feelings about year one to year two Discovery materials were a bit more positive than year three's Discovery materials and the concerns or the negative things that you have said about year three's Discovering materials that yourself*** it's not a very clear, and this is a bit messy or disorganized. Is that correct? Again, you can correct me if I'm wrong. I'm just trying to summarize.

Mihauel:

That's the way we feel, yeah.

Student A:

It's also really big. I feel like it does not hit the point. It does not have the information that we need right now.

Lecturer A:

Sorry for that, so the point of big, is that the same across all three years or is it just for this year?

Student A:

No I was fine with Discovery for year one and year two and year three, sem one was also okay. But this year, especially Accurate Care. I just feel like once I print out, I just feel like there's nothing literally in Discovery, just links and the links just don't...are not really working for me right now.

Lecturer A:

Okay. All right. Thank you. My next question now is in reference to activities that happens in class. So the reality is, well, again, your lectures are a bit more interactive. Your workshops are a bit more Play space particularly for your course work and places in uni's What do you all feel about it? Do you like it or do you not like it? And if so, why?

Student D:

I came from A levels background. So A levels is basic for me that time, I think it was very self regulated because to get tuition, you need to pay a lot. So I think A levels did train me to self, do things and not really need the spoonfeed way. So I think I actually quite like these active learning it. If you don't understand something you just go and sort it out yourself. I think that's how it should always be before you just...You don't understand, you just go and ask someone the first step is to try to solve it yourself first. So I actually, I quite like these active learning and a lot of interactive, we can just learn together. When we talk to each other, it's a good thing. Yeah. I like it.

Student A:

Yeah. I also like it when, in workshops, we are given cases instead of very factual kind of questions because it does prompt you to think about it. Then if your group mates say otherwise, then you guys can discuss as to why it's like that. So I would say it's a good way for us to actually implement what we have learned by giving us case studies to do. So at least, we know that these kind of things can happen and they may be opposing ideas as to what will be the best kind of treatment. Then we can actually discuss among each other why this would be better instead of that. Yeah.

Student B:

I like the fact that for this term, that the workshop group assessments are pre done. You don't have to do it at your workshop where we've been usually doing it like that, because that adds a lot of time pressure. You literally one and a half hours to discuss everything among your friends, clarify all your doubts and submit it. But this one, we usually do it at our own convenience.

Usually after dinner in the night, my group and I would meet up and we used to spend more than two hours, you can spend it... We can practically spend any amount of time, because some submission is usually on Thursday. So you just started like Wednesday night. [crosstalk 01:00:14] So yeah, about a day. So that gives us so much time for like clarifying all our doubts, the confusions, making sure everyone agrees with the MRP.

Whereas back then when it was face to face, we had to do the workshops at class in that one and a half hour timeframe. So that doesn't really give much opportunity for the group to discuss and clarify any doubts in MRP's and stuff. So I like that part in this semester.

Lecturer A:

Yeah. Lei, do you have anything to add?

Student D:

No, basically...I enjoy interactive designs yeah. It really helps us to focus more on ourselves and not just to rely on the [inaudible 01:01:03]

Lecturer A:

Okay. Maybe to help contrast this a bit better. So Student A did share that she did A level in the ***background, can I just ask briefly for the three of you, what was your pre new background? So for Student C.

Student C:

Macquarie's

Lecturer A:

Student C was Macquarie is it?

Student C:

Yeah.

Lecturer A:

[Student B 01:01:23] ?

Student B:

A levels.

Lecturer A:

Okay, and [Mihauel 01:01:26] ?

Mihauel:

A levels also.

Lecturer A:

Okay. So I guess Student A did share some contrast already, but so the rest from the three of you... Contrasting with your learning experiences in your pre uni and to the style that you've had in your undergrad so far. How do you all feel about it? Is there a feeling you feel, do you enjoy your [pre uni 00:01:01:54] better or this, or do you like this and it's very open for -

Student B:

Okay. Yeah. The thing about pre uni, that I really liked is that they were past year questions that I could always do. And they're a mark schemes that I can always refer to because sometimes what you... You're reading information, but you also need to know how to answer questions, which I feel like Monash doesn't really teach us before finals. We only have one paper and the one paper that should teach us how to answer question first for A levels, you have five, six, seven years worth of questions because it's different from studying.

You need to know how to incorporate the knowledge that you have in answering a question. You cannot just memorize from here and just throw it out there. It needs to answer the question. I feel like Monash doesn't really give us practice in terms of answering SAQ questions, because most of our assessments MCQs and then our group assessments a mark**8

And then we go for finals and then there are SAQ questions for like five marks, six marks where you need to write down steps and processes and stuff like that, which we don't really have any training to. I'm answering this questions based on A level change, which I probably did so long ago, I feel like Monash can maybe incorporate like more past year questions for students to practice. If we can do it on our own, and then maybe later they can discuss the answers with us, stuff like that like how to answer questions better.

Lecturer A:

Any, any other thoughts from the rest?

Student D:

Either or is more or not tuition centered like when you go in and you sit down and the lecture is just tell you everything and you just study; this study; at this point; this times. But for Monash, it's more like guys study everything, do your research everyone discuss about it and then they give you case studies and you will just do it. So at first it really takes time to adapt to all these efforts.

I will complain to why not just sit down and teach us everything that we need to learn. I got used to it and it's actually interactive lecture like you said, I mean, interactive designs are actually good for us in the long term, especially for medical students like pharmacy or medicine doctors, because they need to... The materials are always outdated.

So you cannot just be focusing on the materials that your teacher gave you during your university period. When you go out for work, you need to refresh your knowledge by knowing on how to get more updated knowledge or updated information from anywhere when you are. I think it's a really good thing for us in the long term.

Lecturer A:

Okay. So you did share also that you initially thought it was very messy and you're complaining to people around you and all that, and then after you got used to it, right? Can I ask when was the point that you got used to it? [inaudible 01:05:12] when?

Student D:

I got used to it during year one. It didn't take long for me to adapt to it.

Lecturer A:

Was there anything that triggered it or is it just something that you just thought I'm here already?

Student D:

No. Just got used to it.

Lecturer A:

You just got used to it, okay. Is that the same for the three of you all?

Student A:

I...Because during my A levels, I mean, most people agree that levels is quite like a spoonfeeding you know what to do and stuff like this, but I guess for my experience because I took four subjects. All science subjects and one maths, so for my physics, I didn't really have a very good lecturer the school couldn't do much about it. So I actually took a break from towards the exam.

I actually withdrew myself from the class and I signed up on the exam, but I would not...I wasn't in class. So I felt like that was the only way that I could was just do self study things. So I think there was a point where I started the active learning. So anything I don't understand, I'll just look it up on Khan Academy and things like this.

So I think that actually prepared me cause I didn't actually... I used to think that the more prestigious the uni is obviously the better the education is but, I thought of my friends going to UCL and they don't feel like they don't pay...The amount they pay is just not worth it because you're just going to class and you're doing active learning. But I think, this is how uni should be eventually when we go out and go to work we can't always expect for this... We need to have some initiative.

So I think this part of Monash, I was really grateful that A level exams in some point prepared me for that and. I am really grateful that Monash implement this and not the spoonfeed away. I think some uni's had it easier because I just see my friends just starting off slides and they're used to exam.

So they know as long as they study their slides but for us, we need to understand. We ended up considering these factors and things like this, which is really important because when you come and work and what is not just this, this, this, it's a very flexible thing to think it helps us think. I think that is good.

Lecturer A:

Thank you. Student A, can I proceed? Is there anything else that you want or other people to add or?

Student C:

I'll also for me, the transition from Macquarie to Monash wasn't difficult.

Lecturer A:

Okay. All right. So during the-

Lecturer B :

[crosstalk 01:08:04] sorry can I ask? Student C, why wasn't it difficult?

Student C:

Because I think for me once I was done with SPM and I kind of heard from friends about the A levels was basically just another round of SPM. I just told myself "I do not want to study everything and want me to everything out in one final paper." So I opted Macquarie which was semester based.

And like we had our own assessments there and I would say I did have some spoonfeeding in Macquarie, but for the most part of it, my sciences were mainly you had to go in such it by yourself. So coming into Monash in year one, it was still. I didn't have that deep of a knowledge, as those who did A Levels, but I could cope with the design of the entire program.

Lecturer A:

Okay. Given a choice, and this is a hypothetical question because you are, you are in year two **to this though. Would you still like to learn a pharmacy in this style? Again, yes or no. And then if no, or yes. Why this...Which part do you like? Which parts you don't like? I would like to ask the curly questions here. Anybody?

Student A:

You mean would I still take Pharmacy as my course?

Lecturer A:

No, not so take pharmacy as your course but more so going to this style or actively. As you make reference to these ways of active learning where we get you to-

Student C:

By the design of the program, how it's conducted?

Lecturer A:

Correct.

Student A:

I would.

Lecturer A:

You would okay. Lei?

Student D:

Yeah.

Lecturer A:

All right. Thank you for that. So the next question, I'm not sure whether... We'll try, we'll attempt this question, but this style of learning this active learning, do you think it works for all the different uni's that you've had so far? Or do you think some uni's work and some uni's it doesn't work? Because-

Student C:

Yeah. It doesn't work for research.

Lecturer A:

Thank you. You understand the question then? All right then yes so Student C and Sankeri agree that it doesn't work for research. Is that the same feelings for Student A and Lei?

Student B:

Yes I agree.

Lecturer A:

okay. Anything else? What about your other uni's that you've had since year one?

Student A:

[inaudible 00:22:39-44] I think it's, especially for a professional practice in Australia.

Lecturer A:

Hmm. Okay. And you-

Lecturer B :

Sorry could you elaborate the why of...It doesn't work for research you don't think it's useful for professional practice reasons?

Student C:

For me, I feel research is something that if you have no background on it and you are asked to study it by yourself. You will have absolutely no clue what is going on. You need some form of guidance, especially if you're someone who's very, very, very new to research to say, you probably need a specific tutor or have a big group and go through what actually research is.

Instead of asking, giving you the materials to read on your own. And then you're just coming out, even more blur, to say the more science part of it is something that we are quite exposed to already. So if we don't know anything, can go and research on our own and of find the answer. And it makes senseless, but basically something that is very new to most of us. So when, we have absolutely no background in it and you asked to do everything yourself. You'll just be like, huh. You need someone I would say for research because it's like..it's not something.

PART 3 OF 4 ENDS [01:12:04]

Student C:

You need someone, I would say, for research because it's not something like a science, where you can go and you can find answers immediately. Research is very, very, very broad. Some may say yes, some may say no. So you kind of need someone to help you decide when is it that it's right to say no, or when is it right to say yes. Yeah.

Lecturer B :

Any other opinions on the research or the PP even? For PP it's...

Student A:

I think PP is just unnecessary.

Lecturer B :

It's just because of the contextualization, is it? You need-

Student A:

I think PP, it can be done, but we don't necessarily actually need to... I mean, okay, the laws and everything, we will be tested on it separately, right? But in terms of how we should perform as a pharmacist, I think we don't really need a reading method for that. I think we just need classes, an interactive, during in the lecture telling us about it, and things like this. Because all these PBS lister and things like this, we don't really actually use it here, in Malaysia.

Yeah and here we don't use schedules, we use Poison ABC, right? Yeah. So when I went to my pharmacy place, when I talk about schedule and my pharmacist was like, "Oh no, we use Poison ABC." And things are different, so it gets very confusing for us, especially if we are going to practice in Malaysia for a long time. And the marks are quite good here.

Student C:

I think contextualization, maybe give us a part at the end. Because, from what I remember, usually they do a PP, especially, they give us the Malaysian context of it, but it's usually just one page or half a page long. So I think maybe it could be done better by giving us a better understanding of the Malaysian context of everything. If it could be, even I think year one would to be great, where they are... What is it? Exposed to the schedules, right?

And then maybe have another few, a bit more lengthy as to in Malaysia we don't call it schedule, we call it Poison and then equip which schedule is which Poison here in Malaysia. Yeah. I think that would be better because you'll better prepare them for the law exam that will come. Yeah.

Lecturer A:

Okay, anything else Lecturer B?

Lecturer B :

No, I'm good.

Lecturer A:

Okay.

Lecturer B :

Yeah, thanks.

Lecturer A:

All right. So I think the next question really is asking a more generic question, again, this style of learning that Student A has, I guess, just this active learning, whether it has helped you to develop as a person or to grow. I think [Student A 01:15:03] also did mention that this style of learning has helped her to ask more questions, think and to... Yeah. And I guess, in that process, I'm going to infer that it's a good way of helping her to mature as a person as well. Is that the same sentiment shared with the three of you guys as well?

Okay. What do you think about this whole aspect of adapting to new situations? Does it help you to adapt as well, to be more adaptable? Yeah.

Student A:

Just to be independent.

Lecturer A:

To be dependent?

Student A:

Independent.

Lecturer A:

Independent, sorry.

Student A:

Yeah.

Lecturer A:

Yeah, independent, yeah. [Yih-hoy 00:01:15:41], what about you?

Student B:

Yeah, I have the same thought as Student A.

Lecturer A:

Okay. Okay, so then let's say, we don't know what will happen next year.

Lecturer B :

Sorry, just quickly. Do you feel that there are any other benefits to this system of learning? So you're talking about adaptability, independence, anything else you think is good or maybe even bad? Do you think there's any disadvantage, in terms of this?

Student A:

For me or like generally?

Lecturer A:

For you.

Lecturer B :

No, for you. For you.

Lecturer A:

Yeah. You don't have to give a general comment on what do you think is good or what you think is bad. Just personally what do you feel, yeah.

Student A:

I think, at first, when I wasn't very 100% into this system, I feel like it was hard to breathe because I may not always know where to go or what to find, and things like that. But after a while I do realize that, "Okay, now I know where to go. Okay, Google." And I search things out myself. I think it really builds my whole personality and changes me in different ways.

So for example, if I need to go for a holiday, normally people will go for travel agencies and things like this, but because of this active thing, I would know I would just need to plan this myself and know where to search it out, things like this. So I think in this example is just a kind of personal development that I thought that was very beneficial to me as a person. Yeah.

Lecturer A:

Anything else from the other three?

Student C:

I think mine, the one con about active learning is that I would not know when to stop myself. Because I can just continue going and going and going. And it'll take me some time before I realize I got kind of completely sidetracked. But I would say also it's good, because you do learn something new, that's out of your syllabus. But you keep doing that, it kind of takes a toll on your studies.

Student B:

You don't just learn stuff in your syllabus, yeah. You learn outside, which is good for us, I think. Yeah.

Lecturer A:

Okay. All right. I can proceed. So I'll just... Yeah Lecturer B, anything else?

Lecturer B :

No, I'm good.

Lecturer A:

Okay. We don't know what will happen next year. Let's say COVID is still around, and let's say no vaccine, and let's say this is all going to still continue. But I say "this" meaning this whole online delivery of the program. Do you all have any improvements? Do you have any suggestions for improvements that you think could be useful or that you think that you would like to see? And again, we want to take a more big picture thinking and just to consider that in your response. Or if there's anything that you like, or that don't like. And what things that you don't like, what do you think we can improve on?

Student C:

I'd prefer for a better communication between the lecturers, when in terms of planning our classes, so that it's not that much work for the students to keep asking. Like, "When is class?" or, "Why is there a clash?" So I think generally if next year we were to continue online, I don't think year four would be a problem, but since I'll be continuing that year three, I would prefer if the classes would be more well-planned and the lecturers are mobile prepared for when they come, especially when the UC has to brief the other lectures and we have smaller groups.

So I just want the lecturers to be more prepared when they come for class so that if we have any issues or any technical issues or whatnot, it can be rectified immediately instead of waiting for the UC to come in this one. Yeah. Because I think year four, you will not have a problem since it's just the placements and also the research which is done on their own. Yeah.

Lecturer A:

Okay, I'll just say that each year has its different set of challenges.

Student C:

Yeah.

Lecturer A:

But yeah, okay. And then we're just really being asking based on what you've experienced so far, and what you like to see or what you're already enjoying and if there's anything that you don't enjoy, what do you think we can improve on? So better communication is definitely one. Was there anything else?

Student B:

Just to add on for better communication because I experienced this demo, so my lecturer forgot to send a Zoom link and then all the people in the class, we just... It was still online, but we were just texting each other, like, "Do we have class? Do we not have class?" We didn't know what was going on. Then we needed to wait til the UC emailed us and was like, "There's something wrong, join this class." So I feel like better communication, like what [Danielle 01:20:58] said, is required so that the lectures and the students are more prepared, so we can both get the best opportunity from it.

And the other thing that I would add is backup assessments for... In the sense, if your WiFi fails and if something really goes wrong and you cannot do anything about it, just have a backup of different set of MCQs maybe, so that the student can retry. And since it's different, it will still be fair if they do it on the same days. So maybe something like that would also be good.

Student A:

Yeah.

Lecturer A:

Anything else?

Student A:

I agree with the part where I think lecturers should be more prepared, because this is something that I really saw during online. Because we would go into class and then the lecturers wouldn't, some wouldn't, be prepared and wouldn't even know that there was something to be done.

Student C:

Or how to answer the questions that we have.

Student A:

Yeah. Yeah, and they probably haven't gone through the case themselves. So I feel like our time is just being dragged and what are we doing? And okay, this might sound bad, but I really prefer [sem one 00:01:22:17] because I felt like the Australian lecturers... I think because they are the pioneers to access all the materials, so I felt like they were more prepared.

I mean "sem one" is we all get everything from watching our RI, basically, ILs and things like this and we're paying Malaysian fees. But I do see the difference from Oceana [inaudible 00:10:46]. Yeah, but obviously not all. Yeah, I just hope that there are some improvement in that, in preparation before class. Yeah.

Lecturer A:

So fair point. So again, just to summarize it, again, to keep it as generic and neutral as possible, that you felt that awfully you would prefer a more prepared manner from the academics, or from the teaching style and the most similarity with the content as well. Anything else to add?

Student C:

Just to add on. Very general, but sometimes also I notice that the lecturers have technical issues in using the softwares themselves, like sharing screen and posting comments. They're struggling a bit here and there and then. Yeah, so that could also be another preparation. Like the poll EV, sometimes they don't on it and then we have to text them and be like, "Miss, or Mr, your poll EV's not on." So stuff like that. Because we are already assigned for EV, so better preparation means better learning experience for us.

Lecturer A:

Okay. Okay, on the only other hand... Okay, so thank you for all those comments so far. On the other hand, let's say if we are allowed to come back to campus and things can go back to normal, or whatever normal is, back to pre-COVID. Was there any things that you've enjoyed this year that you'd like for it to continue next year, or whenever things go in the next semester?

Student A:

Recorded lectures.

Lecturer A:

Okay. Yeah, that's one good example. So thank you. So you like the recorded lectures and you hope that that could continue. Anything else?

Student A:

I think that's all from me.

Student C:

I think-

Lecturer B :

When you say "recorded" you mean pre-recorded, that means you see them on your own time, right? Okay. Because we always do record lectures anyway in the normal sense.

Student A:

Yeah, yeah. I mean the sem one style, I like it very much.

Student B:

Yeah, pre-recorded lectures. Yeah.

Lecturer A:

The sem one style was... You have a few because different units have different styles. Did y'all have one hour of a full lecture recorded? Or did you have... Because I know you have both three by 15 minutes or four by 15 minutes.

Student A:

It's part by part.

Student C:

Usually, we answer two to three questions.

Lecturer A:

Then you can get to the next part, right.

Student C:

And then the video that's explaining the questions. And then the next three questions, and yeah.

Lecturer A:

So do y'all like that? Or y'all don't like that?

Student A:

I like that.

Student B:

I like that.

Student C:

I prefer that.

Lecturer A:

Interesting, yesterday's group hated it very much.

Student A:

Oh.

Lecturer A:

Interesting.

Lecturer B :

Why do you like it?

Lecturer A:

Yeah, why?

Student A:

I think it gives a more comprehensive understanding, compared to the [inaudible 01:25:48]. Yeah.

Student C:

And then you answer the question and immediately you get the correct answer with feedback and why the others are wrong.

Student A:

Yeah.

Student C:

It's more well explained in that video than what's happening at class right now.

Student A:

Yeah, with the [inaudible 01:26:06].

Student C:

Yeah, and it's pre-recorded also, so if you don't understand, you can just scroll back-

Student A:

And replay it and understand-

Student C:

Listen to it again at that same with your memory, especially thinking about that question. So you can just go back and listening to again, make some notes. But that in all, I don't see myself doing it with [inaudible 00:14:23], honestly I don't make notes, I just listen. And if I don't understand, that's it.

Student B:

I would say the live lectures they were having this semester, we could enjoy it if the recorded sessions were uploaded onto Moodle, so that we can go back, and go through it, and see what we don't understand, we can replay that specific part, and have a better understanding. If we still don't understand, then we can go and email a lecturer or whatnot.

Student A:

Yeah.

Student B:

If the live lectures were uploaded, I think we would prefer it as well. Yeah.

Lecturer A:

I see, okay. So just to get some clarity, because there are obviously different types here. If I was to get y'all to rank them, and this I want when I ask you to rank. You like one hour of live lecture online versus one hour of recorded lecture, so we've already delivered it but it's recorded and we put it up there, and then the third option is pre-recorded, so it means that the three or the four small parts lectures with questions in between. How would y'all rank those, in terms of your preference?

Student A:

For me, pre-recorded is the first one. And then-

Lecturer A:

So the pre-recorded with the multiple... So for a one hour lecture, we'll give you four by 15 minutes.

Student A:

Yeah.

Lecturer A:

Okay. So that would be the first. So is that the same for the three of y'all? Yeah, okay. And number two would be? [crosstalk 01:27:56]

Student A:

The recorded ones, sorry, yeah.

Lecturer A:

Which one the number two? One hour of pre-recorded lecture. Is it?

Student A:

The first one is pre-recorded, right?

Student B:

Pre-recorded. Yeah, the first one is pre-corded.

Lecturer A:

Yeah. So four by 15 minutes, pre-recorded bite-size. Because pre-recorded has two types.

Student A:

Oh.

Lecturer A:

Yeah, there's the four by 15 minutes or a straight one hour.

Student A:

Okay.

Lecturer A:

Yeah, but you can-

Student A:

The 15 minutes. The first one will be the 15 minutes.

Lecturer A:

Okay.

Student C:

The second one would be the one hour stretch.

Student A:

Yes.

Student B:

Yes. And the third is live.

Lecturer A:

I see. Interesting. So can I say that y'all actually don't like a live lecture? A live online lecture.

Student B:

Live online, yeah correct.

Student A:

Yeah.

Lecturer A:

You don't like it?

Student B:

No.

Student A:

I think that because live, let's say, if we don't understand something and then we have to go and watch again. So that takes up two... We have to repeat twice. But then if like pre-recorded, if we miss something, we just have to go back a bit. There's just only one time. We only have to do it once.

Student C:

Time is an issue with live online lectures.

Lecturer A:

Sure.

Student C:

Yeah, because when the lectures are recorded, at least we can go through it in our own pace. If we don't understand, we just go back. But if it's a live lecture, if you don't understand, it may take up another 10 to 15 minutes to re-explain everything.

Lecturer B :

Sorry, if I bring in one extra option into the mix. So if there is a fourth option of live, real-time lecture in a lecture hall, would that suddenly rank the highest? Compared to pre-recorded lecture.

Student A:

No, I would still prefer the 15 minute pre-recorded.

Student B:

Yeah.

Lecturer B :

Oh, okay.

Lecturer A:

Interesting. Okay.

Lecturer B :

So, but I assume the online live would be ranked lower than the real-time live.

Student B:

Yeah.

Student A:

Yeah.

Lecturer B :

Would that be it?

Student A:

Yeah.

Lecturer B :

Okay. But all options of pre-recorded would still be our first and second rank?

Student B:

Mm-hmm (affirmative).

Lecturer B :

Okay.

Student C:

I think pre-recorded is good for lectures. I don't mind the system where lectures are pre-recorded, and we do it at home in our own pace, and then we come to class or workshops. And at that moment we can just clarify our doubts after workshop. I would prefer a system with that approach because that gives us more time to do discovery, more flexibility. We don't have to rush to class because even though you're in class a one hour, that doesn't mean your brain is super focused for one hour. There's going to be that five minute, you just drift off, you get distracted, you talk to your friend, something like that.

But if it's a pre-recorded lecture, and let's just say you do lose concentration for a bit and you realize that, all you have to do is just go back by a few seconds. You can understand again. But let's just say you're tired and it's 9:00 AM in the morning, and then your brain just kind of snoozes off, you can't really do much about it. You're just, what's done is done.

Lecturer A:

Thank you. Any other recommendations for improvements? And again, this is a bit more deep and just feedback a bit more than what you normally do in your [inaudible 01:31:13] as well. Any other thoughts? So you guys have given some really good points already, but anything else?

Student A:

I think the links need some improvements.

Lecturer A:

Okay, yeah. Yeah, we talked about that briefly also. So more clearer discovery materials and all that, yeah. Anything else?

Student C:

I think, maybe this may be in a long shot, but maybe we can implement hybrid learning instead. Whereby if we were to implement, ILs would be the pre-recorded, 15 every four. Four every 15, sorry. And then-

Lecturer A:

Yeah, four by 15.

Student C:

...We come face-to-face for workshops.

Lecturer A:

Yeah.

Student C:

Yeah.

Lecturer B :

Okay. How about CPLs? Would you like them to stay the same?

Student C:

I think CPL, I would prefer a live session and have a poll EV open prior to the CPL, so that whatever questions that we have, we can just post it immediately. So the CPL won't be... We will have extra questions, but there are already questions beforehand for lectures to clarify for us. Yeah.

Lecturer B :

I assume you mean live in place of than if you come to Monash.

Student C:

Live in place or live online would be fine for CPL, because it's just clarifying any doubts that we have. It's not really learning anything, it's just more clarification.

Lecturer A:

Okay. Thank you. I don't have any more questions. Lecturer B, have you got anything else that you want to ask?

Lecturer B :

No, I think we are fine.

Lecturer A:

While Lecturer B is just finalizing what he needs to do, do y'all have any questions for us about anything? I mean, not syllabus specific obviously, but any questions for us?

Student A:

No, I'm good.

Student B:

Yeah, I don't have any questions either.

Lecturer A:

Okay. So thank you very much for your time and your feedback. I really, really do appreciate your honest thoughts and your comments. As I think probably Lecturer B did mention earlier, each of you will be entitled a RM50 Coffee Bean gift card. If you are living close to the campus, or get chance to come back to campus, just let us know and just drop us an email and we can pass it go on to you. If not, we will hang on to it until your next physical activity on campus. Yeah. In the meantime, and this is just something more personal from me, is any of y'all staying from [Sarawak 00:22:06]? Oh, okay. Are you in Sarawak now?

Student A:

Yeah, I just got home yesterday morning.

Lecturer A:

Oh, wow, okay. Okay, thanks. I will keep that in mind. Just, again, this is in the future in the months to come, I may have some student projects in hospital Sarawak. And I wanted to just find out what's the best place to advertise it, to see whether those people were interested. And again, this is not assessment based or linked to your assignments or anything like that, but this basically will obviously be useful for extra curricular and all that. But unfortunately-

Student B:

You are looking for a hospital, is it?

Lecturer A:

Yeah, but it's only, unfortunately, just limited for Sarawak in specific. It could be only limited for people born in Sarawak, who are living in Sarawak, that's the limiting factor, unfortunately. But, okay, is that something that I can advertise on [Mufus 01:35:04], or?

Student B:

Yeah. Yeah, you can use Mufus to advertise.

Lecturer A:

Okay, but Student A, you're from Sarawak, is it? You live-

Student A:

Yeah, I'm from Kuching, so actually this is the biggest GH we have.

Lecturer A:

Yeah. Yeah, I know. Yeah. Okay, thank you for that. Lecturer B, anything else?

Lecturer B :

Okay, I think we're...

Lecturer A:

Okay.

Lecturer B :

More or less, we have what we need here.

Lecturer A:

Yeah. All right. Thank you very much for your time. If there are any questions, if there's no other questions or any things that you have to ask, or clarify with us... Yeah.

Student C:

Between the two of you, whose office... I know Lecturer A your office is in building four, Dr. Ron is your office in building two?

Lecturer B :

Yeah, building two.

Student C:

Okay, so I think Lecturer A I'll come this Thursday to get the...

PART 4 OF 4 ENDS [01:35:53]

Deidentified FG4Y4, Sept 2020

Lecturer A

Okay. So the focus group shouldn't go longer than 12 o'clock. We have just essentially, probably two sections. The first section we'll be focusing on this whole online learning experience. And I know semester one, you have had a very funny or truncated semester. And so your full online learning experience really more so have been felt in semester two. So I guess in your responses, you can be thinking about that. The second half of the focus group, we will then be focusing on the overall design of the program. So you just really want to keep that in mind first. We'll start off with our first part, so really just focusing on this whole aspect of online learning. Very easy. Just from frank thoughts, how has the online learning experience been for you so far?

Oh, so [Student A 00:01:04] you understand? You're nodding your head.

Student A:

For me it feels a little weird at first, because we were really used to seeing each other, especially during workshop. Like in workshop, you need teamwork and stuff. Yeah, so it's a little bit weird, but slowly we're getting used to it. And I feel each of us has more confidence in asking question in the online session, compared to the lecture. So I don't know if you have felt that, but most of our lecturers have a lot of questions to answer all of a sudden during online session. Yeah, for me, I feel like I still like the face-to-face session. I still feel the interaction in the class and the whole environment is still better in the lecture hall compared to online. That's all.

Lecturer A

Thanks. Somebody else?

Student B:

I can go. Yep, like what Student A has said, initially it was a little bit hard to adapt, but slowly after discussing with our [UC 00:02:23] and how they adapt to our needs. It's slowly getting better, but still I would still prefer the physical classes because, you know having workshops through online, it's totally different from face-to-face. And in workshop sessions, we can even go totally silent during the entire workshop session and just have everyone else doing our part. And that's all, not talking at all. We are fine with it. It's the interaction that matters. In real life workshops, that means in face-to-face sessions, we can get lecturer's attention immediately by just raising our hands.

But in breakout rooms, it's quite difficult I would say, because even if we call for help, the lecturer normally takes some time. And in workshop sessions, normally we have on four lectures maybe roaming around, but in virtual workshops we only got one at lecturer attending to four different groups. So yeah, it's quite of the... I know the lecturers are doing their best, but maybe this is the drawback off having virtual workshops. Yeah.

Student D:

I think I agree with [Student B 00:03:35], the most online learning sessions that I find trouble to focus is the workshop session, as we cannot interact with other groups that's what usually we do. We have some chit chat, we have some gathering of information, but apparently we are not allowed to do that because we are only in our breakout rooms, which we're only allowed to discuss among ourselves. And to add with what Student B has said is we can't get immediate response compared to face-to-face lecture because face-to-face, we can just shout out our answer, shout out our questions, and we can get a response immediately. Yeah, that's what I would like to add in.

Student E:

Wow. Okay. So as for me, actually I like the virtual way of learning probably because it gives a lot of flexibility, because I have a very busy schedule and it allows me to have more time to arrange my things properly. Can you hear me?

Lecturer A

Yeah, continue. Yeah.

Student E:

Yeah. So in fact, but I agree a lot with Student A about questions coming out from students. Because it's an online session, so you can just type out whatever you want and you get the answer and it's documented in a way. In lecture hall, I need to write down every single thing or record it in my phone, but now everything is recorded. And all the questions, sometimes the lecturers even go to the extent where they actually extract it from the chats, all the questions and put in answers for us. So it's really easy for me. So except for the on-campus experience, which honestly, myself, it's not a very big part of me because I've gone through campus life and I've gone through another two studies. So it doesn't really matter to me, but for the virtual learning, it makes a lot of difference actually, because of all the documentation, except for the early part whereby when they just switched to online, and there's no updated schedule in the Google calendar in my phone.

So I sort of missed out a lot of deadlines and missed classes and then [inaudible 00:06:00] to Zoom. That's a bit tedious, but now, I think we are getting a hanger of it. So that's my opinion.

Lecturer A

Yeah. All right. Thank you. So we really wanted also you to contrast this with your offline experiences. So what you've had in your years one to three. So I think a few already have alluded to that that number one, there seems to be a preference for some of you, for a face-to-face interaction over an online interaction. I was just wondering when we just start Zooming and contrasting with what you've experienced this year in an online setting with what you've had in years one to three in a physical setting or a face-to-face setting, was there anything else that you wanted to comment on that?

Student A:

I don't feel through what you're learning, that students are putting a lot effort to make sure that everything that they deliver really go through, because I guess everyone knows that there's a communication barrier. There are some things that cannot be achieved through virtual learning, but like [Student E 00:07:07] said, a lot of things are really properly documented for us to refer after that. Well, as compared to face-to-face learning, we probably will only get the lecture recording in the lecture hall, but not like documenting every questions and answer it through our model... Or probably post it through the model for us to refer after that. I'm not sure whether it's like because we are getting different lecturers this year or what? But this semester's lecturers are really putting in a lot, a lot, a lot, much more effort than we have seen compared to past three years, I would say. I'm not sure if it's affected by the virtual learning myself, but we really appreciate that.

Lecturer A

Thank you. Was there anything else? Yep.

Student B:

Yes. Okay. So for me, I really had a hard time looking at timetables and the Zoom links to go to classes everyday, because the Zoom links would be different for everyday. And I really think that it depends on the lecture's dedication in getting everything centralized in one Google sheet for us. And

unfortunately, some of the lectures this semester for certain units are not really, maybe not that tech savvy or what. We often have technical difficulties in accessing Zoom lectures and it can be quite demotivating if I have to be honest. We get our feedback telling him to put everything in that Google form, instead of sending us an email every single week and we need to go and search back. But yes, maybe it's some of the technical difficulties or maybe... It depends on lecturer. It's a case to case basis.

But I must agree that for 4042, the lecturer has put in tremendous effort to make sure that we are kept updated. And the other thing that I would like to feedback is about... In physical lecture classes, as well as workshops, we do not face this barrier of... That means if we have doubts, we can get them clarified right after the class, we can stay back. That means that we can ask lectures face-to-face and things will get sorted out within minutes. But in virtual classes, we need to type email. We need the post forum. I think it's an extra, additional step and effort that we need to take to get our clarifications clarified, I would say. It's one of the very small communication barrier, but I think that it's the limitations of virtual online classes.

Student D:

I would like to add on something. With regards to e-lecture, right? Actually it's easy for me to mix up the deadline for all the assessments and assignments, especially for those group work assignments, because when it's face-to-face, we can still remind each other, but in e-lecture, usually we tend to forget when is our submission date for our assignment and assessment.

Student B:

Because for the past three years, we have been getting access to [inaudible 00:10:36] recordings. And whenever it comes to lectures that has Australian content, we would actually refer to the [inaudible 00:10:44] recordings as the Australia side lecturers are more... They know more about the Australia content. But ever since we started the virtual learning, we no longer get access to the [inaudible 00:10:56] lectures. And I think this is one of the major drawbacks for e-learning classes.

Lecturer A

Okay. Thank you. The next question that we had was really [inaudible 00:00:11:10], which again, some of you have already alluded to. We now really wanted you to just consider your ILs, your [CTLs 00:00:11:19], and your workshops, contrast what you've had specifically this year already, and to compare them with your physical experiences. So I think some of you have commented. Under workshops, you talked about how you felt that you feel like you're talking to a blank screen sometimes in your breakout rooms. Whereas obviously talking with your tables in the room format, that's obviously a bit more ideal or more preferred, but was there anything else? I really want you all to silo them in your contrasting ILs, CTLs, workshops comparing what you've had this year already. And contrast that with what you've experienced in years past. So your physical experiences.

Student D:

I think for the IL and CTL can be conducted online. There's no problem for that. And it's easier for us to follow as everything is in black and white, especially those question and answers, or we can catch up later if we are unable to follow during the class. That's the good thing about IL and CTL being conducted online, but for the workshop, I do prefer that if we really can have some face-to-face time, it's something that we need to create the bonding. This year, I think I didn't meet with my friends. Yeah, there's no more bonding between us, even with the lecturers. Yeah.

Student A:

Yeah. I strongly agree with [Student D 00:12:56]. I really like how everything was really documented for IL and CTL through this virtual classroom. A lot of things need to be clearly communicated among the teammates or even across groups, and also talking to lecturers, because I think it's also related to the way our workshop is conducted this semester. Previously, probably in a workshop, we solve a case and that's it, we call the workshop a day, but then now we have a few things to do like a series of workshop, which are also quite related but in a different way. So probably that's also contributed to some of the communication barrier because like the case-based discussion, the lecturer can only focus in one group at a time. So probably that also have contributed to some of the communication challenges we face during the breakout room.

But another thing I feel is that if we had a workshop face-to-face, it's easier to talk to the group members. At least if they don't talk to you, you can look at them and force them to talk to you. But then through the virtual classroom, I'm just talking to a blank screen. No one's opening their web cams, and then you could go like, "Hi, can I just clarify this point or is everyone okay with adding this [inaudible 00:14:29] to the version?" Then everyone just goes on and it's like now they go, "Hello. Hello. Hello." About three, four times to get someone to attend to me. But a good thing about virtual classroom is that anything that you want to show, anything that you want to share, instead of communicating it verbally, you can just share a screen and tell them, "Look at this. Everyone follow this." Something like that.

So pros and cons, but I still feel the bond, the communication and the team work would have been delivered better if it's in face-to-face. I still miss the PTS classroom, where everyone can just go everywhere and talk to everyone. Yeah. So that's my experience.

Student E:

Okay. Previously in the workshop, there are certain questions that I may not ask, or some of my classmates who have asked the lecturer personally may not have been shared. But I feel in a way, when there's a virtual learning, the lecturers are trying their best to share almost everything that other students are asking them, they will try to share out to everybody. So I feel like I learn more in a way, because sometimes I may not know what I don't know, so I didn't ask them questions, but when my classmates ask those questions, I sort of learn faster in that way. That's my personal view. As for my communication, I guess, maybe previously in all my groups, I don't really talk to everyone. So to me, it's like I go into the worksheet... Even when there is physical face-to-face opportunity. I see just everybody going to the worksheet and work on the Google sheet because we have limited time.

So I'm not sure, but I feel okay. And if I need to talk in the group chat, we still manage it through WhatsApp. Like what Student A mentioned, you can just knock and say hello then my group mates will still answer me. So actually I don't feel the gap for myself, probably because all this while in corporate, we are also very used to e-meetings and teleconferencing. So it's quite natural. And I feel that by doing that, [inaudible 00:16:52] their work on Google sheets and, of course, [inaudible 00:16:56] you have a very good team members who are productive and constantly working on things, it seems like we can get things done faster that way. That's my personal opinion.

Lecturer A

Yeah, sure. Thank you. Thanks. Student B anything else or?

Student B:

I agree what Student A and Student D said, because my virtual experience for this semester has been quite extremely different from what I've been experiencing in the past three years, I will be talking to blank screen, web cam off, mic also off. I asked them a question then only five seconds later, someone on their mic and get their answer to me. And then that's all, end of conversation. I think that we can really bond a lot more better in real life classes because everything will be much easier. I can just show my [inaudible 00:17:49] and say, "Hey, see this. I don't think we should do this." And

we get an instant answer or instant reply. So in my opinion, I really think workshop really makes a difference if we compare the real life one and the virtual one. Whereas for interactive lectures and CPLs, I think that there are pros and cons. The pros of the physical classes is that we get ask our friends outside, "Hey, what did the lecturers just say?" If we miss out some of the points and we can get that clarified right on the spot, right after classes.

But the pros of having virtual classes, like what Student E and Student A have said is that, people tend to ask more questions. We do not need to take photos. We are so close to the screen that we do not even zoom. Yeah, these are the different pros and cons that online classes and physical classes have.

Student A:

I also have a point to add is that, through virtual workshop, I do not know what challenges or what difficulties my teammates are facing. Usually in the workshop, in the real life setting, I can see their faces as they're frowning, or they don't understand what you said. They frown at you, they go like... They look puzzled, but in virtual workshop, I really don't know whether they understand me or whether they need help or they just didn't tell me, sort of stuff like, yeah. So this is one of my challenges as well.

Lecturer A

Thank you. I think those are some good points. Just to summarize and maybe just to get some more thoughts. The nonverbal cues that you're not able to see through your computer screen, I'm gathering from some of you that that is obviously very important in your communication with your group mates. And a few of you have talked about this team of this sense of bonding that you develop while working on your workshops. I feel there are some very different feelings towards it. If I can give you a scale of zero to 10, with 10 being very important and zero, not important at all. Can you just say that how important is this bonding to you that you need to have this bonding as part of your learning? And again, this is a personal preference. So zero, 'not very important, I'm still able to learn," but 10 being "very, very important. It's essential to help me in my learning during my workshops."

Student B:

I'll rate it at seven to eight. Yes. Seven. Okay, seven.

Student D:

I will rate it at eight, I think. Yeah. Yes.

Student E:

I'll rate it at 11 if I am allowed to, but probably because it's not about learning for that workshop solely. It's about getting to know more friends and when you have difficulties in the future, you know who are the people that you can look for. It's [inaudible 00:20:54] studying together and have fun together. It's all about the on-campus experience [inaudible 00:20:59].

Student A:

It would be like a six for me.

Lecturer A

Okay. No problem, thank you. And again, it's personal preference. We're all different. We have different styles of... Different personalities as well. So we just wanted to find out and see where that sits in the bigger picture. A few of you also commented on that you felt that there was some improved documentation or organization of the program compared to previous years. So now I'm

going to go down deeper into that. So looking at semester one and semester two first. This year, what do you feel about the organization? So when I say organization, I'm meaning timetabling, communication with academics. Okay, I know semester one you didn't have classes per se. So timetable may not have been really a big of a thing, but there are still like in terms of rotations or when one group does what and all that.

We just lost Student A for a while, I don't know where she went but it's the okay. Hopefully she comes back. But I guess the three of you all, what do y'all feel about the organization? So contrasting semester one and semester two first, with this year.

Student B:

Contrasting semester one and semester two to what?

Lecturer A

Yeah, or comparing. If they're the same, that's fine as well. Yeah.

Student B:

Semester one, it's very free. I mean, we are so free and so relaxed that we got nothing to do but semester two is so hectic and so busy. And I do not know why [Monash 00:22:39] arranged it like that. It's like we could have some of the workload in semester two back to semester one to even out and balance out everything so that we do not cramp everything in semester two. In terms of timetabling and organizing, I can't contrast much because we practically did not have classes last time in semester one. We got classes in semester two, but then there's a very big difference that I realized in terms of communication is that, I really applaud the effort of lecturers who compiled all the questions that they have received for that particular topic over the week and send us email or post us forums so that we can learn more. Yeah, that's really one of the very great effort that I think that should be continued.

Student D:

I think same comment with Student B, that since semester one we are really having nothing to do because due to the pandemic, our steps has been has been canceled. So we just basically do the cases; one community case, and one hospital case in our own pace. And semester two is quite busy. Maybe the school can think of another way to carry out semester two so that because we are having [OSCE 00:00:23:55], we're having so many assignments [inaudible 00:23:58], maybe it can assign it a bit in the semester one for the future students so that they won't have to face so many assignments in the final year. I know that final year, everyone says that it should be a busiest year, but it can Student De. I think it is something that we can Student De and we can do something to release our stress as we are currently in a very hardship. And we know that every day sitting at home facing computers, doing assignments can become depressed to someone. Yeah, that's my input.

Student E:

I truly agree with the two of them, Student B and Student D, because I don't know why there's like a multiple health promotion activities and proposal and video pitching, things like this, I feel it's very assignment based. Probably they can assign earlier in semester one because in semester one, anyway you'll be in steps. And then there's more flexibility because there's no exams. So we can really focus in doing that type of assignments format, whereas for second semester as we are all getting very anxious about the exit OSCE and now, it's an integrated learning, which I think is really important for us graduating students. We should have get more time for self-learn and revision and to brush up our OSCE practice for the exams and also for us not going to graduate.

So it's time for us to go back and read what we have missed for the past few years that we've learned and then consolidate it in our mind and prepare for the OSCE more constructively. Personally, we have very little time to do preparation. Most of the time we are chasing after deadlines, after assessments, so I totally agree if they could just make a shift on the format of assignment. Since this semester, we have an integrated approach. So we might as well make full use of the integration of what we have learned rather than keep on flooding us with assignments.

Student A:

I have a rather different opinion though. I feel that the assignments are okay. It's just that it's kind of repetitive. We have two different health promotion, we have two different practice innovation. As in like, they are the same. It's just that one is group, one is individual. And for health promotion, they are the same, it's just that one is focused more on indigenous population and another one is just a general topic on a specific health topic. So I guess if these assignments are integrated, as in you'll need to do one. Because they are the same nature, so I guess it would be better because I just feel that I'm repeating the same thing. The same thought for the same assignment. For me, about deadlines and stuff, I feel that it's manageable for me. I think it's still okay if we will...

For me personally, if I were to put a lot of time on OSCE, it will actually stress me out more. I feel that if I put so much effort, I must perform well during the OSCE. And if I didn't, then it's more of like me putting a lot of stress on myself if I really focus on that so much, and I put such a high priority. Of course, it's not that we shouldn't put that high priority, but I feel like if I put all of my effort and all of my attention on it, I got no time for myself to get away, to look at some other things.

So in this semester we had our project, we had our professional practice and stuff. So we get some time to get distracted from the very heavy or very stressful OSCE. Yeah. And as for, 5252, there are a lot of deadlines that every week we have something to submit; alignment tables, stuff like that. But I think if everything was done properly in 5151 stage, which we have all the time to during semester one. So in 5252, all your need to do was to just submit the alignment table and prepare slides to submit every week and present every week. For my group, we did that so we fill everything is still manageable, especially for 5252.

Student B:

Thank you for commenting on 5252. If I were to contrast, because for semester one, we are just told to do our research project, collect the results and describe our data. That's all. They said that they will leave the analysis part for semester two, but then semester two, we got no time at all to do our analysis because we are bombarded with a lot of alignment tables, discussions every single week until we do not have the time because we need prepare our posters or representation, and yeah. And these were really needed to be done in semester one. I think it's better to ask the students to do in semester one, instead of telling us to postpone it to semester two, but at the same time, give us a lot of additional assignment during semester two. It's already quite a hectic semester and we really hope that it could be balanced out between these two semesters.

Student A:

I'm not sure what was communicated by [inaudible 00:30:02], but our group came to a consensus that we finish everything, including the analysis. We know that we're supposed to do it in semester two, but everyone was kind of worried that semester two is going to be very hectic. So we came to this consensus that since we have all the time during semester one, we might as well just do it first. We actually almost have everything done in semester one. So in semester two, 5252 is just like a small little... It's very annoying a lot because everybody has to do alignment table and submit, but it's just that you can spend like five minutes to do it and complete it and that's all for 5252, so you have all your time to focus on 4042 and also 4012.

Student B:

I agree with Student A that we have a lot of time, but we did not make the decision to complete everything in semester one, because we are unsure of the requirements that will only be released in semester two. So we decided to wait and see if... We are scared of doing anything wrong but apparently that was quite a terrible decision.

Student A:

One thing was, we were told that 5252 we will learn analysis, which was why I think Student B, they all wanted to wait and see, but in the end we didn't know we were just doing alignment table the whole semester, so we got [inaudible 00:31:23].

Lecturer A

Okay.

Student D:

I would also like to add in something. Because they are mentioning about the course, the units structure. Then I would like to add in my input regarding the assessment details. For this semester, except for 5252, I think all the other units, their assessment details are being released very early compared to previous year one, two, three. I can accept it if these things happened in the previous year, but not in the final semester. As we keep on waiting for the assessment details to be released, for example, actually up until now-

PART 1 OF 4 ENDS [00:32:04]

Speaker 2:

... assessment details to be released. For example, actually up until now, for 4012, we still don't have the final exam assessment details. Whether it is a MCQ, subjective or anything, we don't have the information, so we don't know how well should we prepare for this final assessment. For 4042, although our UC is really, really good at comforting us, each week she tells us that, "Don't worry, everything will be fine," but we still feel anxious and anxiety because up until now the sub-note, the details that they want, the answers, the [inaudible 00:32:41] that they are waiting for to see from us. The OSCE cases, the OSCE details actually still haven't been released to us. And we are just less than one month from our OSCE. And I think you can imagine our anxious, if the information up until now is still not being released.

Yeah, that's the thing that I find most frustrating for this semester as all the assessment details is really, really, release very really late. Some assessment details actually I expect them to release earlier, for example, the video pitch, at least we can do that beforehand, but they only released that I think week four or week five. Yeah.

Lecturer A:

Okay. So, thanks. Thanks for your comments so far. Yes?

Student C:

Lecturer A, do you have a question later asking us about the content structure, the unit structure of 5252 for semester one and semester two? Or should I give my input now?

Lecturer A:

Yeah, you can give your input now. There is a similar question, but yeah, feel free to talk.

Student C:

Okay. [Crosstalk 00:33:52].

Lecturer A:

Actually, maybe no, I Student Ded my mind. Sorry. Maybe keep it for later because what you say made an influence what the other three are thinking as well. So yeah, there's a similar question. It's not exactly like that, but then if I don't bring it up, then at the end of it, you can let me voice or share that. So I know some of you have been going with quoting some really specific examples, but now I want you just to take a step back and just take a more broader view of the whole of what you've experienced so far. I think if I just to summarize some themes, some of you felt that the documentation and the organization of the unit or some of your units that you're going through right now is a bit better than what you've had experienced in the first three years. So, that's one.

But then again, some of your had some different opinions, where you not breaking it down to your assessments, where maybe the organization or the release of information of that has not been really in at a time that you prefer. So that is maybe a slight drawback or a negative for that. I wanted to ask now, what were your initial concerns when you started this online learning, and if those concerns are still there today? Some of you have said some things already about it, about this feeling of anxiety or unsure, but we're just wondering if there's anything else. So really, what were the initial concerns? And then if those concerns are still there today? So you've obviously gone through quite a few weeks already of online learning. Yeah.

Student A:

My main concern was the communication barrier, which is still there, getting better, but still there. Yeah. The rest of the thing was I thought, "Oh, it's a bit hard to organize or get everyone to respond, especially during IL and CTL." But actually, it turns out completely different. Actually, everyone respond much, much, much better compared to physical classes from the previous year. And like [Vincent 00:36:21] say, you learn a lot when your peers asked a lot of question that you never thought well. Yeah. So this is the perks that I found that I didn't expect to find in virtual classes.

Lecturer A:

So this whole part about your friends asking question and you learning from your friends question, was this non-existent in the previous years?

Student A:

I can say so. One is not a lot of people ask question in IL, in the physical classroom if you realize. For me, I don't know. Probably they are shy or what, I'm not sure. They refer to ask individually. They approached the lecturer individually. No one will know what they have asked, or probably I could have the same question, and then an answer... Or I never thought of that question, and someone did not raise that out in the lecture hall and no one knows... The question goes unknown, unless someone posted on the forum. Yeah.

And I don't think documentation, like the lecturer extracting every single question and answer in written form will be done in physical classes, because it's just a few seconds time that you can just answer the question on the spot, right? So I guess this is the good thing about virtual learning. And because everyone types the question in the chat box, so you can see that question, even though you didn't catch that random person asking about it. Yeah. So, that's the perks that I find. Yeah. Concern is communication barrier and still there. Organization-wise is still okay. I think it depends on individuals.

Speaker 2:

I agree with Student A. Sorry, [inaudible 00:38:13]. I agree with Student A. The communication barrier is one of my top concerns before the commencement of the semester and still there as what we had discussed previously. For example, in the workshop, we can get a direct feedback. The communication barrier is still there, and I think we're already desensitized to it and we're already used to it like, "Nevermind. Okay."

Another things that I would like to add on on top of Student A is my constant and other concern is about the technical issue. Because as we all know, every time we conducted e-exam, even though in school in our e-learning campus or some other way with new fancy e-exam labs, we still can have issues. But frankly speaking, for this semester, I think the technical issue is a bit lesser. Usually, when we are conducting a face-to-face ILs or closing the loop when we are Student Ding our locations due to unexpected circumstances, usually the lectures would take a lot of time to deal with the technical issue in the classroom itself. But I think for e-learning, we don't have that kind of issue. At least I don't see that they take 10 to 15 minutes to just open the computer, and connect to the USB. Yeah.

Student C:

[Inaudible 00:39:31] I still got extra. I still have e-assessment to go.

Lecturer A:

Thank you. Thanks there, [Vincent 00:39:42]. Yeah.

Speaker 3:

Yeah. So I second to the suggestions and feedback here by Student A and Student D. I think, yes, because when we were in a physical class, we shy away from asking questions because we are so worried that either we're taking too long asking certain questions that everyone already know. So I save it after class and ask, because I thought these are really simple questions that only I don't know, but everyone else knows, so why not take it out of the class? Then it will not drag the class, especially when we are chasing out the time. And sometimes we need to leave the lecture hall on time for other class would comes in. But in a virtual learning, it's like you can actually type faster than you speak in a way, and then it process your mind. You can put in your questions exactly what you're thinking. You can delete it and ask it, delete it and ask it until your satisfied, then you post it so that you get a very good clarification.

But in the lecture itself, sometimes when you are standing up and talking, then you might go say something different or try to explain yourself or your questions again and again so the lecturer get it, so you waste a lot of time. But now by typing, you can be sure of the questions that you want to ask, because you can read it a few times before you post it. Yeah. And I feel that some of our classmates are also very helpful. Before the lecturers actually reply, they will also give in their opinions and replying to us, which is also really helpful. And you'll see in a lecture hall, if you're asking, if sometimes I will ask my peers and it's like making a lot of noise. If everybody's starting asking people next to them, then it becomes so noisy in the lecture. But now you can still ask without making noise and get your answers while your still listening to the lecture, which I find it a very good advantage actually, because it looks like you can now [inaudible 00:41:29] effectively in a way.

So yeah, listening to your lecture and just using the questions, and it keep on provoking not thoughts as you learn along, "Oh yeah. I know I start to say thing that... Oh, this is the things that I never taught before. My peers put it up in the question." So, it helps. So that is one of the very big advantage for myself. Secondly, we don't need to travel all the way to the lecture hall for two hours lecture and break and then lecture. It could be the four hours in that campus, and then we have to spend a lot of time traveling and jam and time spent traveling. I think it's not that productive for me, but now I could actually, in between breaks, I can do other things and I can always schedule my own

time too. If I miss anything, I just play back later to check. And everything can be screenshot perfectly clearly. So I think that it's the advantage.

But of course in terms of communication, there was some barrier because you don't meet people in the class. So then discussion have to be done on emails or WhatsApp, so you cannot read the person emotion. Like what Student A say maybe, he or she is reluctant to say something, or she, herself, are not clear, but she can't actually show you her emotion, so you won't be able to gauge. And then there's a lot also miscommunication sometimes. Yeah. But on the management of the organization of uni, I think, yeah, like what they say is very individual. Some who can work very fast and very good in managing their time, they won't see a issue, but those who need a lot of teamwork and people and peers to encourage and motivate them to get things done, then there were issue some. Yeah, just my feedback.

Student C:

One of the major concerns before I start this semester is that I know that this is a brand new unit developed by Monash. And unfortunately, we are still going to do it via online. So it's like they are already many uncertainties, plus that we are going to do it online. I was actually extremely worried about communication barriers, and what they have previously mentioned, the organization, the structure of the content and how fast we can get ourselves updated regarding all the assessment details because that's what concerns us a lot too. But I think as times go through, the concerns are less. I mean, they diminish over time because the lecturers have put in a lot of efforts. And I totally agree with what Student A and [Vincent 00:44:02] has said about the pros and cons of what you're learning, but maybe I can add on to the communication with my peers, with peer learning and stuff like that.

Because in physical classes, one of my major concerns is that I would actually miss class. Before this whole virtual learning stuff, I was so worried that, "Will I miss any class?" And apparently, until this point of semester, I have missed two times of class because of distraction just in time table. And fortunately, my friend managed to identify me. In physical class, it's very obvious to tell that, "Hey, someone is not here because she is not physically here, so I can just... Or we call her." But in online classes, no one will realize you're not there because no one is screw the participant list to identify, "Hey, is Student C here today?" So it's not on the pure learning part that is extremely different compared to physical classes [inaudible 00:44:55].

Lecturer A:

Okay. Thank you for that. I want to just asks a bit as well, some of you said that you like the... That being in an online setting, you seem to be able to ask your questions a bit better and get responses from academics and from your friends. I guess comparing with your first three years, the usage of the forum or the online forum that we have on your Moodle page, I think the spirit behind that setup was there to help, to give you all a platform to ask questions on there where you don't have the opportunities in a face-to-face interaction, but the reality is that sometimes it's not actually really utilized by students. I'm just wondering why? What do you think? Whereas now suddenly, we have [inaudible 00:45:49] even still. Because if the tool and the platform is different, like now you're asking questions from full air, for Zoom chats and all that, but then the context is still the same as via computer screen. Why is there a difference?

Student A:

One is shy-

Speaker 2:

I think [crosstalk 00:46:06]. Personally, I think it's very easy if you want to find out the reason. You just go to the previous... I mean, the platform in which we ask questions and you see whether there's

reply or not, then you will know that why previously no... I mean, we are lacking the enthusiasm in asking questions in that platform because basically no reply. But when you see, come to this semester, there are a lot of replies, especially from the lecturer itself. So it's a two-way thing. When we get a reply, we will get what we waited and ask more. When you get, if you get, you ask more. But when there's no reply like that over there, then no one would go [inaudible 00:46:50] and ask some questions.

Lecturer A:

Okay.

Student A:

One thing is shy. Initially, I think it's shy because if your name is there, and then you type a question and stuff. Another thing is no reply. Initially, really year one, year two, not really a lot of reply. I don't think I get more than five replies. Okay. I didn't post more than five times. Okay. I post three times, I get two replies. Yeah. But whereas for this year, this semester particularly really a lot of question, a lot of replies. So I guess I agree with Student D, it's a two-way thing.

Lecturer A:

All right. Student C, [crosstalk 00:47:37].

Student C:

Actually, there are two major reasons. The first reason was being that in physical classes, we have other methods of asking questions and get my replies instantly. Why would I want to post on forum if I could get a reply within seconds? So, that is a easier result. So I will, of course, use the easy option like this to get things done. The other thing is whereas in this semester, you have no choice. You do not get to see the lecturers, so you can only... Forum, it's one of your very limited choices to ask questions. The second reason was being that initially, there really weren't any replies. It's not redundant post. We tried posting one or two. But in the end, if there's no reply, we get demotivated very easily and say that, "It's just easier asking face-to-face and get my helps clarify that."

Whereas in this semester, everyone gets... I mean, once the lecturer start to reply, we get motivated to post more questions to get more replies now. Yeah. And [Poll EVs 00:16:39], why? I'm also not sure. Oh yes, I know already. Because last time in physical classes, [Poll EVs 00:16:50] are posted on the spot, and then we are told to type our questions on the spot. But then for this semester, they opened the [Poll EV 00:00:48:54] link a few days ago. So that means that we got more time to think of questions and we can type in questions at our own pace. And then the lecturers would compile everything and answer them in essentially in one [inaudible 00:49:09] and get them back to us. So I think it's easier. Whereas, we can't really think of questions on the spot within that one or two minute in physical classes, I think. Yeah.

Speaker 3:

Okay. So I think there's three things that I can define different why. How could they be different when there's a same platform and same tools? Firstly, when you do it on the Zoom chat, you get instant reply. So the lecturer will give you instant reply either in written form or in oral form. And if you are not sure on the spot, you can ask further question to clarify. But if you go on forum, you have to type in a good words of it, and then you wait. And then sometimes actually we say, "Let's wait for other peers to reply you first," then you wait. And then by the time the lecturer actually answered the question, I'd be lost already. It would be like, "When did I ask the question? I need to go back to the forum." But now, on Zoom is right there instantly you can get answers. And if you don't get it, you can unmute yourself and ask further.

So I think the clarification is very instant. Actually, it's very motivating for everyone to start asking. And despite feeling that it is just online so the interaction will be less, in fact, I feel the communication is better because more and more people is actually participating in the question and answers and you have lecturers. And maybe because of the nature of the class delivery virtually, the lecturers feel like they have more responsibility and care to make sure the students really understand what they are talking about. So they tend to be more repetitive, more comprehensive in covering their lectures. So I find that is a different... In previous lecture in the class, maybe they're just like... You don't say it and then... But the effort of this virtual learning is they really keep on trying to make sure, "Are you okay? You really understand or not? Or do you need more supplementary information?" to guide us. So that's the second one.

And the third one is definitely the engagement, because you know that the best time to ask is during the lecture. Otherwise, it will be totally on emails or text messaging and so on. So you will be more willing to say it. And sometimes you won't feel that... Like I mentioned, you will always check your question before you post. That give a lot of security, rather than when you stood up in the class and say, "I have a question." Then you may not be able to explain yourself better, then you feel intimidated and embarrassed, so you may not want. But now, I feel that the question that's coming in... In the summary, basic questions, everybody is very willing and open to asking it. So I feel that it's a very good move actually. Yeah. Because in all my studies before, I see participation of question and answer in classroom is very little and limited. But in this virtual classroom, I find that the number of questions that's going in is really amazing, a lot of it.

Lecturer A:

Thank you. Thanks for that, for the comments. The next question, so you've had some introduction and exposure to research over the past two years. And this is, again, related to this online learning. I just wanted to ask you to what you have gone through so far and what you know, do you think research can be taught online?

Student A:

No.

Lecturer A:

Okay. So no from Student A, no from Elle. Why? Okay. So most of you say no. So, why?

Student A:

Okay. I think a lot of things are not really self-explanatory based on the statement itself, especially... Can I go back to a 5052? That's actually ethics and statistics. I think we cannot stress enough that based on reading the statement, everyone will have interpreted it differently, but then we will have a same assessment, same MCQ question. And the answers, the choices of answer are really subjective. So I guess probably if it's taught online, solely online, like how we had gone through it in 5052, everyone perceived that thing differently. So when we go to the quiz and stuff, everyone would think that, "I feel from my understanding A is correct." But another person would say, "No, I think C is correct, but actual answer is B." So everything is not really standardized.

I actually feel very lost. I feel like I do not know what I actually should understand. Should I understand it as X plus Y equals to Z, or it actually is X, Y equals to Z? Yeah. So some other things like methods and stuff, you can read it and you can understand it, like take it from the face value. You can understand it if they're really... But what it means. But for things like ethics and especially statistics, I don't think online or pure on Moodle is a good option. But if you say-

Lecturer A:

This is not a good option.

Student A:

Pure only on Moodle. We only have it on Moodle.

Lecturer A:

I see. I see.

Student A:

Yeah, we did not have any classroom. We did not have any classroom. Yeah. So that will not be a good option, because I think per user... They use per user, right? Yeah. So yes, we can ask question, but the major drawback is the lecturer has to keep checking. And if the lecture needs them, and then we also have to go back, keep checking. A lot of things maybe it really needs some verbal explanation. So I think no. Even for 5252 as well, a lot of things... Let's say alignment table, we do it, but we do it from what we understand. But actually, there are a lot of stuff that was being asked or clarified during the workshop. Not really doing the lecture, but probably doing during the workshop. Yeah. So I think if it's purely just on Moodle and then just me reading the thing, then probably I don't think I will understand as well as I have verbal explanation. Yeah. That's all for me.

Lecturer A:

Thank you. If anybody else has anything different to add, then feel free to share, but if it's similar to Student A, then we can proceed. Anybody else has anything different or extra, or disagree or not? So mostly agree with what Student A has shared? Okay. Student C, you had something to say about your 5252 and 5151?

Student C:

Yes. So this our first semester research, we were told to finish up our report until results. We even wrote something script or something, a paragraph to describe our results. That means the entire semester we have been doing that report, right? That part really. But suddenly in the second semester, we started from introduction again and we were told to review everything from our study research in whether your objective, is it aligned to your aim and does it address any gap? But the problem is we have already done everything in semester one. And now in semester two, you suddenly want us to review everything that we have done in semester one. It's like, even though it's... Introduction to me, it's like half a year ago thing, and now suddenly, do you want me to go back to see if my introduction is right or wrong?

And I thought it's a little bit not effective in terms of time management, because we could have totally done since from the start. You tell me how to do my introduction. So I actually draft my introduction instead of getting me to finish everything already. And in the end, you tell me, "Actually, we need to start from step one. Let's look from introduction again." And leaves us no time for analysis. I think that's something that might need further tuning in terms of the structure of this whole research thing.

Lecturer A:

Yep. Yes, Student A?

Student A:

Actually, I have a completely different opinion though. I do think doing the alignment table really makes you think what you wrote before that, is it actually appropriate now? So because at first before I do the research, my understanding of the introduction should be like that. But after I've

conducted that research and you now think, "How should I align my introduction to get the audience to understand why I need to do the research?" And then link it to the methods, link it to the result. So actually, it may quite a bit of revision of the intro, of the whole report from what we have been taught in the alignment table. And also, the requirement put forward for us to do the poster and also the presentation. It really gives me a Student Dce to rethink the whole project and restructure to how to make it coherent and how to make the whole flow better when you want to convey it to a audience, be it through poster, presentation, or the manuscript itself. So I guess there is still value to what the structure, to how it is constructed.

Student C:

I agree that it's a good thing for you to retain on whatever you have done, is it better or not? But I also do think that it will be better if we were taught on the whole thing before we even start our research project. We have that clear idea in mind saying that, "Actually, the research project needs to be aligned throughout your research. You need to have that." I think it's better if we were taught of this concept before we even start writing, instead of after, like three months after, we finished writing, only you start to tell us that, "Okay. Now, maybe whatever you have done is wrong. We need to review everything again." Yeah, I think it would be better if the sequence is the [inaudible 00:59:54].

Lecturer A:

So point taken. So I think the comments now are getting a bit more specific to the units and I feel... I think in your set too, and I think maybe Dr. Chong has also shared a little survey with you guys, particularly just focusing on those units. So that's a good Student Dnel to also further expand on that. But definitely now points taken, and thank you for your sharing. Was there anything else to add, Student A? Yeah.

Student A:

Yeah. Probably, I think every supervisor propose a project before very clear in, but then-

Lecturer A:

So that's a suggestion? Yeah. Is that-

Student A:

I mean, I think every supervisor propose their project with a right [inaudible 01:00:43] already, right? Am I right?

Lecturer A:

Yes. Yes. Yes.

Student A:

Yeah. So then the thing is that whether this is communicated to the students, which also depends on whether the students have made the effort to see the supervisors of the project they are interested in before they actually go into the project. So I think maybe if everything was communicated, as in if everyone was very clear to what they have to do when before they choose the project. Because I think that I thought actually find this thing very funny that not everyone in the class actually see their supervisors before they choose the project. Some people honestly telling you they just use for fun. They say, "Oh, the title looks funny." Right? So then in the end, they go into the project not knowing what was actually required, and then they regret it. They think, "This not funny."

This is what I personally had encounter, which was why I think in 5052, before the students are released to choose their project in 5151, it's very important to stress that you need to see the supervisor if you really want the project. You need to know what I required in the project. You need to know what is the project about? What is the purpose of the project? Then you start the project with a very clear mindset, then everything should be okay. And as in you already know what you need to do. You know what you should do. So in 5151, you do according to that direction. Then in 5252, it's just for you put everything together and piece it nicely and present it to everyone. Yeah. So I guess when we were in 5252 and we were choosing projects for 5151, I guess I think that this part was not being highly stressed or done as I've got. It's mostly based on the student's effort, then yeah... So that's why this thing happened.

Lecturer A:

Yeah. Okay. Point taken. Thank you for those thoughts. My next and my last few questions on this whole online learning part now, how do you feel about the assessments that you've had so far in your online setting? So I think Student D did share a little bit earlier about that. You prefer information about the assessments much earlier, but was there anything else? I know you all have not the Student Dce to sit for a final paper in an online setting as you're juniors, but you've had some small assessments here and there in the online setting so far. Well, how do you all feel about it?

Student C:

I feel happy because it's all open book.

Lecturer A:

Okay.

Student C:

Just joking. Yeah. Yeah.

Lecturer A:

It's okay. It is a comment. Yeah. We've had students say that they like it because it's open books. We also had students who say they do not like, they hate it because it's open book. And so then we like to ask why as well, so-

PART 2 OF 4 ENDS [01:04:04]

Speaker 4:

And so then we'd like to ask why, as well so.

Student A:

I like it because it's so simple, because you really can't memorize everything in your head. So then, when we had closed book exam, right? Then the MCQ, where the answers very close to each other, you really have very hard time to sit there and think through about it. Whereas if it's open, or of course, the first time question, of if you know where your things are, right, you can actually flip very fast and get what you want, exactly. And more like when you are in doubt or you're not sure of your answer, you can quickly flip and be sure of the answer. So I like it. Yeah.

Speaker 5:

In addition to the point where assessment details are not uploaded quickly, there's one other concern about online e-exams. The small quiz that you'll be having this semester, it's the technical

issues. We will be in a panic if we experience technical issues because it's different from physical e-exams. In physical e-exams, you can just raise your hand and get that issue sorted out. But at our home, what else can we do?

But I think the Zoom meeting, it's quite a good idea. But again, it's about the technical issue that's not maybe only in Wharf, the Monash site, the Moodle site, it might sometimes be even the Unifi faults at our home. So it's a lot more stress that if technical issues will be experienced.

Speaker 4:

Okay. Maybe to help answer us a bit better now, do you feel that the assessments that you've had so far in an online environment is a true measurement or is a good measurement of your knowledge and your skills? Okay. So two nodding head, two saying no. Why?

Speaker 5:

I feel it's not really a true measurement because it gives a lot of opportunity to students conduct collusion, open books, and stuff like that. It does not have the stressful feeling of having physical e-exams, and it does not duplicate the physical e-exam when we do our e-exams at home now. Yeah I will say that. It's not exactly a true measurement as there are rooms for collusion.

Student A:

Collusion, I don't know. I didn't, so I'm not sure about this. But it's not a true measurement because one, it's open book where you can flip whenever you want, you can Google whatever you want. So then whether or not that knowledge is in your head is [inaudible 01:06:59]. If it's a [inaudible 01:07:00], it's a closed book then you have to stuff everything in your head already. And whether you can stuff it [inaudible 01:07:09] there and stuff. But online assessment is just a big one. Another big part of our assessment is mobile, the [Oskie 01:07:18] workshops, the case-based discussion, Q&A. Given the time restriction that actually truly sees how good you are, how much knowledge you have, how good you are. Because your friends cannot help you in that six minutes right, in [inaudible 00:03:44], so that really, really, really shows how good you are. You really have to perform. And that's an individual assessment. But you do not know if someone private message the person that... Yeah. So whether true measure or not? Yes and no I think, but mostly it's a no.

Speaker 4:

Okay. Anything else to add from the rest?

Speaker 6:

Okay. When I told my peers that we are having open books, so they thought that it's so easy to score, but actually it is not. Because if you don't have certain foundations or basic understanding of the topic, even your having an open book doesn't mean you can score because a lot of the questions are more applications and case-based where you try to Google also, maybe you won't get an answer. So my fear is that probably because in this era, we are always loaded with so much information. It's at your fingertips, basically. Everything is at fingertips you no longer need to memorize every single thing because you can just open your phone, your apps, just get the correct answer. I think that's also because the expectation of the people nowadays, a community that there's a bop of information that you will need.

And so it's more important to be able to learn to maneuver and pick up the information correctly and to apply it. I'm not sure about the others, but actually even if open book sometimes I have difficulty because if I don't totally understand certain concepts, [inaudible 01:09:23] it's applied appropriately, but it's also fair to say, like that [Addis 01:09:31] mentioned that you still get an advantage because certain [inaudible 01:09:37] every single questions are designed or that look in a

very critical way, that role require you to be applying from a multi-angle aspect, but there's the impression that you can really find an answer [inaudible 01:09:54] get it. So I will say, it's an advantage for students like me, so that you still managed to pass, and you [inaudible 01:10:03] go over the line with more effort, but you feel supported and confident.

So I'm not sure if it's a good thing, but information all around the world. I think it's okay. It is the way forward is [inaudible 01:10:18] that for ourself, you really need to refrain from colluding, I think colluding is not the right thing to do. But if we can maneuver through the open-book information and get a good result, I think it's okay for this generation of living because of the information that's abandoned. Because I remember those days when my friends were in pharmacy, they were telling me that they have to really memorize all the dosage and every single thing before they go to exams. But nowadays you don't need to do that because everything's in your phone, you should be able to judge and pick up information rather than memorizing it because things keep Student Ding, updates keep coming. So it is impossible for you to memorize everything.

Speaker 4:

Okay. Thank you for that. So we're going to... [crosstalk 01:11:05] Yes, please.

Speaker 5:

In terms of online exams, since it's normally it's perceived to be easier because we have abundance of knowledge online that we can [inaudible 01:11:18] but I think... I felt should be put in to make sure that the questions are challenging in state. For example, for calculations I remember I studied for a few days, but in the end when that's just give us questions from the check your learning question bank. And I felt that it's really not a true reflect of what I have really known or what has really studied throughout that few days. And I feel that it could be done better. Yeah. Instead of where you can just come up with questions that everyone can google and everyone can get answer, and everyone gets full marks. [inaudible 01:11:52] It's a little bit like in Chinese we call it [foreign language 01:11:56]. Yeah. I want... That's what I feel. It could be more challenging to be able to reflect our true knowledge and our capability in terms of school.

Speaker 4:

Okay. Thank you. Thanks for that. I think we will stop there for, in terms of our discussions on online learning. The next part, we hope to get some thoughts and opinions is really now in the overall design of your whole pharmacy program that you've had so far. So the reality is that, and I'm sure you realize as well that the program that you are going through, almost finishing is a bit unique to Monash, and the style we teach it as well. You have this whole aspect of being shared some discovery materials before going for class. And then you come for your class and all that, and then it ends with a closed loop. It is quite unique to how pharmacy is taught here and this formulation as well. So just starting off first what you think about discovering materials?

Student A:

Starting from year one, right?

Speaker 4:

We would like a more broader view. So yeah. You could do some contrast. [crosstalk 01:13:11] Yeah. As well. Yeah.

Student A:

I think initially with [inaudible 01:13:17]

Speaker 4:

[inaudible 01:13:18]

Student A:

None of us come from a background where we had to do active learning. So at first, we were just given a bunch of things that they say in day 2 orientation. I only found out about modal, and they told me, "You have two things, two refresher module that you need to do before you start the class next Monday." And I'm only like few days from my first day of class. I'm like, "Okay, that's how you a pharmacy school is like." So, okay. We went through the module, and I reread the first week discovery. We go to the lecture, the lecturer...

I don't know. I think that time was just the first time that... So the interactive lecture was not how it was like now. It was a somewhat repeat of the discovery. So then, so everything, then while we read the discovery unit, go through the workshop is a completely different thing. It's a whole new world. Suddenly we were given a case, sort of like a case, and then we had to solve it. Then we asked the lecturer, "How are we supposed to approach, how are we supposed to do that?" They say, "You have to do it by own, but I can't give you any answers." No answers given. No guidance given no. You jump into the pool [inaudible 01:14:40] You don't drown yourself. Now. You see, then there was no close the loop, during year one. So after workshop then, okay, now you forget about that week. You start the next week.

Then until year two, we had our first close the loop, but then it was very weird. It was designed as to present whatever we had done in the workshop, which we are already being debriefed in a workshop. So the whole course [inaudible 01:15:12] the thing that everyone will think for each other to present. No one wants to present. Lecturer get angry. And then everyone just sit there for two hours. Bye, bye. I guess after five weeks, they Student Ded the style of CTL to consolidate idea concepts, to clarify doubts, which is very helpful because in year one, after a workshop, maybe something we don't know, don't know, don't know [foreign language 01:15:42] okay [foreign language 00:11:42] bye bye [foreign language 00:11:42]. Go to the next semester, next topic already. Whereas when we have a close the loop, it really helps to properly close out the whole topic for that week. So I think the way it was the whole active learning thing, especially discovery was developed Student Des across the years.

So in year one, discovery just a bunch of things that for [inaudible 01:16:13], I really spent a long, long, long time reading it. And I do not see the application of it in my workshops for year one. Year two, because it's clinical base already. You really get the patient in front of you on the paper in front of you, and you need to get what you knew, whatever they have learned from your discovery or lectures in school. You have to apply it on a patient that then discovery becomes very important because if you don't do discovery properly, you go to IL. Your IL case study patient die. And then you go to workshop, your workshop case study patient also die. So yeah, discoveries become very... The first thing that you need to do properly from year two onwards.

That's for clinical-base for professional practice. To be honest, sometimes I read discoveries, I don't know why I read already. I go to interactive lectures. Well, I understand in discovery suddenly become completely different in the interactive lecture. So that's what I feel for professional practice. That's especially for year four one, but year one, year two, year three wise is still okay. Yeah.

Four five two five for research. Then of course, five zero five two, we only had the discovery itself. We had learn everything from that. Then everyone perceive it differently though. For five one five one five we've got no discovery.Five two five two discovery is mainly alignment table because they need to individualize... they need to tailor it to each project. For the three articles that were given in the discovery, to be honest, most of the time it's just a copy paste up, but yeah, probably not... We did not find anything useful from that, but it's very useful when it was tailored to our project. When we were filling in the alignment table for our own project. And that's where we really

see how we should align everything together. Yeah. That's what I feel for discoveries across all four years.

Speaker 4:

Yeah.

Speaker 5:

For me personally, [inaudible 00:14:38], just based on discovery materials. I'm someone that prefers discovery materials over slides because I feel discovery materials are better organized, and it's like a textbook to me. So, but sometimes I do feel that the discovery materials are sometimes too heavy with a lot of external links. And sometimes the funniest part is that they want us to go into the external link to read 20 pages of PDF, just to find an answer in maybe just one of the sentences. But I think this has been improved over the years after we have even our feedbacks that you have specifically for us, which section to read and which section to find answers from lab for this semester. I felt that it's quite okay, but there were some of the discovery materials in the past three years that I personally feel that I will need [inaudible 00:15:28].

I will need more than two hours to finish that. I don't think the time that they suggested for us to finish the activity is actually truly reflective of the time that we spent. There was one year, I forget what unit was that. I remember spending the whole day reading a discovery materials where it only states that I need two hours. And I think it could be better fine tuned, when you do not include so many external links. Instead, you can just extract that specific part where you want us to know into the discovery materials instead of us flipping through 20 PDF pages, just find one mini answers. And I think that the external links can sometimes be quite hectic. I understand that they want us to explore more on the information outside, but I think there should be a fine balance. As in you do not want the students to spend so much time reading some of the stuff that you don't even think that it's important.

But in general, I think that this discovery materials has improved over the years. I personally felt that this year one discovery materials was the most hectic one, because like what [Student A 01:20:32] mentioned, we couldn't link it to our hour or two hour workshops, probably because it was not clinical based. And it was a lot, a lot, a lot of information in the year one discovery materials. But for our comprehensive care and integrated care, it has been improved over time. And I really see the correlation between discovery IL and workshops, yeah.

Speaker 4:

If there's anything different to add from the other two, if not we will proceed. Yeah, we proceed. Okay. All right. So thank you. They just have similar thoughts as both have been shared already. Now the next thing is about [inaudible 00:17:17], what do you think about the in-class activities? What do you like about them, dislike about it? So the reality again, as well, that the lectures that you have here in Monash, they're a bit more interactive in nature. From your earlier comments, I get it that a lot of you does seem to like the interaction in your workshops, but what about the interaction that happens in a lecture format? Is there something that you all like or dislike about it and why? Anybody?

Student D:

So you mean the online or the face-to-face that we have previously conducted in our previous year?

Speaker 4:

So now I want you to take all four years. So don't think about online offline. The reality is the reality is that all your lectures, whether online offline will have some interaction in there.

Student D:

Yep. Yes. Yes.

Speaker 4:

Yeah. So, whereas some unis, their lectures, they don't have any interaction at all. It's just a one-sided communication in the lecture format. So yeah. So what do you all feel about it? You like it, you don't like it and why?

Student D:

I think for year one semester one, the word that I've heard the most from almost every lecturer is they're not supposed to spoonfeed us. That's the one that I received most of the time when I want to ask questions, especially in year one. But sometimes in my mind is "I agree with you. You are not allowed to spoonfeed me, but you have to ensure that my plate is off healthy foods that I can pick from. You cannot just put everything on my plates. And you say, you pick yourself, and I'm not allowed to spoonfeed you." I think that's the wrong concept of... I mean, I think somehow maybe their integration of spoonfeed is not what I mean. Yeah. It's not something that I expect. You can choose not to answer my questions, but at least give me where should I find the answer in case... I mean, instead of just giving me a word, they are not allowed to spoonfeed us.

That's a really, really tough time for me, especially in year one semester one, because basically frankly that's the answer that I get. So across the semester, then I received more and more constructive feedbacks from the lectures. Maybe they are also learning as what we did. Yeah. Because it's a new syllabus for everyone. So I think I can know... I mean, I get that feeling and maybe they're anxious as well. So then across the semester, plus the years we have gained something more deeper, especially in the disparity, as well as doing the interactive lecture, especially the closing the loop as well. Student A has mentioned previously, the closing the loop is just control C control V of the workshop. But now the closing the loop, I can know that I can expect that there's somethings important about the topics that I can get from closing the loop instead of just me discussing the workshop materials. Yeah. That's the improvement that I can see there. Of course it can be better.

Speaker 4:

Okay. Yes. Yes. [inaudible 01:24:33]

Speaker 5:

So the interactive way I have actually adapted to it. I feel it's quite a good thing, but like what Student D has mentioned. In my first one or two years, I always get, even though if I ask questions, the replies I get will often be, "I'm not allowed to tell you. Your discovery has this answer. Restructure discovery, or either please go get answers. I mean, basic answers on your own." It was quite demotivating for me because I came to the lecturers because I have tried looking for answers, and I couldn't get, but you're sending me back somewhere else and asked me to get answers from my own. And as someone who has went to [inaudible 01:25:16] for exStudent De for one semester, I can tell it's quite different. The active learning was quite different between both campuses.

And as in that view, it time management, everything was very accurate. It's like we wouldn't experience things like class being delayed for 20 minutes, but it's quite frequent in the Malaysia campus. And for lecturers, over there the lecturers will actually answer your questions instead of sending us back to no where and ask us to get our answers alone. Even if workshops where the tutor, although she asked me to find answers on my own, but five minutes later, she would come back to check on me and see if I had gotten my answers. Instead, in Malaysia, your lecturers ask you to get answers on your own, but in the end, he or she doesn't really care whether you have gotten your answers or not, unless you asked again. I think that's what makes a difference. But personally, I felt

the interactive way. It's okay because it makes sure that the students are on the same page as the lecturers, because you can tell by their expressions or by their answers better, have they already grasp the whole... the basics of what the students are supposed to [inaudible 01:26:25] the discovery materials. Yeah.

Speaker 4:

So yeah, Student A, yeah.

Student A:

I guess it still depends on individual lecturers. So [inaudible 01:26:41] are really interested. They really want you to get what they want to deliver. They really care for us. So I think it also boils down to when the syllabus just after developed, no one knows what they should do. No one knows how they should handle it. So the interaction in the interactive lecture initially was very quick. The lecturer ask the questions, quick silent. No one knows what they should answer. No one know how they should answer. No one knows how they should handle... How they should go to interactive lectures. Everyone's shy. Everybody's just keeping it to themselves.

Then throughout the years, more and more interactions are seen. Everyone becomes more and more active, more and more proactive, and really knows that we are the one who should take charge of our own learning. So if you don't ask, if you don't interact, then you [crosstalk 01:27:47] what you should know. Yeah. So then, [crosstalk 01:27:51] sorry.

Speaker 4:

Yeah. So y'all are giving very third party opinions. I want to know. Do you like it or not? Yes.

Student A:

I like it because without the interaction, there's no point of interactive activity. You just go down and sit and then...

Yeah, then you just answer a few activities, questions then bye bye 了. You conclude the day. You don't gain anything. So-

Speaker 4:

Sorry.

Student A:

[crosstalk 01:28:20] so with the interaction then you gain what you want or what the interactive lecture is supposed to deliver to you. So I think the interactions very important you really need that interaction to sustain, especially close to the loop because, without any questions asked, then close a loop has no point already.

Speaker 4:

The other three, do y'all like it? Or don't like it? Just like or don't like?

Speaker 5:

I didn't like the year ones and year twos one, but I liked year three and year fours.

Speaker 4:

Okay. [crosstalk 01:29:01].

Speaker 5:

Yeah. Because I felt that maybe as time passes, we are more... We are less shy in answering questions. We get feedbacks from the lecturers more. We get more answers. We are more motivated in learning. So I thought that if you asked me in year four, yes, I like interactive because I always get my answers, despite me not getting answers from somewhere else. Yes. I like it.

Student D:

I think my answer would be similar to [inaudible 01:29:25]. I disliked it in the previous year one, year two, but I kind of more like it in especially the final year.

Speaker 4:

[inaudible 01:29:36]

Speaker 6:

Yeah. I feel the same with [Student D 00:25:40]. I think the fourth year one was the... It's the best discovery material. And it's meaningful in the interactive lecture because even for the past three years, I don't think... I myself is open and active enough to ask questions, to call it an interactive lecture. And to some extent, some lecturers are being very mean or being angry just because people don't ask question. So I think if they motivate a lot of us at the very beginning because emotions were brought into the lecture hall. But as the contents mature, and we as a student get used to the delivery format, currently, I think I like it a lot more than the past three years. So I think from this point forward, I would like my discovery more, then I like the interactive lecture more.

Speaker 4:

So. Okay. So some of you commented that there was an initial dislike, and it Student Des over the year two, year three period. I want to know why. I guess the initial dislike would be because it's new. It could be a lot or yeah, you just get a bit... Or there's so many things that you've said already, but what triggered this Student De or why the sudden Student De?

Student A:

I think initially was... Oh, sorry [inaudible 01:31:10].

Speaker 6:

It's okay, go before me.

Student A:

Initially it was because you don't know what to expect because there's no experience of [inaudible 01:31:18] classroom. All the while or these year was just waiting for what the lecturer has to say. Suddenly they say you are in charge. You have to say something, then only I can tell you what I'm supposed to tell you. Then it was like, "What am I supposed to say? I do not know what I should say. I do not know whether I have understood the discovery correctly to... or Whether what I have now is up to the standard of what you want." So everyone was very puzzled. So we do not know how to initiate.

Then the interactive lectures evolves. It comes to have a lot of question and answers. So that it helps the interaction factor. It helps you to start thinking what you have learned in discovery. It's time to apply it now. And it's time to explore what you have not understood correctly, or why you have not understood adequately in the discovery through the question and answers in interactive lectures. So then when you start being able to ask questions, you start getting very constructive

feedback. You feel that you gained something. I feel that I gained something from the interactive lecture. So I start to like it more. I started to see the importance of interactive lecture. I start to see the application, the value of interactive lectures, and it helps me move forward to my workshop. It helps me to consolidate my knowledge and further apply it in my workshop.

So with that, a lot of things can be clarified in the interactive lecture. So after you have everything clear, then when you go to the workshop, you will perform... I personally performed much better. I know what I'm supposed to do in the workshop. I have my knowledge ready already after the interactive lecture, rather than in year one, year two. In year one, especially I go to discovery, and then the interactive lecture somewhat has nothing to do with the discovery, and then in the workshop it's another whole new world. So I cannot find the link. I don't know what I'm supposed to do every day. It's just very puzzled, yeah. That's why the transition is there.

Speaker 4:

Thank you [inaudible 01:33:47].

Speaker 5:

I'm not sure if what I'm going to say next is really the major factor. But I felt that in year one and year two, we got a lot of questions, such as "Discuss with your peers for 10 minutes, then we will come back and discuss." There were too many of those. I'm not sure if it plays a role in me not liking year one and two lectures because firstly sometimes I really sit on the first row without friends [inaudible 00:01:34:14]. And sometimes the question, I got no one to discuss to with. And the second thing is that some of the questions are really quite simple. It's that maybe there's some of the factors that you can find from the discovery, but you just give us 10 minutes.

It's like, when we are so interested to learn this, and then you say, "Oh, just discuss among yourself we think for 10 minutes, da ba da." Oh, 10 minutes. After 10 minutes, "Oh, okay, come back." It's quite... I would say mental draining, but it's quite tiring to be honest, to get so much of that 10 minutes peer discussion time. Whereas in year three and year four, there are less of those peer discussion time, and it's more about our interaction with the lecturers itself. I think it's better in this way, rather than you forcing us to have discussion among ourselves, when we meet alone can get those answers. Yeah. I think one of the factors that make me like the year three and year four better.

Student D:

From my point of view, I think that I don't like the year one year two, but I like the final semester better compared to other year, because one of the major factors from my point of view is the lecturer itself. Because for this semester I can feel more. What do you say, [inaudible 01:35:31] enthusiastic and to, I mean, they are more enthusiastic. Yeah. In... Discussing the questions that I'm not implying the previous year lecturers are not good. I'm not making the implication, but I just have to say that sometimes we are human being. We tend to feel what others feel. When we feel that the lecturers really care about all the questions, even though the questions you can directly get answered from the discovery, but you can still see the effort in explaining everything in details. Then you will feel that, "Oh, this..."

PART 3 OF 4 ENDS [01:36:04]

Speaker 7:

... in explaining everything in details. Then you will feel that, "Oh this lecturer is really care about me and I have to make up to her expectation." I have to do more to ensure that I receive greater feedbacks from the lecturers. That's what we get in the final semester. We really, really get a lot of

positive words from the lecturers that trigger us to learn, to ask more questions. Even though we know that our questions might be stupid, might be very simple, might be not going to come up in the exam, but we're still willing to ask. The most important factor is because of the lecturer itself. Yeah, just that support.

Speaker 8:

Yeah. Starting from your [inaudible 01:36:46], I start to get the feeling what Student D has. And yeah, it's really like a two-way thing.

Speaker 9:

Yeah. Okay. Thank you. Thanks for that. I am conscious of the time. It's 11:40 on my clock now. I said that we will end at 12:00. If anybody needs to go somewhere by 12:00, feel free to leave, but potentially we could go up to 12:15. We just have a few more questions and then we can [inaudible 01:37:21]. But if anybody needs to go, just let me know and just leave, okay? So given a choice. Yeah. Sorry. Given a choice, would you go through this style of learning? I know you're final year pharmacy students already, but given a choice, would you have still gone through this style of learning, number one. And what do you think about it when you're comparing this with what you've experienced so far? So think about what you've had in your style of learning in your secondary school or in your Pre-U. What do you think about it in terms of comparison and yeah.

Speaker 8:

I would still go through it. I do think that it's very different. I feel more confident now. I think now, if you knew me back then, but back then, I had really quiet. I don't talk to anyone. I'm even scared to talk to the lecturers, that I won't approach lecturer. I probably most likely will just talk to a different teacher and stuff like that, because I feel that, "Oh, we're closer." I think is actually the content delivered in interactive lectures. Old-fashioned one way delivered lecture is very different. In interactive lecture like ours, the scope was only to one certain topic. Whereas in a one way lecture, the lecturer can deliver whatever they want to up to let's say one hour time. So I think that interactive lecture helps me focus more on one topic and explore much deeper. It helps me explore much, much, much deeper into a certain topic and I know much more.

Then again, let's say in this eighth semester, you only know what was delivered throughout this eighth semester. Whereas in one way lecture, they can stuff like a lot, a lot, a lot of things and they just deliver it to you. I like interactive way of learning is that it gives you time to think what you have learnt. You have time for discovery and then you have a daytime of interactive lecture. Then you constantly have time to keep on reflecting oN what you have learnt in that one whole week. Whereas in the conventional lectures, you just sit there and then they actually deliver everything, then you have no time to think then it's the next class already. Yeah. So I feel it helps me learn better personally and it builds up my confidence. I think that now I know how I should pick up knowledge as in like throughout this through discovery.

There are some things that discovery cannot deliver, and if you want to know, then you find out more. And you are still in doubt, then you can ask your lecturer. So then it becomes like I'm more motivated to learn, whereas in the conventional ways, I just learn by what the lecturer has for me. Yeah. So I would still go through it.

Speaker 10:

Not only will I choose to go through it again, I would also prefer interactive lecture compared to the traditional way of one way teaching. This is because this whole new curriculum has really given me the flexibility of time. I'm in charge of my time, my study and everything. I can arrange my time freely. I think that in the traditional way of teaching, it relies a lot on the lecturer's knowledge and his teaching ability. Instead, in this interactive way of lecture, we are in charge. I mean, we take charge of

what we learn, at the same time, the lecturers are there to support us to clarify stuff. It has made me into someone who is more resourceful, that has the skill to be able to look up for information or answers on my own, instead of relying on someone else to give me the answer. I feel that it's quite a good initiative of Monash trying to Student De it into a curriculum like this. And yes, no doubt. I would definitely go through it again.

Speaker 9:

Thanks. Based on in [inaudible 01:42:14] similar thoughts or?

Speaker 11:

Actually yeah, it's similar. Can I say that initially the quality of the discovery is not there, making the interactive lecture very challenging. There's no cohesivity from one activity to another. And there's a lot of wasting of time doing the peer to peer learning thing. Something that has already been mentioned, and then you are also talking about it, and then your peers maybe, and then the lecture has to find someone to make them talk in the class just to make sense of the interactive lecture. But if the interactive lecture was started off just like what we are experiencing right now, where everything is cohesively fine, and we are discussing cases instead of the peer to peer sharing, I think it'd make more sense. And it's definitely a better way compared to all the previous methods that I've experienced. When you're giving a discovery, you feel obliged. So when everyone is prepared for the class ahead, the learning is more effective actually for myself.

So if I were to put back to the conventional way of lecture delivered to the class and the student, I wouldn't have read ahead. I would just wait here and then only I'll do my revision. But now it's like the other way. I actually have to read first, and then after I hear from the interactive lecture, then I need to reread again to make sure I understand what I've missed earlier. So that actually give a very good opportunity, and in a way it compel to them to work on their studies diligently. Yeah, that's my idea.

Speaker 12:

I think if I was given the opportunity to choose whether to join a traditional, conventional or interactive lecture, I would prefer interactive lecture. One of the most important issue for me is that because the time is really, really short compared to traditional. My friends in other universities, they have to go to school at 9:00 and can only leave at 6:00. But for Monash, I only need to attend school four hours daily. That's the greatest thing, because you can't expect me to sit there all day and be focusing on all lecturers. That can't be. So I think four hours should be okay, seems good to me. And if the school really take into our comments, our feedback and make some Student Des that suit to the student, then I believe that interactive lecture would be an enjoyable moment for the student.

Speaker 9:

Okay. Thank you. I think because a lot of you have the same things that we wanted to find out earlier already, but if I can maybe just give some of my understanding of what you're saying. Again, you all just let me know if I misunderstood it. We wanted to also find out whether this style, this method of learning is useful for all your different units that you've had so far. So from some of your earlier comments, the sense that I get, that it seems to be useful for some of your CC units, but maybe not really for your PP units. Okay. If I can take a step back now to your earlier years, like your years one and your years two unit, do you think this style of learning where we give you discovery and all that, was it good? Was it useful or not?

Speaker 8:

I think it's definitely useful for CC and integrated care. As for PP, yes, we can read, but we might not understand. A lot of things, hard to say, really need some explanation before going into a picture. Then for how medicine works, especially because it's very chemistry based, a lot of things it's not like, "Oh, you read then you know why the electron jump from here to there." That kind of thing. So a lot of consolidation is needed in the interactive lecture. But of course every time... Yeah.

Speaker 9:

Is it useful? Do you think this style works for teaching chemistry or not?

Speaker 8:

Half-half. Not really.

Speaker 9:

Half-half?

Speaker 8:

I think a person will only get 50% of what was intended to be delivered. And time constraints for interactive lecture is only one hour. So maybe a lot of things need to be explained in a very short time. So I think the knowledge base for home exemplars was not very well built up for me, I think. Yeah. I think I've forgotten everything that I've learned, almost everything that I've learnt in home exemplar now. The application that we see, we can't really see the impact and application of it yet. I think that's why. So probably maybe... I know there is time for clarification of discovery in the style of interactive lecture, but I'm not sure how the discovery material can be Student Ded to convey the concept or gauge understanding of them.

Speaker 9:

Okay. Thanks.

Speaker 8:

For me it's just-

Speaker 9:

Yeah.

Speaker 8:

50%.

Speaker 9:

Yeah. Thanks.

Speaker 7:

I think for PP, those moral units, maybe we need sometimes to interact with the lecturers and they give us some real clinical case. That we can know whether our morals, whether it's up to expected from the ethical requirement for pharmacies. For example, the tests that we have to do it every year, the judgment scenario. Actually we only get the result. And I don't know why my answer... I mean, every year I almost pick the similar answer, but every year the feedback would be different. I don't know why. I mean, I don't know why in some scenarios, I think that that answer is the best, but it comes out, it shows I don't have empathy at all. Now I have a lot of question marks in my mind as no

one explained to me, what is the optimal answer in that scenario. And now I'm going to leave Monash and I will carry those question marks with me. Yeah.

Speaker 9:

Okay. Thank you. Anybody else?

Speaker 10:

Yes. This mode of learning, I personally feel it's very useful for [inaudible 01:49:20] but for year ones, I personally felt discouraged. My theories were too heavy and interactive lecture and workshop, like what Jenin's just mentioned has no relation to what we have studied on discovery materials. I feel that it could still be better structured. As for professional practice, I personally feel that it's not really that useful. I think the most classes that I have skipped is professional practice classes. Yeah. Because I personally felt that those stuff, I mean, I can study on my own, so I don't really need to go for classes or for those PP units. But it also depends, but for stats and for professional practice where it involves us solving community case, then I would say it's still a good way of learning. But for research, I think we need more time in clarifications instead of us doing our own discoveries.

Speaker 9:

Thank you. Okay. We've talked a lot about a lot of things today already. Lecturer B, do you have anything else to ask or to add. Hello.

Lecturer B:

Sorry, sorry. I forgot to unmute. Yeah.

Speaker 9:

Okay.

Lecturer B:

Yeah.

Speaker 9:

Anything else you would like to ask?

Lecturer B:

Well, I guess we will just finish up the session the two final questions of which you actually have already partially answered. The first one would be in terms of flip, I mean from the old syllabus that you've already gone through. How do you think it's helped you? Do you think it has helped you grow as a person in any particular way? I think Shen Ying has mentioned a few things, but how about everyone else? Do you feel that the syllabus helps you in any way going through this whole thing? If you have anything else to add from what's already been said. I think some of you have already said some things.

Speaker 8:

To me. I think it really helps me and makes me to think of what I've learned. Yeah. So then when you start to reflect, then you know what I should improve on. Then throughout the years keep track of the consistent of this practice. Then I start to learn faster. I learn quite fast now. I'm more confident in voicing out my doubts or more like voicing out my opinions. I start to take charge of my own learning. In workshop, I start to take charge of the whole group, teamwork, the lives and all that. I

start to find the value of peer learning. I start to appreciate that learning is not just about the lecturer, but it also depends on myself. If I don't put in effort, then the lectures I thought, will go down to the drain. Like, yeah. So that helped me to grow as a person throughout these four years. Yeah.

Speaker 9:

Thank you. Anybody else?

Lecturer B:

Okay. I constitute this. I don't know why everyone's video is frozen in my screen. So I can't actually see anyone's reaction.

Speaker 9:

The same is for me, it's [inaudible 01:53:42].

Lecturer B:

Okay. Yeah. I'm not quite sure what's happening. So I don't know whether... But anyway, since everyone was silent, I assume that that was more or less it. The final thing would... Actually, did anyone have anything else to add ask? I can't read expressions now because the videos are frozen.

Speaker 10:

What [inaudible 01:54:01] it's about more in charge. It's like giving us the opportunity to be in charge of our studies, our times. At the same time us being resourceful in getting our answers. We tend to be more proactive in seeking answers instead of just students sitting there and wait for someone to give us the answers of the syllabus.

Lecturer B:

Okay. Cool then. Okay. So very similar views. Okay. That's fine. Any contrasting views towards this? Do you think that you've actually lost anything or did it... Do you think there are any disadvantages in that sense? To yourself, to going through this system. I was just a bit curious on whether you felt anything negative towards this.

Speaker 8:

I think the major drawbacks would be each way, like only focus on a specific topic. So then it becomes like, let's say each semester 12 weeks. Right? So 12 times eight, which will only cover that much of topics throughout our four years of studies. Then the concept where really like more... The whole concept of the syllabus is that they may want you to understand the very core concept of these specific topics. Whereas if it's a free classroom then the depth of knowledge, the scope of topic being covered is all based on the lecturers. So maybe that's a difference between this and flipped classroom. But of course the take away from interactive way of learning is much more deeply rooted in myself compared to conventional ones. So that's what I thought.

Lecturer B:

Okay. All right. Thanks a lot for that. Then I guess the final question will be-

Speaker 8:

Sorry Dr. Lecturer B.

Lecturer B:

Sorry? Do you need to go off?

Speaker 8:

No, no. There's another student who would like to-

Speaker 10:

Oh yeah.

Speaker 8:

Point something in.

Speaker 10:

Yes.

Lecturer B:

Oh, sorry, sorry.

Speaker 10:

Yeah, yeah.

Lecturer B:

Sorry, I can't see the video. Sorry. Yes?

Speaker 10:

If you do not develop the discovery properly, that means it will subsequently affect the interactive structure and workshop. Students will be blur going to interactive lecture despite studying discovery and also be confused during the workshops there. So I think it's quite important in getting the discovery really done, yeah. And also the student's initiative and practitioners in actually studying the discovery before lectures. I think it's quite important. Yeah.

Lecturer B:

Okay. The final question I have is actually what you, more or less just answered. It's do y'all have any suggestions that you think can be used to improve the program in a general sense. Also, perhaps we can contrast this by, because you have some online things that you like. Let's say next year, if we go back. Let's say, is there anything that you would take from the online that you would implement offline? Or anything like doing it a hybrid in any sense, like if you could redo things.

Speaker 12:

I think I will take some things from the online, for example, discovery materials to the clinical setting, because some of them are quite useful. But most of it, I think I'm just doing for the sake of the exam, the final exam. So I'm doing it for the exam only, but definitely some are helpful. And in terms of improving the program, I think the school has to be clear that the sequence is correct. For example, you must put the discovery before the workshop and not the other way around. You shouldn't put the workshop first, then only discovery or interactive lecture. If not, then it doesn't serve the purpose of discovery and interactive lectures. Because sometimes in our course, I think there's only a few sessions in which we have to attend workshop first, then only we go back to discovery. Throughout that, I think only a few weeks, we feel that we don't know anything about a topic if we

are forced to go into the workshop first, and answer the clinical case before the discovery, the interactive lecture.

This, I think the school have to really ensure that the sequence is correct. The discovery day, I think the best is if the discovery day is Friday, because we can prepare better. If the discovery day is Wednesday, then it's halfway. I mean, should I prepare for tomorrow case, or should I prepare for the next week discovery material. There's a dilemma that we have to pick up. That's why I think the sequence is the most important thing. And it's something that the school can do, can modify.

Speaker 10:

Some of the things that I prefer online learning is, maybe can home see that having interactive lecture online, if yeah... And another part is that, I really like the idea of the lecturers compiling all of our questions and then sending us email, or answer them all together on the forum so that everyone can learn. I also like the idea of having the [poll 01:59:35] every link open at least any one day or two before the interactive lecture, because it gives us more time to actually send in questions. Instead of having them open only for one or two minutes, right before the interactive lecture. Other than that, I think the discovery can be better developed. And overall for research, the structure can be better. At the same time, I know that we are studying an Australian syllabus, but perhaps can try to input some of the very common diseases in Malaysia, such as dengue in our curriculum. Yeah.

Speaker 8:

For me, I think maybe for the year ones, you need probably the discovery scope could be refined to make us see the application, or maybe link it through the interactive lectures, the workshops, and yeah. Year one has no host a loop, when then we were having year ones. I think something to keep is the host a loop, which is very important to properly, constantly get every ideas or clarify doubts and close the week. Like properly so everything is clear. As for what we can take from online learning to the offline learning is I think the question and answer session. It's very successful in virtual classroom. So if that was, I'm not sure how it could translate in to physical classes, but if the same thing were to happen in physical classes, it's actually very beneficial to me at least. Yeah. Yeah. That's all that I have.

Speaker 11:

I'll just add on, right. I think that it would be good if we can maintain the IL through the online learning to save the students on time. If possible, maybe we can do like a lecture for like a consolidation during the CTL or something like that. Just to refresh before they start the CTL, just to close, which is already done now. But probably the content always getting increased, because it may help some weaker student who may not be benefiting from a discovery. So they got a second Student Dce to attend the lecture, probably a short one, since the IL will be online anyway. So that may require additional hours, but I think it will benefit for those students, especially myself. Sometimes if, you know, some of this career can be very long, some of this career can be quite short. So those that cannot manage a longer one. So they can attend the optional lecture after the interactive lecture, just to catch up on the part that they didn't do discovery well.

Lecturer B:

Okay. Yep. All right. Thanks a lot for your feedback. And I think that's probably the... I'm kind of done with my questions. Wei Jing, do you have anything else to add?

Speaker 9:

No, but just to summarize just some thoughts, can I assume that again, taking a very general view on this, that the general feeling towards flipped learning or the style that you have gone through in

Monash is positive. That you're a bit more, you like it, but obviously with some rooms for improvement and some Student Des here and there, as to what you all said earlier. Is that generally the consensus? Your screens are frozen for some unknown reason. If it is just give me a thumbs up or just put yes in the chat group. Or you can just say yes. Yeah.

Participants:

Yes.

Speaker 9:

Okay. All right. Cool. Thanks. All right. Thank you. With that, I think that ends our session. We've constantly gone over time-

Lecturer B:

Yeah, sorry about that.

Speaker 9:

... and I do apologize for that. Again, thank you for your input and your feedback. I'm just going to stop the recording here.

PART 4 OF 4 ENDS [02:04:19]

Deidentified FG5Y1, Sept 2020

Lecturer A:

Briefly as well, can I just ask what's your preview background for the both of you?

Student A:

Yeah, you go first.

Lecturer A:

Okay, Student A, what's your preview background?

Student A:

I studied UEC before this.

Lecturer A:

UEC, is it? Okay. And Student B?

Student B:

I'm same as well, UEC.

Lecturer A:

Okay.

Lecturer B:

Oh, okay.

Lecturer A:

Thank you. Okay. Over to Lecturer B. Yeah.

Lecturer B:

Ah, okay. So the first question is very general. So how has the online experience been so far? So you all started online in year one. So overall, what do you feel right now? Just up until now, just tell us what you feel about that.

Student B:

Quite okay, I think. I think everything we should study and we can also get the same as well using online.

Lecturer B:

Okay. Student A, how do you feel about it?

Student A:

At first, I'm a bit happy because yeah, I'm from Sabah.

Lecturer B:

Oh, okay.

Student A:

So I can do everything comfortably at home so and in my own time, so I don't need to follow the timetable. But now I don't really know anyone in uni and I'm so desperate to go back now.

Lecturer B:

Okay, okay.

Student B:

Actually, that's also my problem because I'm from Chinese independent school and all my friends, my group mates, they're from the foundation in [inaudible 00:02:07].

Lecturer B:

MAFI. MAFI.

Student B:

Oh yeah, they are all from MAFI so they prefer their own MAFI friends instead of us because we didn't actually meet each other. So that's also my problem of friends. Yeah.

Lecturer B:

Oh, okay. So okay, which I can imagine it is a problem because yeah, it's online, you suddenly go and you don't meet anyone physically. [inaudible 00:02:36] try enough. Okay. So I guess, wait, let me go through. So when you all started... Actually, do you all come to campus a little in the beginning? [crosstalk 00:02:55] in the first week or two, did you all get the chance to actually enter Monash or?

Student A:

I went there during 14 March and came back to Sabah on the 18.

Lecturer B:

That's quite short.

Student A:

Yeah.

Lecturer B:

Okay. Both of you know each other is it? Because both of you are from UEC.

Student B:

No, actually.

Student A:

No.

Lecturer B:

All right. So I guess the next thing I want to ask is how do you all feel about the organization of the online thing from the very beginning, in sem one?

Student A:

I think it's quite well because it's such a short time but the lecturers, they managed to arrange everything. So organizer in the [crosstalk 00:03:57] problems.

Lecturer B:

Okay. You felt it was okay then. Student B, how about you? In terms of timetabling, the Moodle communication and everything, do you think it was okay or could it have been better or?

Student B:

At first, I'm quite confused, I don't know what should I do actually. Although the lecture have from a video tell us what to do but I don't know the video is [inaudible 00:04:22]. So actually at first I didn't watch the lectures, I only study the discovery. Then after one or two ways, I only discovered that, oh, I also have to study the lectures. So yeah, this my situations.

Lecturer B:

Yeah. So it was a bit confusing initially. All right. And then so now in the second sem, do you think there's been an improvement or do you think it's still a bit confusing or? Yeah, if you contrast sem two to sem one, what's your feeling about this?

Student A:

What I like about sem two is their live lectures and there's a weekly clarification system [inaudible 00:05:08]. So yeah, I get to ask the lecturers things, it's live that I get to ask things sort of like in person.

Student B:

For me, I also think that sem two is well organized and everything is very clear. The lecture we have our link and Zoom link on the Google form [inaudible 00:00:05:38]. Yeah, so everything is very clear and I can find everything I need.

Lecturer B:

Okay, okay. All right. So perhaps in a sense sem two is a bit more organized than sem one in a way. And so in sem one, you didn't have this clarification thing at the end? Like this [inaudible 00:05:58] thing or was it not [inaudible 00:06:01]?

Student A:

I think if it's sem one only professional practice but it start from week nine.

Lecturer B:

Oh, okay. Okay.

Student A:

And it's not every week, maybe once in two weeks.

Lecturer B:

Okay. I guess then the next question will be, so again, you all started university online. Were there any concerns, the moment you all came into Monash and you originally expected to go to a campus, meet people, go for classes, things like that? But actually what really happened is that suddenly COVID happened and then you found out that you probably need to spend one semester at least, or

well now you know it's the whole year online. What were your initial concerns or thoughts about this?

Student A:

I mean I imagined my uni life to be not just studying, there's a lot of club activities. Yeah, a lot of events I can get to meet other people, not just from my course but now yeah, everything is just studying only. So I'm a bit disappointed, I just want to go back soon.

Lecturer B:

Student B, do you feel the same or different or?

Student B:

Yeah, I also expect my uni life to be more interesting but actually, because I learn new things so it's a new thing for me and I like this actually, it's a new experience.

Lecturer B:

And so you kind of found the online thing somewhat interesting, is it?

Student B:

It's not interesting, it's very comfortable. Yeah, our lectures is on night and I can wake up at [inaudible 00:08:24] instead of in the morning. So yeah, and I don't need to prepare myself, I can just sit on here, that's why it's comfortable.

Lecturer B:

Okay. Okay. Well yeah, in that sense, yeah, comfortable, a bit more convenient.

Lecturer A:

Yeah. Looking at your background, I think you are in the same room with Dr. Lecturer B right now.

Lecturer B:

Yeah. Okay. So I guess one, if I ask the question in a slightly more education-based thing. In the sense that before maybe, UEC of course you had live classes, everything was [inaudible 00:09:07] an offline class. And then now you switch to online. Initially, were you worried about any particular aspect of the education experience when starting to do online thing? Was there anything you were concerned about?

Student B:

Something I felt not that good is sometimes our lectures in sem one our lecture is recorded, so sometimes the lectures didn't, there are some technical issues. We can show the pointing of the lecture so the lecture was at here, here, here, and we don't know where is it. And sometimes the style is not clear or the voice will be very soft and suddenly very loud. Yeah. And sometimes when we don't really understand what the lecture is talking, we can't really find our lecturer and ask them to clarify for us. Yeah.

Student A:

Me I think [crosstalk 00:10:19]. To me, I think it's okay because when I don't know anything, like I don't understand the lectures, they just give you the lectures and they usually respond very quick. Like maybe one or two days.

Lecturer B:

Okay, okay. So the response in year one has been reasonably good. Okay, okay. Okay. Then the next question I have is about assessments. So you've probably had a few types of assessments, I guess workshops will be one of them. You might also have exams, you already took some exams in year one. And you have all your in semester assessments in, well, usually in e-assessment platform. How do you feel about these assessments? Yeah. What do you think about these assessments?

Student B:

For the mid-year assessments, I think it's easier because we have open books but for the final exams, it's turned out to be very tough. Yeah, because it met for open book and we don't really have enough time to finish all our questions. Yeah.

Lecturer B:

Okay. It was quite difficult. All right. All right. Then for Student A, how do you feel?

Student A:

For last semester, I think the in-semester tests are okay. But there's a presentation, I think a group presentation for professional practice. I feel like it's harder to do it because we need to prepare the slides separately and yeah, [crosstalk 00:12:35]. Yeah, we need to prepare slides separately and sometimes when we plan to practice together, then we got internet problems.

Lecturer B:

Oh, okay.

Student A:

And during the assessment, yeah, our slides hang there for quite some time, so I don't know if it's affecting anything. And for this semester, maybe I enjoy too much completing my things in my own time now. So I sleep very late and wake up very late. So during one assessment, I woke up half an hour late for the assessment is a one-hour test. So yeah, I lost half an hour to finish the test but it's still okay. I got, yeah, quite satisfied for the results.

Lecturer B:

Okay. So I guess online can be challenging for working together in that sense because yeah, maybe your group mates may have technical issues. And also because you can't really talk face-to-face that easily so working on a group assignment together is difficult. And I guess you also mentioned time management is a bit challenging, is it? In terms of self-management. Okay. Yeah, all right. Thanks, that's good. I'm going to continue another questions on assessments. Do you feel that the assessments we have are a good measure of your knowledge and skills?

Student A:

Sometimes I wonder if everything is open book for the final exams. So sometimes I wonder if I'm really competent because maybe open book exams are easier, I don't know. [inaudible 00:15:09].

Student B:

For the question that sometimes I don't know so I can search online so yeah.

Student A:

It's easier to find answers but sometimes I don't know if I'm expert enough or [inaudible 00:15:31] enough to pass the course if I'm really doing this close book.

Lecturer B:

And last time all your exams are close book, is it? In UEC.

Student A:

Yeah.

Lecturer B:

Okay.

Student A:

Except for SPMs [inaudible 00:15:48].

Lecturer B:

[inaudible 00:15:50] oh, okay. Didn't know that part. And do you feel that the assessments are fair?

Student B:

It is quite fair.

Student A:

Yeah.

Lecturer B:

Okay. You don't feel that there's some advantage or disadvantage to anyone based on the fact that it's open book and... Okay, okay. Yep, all right. That's good. And then do you all have any technological challenges, hardware, software, internet connection, or anything during this whole online thing? Was it a big challenge to you or was it not much of a concern?

Student A:

Last few months there's one week my internet connection is very weak. There are I think four workshops that week and I got disconnected out of three. Yeah.

Lecturer B:

Ooh, okay.

Student A:

So it's quite unstable during that time, the internet.

Student B:

Yeah. I change my connection so as they go for the whole year actually. Yeah, so during workshops I was disconnected and also exams I lost the connection, so I have to use my phone data to connect the internet. Yeah.

Lecturer B:

Oh, okay. So but the phone data solves the issue, is it, more or less?

Student B:

My phone data is quite stable so it's no problem. But during workshop, I almost will disconnect during every workshop and lectures we had Zoom. So it's quite a problem for me.

Lecturer B:

Then I guess the next I have the follow-up question will be then in assessments when you have your final exam and things like that, wouldn't that be a bigger issue or anything?

Student A:

[crosstalk 00:18:17]. Yeah, you go first.

Student B:

Yeah. So when it happens so I connect my data using my phone data.

Student A:

Yeah. I'm lucky enough to not experience that problem during my assessments so far. Yeah, I hope not [inaudible 00:18:37].

Lecturer B:

Okay. Okay, was fine at least. Okay. So then maybe to dig a bit deeper. When this connection issues happened, how do you feel about them? Does it cause you distress or is it something you just deal with and it's not a big problem or?

Student B:

I'm quite frustrated, like again.

Student A:

For assessments, yeah, like I said, I haven't experienced that but if I do, I will panic and I get very lost, and yeah. I think I will, yeah, freak out.

Lecturer B:

Okay. Oh, all right. And I guess then another question that's a bit in the same line is, okay, so in, I guess when you come to Monash, you'll notice there a lot of new software you use, right? Moodle might be quite new to you, you might need to use things like Poll Everywhere, then the whole Google suite of things, all these kind of things. So all these new technology software and all that, was it easy to transition in? Or how was the experience of having to go through all this suddenly Zoom and all these kinds of things?

Student A:

For me, I think it's not a big problem because I like to click around with these things. I'm not scared of getting it or method or something, I can always start over if I destroy anything so.

Student B:

I'm not good at this kind of thing. So at first, I can't really find the things I need but after the lecturer got tells us how to use these apps. So at the end, it was fine.

Lecturer B:

Okay. Oh, okay. So for Student B initially, a bit difficult but eventually adapted quite okay. And for Student A, you are very tech-savvy so it was actually okay from the beginning. Okay, very good. Okay, cool. Lecturer A, is there anything I missed out in this portion?

Lecturer A:

Yeah. No. I just wanted to ask, when you said, how long did it take for you to familiarize yourself with the softwares? Roughly, what did it take, one week, two weeks, one month, three months?

Student B:

One to two weeks I think.

Lecturer A:

One to two weeks, okay. Okay. And then I think I just wanted to explore a little bit more on this whole aspect of starting university in an online environment. As we all aware of what is happening across the world is forced us to change how we do some of the things that we normally do. And we had to do it very quickly. And I think both of you all did share earlier that you all had personal expectations of when you all started uni of how you all think a uni life could look like. And then so far because of all that is happening, the expectations hasn't matched up with what you are currently feeling right now. But I'm just wondering also, are the concerns still there? Because the reality is that we have already created opportunities for you to interact with your fellow classmates. Even though via computer screen, through your workshop activities and all that and there is one platform of how you can make friends. And I think I believe [inaudible 00:22:38] also has had some activities to help. Yeah. But yeah, so what do you all think about that?

And again, it can be very frank and honest answer and it's all confidential. Yeah. So Student B, I saw a reaction first that you want to start.

Student B:

Yeah. Actually, I was a staff committee of [Mostar 00:23:01] so-

PART 1 OF 4 ENDS [00:23:04]

Student B:

Actually I was staff committee of [Musa 00:23:02], so I have involved in planning those activities. But the response was not good.

Actually, for the first activities in Sem One, we have online games. The participants, it's around 20 I guess. I can't remember. But for Year One student, I think it's less than 10. So we're very, very less, so it didn't turn out nice, the games.

Also, via Zoom, we can't really interact with our friends when you will close their video and keep silence, so it's not that good.

Lecturer A:

Okay. Student A, anything similar, or anything different?

Student A:

I think similar with her is everyone close the screen, and you can't see their reaction. I think making friends is not only just during workshops, or interactive lectures, or something. Maybe after class you can go for lunch, or during the weekends you can go maybe shopping or something. So I think it's not enough to know them more.

During high school, I have friends that very attached to. We go out quite often, even still now. But now, I don't feel like I know anyone good enough to have that kind of friendship so far.

Lecturer A:

That's very good. Very honest answer, that. Thank you. Now I'm going to ask another next question. [Quyan 00:25:21] did say she was part of the committee that helped organize those orientation or activities, right? Student A, did you go for this activity?

Student A:

I did go for some activities, but I'm not sure if it's organized by Musa.

Lecturer A:

[Mufas 00:25:43].

Student A:

Mufas pharmacy. Yeah.

Lecturer A:

Okay. So you did go for this?

Student A:

Yeah, but it's not as exciting as I thought. Everyone turn off the screen, so we can't see. You can just see a bunch of names.

Lecturer A:

Yeah, you just see a bunch of names, or a bunch of avatars.

Student A:

Yeah.

Lecturer A:

Fair enough. Okay. Obviously making friends was a very important thing that y'all wanted to explore and build on when y'all started university, which y'all have already shared about that. So far, that has been a struggle because of the limitations or the challenges that we have.

What about, was there any other things about starting uni that y'all were excited about? Like, "Oh, I'm now an adult already. I'm starting university life." Or, sorry, "I am a young adult now," or, "I'm going to have more independence in the decisions that I make."

Student A, you said you're from [Interstate 00:26:51], so I'm assuming uni start, you were going to be staying on campus, or close to campus. I'm assuming that has some elements of yes, to me university was I'm going to move out of my parents home, and I'm going to be a own person already. I don't know, any of these ideas or not?

Student A:

At first before the MCO, I went there. And to be honest, I got homesick for almost a few days. I was crying every day, "I want to go back." And when the MCO started, I was so happy I can finally go back. But after the mid-sem break last semester, I was very excited to meet everyone in university because

I just can see their face and... I don't know, it's different now when you meet someone physically and virtually.

Lecturer A:

Of course, yeah. Agree.

Student A:

I met some [inaudible 00:28:08] that are very, very friendly, and very nice to me. So I'm very excited to go back. So from that time... Even though I'm safe at home, I don't need to worry about getting up late because until now my parents still wake me up.

Even though I think I'm not really ready to be that independent to live alone, I still want to go there.

Lecturer A:

And Quyan, about yourself? Any other themes, or any other feelings?

Student B:

Actually I live in [Putrong 00:28:59], so it's-

Lecturer A:

Okay. That's close. Okay.

Student B:

Yeah, it's where I live, and I went to Sunway every week, so it's all in my comfort zones. But I expect to join some clubs, also that stay up late for we get home. There's something I expected to do, but yeah, it's not now.

Lecturer A:

Why is this wanting to join these clubs, and all these activities, why are they important to you? Why do you think it's important? Or why do y'all want to do it?

Student B:

I like new things, as I said just now. I like exciting things. Instead of joining club, I also think of doing some part times, but I don't really can find some now because the economy is not good. So only thing I can do is stay at home.

Lecturer A:

Okay, thank you. Student A, anything else to add, or anything else to share?

Student A:

Not much.

Lecturer A:

Not much. That's okay. All right, thank you for your honesty. That's all from me. Lecturer B, back to you.

Lecturer B:

Sorry, there was one question I missed out just now. If we zoom into... We have discovery work, interactive lectures, workshops. And you have closed loop lectures, do you? Or do you have summary lecture at the end where you can ask questions nowadays?

Student A:

Yeah.

Lecturer B:

It's both in HMW and also Professional Practice, is it?

Student A:

Yeah.

Lecturer B:

Both. Okay. All right. I'm going to ask your opinions on what you feel about each individual component. Let's start with the interactive lectures. How is it for you? These online interactive lectures?

Student A:

I think this semester is better than the last one because there's live one.

Lecturer B:

Okay. Last semester recorded, this semester live. All right. And Quyan, do you refer either or?

Student B:

Yeah, I am okay with both, with or without the live lectures.

Lecturer B:

Okay. So for you, more or less the same. Why don't you have a preference?

Student B:

I'm not sure. Because I don't actually ask questions during the live lectures. When I didn't understand, I prefer to address it myself. If I really don't understand, I will post it to the forum, so it's more or less the same.

Lecturer B:

Oh, okay. And for Student A, you like the live one because...?

Student A:

Because I can ask questions, and let them answer immediately.

Lecturer B:

All right. Okay, then let's move onto workshops. How do you feel about the way workshops are done online in general?

Student A:

For Professional Practice, I think it's quite fun because the time is quite sufficient. It's not rushing. But even if it's over the time, the lecturers, they don't seem to rush us. They just go on until the whole workshop is finished.

But for last semester, How the Body Works, and this semester, How Medicines Work, the time is quite short, and we need to do a lot of case studies. Sometimes we need to do presentations again, and I'm not really good at presenting. I don't talk very well. My public speaking skills are not good, so I need time to prepare. I really need this kind of thing, so I think I can't really adapt to these kind of workshops.

Lecturer B:

All right, so you feel that the HMW and How Body Works is too fast paced, quite rushed?

Student A:

Yeah, it's not enough to search for things and prepare.

Lecturer B:

Understood. Is it... Compare same to the same one, so in How Medicines Work compared to How Body Works, do you think that it's really better now, or it's still just as stressful as it was before?

Student A:

I think it's more stressful because for my group, none of us on the video, so I don't really know what others are doing. And when we discuss things, it's always in the chat area. There's no...

Lecturer B:

Okay. So communication a bit difficult because everyone off their video, then you cannot feel that anyone is there, is it?

Student A:

Yeah.

Lecturer B:

Okay. Quyan, what do you feel about the workshops then?

Student B:

Quite same as Student A. The Professional Practice is comfortable. It's in small groups, so everything is quite flexible, and we just have to talk about our opinions, not really pretending, so it's quite easy. But for the How the Body Works and How Medicine Work, it's stressing because we have to search for the informations, and prepare for our presentations.

Actually, I am from Group Two last semester, and Group One this semester. Our group is always the first to present, so sometimes we didn't have the time to sort out our information, and we have to do the presentation already. So it's not really stressed, but quite rushed.

Lecturer B:

Similarly, do you feel Sem Two is more rushed than Sem One, or...?

Student B:

For this, my group, they will turn on their videos and we will discuss verbally, so it's okay for me. But I do encounter some problems like Student A, during interactive lectures. I think it's the first or second interactive lectures, the lecturers randomly break up us. Sometimes our group will discuss via chat, so I don't like that.

Sometimes I will voice out, by my friends, my teammates, they will answer me via chat. I prefer [inaudible 00:36:59].

Lecturer A:

Okay. So Quyan, you prefer talking versus chatting, right?

Student B:

Yeah.

Lecturer A:

But Student A, are you okay with chatting, or do you also prefer talking? I know the experiences you've had so far, most of the people all chat. But if you can control it, what would you like?

Student A:

I would like chatting, but I tried that before. You mean chatting is like now [crosstalk 00:37:29]?

Lecturer B:

Typing.

Lecturer A:

Talking or typing?

Student A:

Okay, talking.

Lecturer A:

Okay. You prefer talking as well, but reality, the experiences that you've had so far in your group was that most of your friends all end up just typing in the chat box?

Student A:

Yeah.

Lecturer A:

Okay. All right, thank you.

Lecturer B:

Okay. Why do you feel they don't want to talk? Because both of you prefer talking, but how come everyone else doesn't want to talk?

Student A:

I don't know. Maybe their place is noisy. I don't know.

Lecturer B:

Do you think it's a good idea if the lecturers force everyone to on their video?

Student A:

For workshops, yes. But interactive lectures, no, because too many people... I think some of our computers will hang.

Lecturer B:

Okay. So you're saying in breakout rooms, it's probably a good idea to force everyone to on video, is it?

Student A:

Yeah.

Lecturer B:

Quyan, do you feel the same?

Student B:

Yeah, I feel the same.

Lecturer B:

Okay. Then finally will be the Close the Loop thing. Close the Loop is quite new to you, is it? It only happened this sem. Last sem, it didn't really happen. So what do you feel about the summary lectures that you have?

Student A:

I'm okay.

Lecturer B:

Are they much different from the normal? The initial lecture and this final lecture, is it quite same, or...?

Student A:

For this semester, How Medicines Work, I think it's quite the same. Sometimes it's quite rushed. But Professional Practice, they sometimes teach different things, different from the first [inaudible 00:39:33] lecture.

Lecturer B:

Oh, okay.

Student A:

So I think, yeah.

Lecturer B:

Quyan, any thoughts on this? This summary lecture, is it useful, not so useful? Or is it more or less the same [inaudible 00:39:53]?

Student B:

For me it's more or less the same.

Lecturer B:

Okay. But usually the first and final is on the same topic, right? It's basically you have one topic, you have interactive lecture, a workshop, and then final summary lecture, right? That's the system in year one?

Student B:

Yeah.

Lecturer B:

Okay. Because from year two onwards, that's exactly how it is. I was told in year one, it was slightly different, but okay I just wanted to make sure because I wasn't fully familiar with what you're going through. Okay.

Let me go through... Those actually were a lot of the questions that were dealing with the online experience. Now I'm going to ask some questions that are more related to the general syllabus in a more general sense, not just online. Maybe we want to talk things in general.

In your case, what do you feel about discovery materials in general? This is probably something new to you. What are your thoughts on it when you started with this here? Any thoughts?

Student A:

For me, I like to print out the discovery and read it when it's printed in paper. But some of it, especially for this semester, How Medicines Work, there are a lot of videos. I need to screenshot the slides, and print them out. And need to watch the videos again. For me, I prefer reading more than listening to the videos, so for me I prefer it written.

Lecturer B:

Oh, written. Okay.

Student A:

Less videos is better for me.

Student B:

For me, I'm on the opposite. I like video more than written. Also, the slide, actually we can download it. So actually [crosstalk 00:42:27].

Student A:

But some can-

Student B:

Really? For me, I like to scan the discovery for the first times. And after a few days, I will do an in-depth study, so I will have to read the discovery materials twice before I can fully understand all the things that I need to know.

Lecturer B:

Okay. All right. Discovery materials. I guess then a question I would want to ask, both of you are from UEC. In UEC, how it was done was probably the teacher is standing in front of the class, and then just lecturing, right? Is that correct, because I didn't go to UEC?

Student A:

Yeah.

Lecturer B:

If you want to compare that to how it's done in Monash, which do you prefer? If we switch your pharmacy program to the way it was done in UEC verus the way we have it now, discovery, IL, workshop? Do you have any preference?

Student A:

I think more interactive lectures. And sometimes explain the discovery materials more during that time.

Lecturer B:

Okay. Actually, do you mind having the discovery, or you prefer just one lecture that's not much discovery? It's really just you go into a lecture directly, and then the teacher is standing in front of them going through slides and explaining the discovery material? Or would you prefer the way it's done now, which is you read the discovery yourself, then you go for interactive lecture?

Student A:

I think reading first is better because if it's only lectures, then I wouldn't have that much materials.

Lecturer B:

Okay. All right. So, you prefer the way it's done here in a sense?

Student A:

Yeah. But one thing I really want is they give us more past year papers.

Lecturer B:

Past year papers. Okay, that's for the assessment part of things. All right. Okay.

Quyan, do you have any thoughts on this?

Student B:

I think having the discovery beforehand is better, so we can study first, and if we have any doubts, we can ask our lecturers during the interactive lectures. So I think it's better having the discovery.

Lecturer B:

Okay. But what didn't you like about the system in UEC?

Student A:

They didn't have any oral exam, so I'm not really trained to speak fluently in public or something. And sometimes I can't communicate well.

Lecturer B:

UEC, everything written-

PART 2 OF 4 ENDS [00:46:04]

Lecturer B:

You will see everything written, a hundred percent written.

Student A:

Yeah.

Lecturer B:

Okay.

Lecturer A:

Can I ask first, also on that thought, can you tell me now what are the obvious differences in what you've experienced so far in your first year uni and your experiences in UAC, but don't talk about the online aspect, but just taking a big picture view, what have you gone through so far in uni that you say, I didn't have this in UAC or this is new to me.

Student A:

I had no opportunities to do public speaking. Even I don't like to speak in public, but that's something I should learn. So I think it's better than UAC at this.

Lecturer A:

So, to summarize to Student A, you've experienced so far and you need how come in my learning in my classes, they get me to talk a bit more than in UAC. So you think that this is good and useful for you, is that correct?

Student A:

Yeah.

Lecturer A:

Okay. And Hu Yang what about you? Anything that you've experienced so far?

Student B:

I think quite the same with Student A, although our school, they asked us to do some presentations, but it's not that much. Yeah. So, and USC is quite boring because I was busy.

Lecturer A:

Sorry, you what?

Student B:

Busy.

Lecturer B:

Busy.

Lecturer A:

Okay.

Student B:

Because we did both SPM and UAC. So during form four and five, our school focus on SPM. And form six, we did all the study, all the reading materials from form four to form six. So we studied a three years thing in one year, so it's very busy.

Lecturer A:

I see. So it's too packed by that, like two combined season? I see.

Student A:

Same for me.

Lecturer A:

Same for yours Student A, okay. Alright. So you see, it's nice that even though you all have just met, face... I don't know whether you have seen each other before this, but now this opportunity you're to see each other and you'll find that you've got some similarities already or similar background experience before coming to here in uni. Okay. Was there anything else, what about the way the teacher conducts himself or herself compared to UAC and compared to what you've had so far, is the approach different? Or do you feel like it's the same or anything that came across your mind during one of your classes, in a day. How come I've not had this before in UAC, and this is new.

Student B:

During UAC we studied all is theory, but in now Monash, we study more... We have more case studies so we can understand the concept better.

Lecturer A:

Okay. So, for Hu Yang, you believe by being introduced to these case studies, you understand it better, is it versus looking at the theory version?

Student B:

Because during UAC, we don't have the case study, so everything you know is about the theory. So now in Monash, we have learned about the theories and also the case study. So we can understand better.

Lecturer A:

Okay. Can you... I don't know, this is a bit impromptu. So can you give me a specific example? Whatever you can remember. It doesn't have to be perfect, just in your own words, you just say, yeah.

Student B:

I have a little bit for my discovery so I can just forward it.

Lecturer B:

Take your time no worries.

Lecturer A:

In the meantime, yes Student A what about you? Anything similar or anything different?

Student A:

Yeah, quite the same with Hu Yang but for me, another thing is during UAC, the teachers just teach everything in the textbook and highlight the points ask us to memorize this, memorize that, then they will give us a bunch of past years so we can memorize those important stuff, but they don't really make us think before giving us the answers. They just give us the answers, they don't care if... But for uni, sometimes when I go to email the lecturers, they'll just give me hints first. But if I really don't understand, maybe I'm stuck there, they don't really give direct answers, you need to think first.

Lecturer A:

And, what do you like? And it's okay to not like, and I know both of us are lecturers here. Dr. Lecturer B and myself, if you don't like us, you can tell us. There's nothing affect anything. What do you like?

Student A:

I like the approach here, really [inaudible 00:06:10].

Lecturer A:

So you prefer, so far you're okay?

Student A:

Yeah.

Lecturer A:

Why?

Student A:

They don't treat us like robots, they want us to think and they care about thoughts.

Lecturer A:

Yeah, okay so-

Student A:

[inaudible 00:52:28].

Lecturer A:

Sorry, go on. I missed you a bit.

Student A:

Yeah. That's all.

Lecturer A:

Okay. Thank you for that. So if I can summarize how you feel, and if I understand correctly, you do like this invitation to ask questions or you like to be challenged, and you feel that this is more enjoyable for you. You like it more now.

Student A:

Yeah, because if they gave me the answers like in UAC, maybe I would think the answers are too easy to get, I just ask and the answers will come to me. But if I think I put my effort to think, then maybe I can remember the answers more longer. I don't need to always memorize or something.

Lecturer A:

Yeah, that's a good-

Student A:

I go over the books a lot of times only I can get into my mind. It's because I think very long, so I came to this thought or something. So yeah.

Lecturer A:

I think that's a very good point, Student A, I think that's a very good point. So thank you for sharing. And again, there's no right or wrong, we actually just want to get to know how you feel and what you are thinking about it.

Student A:

Well, I'm not really good at explaining things through.

Lecturer B:

Don't worry.

Lecturer A:

No, I don't think you should fear Student A. I think you've been doing quite a good job so far. We do understand you, what you're saying. It's just that for me as a moderator, I always... I like to summarize after each person's comments, and I've been doing that for every everyone else's as well, it's not just you. So I really I don't know why you say that you're not good at explaining things, because so far you've been doing quite a very good job. So yeah, don't worry too much about that.

I think it's just the computer, we're talking via the computer screen. And I think we didn't mention this earlier or we forgot so, we are running these sessions, not only with the two of you, but we are doing this with other students as well, across the four years program as well. So that's why the questions, so far it's been quite generic or quite general, but yeah. Hu Yang, have you found an example to share?

Student B:

Yeah. I've studied drug metabolisms and we know that some drugs is being metabolized by MRP through and [inaudible 00:55:20]. So our lecturer was sometimes give us some case, like Dubin-Johnson syndrome, which the patient has deficiency of MRP too, so that the lecturer will give us a question like for a patient with Dubin-Johnson symptoms, how are these drugs being metabolized, will it be metabolized more or less. So the answer is definitely less because you have the deficiency in these enzymes. These some kinds of scenarios that the lecturers gave us during the intellect lectures.

Lecturer A:

Okay. So in total you're also saying then that in UAC, if we were to teach you, I don't know something about Biology or something about Chemistry, the examples that you had in UAC were not very real life or, they are more theoretical, is that correct, or?

Student B:

Yes. Maybe they would then give us this Dubin-Johnson syndrome, they would just tell us that, Oh, we have this RP2 that metabolize the drugs.

Lecturer A:

I see. Okay. Thank you. We just wanted to find out bit more. Okay. Thanks Lecturer B, over to you.

Lecturer B:

Okay. So next question I have is a bit of a difficult one let's say. So in your course you have many different types of units, right? Some, I think you've done a lot of science units in a sense, how body works is physiology and anatomy, how medicines work is chemistry, but then you also have professional practice, which is a bit more practice-based type of things. Do you feel that this method of the discovery lecture or that, is it useful for all types of subjects, or do you think is more useful for some more than others? Just try and give me your thoughts. It is a bit of a challenging question. If you don't understand what the question is, I could explain. Sorry. Do you get what I'm asking actually?

Student A:

Well, professional practice, I think some of the topics are more to the Australian context, so I'm not sure if I'm going to go there, but yeah. If I'm not maybe teach more about the Malaysian context and something like that.

Lecturer B:

Okay. So you feel that for professional practice it's [inaudible 00:58:18] nicer in that context?

Student A:

Like last semester there's, I don't know, maybe about 10 topics, but I think two or three is more to Australian contexts, and some of those things maybe if we re-read practice here in Malaysia, it's not really... Yeah, I think it's a very good thing to know about the system, but in Malaysia we're not using it.

Lecturer B:

Okay, well fair enough. It is true that if you practice in Malaysia, you may not use some of the things that are learned specifically for the Australia context. But yeah. Thanks for that. Hu Yang do you have any thoughts?

Student B:

Sorry. I didn't really get your questions.

Lecturer B:

I dealt the question was, so you have some basic science things, okay, you're learning Chemistry, you're also learning Anatomy Physiology. And you also have some units that are practice based, like my professional practice. Do you think that this way of learning is useful? So when I say this way of learning, I mean the discovery lecture workshop, do you think this method is useful for all types of subjects?

Student B:

Yeah. I think it is quite useful. How the Medicine works and how the body works. During workshop, we have case studies so that's quite good, and during for professional practice, we have activities like the lecturers will ask us to speak, so to change our communication skills. So I think, although it's the STEM workshops, but they introduced it in different ways, for professional practice, our lecturer would then ask us to do case studies. They would ask us to have discussions. Although it's the STEM workshop that is quite different also.

Lecturer B:

So generally you feel that it can be adapted to most types of subjects that it's not... Okay, I think thanks for that, that's fine.

Student B:

Yeah.

Lecturer B:

So basically, there is a slight difference. Although it's still workshop, but they just change it a little to adapt to how Medicines work, or if it's personal practice, they change it a little. All right. Fair enough. Okay, good. Wait, let me see, is there... Okay. I think there's only not that many questions left to ask.

So, you have friends in other universities doing perhaps a more normal... You have friends in other units, how does their uni work in terms of lectures and all? Is it quite similar to what you have all quite different?

Student A:

I have a friend studying hotel management in UCSI Cheras campus, I think if we compare our uni to hers, our uni is more... The system is more organized because they sometimes run live lectures, but only the people who are in Sarawak in the lecture hall, and my friend needs to do something like zoom and the lecturers sometimes go around and she can't hear or see what they're teaching. Sometimes she tries to tell them, they don't really care, they don't know how to solve the problem. So, I think that affects her and... But one thing is, I don't know why the school doesn't let the lecturers give us their personal numbers, because I see a lot of schools doing that. And it's like, the lecturer opens a group chat and let's everyone inside. So it's more convenient because we don't need to always go through email. There's WhatsApp now.

Lecturer B:

Okay. You would prefer that type of communication. Okay. But we also have forums and things like that. Do you think the anonymous forums are useful for asking questions and things like that?

Student A:

Yeah, because the forum doesn't show my name. So it gives me more courage to ask questions. I don't need to think if my questions are stupid or something.

Lecturer B:

Do you all prefer email or forums to ask questions?

Student A:

For me it's email, because the lecturers respond more faster.

Lecturer B:

For Hu Yang, how about you?

Student B:

I prefer forums lectures they encourage us to ask questions, so we have forums. So I do ask their forums, but sometimes the lecturer didn't really answer some of the questions. Maybe they pass it. I don't know. Yeah.

Lecturer B:

Okay. But I guess if you all could choose, you probably prefer to ask in talking like you could directly ask, is that your preferred method?

Student B:

Yeah.

Student A:

But in talking, I mean, like talking with less people. Yeah. Maybe like this, if it's asking in front of a lecture hall or something, I don't prefer that.

Lecturer B:

Oh, okay. So yeah, actually, all right. Okay. So I'm going to ask another big general question. So in terms of this, the way that education is done in Monash overall, do you think that it helps you to... How does it help you or how does it... What benefit do you think this whole way of doing things in Monash brings to you?

Lecturer A:

Maybe, if I can just paraphrase, okay, this is another way to ask the question. First, can I... So, obviously throughout the course of the conversation that we've had so far, and what you've also do that, what you have shared, then the reality is that what you've been experiencing so far in your uni is different than what the one you've had so far in the past, is that correct?

Student B:

Yeah.

Lecturer A:

Okay. Do you notice that personally has this style of learning that you are going through in uni, has it helped you to grow in any way or has it helped to improve or make you aware of some things that you need to work on? I mean, the reality is that this is still your first year of uni. If I give you an example, so Student A did share earlier that UAC or in a previous schooling experience, she didn't have much opportunity, or wasn't asked to talk a lot or to do public speaking, but she realizes that so far in this so far in the past few months in uni this year, there's a lot of activities that requires her to speak even more and even so in an online environment.

So she recognized that, it's something good to pick up, something good that she should maybe work on a bit more. And so she sees this as opportunities to develop that. Is that correct Student A?

Student A:

Yeah.

Lecturer A:

Yeah, okay. So, we were just wondering, was there anything else, and it can be your time management skills, it can be your sense of being more responsible or it could be anything. And if you say... Even if you feel like there's nothing so far, then that's also okay. So maybe for Student A, was there anything else apart from your communication skills so far that you found this style or this, that in your uni so far, that it has helped you to develop or to improve?

Student A:

I need time to think first.

Lecturer A:

Yeah, it's okay. Hu Yang anything?

Student B:

Team working skills.

Student A:

Team working skills is it?

Lecturer A:

Because during high schools [inaudible 01:08:00].

Lecturer B:

A lot louder please.

Student B:

So during high school, the tasks for our teamwork, we were grouped with our best friends. So everything is smooth and fine, but now we work with strangers, because we didn't really know each other. Yeah. So there's something new to me to work with someone you didn't really know about. And also the public speaking skill that Student A just now mentions. Yeah.

Lecturer A:

So, Hu Yang, you think this whole aspect of learning to work with strangers, is it good or bad? Is it useful or is it like a not necessary. Do you think it is...

Student B:

I think it's useful.

Lecturer A:

Why?

Student B:

Because we can't really work with everyone we know.

PART 3 OF 4 ENDS [01:09:04]

Student B:

Because we can't really work with everyone we know in the future.

Lecturer A:

Okay. So you see the way your syllabus has been designed, where it forces you to work with random people, this is a useful thing, a useful skill to develop, and it will definitely help you in the future, so you see this as a valuable thing? Is it?

Lecturer B:

Oh, she's... I think she froze a little, is it?

Lecturer A:

Hello, [Huiyan 01:09:38] can you hear me?

Student B:

Yes.

Lecturer A:

Okay. Did you hear what I said just now?

Student B:

Sorry, no.

Lecturer A:

Okay. That's okay. That's okay. I will repeat myself. So just to summarize, you feel that because of the way our syllabus is designed, it makes you work with strangers and you think this is a useful skill to develop for the future.

Student B:

Yeah.

Lecturer A:

Okay. Thank you. Thanks. [Student A 00:01:10:12], anything else that you want to add?

Student A:

Almost with the same with Huiyan, but one thing I find interesting is during how the body works, we have one individual presentation, plus we need to write an essay and we need to research on a disease and the medication to treat it. I don't know but if hopefully I finish the course, I'm also thinking of continuing doing masters or something. Yeah. I don't really know if what I'm doing last semester is counted as research, but at least during my final year, I can do something like that. I don't know. I like going to the library website and searching for random articles. During that time, I was focused on that particular disease, but I like doing that kind of stuff.

Lecturer A:

I see. Okay. That's a good point. Thank you for sharing. So you like to be given questions and we give you time and you go and search for the answers to these questions. You like to do that?

Student A:

Yeah.

Lecturer A:

Okay. Okay. No, that's good. And again, we all have different learning styles or different, different liking for some, for different type of assessments. So personally for us here, Dr. Lecturer B [Doctor Lecturer B 00:03:08] and myself, we just want to find out a bit more about how you all feel and how do you all think. Yeah. Yeah. Okay. Lecturer B, anything else?

Lecturer B:

The final question is again, a very similar kind of question in a sense that... so from what you've gone through this year... You can take a bit of time to think about the answer. Do you have anything you feel that we can improve, at the top of your head, just from what you see? Yeah. It's a slightly difficult question and you can take a minute or so to just think through. From what you've experienced... Perhaps a good way to start is to think through the parts that you don't like, or you don't prefer or what and how we could change those to something that's useful, or maybe certain things you feel are not implemented very well. How could we do that better?

Lecturer A:

Okay. Student A said just now that you wish that we will give you more past year papers, right?

Student A:

Yeah.

Lecturer A:

Why? Why would you like that?

Student A:

I can focus on what will-

Lecturer A:

What will come out?

Student A:

Yeah.

Lecturer A:

And if I tell you, "No, we don't do that in uni." We're not saying yes or no yet, but I'm just saying, let's say if I tell you no, and we don't do that at uni, what would you respond, or how would you feel? It's okay, too bad, [inaudible 01:14:00] right?

Student A:

Yeah. But I hope you all can suggest how I can do better.

Lecturer A:

Yeah, sure. Okay. So if we do give you some kind of feedback, because obviously feedback or some corrections where you did wrong, that's always the important in teaching. So if we just give you a general one, and that's all we can do, is that sufficient? Is that okay for you?

Student A:

For me, no, but I know you don't have a lot of time to do feedback for all of us, or say individual feedback, if you had other students. But sometimes, maybe the feedback.... maybe the part that am weak is not... Not majority of the people are we in that part. So I don't really know. I don't realize that I didn't get that part. Yeah.

Lecturer A:

Mm-hmm(affirmative) [inaudible 01:15:24] So [inaudible 01:15:27] yeah. Anything else? Yeah.

Lecturer B:

Who, has any thoughts on what could be improved?

Student B:

The past sections? Because we have only four [inaudible 01:15:43] and each [inaudible 01:15:46] can only have about 16 of us to join. Yeah. But for me, I think if in real life face-to-face that limited numbers is important, but we are Jews. Actually. I think we can have more students to join the past sections because the students don't really talk.

Lecturer B:

Yeah. Okay. So, Okay one thing is to improve paths in terms of capacity, basically.

Student B:

Yeah.

Lecturer B:

Okay. All right.

Lecturer A:

Have you had much interaction with your seniors with any platform in any way?

Student A:

Yeah. I met two of them, but I don't know them before that, it's during the campus tour.

Lecturer B:

Okay, you mean life, you mean in... the next trip when you came to campus?

Student A:

Yeah. Before the [inaudible 01:17:01]

Lecturer A:

And, Huiyan, what about you?

Student B:

I know the seniors because I've mentioned I'm in the [inaudible 01:17:13] community. So I know some of the seniors, like female representatives of different school from a CML represent. Yeah. So quite some that.

Lecturer A:

Have you all had a chance or any opportunity to ask them about... Ask them for any help or ask them for past year papers. Also, if they have any past year papers or ask them about how, how to navigate through the Model or ask them about anything. Have you all had that?

Student A:

Not really.

Lecturer A:

Not really, Yeah, Okay.

Lecturer B:

What's the... if you have to... If you each of you have to say it, what would be the biggest challenge you face so far in this one year? In terms of city life?

Student A:

Finding friends?

Lecturer B:

Finding friends, okay.

Student B:

Though some of them.

Lecturer B:

Okay. So the social aspect of things is really important to you. You feel that's the biggest thing that's lacking in this whole online thing. All right, all right. Do you think there was a way we could do that better? How could we have improved that for you? I mean, let's say we had to do it online that let's say we couldn't go live because you know, COVID is something we couldn't control, but with an online thing, was there a way we could have done it better or?

Student B:

Supposing everyone to turn on their videos.

Lecturer B:

Okay, fair enough. But I guess you mean doing workshops in breakout rooms in this small little things? Not in the whole lecture hall.

Student B:

Yeah.

Lecturer B:

Okay, you think videos could have helped. Okay. Any other thing? I don't know any social media platform, I'm just throwing some thoughts, but

Student B:

I think not much because [inaudible 01:19:27] would break us into different backup roles, the students will remain silence, so, yeah.

Student A:

Can't really help.

Lecturer B:

So the video thing may help. You feel that having... If you can see face to face, you probably will talk more. Well, I guess if you went far with here, already, you can see that because we can see each other then there is as a higher tendency to talk also, could be true. Yeah.

Student A:

Yeah. I found something in the Monash website. It's the monash.edu.my that thing yeah. Well, I forgot the name. I can try and find it and email it to you guys. It's a bit like a social media platform or something, but it's only for Monash students. I think the Australian campuses is using that, because when I, when I look into that thing, it's mostly for those Australian students, but I think we can try to encourage students to use that. I don't know if it will help, but yeah, we can try.

Lecturer B:

Okay. That may help. Well Yeah, I guess both of you are really looking forward to coming back on campus soon. Yes, yeah. And I guess this is something we'll... hopefully the situation will improve and next year you all will start year one as your real campus university year. Okay. [Way Ching 01:21:09], do you have any other questions to ask?

Lecturer A:

No. So yeah. Similarly for us, as well as your lecturers, we also do miss the physical interaction with the students. We also find it a challenge to communicate via the computer screen as well. But we really do thank you for your patience and also for your feedback. Moreso when the lecturers asked you. I know both of us haven't really taught much to you in year ones, but then we on behalf of our colleagues as well. We do thank you as well for your patience and for your cooperation. So you will see more of us in the later years of your program as we are a bit more involved in the teaching part, at least in the later part of your undergraduate program. Just to summarize some of the themes that we've talked about today, as I said earlier this discussion that we have for this focus group that we have right now, it's one of a few that we're doing with all the different students.

And is also event... Is just to find out how students are feeling towards two things. Number one, this whole aspect of online learning, and number two, this whole aspect of the uniqueness or the design of the Monash pharmacy program. If I can just summarize some of the responses from the two of you all so far, generally you are okay with this whole online learning, but obviously you prefer a more physical interaction because you miss the whole aspect of making friends. And that to you is very important to help you in your individual university experience. In terms of the design of the syllabus, so far again, you do like the getting the discovery materials before coming for classes, it gives an opportunity for you to read, think about it, meditate on it. If you need to, and then ask questions and then with that, you also do enjoy the whole opportunity to ask questions with your lecturers and with your friends. Even though sometimes that especially your friends, that doesn't happen very well because of the limitations of group discussions now.

But again, you do enjoy the aspect of asking questions. And then with that, you will realize that communication is something that we put some emphasis on here in your undergraduate program. And you'd like see this as a platform to develop this further. And y'all think that this is obviously useful for your future career and also getting the discovery materials a bit handy. Also it

gives you opportunity to ask more questions about it. I think this was more what Student A mentioned about. She does enjoy being, given a question and being given access to resources to get to explore the answers to questions. So you like to solve complex questions. If I can summarize that. Is that a fair summary?

Student A:

Yeah.

Lecturer A:

And anything that I said that wasn't correct, or that you feel that you want to add anything else or not?

Student B:

No.

Student A:

One thing I wanted to add, but I don't know where this fits in. During high school, my teachers are like, if you don't get this score, you're not good enough. You need to do better. But I think in Monash, the lectures a very supportive. Very, I don't know. I sometimes I think I'm not doing very well, but they are like always well done. Yeah. They're encouraging and they're not like in high school its... I feel like they're discouraging us.

Lecturer A:

We don't have time to, to beyond that.

Student A:

They want to keep me weak, but, yeah.

Lecturer A:

I think for us in university, we don't really approach like a [roatan 01:25:39] method, but you all have to take your responsibility for your own studies and which so far from the hearing, the two of you, I think you all do okay and you're doing... Trying to do your best for us. We cannot [inaudible 01:25:56] we just say, and then it's up to you whether you want to listen, or if you choose to do your own thing as well. So that's university. But I know that's good. I'm glad that you feel that way. And again, some students actually don't like it. Some students prefer the Roatan way as well. So for whatever reason, some students will perform very well if the roatan is there. So, like I say again, it's different student, personality, different liking with different preference for study. So hopefully I know it's your first year uni, hopefully that it will be a good and fruitful and you will find it a rewarding time for yourself. And when you go to your undergraduate program in Monash pharmacy. Do you have any questions for us? You can ask us anything.

Student B:

Not really.

Lecturer A:

Not really. Okay. So Student A any questions or

Student A:

Not now, but it I can always see both of you if I have.

Lecturer A:

Yeah. You have our email addresses. We are currently on campus. The lectures have been allowed to come back to campus already as well. So, Lecturer B, do you have anything else to add or?

Lecturer B:

No, not really. Just if you do drop by two, if you can collect your coffee bean, I'll show any time, if not should be next Sem, if not, so-

Lecturer A:

Well, students are allowed to come back to campus already and Lecturer B you live in Puchong, right?

Lecturer B:

Yeah. Actually you can come. If you'd like that. If not, I will... because sem two year one, I don't know, year two sem one I'm teaching one of the units. I'm using one of the units. So I will see you then. So that could be a good enough opportunity if it's a live lecture then yeah. You can just come down. Yeah.

Lecturer A:

So Student A you have anything else, do you have any trips back to Peninsula Malaysia plan or?

Student A:

Yes. And I'm in Saba and I don't know, the COVID cases are raising here, so... And they need to do the sub-tests and I don't want to do that because I don't want to cut my nose... Yeah.

Lecturer A:

So Student A, do you know of any other Monash pharmacy students that are living close to you in Saba?

Student A:

I know one, but I'm not really close to her. So, we stay in the same city [inaudible 00:19:49].

Lecturer A:

These other student is in year one as well, is it?

Student A:

Yeah. And I know a few who are not in pharmacy course, but also in year one.

Lecturer A:

Okay, [crosstalk 01:29:04]. Okay that's good. So I think I only... Because I think for Huiyan, she's close to the campus. So it's a bit okay. And I'm sure there's some friends around there. And just from y'all for you, cause you're obviously in a different state and sometimes you may feel a bit lonely or you just don't have that... you say that you honestly feel, I just want to make sure that at least that you know, of some people who are close to you, that you can just ask, or I do know of a few of your seniors who are also living in Saba, who are in Saba right now. So if ever you feel that you want to connect with them, I can help introduce you to them as well, but you just have to let me know.

Student A:

Okay.

Lecturer A:

Yeah, Okay.

Student A:

Thank you.

Lecturer A:

No problem. So yeah, just anything to support your education experience. I don't have anything else.

Lecturer B:

Neither do I.

Lecturer A:

Yeah. So just email us, you have our emails, let us know whenever you're on campus to collect your 50 ringette coffee bean gift card. And until then look after yourself, take care, enjoy the holidays. Look after yourself as well. And then when studies next semester start again, all the best with the remaining of your studies. Okay. [crosstalk 01:30:32] All right. I will stop the recording now.

PART 4 OF 4 ENDS [01:30:35]

Deidentified FG6Y3 - Oct 2021

Lecturer A:

... started. Okay. I will just quickly start off your first question, and I think this is a very interesting question to start off our discussion this morning. And I have started the recording. Yes, I can confirm that the recording is started. As I mentioned earlier, we did run a survey so we got the class to fill it complete. And we noticed a real interesting trend which was similar across the different cohorts that when students move from years to two years three, there was actually a drop in their overall feelings of attitudes and their preferences towards the style of learning here as part of your program. I think, Dr. Lecturer B just has a slide to share this or to just illustrate this just so that if you don't understand.

Lecturer A:

So if you recall, we shared a survey, we got you all to fill some of these questions that look like what is here that's on the chat screen now. So essentially, what these questions were actually designed, they were actually try to find out students attitudes and preferences towards this style of learning. And so, what we noticed was that there was a general drop from years two to years three. And so, we were just curious from your end, why do you think that was so or what do you think about it? Anyone to-

Student C (Focus Group 3):

Okay. I'll-

Lecturer A:

Okay. Student C. Yeah.

Student C (Focus Group 3):

I think the main reason is students are required to self-study more in year three compared to year two. And the difference is really huge as in I know that Monash has been promoting self-studying since year one, but then the difference is really huge. Is like for you three, the units they'll be throwing you a lot of external links and study materials. And then the discovery materials, they are not even complete. And we'll have to click on the links and read ourselves. And then the links are like super long. And then the videos, we'll have to watch all the videos and stuff, which is very confusing. And we don't know which materials to focus on and which points to emphasize on.

Student C (Focus Group 3):

So I think mainly it's the self-study part. And then also on some units, I feel like even the lectures are also quite overwhelmed and not that organized. So maybe even the students are also confused by the lecturers announcements and the general structure of the course. Yeah. That's it.

Lecturer A:

Thanks, Student C. Then thanks for the opening comment. Anything similar or different to add, Student B?

Student B (Focus Group 3):

Similar to Student C's comment, I want to add that imagine just like the entire unit is just summarized behind two lines, and then you're expected to read more than you're given. And also, one of the things that okay, I'm talking about what specifically affected me, so one of the things that some references are actually, not contradicting but they give us conflicting information.

Student B (Focus Group 3):

So if you go to this reference, you might find this and we're not getting enough clarification on this. I don't think we had this issue back in year two, we rarely had any come any conflicting information. I think it was fine. But in year three, it was a little bit chaotic. Especially one of the units was just simply unorganized. I'm not going to mention which unit for now.

Lecturer A:

Thank you. Thanks, Student B. And I guess the word that you used, chaotic, it's quite a big word. But I guess students have had opportunities to also express their thoughts through the SSLC and through the difference format in a formal structure that is more linked to your learning activities. Our intention here really is just to ask more about the overall design and the fact that you'll have to do a pre class activity, and then you come for class and you do learning steps. All right. And I believe you'll have shared as well your thoughts with the relevant units and the UCs as well. So thanks, Student B. And, Student A anything else to add, anything similar or anything different?

Student A (Focus Group 3):

I agree with both of them because year three we have too many information to take in, too many links. Actually, extracting information to make your own notes, I think it takes more time for me to do so rather than actually have the time to absorb the information as compared to year two where the links are quite direct so we say this like is the main thing. So if you want you can read this optional activities, but actually they say to read all but they didn't mention which is supported and which part is not. Yeah.

Lecturer A:

Thanks, Student A. So thanks to the three of you also first that opening question. And I guess we'll link to that as well. So to that question, similarly, we found also, generally students also felt had a more higher, had a higher scoring towards their workshop sessions than compared to their lectures. And so, now this is across all the years, so not just referencing years to two years to years three, but across the years we found that students scored a bit much higher, the scoring was a bit higher or more positive towards their sessions in that, towards their workshop sessions in contrast to their lectures. Any reasons why that was so and what do you think about this?

Lecturer B:

Jin, just to give you some context, we asked more or less the same questions both for lectures and also for workshops, but it's the same set of questions, we just put you into different settings. And the scores were generally higher for the workshops. So just for context.

Student C (Focus Group 3):

Okay. I think I know the reason. I think it's probably because usually for our unit will have two lectures for the acute care unit. So the acute care units will have IL-1 and IL-2. And the IL-1 is mostly just discussing the answers for the [inaudible 00:07:06] which is basically the same questions, seven questions. And other than that, we will not be discussing any other stuffs from the discovery topic, sorry, for IL-2, we will be discussing more. But then I just feel like one hour is not enough to cover everything in the discovery, especially the external links and all the references that we are required to go to. So I think this is the main reason mostly on the first IL, where we only discuss the questions and then we are asked to study on ourselves and look for more information on ourselves. Yeah.

Lecturer A:

Thank you. And, Student B, anything else to add?

Student B (Focus Group 3):

Can you just, sir, repeat the question so I can just gather my thoughts again?

Lecturer A:

Okay. Sure. I'll just repeat the question again if I wasn't clear earlier. So from our survey results as well, we found that students scored a bit more positive results towards their sessions in their workshop in contrast to the other times, their lectures. And so, we were just curious as to why that was so? And we are now moving away from the differences between years two and years three, and we're taking more of a general approach across all four years.

Student B (Focus Group 3):

Well, I honestly think it's the most useful session for me because from my perspective, I'm most of the time not really focused on the lecture, I'm like, I just want to get it done because I don't find myself benefiting a lot from it. However, in the workshop there is hands on practice, I am actually applying what I've taken in the discovery or in the lectures and applying it in the workshop. So I think there is a more practical approach in the workshop rather than lecture

Lecturer A:

Thank you. And, Student A, anything similar or different to add?

Student A (Focus Group 3):

Yeah. I would say that the most information come from discovery, I always like to transition but workshop is actually where we apply and actually this task because there can be some discrepancies that we don't catch them you're just reading it. But when we are applying we notice what is the issue and we can understand the topic better because it's a group discussion hence our... Personally I also prefer workshop more than that lectures, and lecture time isn't really enough to explain all the concepts that, Student C said.

Lecturer A:

Thanks. Thank you. Thanks the three of you. Dr. Lecturer B, anything else to add?

Lecturer B:

No, no. That was the other main things for this part of it. I will stop sharing because the rest of the questions don't need this.

Lecturer A:

Sure. Thank you. So we'll move on to the next question now. So thanks for the start for the first question. The next question is about this whole area of the logistics of learning. So the reality is that we've spent already quite some time learning virtually already. Student B has dropped out, but I think we'll just continue until he's joined back. Yeah.

Lecturer A:

So we've spent quite a bit of time learning virtually already. Was there any aspect or anything that when you're doing or learning virtually you all found that this was actually just really hard to do this virtually so far? So this is up to anything that you've experienced since the beginning until today, anything you felt that was hard or was challenging to learn virtually?

Student C (Focus Group 3):

Actually, I think there are pros and cons to both online and physical classes. So maybe some people might think that online classes are more flexible, they can just wake up and then just switch on their camera, switch on their computers and then go to the classes. But for me, I would prefer to do physical classes because I don't know but I feel like I'm staring at a real person talking rather than your computer screen is more interactive. And it gives a more realistic view and then you are more focused. Because usually when I'm on online classes, I'll get distracted and then I'll go on to social media, or maybe just google something that I don't understand. And then later, I'll not focus on the classes because the lecturer speaks too fast.

Student C (Focus Group 3):

But usually in physical classes, I can pay full attention because I usually switch off or turn my phone to airplane mode and then just focus on the class instead of getting distracted easily. And then at home because online is usually at home, so I'll also get distracted by my family members if they do something too noisy and distract my online class. Yeah. Well, I think that's the difference.

Lecturer A:

Okay. So thanks, Student C, you shared some differences. So now I wanted you if you was there, could you name anything that was specific that you were learning up to this point and felt that oh, this was just hard to be learning virtually?

Student C (Focus Group 3):

I think it's not very hard but there are quite a few challenges. Firstly, the distraction that I just mentioned and the Wi-Fi problem. If I'm doing a presentation, I might drop out very easily because my Wi-Fi is not very good. And I think it's mainly the connection and distraction issue. Yeah.

Lecturer A:

Okay. And, Student A?

Student A (Focus Group 3):

For me the toughest part of virtual since the beginning is actually asking questions because usually in lectures, we can listen to everyone's questions and then there's no repetitive questions. And then other lectures will be able to pinpoint where students are confused. But because we're doing it online, there are people just ask random questions without thinking it through. So we have a lot of small, small questions but we kind of lost track of it. And I feel that verbal communication is easier to follow than written communication. And it explains concept, especially concept much better. That's my main challenge.

Student A (Focus Group 3):

And the second one is Wi-Fi issues. As well as some technical issues in Zoom sometimes that if the lecturer speak too fast and we can't really trace back, and we can't really remember which part she stopped at, is really hard to just stop the lecturer at that spot and just chip in.

Lecturer A:

Thank you. And, Student B, what about yourself? I don't know you heard the question but I'll just repeat the question again. What have you found to be difficult to be learning virtually?

Student B (Focus Group 3):

Something that's evident, my connection lags a lot especially when I moved here. For some reason, the entire city where I'm living in the internet is horrible everywhere. I tried everything, it's horrible. I don't know why but it's just the way it is for some reason. So internet is a very horrible problem.

Student B (Focus Group 3):

Also, collusion is a problem as well. I noticed, without mentioning names, of course, I noticed some people are actually colluding with each other in assessments, which are supposed to be individual. I've communicated with the some of the teachers, some of the lectures about this but nothing they can do. So we're just dealing with it.

Lecturer A:

Okay. All right. Thanks, Student B. Let me just send out a specific question, what do you all think about learning the use of the different medical devices in your lab sessions, how has those virtual learning experience been? Anyone?

Student C (Focus Group 3):

We haven't even touched one.

Lecturer A:

Okay.

Student C (Focus Group 3):

Yeah. I can't say that experience because I've never touched one, we only get to buy our own kitchen appliances during year two to do the labs. So unless some students have already done it before giving them a few times, but I think none of us has ever experienced this. And it's very disappointing and very sad because a lot of students look forward to doing the practicals. But I guess it's a global issue and nobody can control this. Yeah.

Lecturer A:

Thank you. Anything similar or different from the other two students?

Student B (Focus Group 3):

I honestly expected the more from the, what do they call it? I honestly expected more from this hands on activity, I was anticipating but then nothing we can do about it and it was gone. Yeah.

Student A (Focus Group 3):

To add on, we're just hoping for a replacement session if possible for the labs, especially placements because I feel like they are very important skills for pharmacists. And if we don't know them, it is a very dangerous to practice so to say.

Lecturer A:

Sure. And one of you disappeared, but that's okay. Thank you for sharing those thoughts. And we'll move on to the next question now. So one of the themes that came out from the earlier focus groups or the earlier discussions with different batch of students was yes, even though there has been some challenges with the virtual learning, but there has been also some positives. And one of the positives as I think one of you mentioned earlier about is this whole idea of flexibility and the fact that you're just able to just rock a few minutes before class start and that's all fine.

Lecturer A:

Verses, I know before some of you all may have to travel for up to one and a half to two hours just to get to campus. So I guess our question to you this morning to see even if you follow this question is that this whole idea of flexibility, how important it is to you to the overall learning experience?

Student C (Focus Group 3):

I think it's not really important but it's more too comfortable. Because I don't know the other students, maybe they drive to uni or maybe they have to catch a bus or something to uni, but usually I walk to uni because I stay nearby in the hostel. So I think it's a good chance for me to exercise. But if everything is online, I'll just wake up, walk to the study room from my bedroom and then switch on the computer, and then it's very comfortable, very easy, very efficient, there's nothing to complain about, everything is very simple.

Student C (Focus Group 3):

And then I think it makes life easier and then we become more lazy. But I think if you do it every day, you'll feel your study life is super bland and then you'll get tired of it, especially after doing this for two years. So I think it's not really that good. But for some people, they may prefer it because it's easier and they don't have to drive, and they don't have to go through the traffic jams and stuff. Yeah.

Lecturer A:

Thank you. Thanks, Student C. Student B, anything to add?

Student B (Focus Group 3):

I honestly don't find the flexibility as being an advantage just like any way I just attend my classes early, I don't have a problem with attending my classes early honestly, it's just depend on time. It's the flexibility part for me is that I can attend from anywhere. So sometimes I can just go in my, sorry, it can be somewhere else like on my car or something and attend the class or do my item IL in class, it's flexible in this part.

Lecturer A:

Thank you. Thanks. Student A, anything else to add?

Student A (Focus Group 3):

For me flexibility of time is really important because I can do much more so because I actually very near the campus however, it still takes time to commute like if I have special events going on at certain areas. However, not everything is transferred online, so I could actually attend two to three events back to back. The downside would be to make me lose focus and I don't feel like I'm fully participating in the event. But this flexibility is quite useful because I could help out with my family work and also other stuff.

Lecturer A:

Thank you. Student A, you mentioned this thing flexibility of time earlier your response can you just explain to me a little bit more, what do you mean by that?

Student A (Focus Group 3):

The flexibility of time as in because usually we would have to go in a few more 10 to 20 minutes before class and including the commitment time. However, if it's online you could just go in anytime

you want. And the good thing is that because of online, we would have like better recording quality. So even if we miss it, we still are able to play back. I would say that that gives a bit more flexibility in terms of when to watch your ILs and revise your CTLs.

Lecturer A:

I see. All right. Thanks for clarifying that. Okay. And then I guess the next part of this flexibility as well this is similar, is sort of link to the next question. I'm sure you all have received some newsletter, some discussion or some emails from the campus central. And also some, you've been following the news as well, there's some discussion thread of trying to reactivate campus or to try to slowly transition to some face-to-face sessions and activities. Obviously nothing is concrete, so everything that is a part of your classes or your individual units please refer to your individual OSCE for how you'll progress in the future.

Lecturer A:

But just right now we just curious about your personal feelings, how do you feel about this whole idea of coming back to campus post COVID? Actually not really post COVID, we're still in the middle of the pandemic but what are your feelings towards about this whole discussion of this possibility of coming back to campus?

Student B (Focus Group 3):

I want to go back to campus.

Lecturer A:

Sorry, Student B, you want or you don't want?

Student B (Focus Group 3):

I want to go back to campus, I can't just stay out of the loft like this.

Lecturer A:

Okay. Thanks, Student B. Any feelings of nervousness or anxiety within or you're excited, you just can't wait to come back? Student B?

Student B (Focus Group 3):

I thought you'd question someone else. Honestly, I want to come back because what am I doing here? That's the point. I want to come back as fast as possible so I can just start doing something in the campus.

Lecturer A:

All right. Thank you. Thanks, Student B. And what about the other two? Maybe, Student A, any thoughts?

Student A (Focus Group 3):

Yeah. I would like to go back to campus as well because we have been losing the physical touch for some time and I feel like the communication and the human interaction is also a very important part of being a pharmacist, especially the working experience also. And secondly, I feel like most people will look forward to campus re-opening up but we must make sure that there are no cases actually happening in Monash because that would be quite dangerous. Yeah.

Lecturer A:

Okay. Thanks, Student A. And, Student C, what about yourself?

Student C (Focus Group 3):

I'm actually super excited to go back because my house is not made for studying, it's super noisy and the Wi-Fi is not that good. And I don't even have a proper study table to study. So the school is made for us to learn, a conducive environment for us to study and learn, so I think the best places is to go back to the campus. But I'm also worried, at the same time I'm also worried because I often travel back from uni to my hometown, so I'm scared that if you there's some cases in the uni, then I will become high risk. And there's high risk on patients in my family, so I'm afraid that I'll waste them as well. But we'll see how that goes. But yeah, I'm super excited to go back to the uni. Yeah.

Student A (Focus Group 3):

Yeah. For the challenges, I'm just worried about the future OSCEs and exams, since we're so used to [inaudible 00:25:21] reference and we haven't even had physical APA before.

Student C (Focus Group 3):

[crosstalk 00:25:26].

Lecturer A:

Yup. Thank you. Yeah. Your academics are also excited to come back to campus as well when it is safe and possible because obviously, we also do enjoy the face-to-face interactions with you, all of you. We have a subsequent question about your OSCE later on but you definitely brought up an interesting point. If there's nothing... Lecturer B, did you have anything else to ask before we can move on or?

Lecturer B:

I'm not. Yes, you can. Continue to the next-

Lecturer A:

Okay. All right. Thank you. So the next question moving on, so you all had the privilege to start a face-to-face learning in your years one or the earliest part of your program. And then the unfortunate was that this, we went into a virtual setting because of COVID and all that. Just out of curiosity, has there been any concerns so far about your virtual education so far while in the middle of going through a pandemic? We asked this question very early last year, where we all went virtual. And so, there were some concerns, there was mentioned then already. But we're just curious as well whether if those concerns were still there, or do you feel that it has been resolved already? Or do you still feel that there are some issues that you're thinking about, your online education experience so far?

Lecturer A:

Because the reality is that I think when we asked you earlier, that was very much earlier during when we just started doing things virtually. And at that time, we were also learning as well. And I guess to that time, we've adapted, adjusted, we've got some feedbacks as well from the different environments or settings. But just curious, now that you've gone through three and a bit semesters really, whether those concerns are still there or whether they have improved or not? Yeah.

Student B (Focus Group 3):

I don't get the question. I don't really get it.

Lecturer A:

Okay. All right. So I'll just paraphrase the question in a different way to see whether if you understand this now. So some students shared that, no, they were quite used to... Okay. There's this whole idea of communication, they felt in a face-to-face environment, they were able to communicate with their lecturers much easier, and they were able to ask the questions and get the appropriate responses in a timely manner that the students appreciated. And then one of the concerns that came out when we went into a virtual format of learning was that students felt that oh, I don't know whether I can still ask or can they mix or still the response in the same amount of time, or whether I can just even have the opportunity to ask in a way that I like to us.

Lecturer A:

And I guess through that we then have obviously tried to encourage the usage of the forum, encouraged the use of speaking up during our Zoom, out during our ILs so during our workshops. And then I think some students did that, and we don't know whether if that has been satisfactory enough from the students point of view. But we haven't seen that crop up again in any form of evaluation so far. So we were just wondering, how students felt about that already? So that's just one example of the concerns that came up, we will be flipped over to a virtual learning environment. There's so many other examples I can think about as well. But does that help give you some clarity on what this question is about, Student B?

Student B (Focus Group 3):

Yeah. It makes sense.

Lecturer A:

Okay.

Student B (Focus Group 3):

Okay. So one of my seniors said, yesterday were on the event, they said that maybe not the You Tubes and the people who never had face-to-face interaction will know that, but as for us because we've seen both sides, it's much easier just to go to the lecture and just ask them directly, ask them at the end of the lecture. Instead of having some lectures just straight up close the Zoom meeting the moment they're done, they don't even give room for questions.

Student B (Focus Group 3):

For like the space we call the informal space of asking, you just have to ask it either in the Q&A session in the lecture, or alternatively have to ask it in the forum, which people are actually, some people, I don't know why they don't want to ask in the forum but it's just personal preference honestly. I myself, I'm not really a fan of asking in the forum, but I do it from time to time. But otherwise, I don't think it's a major issue that people should be concerned about anymore. We're just used to it, I guess.

Lecturer A:

I like that phrase that you used, Student B, the informal, the formal space of learning or the perceived when the classes finish, you can just come down to lecture and just quickly ask your question. And so, that's really good point. So thanks for sharing that, Student B. Any other things from the other students? And doesn't really have to be this whole questioning aspect, there could be

other aspects of your virtual learning as well, it was just one example that I used. But even if you've got any similar or different points to add as well, that's fine as well. So I'm just putting it out there.

Student C (Focus Group 3):

I think the other concerns are maybe like, Student A mentioned, maybe our practical because everything's online and we don't get to do practical. So maybe many of us will be very inexperienced when we go out for internships, placements or PRPs. And I think the other concern is interpersonal skills, what you just mentioned, because everything is online so some introverts or shy people may feel more comfortable doing it online, without switching on their cameras, and just having a chat. But it may become a problem if they go out and speak to future customers or future patients because even our OSCE is done online. And they can even read, actually they can read from the screen. So I think it might become a problem for our generation who do their courses online for communication, interpersonal skills. Yeah.

Lecturer A:

Thank you. Thanks, Student C. Student A, what about yourself?

Student A (Focus Group 3):

Yeah. I think the asking questions like, Student B said, we have already gotten used to it. Of course, the physical one is the best method to go. But forum, actually I don't really use the forum that much because sometimes the lecturer will wait for other people to answer your question before they actually give a reply. And that lag in time may let you forget what was your initial question in the very first place, however, email we will usually get a quicker response and more direct. So personally, I will prefer email over forum.

Student A (Focus Group 3):

And like Student C said, the interpersonal skills part. Yeah, that one I totally agree, it's very different. And we can't really get everyone to contribute equally in terms of speaking because some people are just more outspoken in general and some people just prefer to be more quiet. If it's in a physical space, we could go round by round but in virtual people tend to speak at the same time. And we are imbalanced in speaking and sharing your opinions.

Lecturer A:

Sorry, Student A?

Student A (Focus Group 3):

Yes.

Lecturer A:

Did you finish that point, your last point?

Student A (Focus Group 3):

Yes, I did.

Lecturer A:

Okay. Sure. All right. Thank you. I guess in lining with that as well, we were just curious as well, this whole online education that you've been [inaudible 00:33:54] doing it, so do you feel, do you think [inaudible 00:33:57] depends on your future. Another way to ask that?

Lecturer B:

I think his connection might be... Lecturer A, your connection is gone.

Lecturer A:

... was frozen for a bit. I will-

Lecturer B:

I think maybe I'll ask the question. But essentially, it's what Student C actually mentioned in terms of, your answer was actually for this question more or less. You have two years online you have one year off, now you have one year offline, two years online, how do you think this affects your career? Do you think it has any effects on your future career in some capacity or are you actually not worried at all? That's basically the question. What do you think are these impacts of these two online years options, Student B, sorry, Student B did you catch the question? So the question is, you basically have two years online, one year offline, what do you feel are the effects of this two years online on your future career as a pharmacist?

Student B (Focus Group 3):

Honestly, if anything, this year, this two years was it? Two years, yup. These two years online helped me to improvise. If anything, it challenged me how to improvise, if something happens, what to do, if this problem occurs, how to get over it. So I think it's not as bad as people say. With pretty much looking at the bright side basically, things will be fine, things, you get used to it.

Lecturer B:

Anyone any other thoughts on this two years on your future?

Lecturer A:

Actually, a few days ago, I had a meet up with my friends online. And then we talked about our risk for the future by doing this course online. So the main thing is, we are worried that we will kill our first patient once we go to hospital placement because since the exam, the finals and the assessments, they are mostly open book. So because it's open book, so we tend to not memorize or not pay so much attention on the technical part and the knowledge part. So we are very dependent on the resources and our own notes. So we are worried that once we go out, and practice in real life, we might accidentally kill or maybe harm a patient because we are too dependent on resources, and we can't do it on our own. And even our decision making we are fully dependent on the resources instead of for skills or... So this is the main issue.

Lecturer A:

And then other than the other ones that we talked about, we were also worried that we might not get a job because the future employers may think that we are not competent enough because we studied everything online. And what's the difference between studying online and studying from Google, right? So I think this is the main issue that we're concerned about.

Student A (Focus Group 3):

For me I think the lack of placement has two main issue for us. This is like we can't try out different conditions, we cannot go to the hospital and also committee actually try how it actually works and know what we like to determine the future path that we're going. And second one is less confidence because we are not actually talking to patients, we're just speaking to our classmate who actually know the topic well or the lecturers. So I feel that's the thing that we have to look out for this.

Lecturer B:

Okay. Thanks. Yeah. That said, and I think you mentioned some things at the end of the whole session, we will address some of your concerns here, what you just mentioned. But let's do it after the recording has stopped and then we can open and ask some questions. But anyway, let's move on. Lecturer A, do you want coordinate the next.

Lecturer A:

Yeah. So thanks, Lecturer B. I do apologize for the neck connection earlier. Just moving on. So last two questions with you actually as well. So the first one that we're going to ask, it's about your OSCE. So you've now gone through a few rounds of virtual OSCE already. So the first part of this question is, do you think these sessions have improved? And I will leave that to your interpretation.

Lecturer A:

And then the second part of this question is, do you think this virtual OSCEs are helping you to prepare you for your future practice? So two parts to this question is about the virtual OSCEs that you've been having. And so, just asking whether now that you've gone through a few rounds of this virtual OSCE, do you think it has improved or do you think there's been any changes? Do you think there's a better way? Do you think there's been any changes? And then the second part of the questions, do you think that it helps you to prepare you for your future practice?

Student C (Focus Group 3):

Yeah. I... Oh, okay. You may speak first.

Student A (Focus Group 3):

Yeah. Sure. So I feel like OSCE has improved over the years, as in lecturers got to know the Zoom better, and we had a check and everything. So it's also better for us because we are already used to it. And then does OSCE prepare you for future practice? Like, Student C mentioned before, because we're too reliant on our references, and I actually make simple notes on what to give for each disease. So I feel like I'll be less confident if someone just came in person and we all right away, and I have to think on the spot, it might be tough for me. Yeah. Student C?

Student C (Focus Group 3):

Yeah. I agree with your point. I do agree that OSCE has improved since the start of our online courses, the flow has been smoother, everything is okay. And the lecturers are also used to online OSCE. But sometimes some of them, I heard from my friends that they might still not be very, how to say? They are not familiarized with the scripts, so they might have to fumble around when they start with the OSCE. But I think this is not the only issue that we... This is not exclusive to online OSCE, it also happens in physically.

Student C (Focus Group 3):

And also, we are too reliant on our resources and not so I'm just get that we will be at a loss of words when a real patient, real customer or real doctor comes in and ask us questions. But there's also some good things about online OSCE is that I feel more confident and less nervous when doing online OSCE, I don't know why. Maybe it's because I don't have to stay in the environment for too long. And then it helps to reduce stress. So I can perform better in OSCE, even though I failed, but I do feel that I perform better. I feel like I will probably fail most of them if it's not done online. So I think it does help take away the stress and help us perform better. But it does not help with our reliance on resources. Yeah. And script.

Lecturer A:

Thank you. Student B, anything else to add?

Lecturer B:

He might have disconnected. Yes. I think he's stopped. Oh, he's back. All right.

Student B (Focus Group 3):

Oh, and I think... Sir, am I lagging?

Lecturer B:

Yes. You were lagging a while, but maybe you need to start over if you actually started saying something, just restart.

Student B (Focus Group 3):

All right. Okay. For the OSCE part, I think that OSCE is merely a patient to pharmacists interaction. So you have to see the patient, you have to have interaction with an actual patient. And yes, their health is something and online pharmacist are a thing nowadays. But still, you need to get how does it feel to be talking to a real patient with actual problems. And there is also your body language, which is not really shown. I might be using now some body language, but it's not shown because the camera doesn't capture everything. So it's not perfect, but it's what you have to work with.

Lecturer A:

Sure. All right. Thank you. Thanks for that. This is a really good point. Yeah. I guess OSCE in the general sense it does assess or we're trying to help reprove, I guess practice students communication skills. And whether that happens in a virtual format or a face-to-face format, there are both advantages and disadvantages as you've finally described already. But we do hope that it certainly helps you to prepare you for your future practice and for your personal growth as well.

Lecturer A:

I think moving on to the last question already for our time together this morning, so your best have had the, I wouldn't say privilege or maybe just the opportunity to have the experience of offline learning experience and also online learning experience. Going forward, is there anything that's happening in the online, any experience that we could carry across into the offline learning experience or that you would like to see being carry across into the offline learning experience?

Student B (Focus Group 3):

Honestly, I like the format of the exams, it's more application, more than memorizing. I hate memorizing. Although, I used to memorize a lot but I don't like it. No. I'd rather just apply what I've learned.

Lecturer A:

Okay. Yeah. Thank you.

Lecturer B:

Sorry, to clarify, you what open both exams where you don't need to memorize and you can bring in resources, is that clear or you need the nature of the questions? Oh, I think he might have got stuck again. But if both of you, if, Student C, Student A could share, you can go ahead, I think this kind of goes in along.

Student C (Focus Group 3):

So just to confirm, you're asking is there anything that we like from online learning that we can bring to offline, right?

Lecturer B:

Yeah. Basically what you're doing now and remember what you did in year one and what you want to implement from now into that year one scenario where you have to come for, just I know that last time workshop everything is live, but now that you get some things on the online experience, anything that you think is good now that you can hide it to your offline.

Lecturer B:

So let's say next year for someone you come back to campus and you have to take some units, how would you like it to be basically if you have to go through the normal week of discovery, IL, workshop, CTL, how would you like your week to be combined elements from everything we have so far?

Student C (Focus Group 3):

Student B, would you like to continue what you said earlier first?

Lecturer B:

Yeah. Sorry, Student B. Continue please. Sorry.

Student B (Focus Group 3):

I was saying that, wait, what was I saying? I forgot. Oh, yeah. I was saying that I want more application questions like how we used to have now rather than memorizing stuff and just spitting it out in the exam, I find these are more helpful. Yeah. That's all

Lecturer B:

All right. Yeah. Student C, you can continue.

Student C (Focus Group 3):

Yeah. I think the recording for the lectures are quite okay for online classes. So I think it should be continued and implemented in physical classes because what I noticed in year one was the recordings are not properly recorded, either that or they don't record that all because I think mainly because I want to encourage the international students to attend their classes. But I think it would benefit the students if they can record the physical classes, lectures or close the loops for us to revise that or refer it because sometimes lecturers will speak too fast and then we will miss something so we can refer back too. I think that's the thing that we can bring from online customer physical class.

Lecturer B:

Sorry, just to confirm, you want the lecture to be live, sorry, you want it live but recorded or you want it live for-

Student C (Focus Group 3):

Yeah. I want it live-

Lecturer B:

... right now where you have... Oh, okay.

Student C (Focus Group 3):

I want it live but it can be recorded for those who want to refer back-

Lecturer B:

I understand.

Student C (Focus Group 3):

... if they can understand the live one. Yeah.

Lecturer B:

Oh, okay. Understood.

Student A (Focus Group 3):

For me, I really like the idea of pre recorded IL and also like IL the mixture of that because we have something called iREAD which is based on ILs. So if we have a pre recorded, IE is easier to follow and it's steady interactive as well. So I like the combination of having pre recorded ILs and having live lectures if possible. And secondly, I feel like because we are doing it online, sometimes when the lecturer have additional information to share or they feel like their slides have too much information as compared to the slides they gave us, they will provide us with the materials and the slides, and I find that really useful. So if that is possible to implement this to pre recorded IL and materials.

Lecturer B:

One thing though, Jin, of course I don't teach you three, but you have a pre recorded IL, a live IL and a CTL, is that it?

Student A (Focus Group 3):

Yeah.

Student C (Focus Group 3):

Yup.

Lecturer B:

Thanks.

Lecturer A:

Yeah. So my question back just similar to that as well both to, I guess, Student C and Student A is why do you like what you've suggested? So maybe to start with Student C first, you said that you prefer live lectures, why?

Student C (Focus Group 3):

Actually another thing that I like for online class, the asynchronous workshops because I think that usually, this is the other questions the previous question, because usually the students will have to attend both... Because usually we'll have two workshops and then we'll have to attend both. So I think for case study, it's okay if it's live. But I think for the first one, it's okay if we keep it asynchronous, so we can do it, meet up another day with our own teammates and do it outside a cafe or something or the library, instead of going to the class and do it on the spot. It's less stressful, then I think it's more fun, and then encourages the students to interact. That's just my thought. Yeah. Okay.

Student C (Focus Group 3):

And as for why I like these two points that I suggested, the first one was recorded lectures. So I've heard a lot of feedback from my friends that sometimes the lecturer speak too fast, or maybe we just can't understand that concept at that moment. So we need time to regurgitate what they just told us and we have to process everything. So not everyone can record, some of my friends, they can record the audio on the spot but not everyone has the time or the devices to do that. So I think if the lecturer can record the slides, the audio and the video if they want, it will benefit us greatly so we can like revise back to see where we don't understand and maybe the points that we missed out.

Student C (Focus Group 3):

And as for why I like the asynchronous workshop is, it's flexible, the time is flexible, we can choose to do it anytime before the submission time. And we can do it anywhere. And then we can do it when all of us are free and less stressful, maybe during the weekends we can discuss. And the place is flexible, we don't have to gather in one place at the same time during classes and do the workshop. Yeah.

Lecturer A:

Thank you. Thanks for that, Student C. And, Student A, back to you, why do you like what you propose, so why?

Student A (Focus Group 3):

Yeah. I like pre recorded lecture for the same reason as, Student C because you really need time to digest everything and listening on the spot may cause you to miss some very important details. And then as for having materials that is provided by electron during after the lecturer has completed, is so that we have the access to images. Because I remember in year one, the lecturer actually displayed on the screen, but my phone was of a really low quality. So to be honest, I think half of the slides has actually gone missing. So I don't really have access to those slides, and it came out in the exams so there was a loss.

Student A (Focus Group 3):

And I really like, Student C's idea of asynchronous workshop because the usual type of workshop they recommend that was two hours. But my group usually will take three to four hours because we will also include discussion and clarification of concepts in discovery, which we didn't get to cover in ILs.

Lecturer A:

I see. All right. Thank you for those thoughts and sharing your frank opinions. And I think that's essentially all the questions that we have, that we wanted to cover for this morning. And as I said earlier, it was more so just to get some further confirmations and clarifications on some of the things that we got from the earlier completed focus groups. I don't have anything else to add but, Dr. Lecturer B, did you have any last few questions or clarifications for the students?

Lecturer B:

I have one question which is very just close to what you just said. So you have basically your suggestions are assuming that the unit is large enough that it has to ILs, two workshops in a week more or less, right? But that's not the case for most units. Most units probably only have one lm one IL and one workshop and one CTL. So basically, I want you to rank, if you had to choose pre recorded only one live one where you recorded, which would you choose? And also, between workshops if

you had a live or just one live or one asynchronous workshop, which would you choose? Because that's just the case for most units, so I'm a bit curious which do you prefer a bit more?

Student A (Focus Group 3):

I would definitely prefer live over recorded. Asynchronous workshop it is good because we get to discuss our ideas beforehand, before we jump into the actual workshop, which we have lecturer and we could clarify our concepts that we have brought up in the group. Yeah. That's just my session I would definitely prefer live over recorded.

Lecturer B:

Okay. Over asynchronous. Okay. Understood.

Student C (Focus Group 3):

For me-

Lecturer B:

Yeah.

Student C (Focus Group 3):

For me I'll prefer the live and the record for lecture. But as like you mentioned some units they don't have two workshops, for those I think it can be a mixture of both asynchronous and synchronous maybe. For example, for the case studies, every group they have to do a different case, maybe this one can be live. But maybe some calculation or any other simple ones, we can do it without the lecturer's guidance only to discuss the answers. I think that one should be okay if we do it asynchronously and the lecturers will discuss the answers later, like that. Yeah.

Lecturer B:

Okay. Great. Thanks. Yeah. That's all I needed. Student B, you have any thoughts on this?

Student B (Focus Group 3):

I would prefer, honestly a live IL. However, for the workshop, I prefer if it's just the simple asynchronous one because asynchronous workshops promote team building, which is a very important skill. And if we can manage an offline situation of people gathering together and discussing the questions together, it might produce better results even.

Lecturer B:

Okay. Thanks. I'm good. Thanks.

Lecturer A:

All right. Thank you very much for the feedback so far. Just so as a way of concluding from the three of you, is anything that we talked about this morning to anything struck to you, anything that you just wanted to highlight or you just wanted to reaffirm again, any of the points that we've talked about in the past hour?

Student C (Focus Group 3):

I think no, from my side.

Lecturer A:

Okay. Nothing from, Student C, nothing from, Student A. What about you, Student B, anything?

Student B (Focus Group 3):

Nothing from me as well.

Lecturer A:

All right. Thank you very much. I will now stop the recording. So that concludes our focus group this morning around your pharmacy education-

Deidentified FG7Y2 - Oct 2021

Lecturer B:

[inaudible 00:00:03] just to tell you first, at 9:45, I need to go [inaudible 00:00:07]. My video will just off, and I'll be gone for 15 minutes and I'll come back when we're through. At that time, Mr. Lecturer A will be carrying the focus group. Just to let you know in case you see me disappear.

Lecturer A:

Yep. No problem. Thank you. I've started the recording audio, and we might just get a head start. Oh, yes. I forgot to say one more thing as well, which is also important. We unfortunately don't have enough coffee bean cards to give to every single participant. Last year, those who took part had an option to get a Coffee Bean voucher for 50 ringgit.

So what happens is that because there are a few groups that we are running- so for each group, there'll be just one that's on offer. So at the end of this, we'll just do a ballot. And then one of you will be the lucky one to get a 50 ringgit Coffee Bean voucher. And I'll reach out to you for that as well via email. Okay? So I just wanted to share that with you. Also to thank and acknowledge you for your time.

All right, so just get a head start. I remember Student C and Student B told us that your background was university, if I'm not mistaken. And so I'm just referring to your time before university. But can I just check with Student A? Can I just check what was your pre-university background?

Student A (Focus Group 2):

[inaudible 00:01:24].

Lecturer A:

Within [inaudible 00:01:28], I see.

Student A (Focus Group 2):

Yes.

Lecturer A:

And this was at Sunway University?

Student A (Focus Group 2):

Yes. But it's in the Johor branch. Sunway College Johor Bahru.

Lecturer A:

I see. I see. So the most of your pre-university background you spent in Joho and then the plan was to come to Monash and Sunway for your university. Obviously because of COVID and all that, okay.

Student A (Focus Group 2):

Yeah.

Lecturer A:

I see, all right. Thank you, thanks for that. And so the first that I guess just really wanted to get some clarifications from all of y'all, you are all now year two students. We've told- I think, as you can start to appreciate that maybe the way we teach pharmacy here is a bit different than the other

universities. And again, as honestly as possible, after going through probably a year and a bit now of how we teach pharmacy at Monash, would you still choose to go through again the same style of teaching, the same style of learning? Yeah, anybody?

Or maybe while you're thinking about it, so I guess now you've gone through about a year and a plus now, about three semesters plus a bit. Three and a half semesters now, really, of pharmacy education here at Monash. And I'm sure, or I hope that you start to realize or appreciate the way we teach pharmacy has been a bit different when you compare with your friends in the other universities. So maybe there are things that you have liked or maybe things that you don't like about what you've been doing now.

And then maybe some things you hear that you hear that your friends are doing at their unis that you think, oh, maybe I like those? And why don't we do those at Monash? But again, just a very general question first. Would you- any regrets, if I could ask it that.

Lecturer B:

Yeah. And we don't mean in the sense of pharmacy. Assuming you are still taking pharmacy, would you choose to come to Monash or to take a, say... I guess most other universities would do it the normal, traditional way. You have a normal lecture, and things like that, versus here you have the discovery material, and then you come and do your IL.

We're not really lecturing you directly, you're not doing questions directly, things like that. So it's more the style. Would you prefer this style? Would you choose this style again or would you instead prefer something that you were more used to in your free university where a teacher comes in front of the class and tells you everything? Yeah.

Student A (Focus Group 2):

[inaudible 00:04:27].

Student C (Focus Group 2):

Okay, so in Monash, we have workshop. And sometimes the workshop SS. And then on the CTL. So sometimes it's a bit stressful because we didn't get all the knowledge. But it's also great at the same time, because we can really use our knowledge into practicals. And then sometimes we can also learn from our mistakes so we can remember better the knowledge.

So you asked any regrets. I think... Some of my friends are in the National University that has the optional courses, or the language courses. So I would also like to have some optional courses there. Yeah. So I think that's all for me.

Lecturer A:

Okay, Student C. So thanks for that. But you said some positives, but also negatives. But overall, if I would ask you to just choose one, would you still do this again if we gave you the choice? Again, it's a hypothetical situation or question. But could you just maybe just... Yeah, just share with us some of your thoughts? And again, you can be honest. It doesn't really impact anything. Yeah. I know we are your lecturers, but we won't feel any offended as well, so yeah.

Lecturer B:

By the way, the option of unsure is an option also.

Lecturer A:

Okay.

Lecturer B:

You can say yes or no, or you're not sure. It's okay to be not sure. We're not saying you must say yes or no. But just be honest with us about what you think.

Student C (Focus Group 2):

Yeah. I think [inaudible 00:06:13] because that does affect us and affect them. So I can't really have a certain answer for it.

Lecturer A:

Okay, yeah. Thank you. Thanks for that. Thanks, Student C. Anybody else? Student B?

Student B (Focus Group 2):

I think... I just... I not regret this. [inaudible 00:06:37] I don't regret my choice overall.

Lecturer A:

Okay.

Student A (Focus Group 2):

Sir, to me, I actually quite like the idea of we study this course before we enter any lecture, because I heard my friends saying it's very hard to test out. Like if the new topic is given in the lecture, because you have to follow [inaudible 00:07:08], yeah.

And then only to choose, I'm not sure, because I've got friends who also study pharmacy from USN. And also one is in... I'm not sure about the university name, but it's [inaudible 00:07:25]. And then sometimes I also think we all study similar stuff, but it's just that my course is a bit, I would say, interactive more, and then we've got some difference. But I'm not sure I would choose them, because there are many factors that consider it, and then, yeah.

Lecturer A:

Great, yeah. Thank you. Thanks for sharing, the three of you all. So definitely appreciate your honesty and your thoughts as well. So I think we can move on to the next question, unless, Dr. Lecturer B, is there anything else you want to add for us?

Lecturer B:

No. That's good for this question.

Lecturer A:

All right. So the next question that I have, so you've pretty much gone through almost three semesters of learning, having your classes virtually. And we were just curious was whether over the time that you spent already, was there anything that you realized that you felt that was a bit hard to learn this virtually? So was there anything or any class, or any of the sessions where you felt wow, this is actually quite hard to learn this virtually, and I wish we were in a face-to-face environment?

Student B (Focus Group 2):

Maybe the extemporaneous session in Dr. Lecturer B's unit, and the device counseling [inaudible 00:09:09].

Lecturer A:

Okay. And maybe just Student B, maybe just share with us why. Why did you think or why did you feel that way?

Student B (Focus Group 2):

For the extemporaneous session I think it's because after I finished my product, I'm not sure I did it correctly. And there's no one to physically check. They just can look at the picture. And for the device counseling, I don't have all the devices at home. So even though I just watch the video, I cannot familiarize myself with all the devices.

Lecturer A:

Yep. Thank you. Thanks for your honesty. And you felt that... Did you feel that this would impact your overall preparation to continue on in your own journey as a pharmacy student and later on into practice?

Student B (Focus Group 2):

I'm not sure, but I think yes.

Lecturer A:

Okay. Yeah, no problem. Thank you, Student B. Thank you. Anybody else? Did you have anything else that you'd like to add? Anything similar or anything different to what Student B has just shared.

Student A (Focus Group 2):

Yes. Mine is same as Student B, mainly about the extemporaneous class. Because I heard my friends, they've got two capsules, and also injection vials and things like that. So I'm just wondering if we also got the chance to do that. Because I understand it's very hard to do it at home because you can't really buy those kinds of ingredients. But just I think the experience would be great if we can carry out on campus, because they also can return back to campus to do that one last year [inaudible 00:11:17].

And also OSCE, because I think if OSCE can be carried out in a face-to-face, and then like that, and then maybe we'll get the chance to practice. It's more realistic when we- I mean, yeah. Because online, then... I'm not sure in the real one, but I think online we can refer to screenwriting but in real life, often it's really hard to refer to screen- then you have no choice. You have to really go oh, okay. Yeah.

Lecturer A:

Sure, thank you. I just wanted to confirm, Student A, that you mentioned earlier your other friends who went to do capsules and injections. They're from other unis. You said they were allowed to go back to labs to do it over this pandemic. Is that correct?

Student A (Focus Group 2):

Yes, but it's not currently. They did it maybe last year or early of this year.

Lecturer A:

Okay. Yeah, all right. Thank you. Okay, so thanks for sharing, and thanks for bringing up OSCE as well. I do actually have a question on OSCE later on. So I will continue on that discussion later on. But just stay focusing on this first question. And this question is still, again, on whether there was anything that you felt that was a challenge to be a part of virtually. Student C, did you have anything else to add? Anything similar or anything different?

Student C (Focus Group 2):

Yes, definitely extemporaneous and the device. But it's great that some of us can go back to campus this week, learn about the device. So I think, yeah, because I'm able to go, so it's not for me. And the other thing is... Actually I prefer everything to be face-to-face, especially the workshop, because sometimes workshops are randomly allocated.

And the student may be shy, and they don't talk. So our discussion becomes... We can't really discuss the question. Sometimes in the professional practice, I once experienced that there are seven of us in a group, but only two of us is discussing. So it's very hard, yeah.

But if the group members are... Sometimes we have fixed group members, it [inaudible 00:13:58]. At least everyone is comfortable so they can talk. But it's just the randomly allocated one.

Lecturer A:

I see. Okay, all right. Thanks, Student C. Yeah, so the points on what you mentioned as well has been also mentioned, particularly the workshop has been mentioned by other groups as well already. And so we really take those on board. So thanks, definitely, for sharing that.

So thanks again. I think that was all we wanted to find out from students. We'll just move on to the next question. So the next question is around this idea of flexibility. So from the earlier focus groups that we conducted, one of the themes that came out was this idea of flexibility that's associated with virtual learning. Because we're all, I guess a lot of us, at home, we seem to have this flexibility to how we interact with our learning materials, how we go for our online classes, how we go for our workshops.

For some of us, we don't have to travel 45 minutes to go to campus. We can just in two minutes get ready and dial in for class. So that's one example that some students have shared about this idea of flexibility. So my question to the three of you, how important it is... This whole idea of flexibility, how important is it to you personally when you think about your learning experiences?

So it's a big question, I guess, now that I've said it. But maybe if I can try to paraphrase a little bit more. When you think about your personal learning experiences here in university, yes, we have time tables, we have structures, we have all these things that are set up that sometimes we just cannot escape because we need to follow this format.

But then sometimes to a degree, we may have some room for flexibility, in terms of how the workshops are being run, whether the setting is a physical one or a virtual one. And, yeah, we were just wondering from your personal opinion, how important is being flexible when you are going through your... How you affect your learning experience?

Lecturer B:

I guess you can also think about it like when you're [inaudible 00:16:36] flexible in the sense that there's a time for every class, a mathematics time slot, or physics or whatever, you'll all have a time slot for everything. But because of the online thing, with discovery, you technically have flexibility, right? It's there. You can do it whenever you want.

Even for interactive lectures, I think a bit [inaudible 00:16:57] due to COVID. Actually, before that, it was a bit more rigid. And now it's actually a bit more flexible because they're being recorded. So that is kind of what we mean by flexibility, just to give you an example.

Student C (Focus Group 2):

I think flexibility is good. But it doesn't always... Face-to-face learning, I think it's... The only thing we can confront ourselves in this COVID pandemic. We have flexible time tables. We can do things whenever we want. Yeah. Yes, and I've thought about it.

Lecturer A:

Okay, so Student C, I guess, the context of that question as well. So moving forward, as you can now sense with this discussion of how we're going to bring students back to campus and all that. How do you think this can look like, moving forward? Or any hopes or aspirations from your end as a student?

Student C (Focus Group 2):

[inaudible 00:18:11] if we can keep this flexibility whenever we are back to campus.

Lecturer A:

Okay. So could you give us an example, like how do you think that would look like?

Student C (Focus Group 2):

Yeah. I'm also not that sure. But maybe if we can choose our class? [inaudible 00:18:41], but they're all the same. I think it's really difficult to do that.

Lecturer A:

Okay.

Student C (Focus Group 2):

Yeah, it's [inaudible 00:18:42].

Lecturer A:

All right. Okay. Student B and Student A, anything to add?

Student B (Focus Group 2):

I'm still thinking, but if I have any thoughts about this, can I come back again?

Lecturer A:

Yeah, no problem. Thanks, Student B. Student A, what about you?

Student A (Focus Group 2):

Yeah, same as Student B. I'm still thinking about... Yeah.

Lecturer A:

Sure. Sure. Okay. No problem. I realize it could quite a big question as well. But I guess if I could summarize, so just to summarize some of Student C's thoughts, and you can correct me if I've misunderstood it, you do hope for some degree of flexibility going forward, but you don't know how that can look like. Is that correct? Yeah, okay. All right. Thank you.

And I think we'll just continue on to our next question first for now. I guess this is related to something that I did mention earlier as well. So now there seems to be this discussion now about coming back to campus now even though in the middle of the pandemic, but we seem to have some plans or some discussions ready to bring students back to campus, and to probably kickstart our face-to-face session maybe in the month to come.

You know already for 2012 there are some sessions planned for this week, and I think there will be more in your other groups. And I think there will be more structured plans from next year

onwards was well. Just out of curiosity, basically, do y'all have any concerns? Or what are your thoughts about it? How do you feel about it?

Student C (Focus Group 2):

I'm really looking forward to going back to campus. My little brothers haven't been vaccinated, so I think that's the only concern.

Lecturer A:

Okay. And Student C, why are you looking forward to going back to campus?

Student C (Focus Group 2):

Experiencing uni life, get to know some friends face-to-face, not virtual friends.

Lecturer A:

Thank you. Thanks for sharing, Student C. Also personally for us, we are looking forward to having some face-to-face interactions with students as well. I don't think we've actually had the chance to have met y'all in this class physically as well.

Thanks for that. And I guess your concern about your little brother is that because you know you're thinking of going out to be interactive more with people physically, you also want to make sure that you don't want to be any... To obviously be a carrier for the disease for your family members. Yeah. Yeah. Thank you. Thanks for that, Student C. The other two? Okay, Student B?

Student B (Focus Group 2):

Yeah, I'm also excited to go back to campus, but to be honest, I don't put a lot of hope on that, because... Every time I put hope on that, I'm always disappointed. So, yeah. To think about going back to campus. So it doesn't really excite me right now, because of, yeah.

Lecturer A:

Sorry, because of? Sorry.

Student B (Focus Group 2):

[inaudible 00:22:50].

Lecturer A:

Oh, okay.

Student B (Focus Group 2):

Because...

Lecturer B:

[inaudible 00:22:56]. That's kind of the issue, in a sense.

Student B (Focus Group 2):

Yeah. So I better not put hope on that, because...

Lecturer B:

Okay.

Lecturer A:

I see. Okay. Thanks, Student B.

Student A (Focus Group 2):

Yeah. Okay, I think I look forward to going back to campus. Also, like some of our friends, we all think we can go back to campus next year. I have a very strong stance, like we can go back next year. Because in terms of concerns about COVID, I think we just follow the SOP, and just wear the mask, and then even wear the [inaudible 00:23:44], and then I think we should be okay. Because to me, I really want to have a physical [inaudible 00:23:53] because it's already two years staying at home.

Lecturer A:

Understood. Yeah, thank you. Thanks very much, Student A. So I guess yours is similar. Your thoughts sound similar to what Student C was sharing, that you're definitely looking forward and feeling excited for this returning to campus. All right, thank you.

Moving on to our next question, again, you can be as honest as possible as well. Out of curiosity, just going through now you're early three semesters in a bit now virtually in the middle of this pandemic, from a general sense, has there been any concerns so far, or any issues that you felt that you just feel nervous about, or maybe anxious about? And this is towards your education experience.

Again, it's something of a big question now that I've said it as well. But if I could paraphrase it a little bit more, yeah, I guess we all didn't plan for this, but this happened. And then now you've really gone through about three semesters and a bit over a virtual format. We're just wondering was there any concerns. So maybe if I can share some examples first.

So some of your seniors who they started uni in a face-to-face format. And then COVID happened, and then obviously they also had to go to a virtual format. So when we flipped over to a virtual setting, they were concerned whether they could still learn and still understand. And, yeah, go through all that difficult experience while in a face-to-face environment.

For yourself, I think you probably maybe had maybe only a week or two orientation in a face-to-face format last year. And then after that, covid happened and we all went virtually. So just wondering, over the whole time, over the past three semesters and a bit, really, was there anything that bugged you, or any concerns?

And I know we do have your SSLC's, your [inaudible 00:26:25] where we always get feedback from you. And then from us as an academic, we try to resolve any of your concerns. But we're just wondering whether there's anything else bigger than that from your end. And if there's no concerns as well, you can also say no, I'm okay. No worries.

Student C (Focus Group 2):

It's just at the start of this, at the start of year one, because everything was transferred online, [inaudible 00:27:00] I'm quite lost, actually. I'm not familiar with those, and I don't know where to find the information. But after that, I think...

Lecturer A:

Okay. So there's a period of feeling lostness. But then over time, you don't feel so lost anymore. Is that correct?

Student C (Focus Group 2):

Yeah. Just the first two weeks.

Lecturer A:

Mm-hmm (affirmative). Thank you. The other two? Anything else to add? Anything similar or anything different.

Student A (Focus Group 2):

Similar to Student C, yeah. The first one month, for me, in the first year, my first semester. Then I actually had to figure out everything. Because I feel very lost at times, and also very helpless. Because to me, I don't have any other friends study the same course with me. And I really got no one to ask about this [inaudible 00:28:05], yeah.

Lecturer A:

And right, Student A, do you still feel the same, or do you feel confident? You feel okay?

Student A (Focus Group 2):

Yeah. Now it's like much, much better. I'm confident in such [inaudible 00:28:24]. Yeah. And then I'm [inaudible 00:28:27].

Lecturer A:

Yeah, like Student B and Student C today as well. I don't know whether you know Student C, but at least you know Student B, which is good. Yeah. All right, thank you. And Student B, was there anything else?

Student B (Focus Group 2):

Yeah, the same like Student C and Student A. I'm not sure that this fits into the question.

Lecturer A:

Mm-hmm (affirmative).

Student B (Focus Group 2):

First time I used to email lectures, and I think it's the same, the SSLC that students prefer that all of us use the discussion forum so all of the information can be shared. And for some reason, I think it's not wrong for lecturers to encourage to use the forum. But they're not [inaudible 00:29:28] talking about any lecture. It's just I feel personally that maybe not all the lectures, just also it's the use of forum.

Lecturer A:

Okay.

Student B (Focus Group 2):

If I am comfortable with using email then I hope they understand that I'm very busy, or something that they understand.

Lecturer A:

I see.

Student B (Focus Group 2):

I'm not saying that I email them, then I think I will trust a reply back. I personally like using email compared to the forum.

Lecturer A:

Okay, sure. Thanks, Student B. I think that's a really interesting point. So if I could talk on this whole idea of your communication relationship with the teaching staff or with the academics in this virtual format. Hypothetically, if we were in a face-to-face class would you have been more comfortable, say, putting up your hand and asking in front of the class? Is that something you would do naturally or not? Just out of curiosity.

Student B (Focus Group 2):

It depends on the size of the class, maybe.

Lecturer A:

Okay, sure. So do you feel a bit that you'd be more comfortable doing that in the smaller class. Is that correct?

Student B (Focus Group 2):

Yeah.

Lecturer A:

Okay. All right. Thanks, Student B. So thanks as well to Student C and Student A for these questions and answering those questions. I think we can move on now to the next question. So we've got about two more-

Student C (Focus Group 2):

[crosstalk 00:31:06] I think I have something to add to that.

Lecturer A:

Oh, yeah?

Student C (Focus Group 2):

Regarding the use of email or the forum, because if I email the lecturers, I can really get the answers I want. But if I use the forum, sometimes the lecturer doesn't reply. And [inaudible 00:31:24] on discussion [inaudible 00:31:27]. I can't get a good answer there. Sometimes that's why I trust to use email.

Lecturer A:

Yeah. Understood. Yeah, thanks for adding on to that, Student C. Was there anything else from anyone? Okay, all right. So I'll just move on for now. And then just, yeah, as I said, two more questions, and we're done for the day already.

For the next one, I just wanted to check a bit, it's on your OSCE. So Student A did mention a little bit about OSCE earlier. But by now already, you've already gone through a few rounds of OSCE. We were just curious, do you think this form of assessment, having a virtual OSCE, does it help prepare you for your future practice?

And then I think Student A earlier did say there were some shortcomings or limitations about having a virtual OSCE. We were just wondering, do you think after going through it a few times, was there any improvements, or your contempt about the virtual OSCE is still there?

So two parts to the question, all right. So the first part of the question is do you think this kind of virtual OSCE assessment, does it help prepare you for your future practice? And then the second part of the question, if you agree that there was some shortcomings or limitations in the earlier rounds of the virtual OSCE, do you think it has improved over time?

Student C (Focus Group 2):

In the virtual OSCE, it prepared us for the future but [inaudible 00:33:24] because we haven't physically get the device, and we are not that sure how to use it, and now we have to teach someone else how to use it. So that's the only problem.

Lecturer A:

Mm-hmm (affirmative). And anybody else? Anything else to add?

Student B (Focus Group 2):

Compared to physical OSCE, I think the Zoom OSCE prepared us less, because I can rely on something like a script. And, yeah. In practice, I think it's not possible to do that. So, yeah. But I think it can improve over time. It depends whether or not I want to change this, that have been abusing a script.

Lecturer A:

Okay.

Student A (Focus Group 2):

I think OSCE is very useful, and I think [inaudible 00:34:41] initially is that OSCEs since year one, I think some universities even really start OSCEs since students are just year one. So I think OSCE is really useful because it helps us and prepares to talk to patients in real life.

So I personally love OSCEs at this point. But it's also very scary [inaudible 00:35:11]. Yeah. And this is the benefit. And then limitations about the device counseling role, because we really don't ever touch or use the device before. And then maybe we should just simply memorize the steps, and then we teach the patient instead of relearning everything.

Lecturer A:

Okay. All right. But just stepping aside from the- so I appreciate the points that you make about the medical devices. But just stepping aside, away from the point, something about OSCEs that we also test a lot on, we're trying to assess students' communication skills and abilities, and I think that's really been quite obvious and apparent.

So out of curiosity from your end, do you feel that having one true virtual OSCE so far, has it been helping you improve your communication skills and abilities as a future healthcare professional? And again, you can say yes, it has, or no, you felt no. Or if you're unsure, that is fine as well.

Student B (Focus Group 2):

I think yes, because before this, I don't really... Oral examinations, when I talk with them. Yeah, I just [inaudible 00:36:51]. But for this OSCE, because it's harder, so I put a lot of effort to prepare for it during the [inaudible 00:37:07]. So I think it improved a lot, in terms of communication.

Lecturer A:

Okay. So just to summarize Student B's point, and again, if you can correct me if I misunderstood it wrongly. So you felt that even though the OSCE has been in a virtual format that having the need to

prepare for it and still go through it in a virtual format, it still has helped you improve on your communication abilities. Is that correct, Student B?

Student B (Focus Group 2):

Yeah.

Lecturer A:

Thank you. And then, I guess, anything else to add from Student A or Student C?

Student A (Focus Group 2):

Really, I agree with Student B. I think this OSCE really prepared us and our communications.

Lecturer A:

Okay. So then I guess the other question now, because we are doing it on a virtual format, so maybe we are preparing you for virtual interactions, but do you think it has helped prepare you for face-to-face interactions. Or do you feel that the assessments that we've done through this virtual OSCE, does it help prepare you for any future or even any current face-to-face conversations?

So, for example, after going through what you have gone through in the virtual OSCEs, do you suddenly find yourselves realizing that even though we don't have so much face-to-face interactions now, but the little face-to-face interactions that you have, do you notice, oh, I seem to be getting better or not? Or if that's also non-existent, you can say I don't see anything.

Student C (Focus Group 2):

If they do the virtual OSCE, it, of course, prepares us for face-to-face work. Maybe when we are doing it face-to-face, we'll have some other problem, like [inaudible 00:39:22] there's some pressure at the end.

Lecturer A:

Mm-hmm (affirmative). Mm-hmm (affirmative).

Student C (Focus Group 2):

It does prepare us, but maybe we're already face-to-face with some other problems.

Lecturer A:

Okay. That you may not be aware of yet. And then maybe these other problems can help you to grow as well. Is that correct?

Student C (Focus Group 2):

Yes.

Lecturer A:

Okay. Thank you. And Student A or Student B, anything else to add?

Student B (Focus Group 2):

Yeah, I agree with Student C. But another thing is maybe a non-virtual communication. Because during the Zoom OSCE, you can just see our facial expression. You cannot see... I mean,

communication is not just you talking. So in the Zoom OSCE, I can maybe put on a poker face or something. I act very not nervous.

Like my hands and, yeah, other parts are very- you can see that I'm nervous, and during physical interaction, it's obvious. And so I think for the Zoom OSCE, it's quite hard to improve this, because the lecturers cannot see this and they cannot correct us when they notice it.

Lecturer A:

Okay, yeah. Thanks, Student B. And Student A, anything else to add?

Student A (Focus Group 2):

Yeah, I think Zoom OSCEs- or OSCE that are not only Zoom, OSCE really prepared me to communicate in real life because I have a short working experience in a community pharmacy before, but [inaudible 00:41:22] in 2019, I haven't studied uni. So then if this time, I was like, you got a chance to go working part time in the community pharmacy, I would change my way to communicate with patients, because I have learned all these structured ways instead of just simple talking. So I think this really helps me in obtaining more information from patients, and also helps me to structure my interview better. Yeah.

Lecturer A:

Okay, all right. Thank you. So those are all really good points, and I really appreciate those honest thoughts. But just out of curiosity now, just similar to this sub-question or this main question, how would you all feel if we told you that okay, for those who can come back to campus, we are going to have a face-to-face OSCE this semester.

I'm not saying it's happening. I'm just asking first. For those who are, let's say, hypothetically again, if this was to happen, how would you feel about it for this semester? So that means after we come back the next few weeks, for those that can, we're going to get you to come back for a face-to-face OSCE. How do you feel about it?

And I know hypothetically we're all- because I don't know where all of you are. Some of you may be interstate and have barriers and all those things. But let's say hypothetically for those who can come back to campus, we're going to get you to come back for a face-to-face OSCE. And then for those who can't come back to face-to-face to campus, we'll have a virtual OSCE for you.

So two parts of the question, how do you feel about you coming back for a face-to-face OSCE assessment, and then how do you feel knowing that oh, I'm coming back to campus for a face-to-face OSCE, but my friend, who can't come back to campus, they will have a virtual OSCE. How would you feel about it?

Student B (Focus Group 2):

Does it mean that assuming I'm the one going back to campus?

Lecturer A:

Anything. It can be anything, yeah. You can put yourself in different positions and then just let us know how you feel about those situations as well. And it's a friendly discussion. And again, I'm not saying it's happening. It's a hypothetical situation. Anything about the uni, it's all going through the proper UC. So listen to them. Yeah.

Student B (Focus Group 2):

If I'm the one going back to campus, I think it's not fair for me.

Lecturer A:

Mm-hmm (affirmative).

Student B (Focus Group 2):

Because to be honest, I think the Zoom OSCE will be easier than the physical one, because you can do a lot of things that... You can use a lot of things that doesn't apply to physical OSCE [inaudible 00:44:25]. Yeah. I will accept that, because for my learning, I think it's better because I'm actually doing it physically, and it'd only be I will get... Maybe I will get lower grades, because I'm still not really prepared for a physical OSCE. But at least I can know [inaudible 00:44:59].

Lecturer A:

Thank you. Thanks, Student B. And Student C or or Student A, anything similar or different to add?

Student C (Focus Group 2):

I agree with Student B that is not fair, because virtual OSCE is definitely easier. And also to practice the OSCE, if it is a virtual OSCE, we practice online. So if it is a physical OSCE there, we will [inaudible 00:45:36] practice face-to-face with our peers. So I think this kind of thing was better than that. So this is a problem.

Lecturer A:

Okay, all right. Thank you. And Student A, anything else similar or different to add?

Student A (Focus Group 2):

I have nothing to add, but I agree with Student B and Student C.

Lecturer A:

Okay. All right, thank you. And again, thanks for sharing. And again, not saying that it is happening. It was just a hypothetical question that I just wanted to know how students felt about it. Anything that is linked to your formal unit assessments about OSCE, again, direct back your quarries to your unit coordinators and the OSCE coordinator as well. Okay? So just a reminder on that again.

I think we're on to our last question now. [inaudible 00:46:30], I know we have mentioned something similar already, but again, this is linked to this whole point of coming back to a face-to-face learning environment with looking ahead. From your perspective, do y'all think was there anything that has been happening well that happens over virtual format that you think would like to see this happen again when we come back to a face-to-face session? And it can be anything.

So give some time for you to think about it as well. But yeah. So I think from the different focus groups we've conducted so far with the different students, some students have mentioned about some aspects of this online learning or this virtual learning that they have liked. So now from our end, as your academics, we're just wondering is there anything you think you'd like to see this carry on in our face-to-face learning and teaching?

Lecturer B:

One thing I noticed is this person is hard for them, because they've never been on campus and then to the offline way of teaching.

Lecturer A:

That is very true.

Lecturer B:

So you have to draw your assumptions based on what you [inaudible 00:48:06] in terms of what the face-to-face class looks like. And the question is essentially what do you have now that you think would be good to transfer over when you have to come back to campus? Something like that.

Something that you think we ought to keep, that's good, that you actually like it versus- you've mentioned things you don't really like right now, and that you [inaudible 00:48:31] offline. This is great, and [inaudible 00:48:34] you really want to just have it offline. But anything [inaudible 00:48:36] kind of mix it up.

Student B (Focus Group 2):

Maybe for the lectures, continue recording them. Yeah. For physical class, I think it's not recorded.

Lecturer B:

It is still recorded. We used to have- like your CTL. So I think the new thing- oh, maybe we can explain. So one of the new things you all had for last time, the IL and CTL used to be quite similar in the sense that it would be a live person talking in the ILs. And then you notice you usually get [inaudible 00:49:23] at least something like that for your IL. It's actually given by the lecturer, and each of those activities you are discussing among your peers in your [inaudible 00:49:28] class itself, and then you would answer them live in the lecture itself. So the IL method will be done [inaudible 00:49:28] campus.

And the one where we prerecord and then you have the answers. So you have questions and then a small recording that you answer, [inaudible 00:49:28], that is an online implementation. So the CTL is quite similar. CTL would more or less be the same thing you have now where usually the lecturer is there speaking, and then you can ask your questions, and we will summarize things, and things like that. So, yeah.

Student B (Focus Group 2):

But both IL and CTL is recorded and shared to us, is it?

Lecturer B:

Yeah, they will both be recorded. Yes.

Student B (Focus Group 2):

I need time to think.

Lecturer A:

Yeah, that's fine, Student B. Thank you.

Student C (Focus Group 2):

I don think I have anything to share, because [inaudible 00:50:59] quite good.

Lecturer A:

Yeah, that's fine as well. Thank you. Thanks for your thoughts, Student C. I guess, in a general sense, can I assume that students would like the interactive lectures and the CTLs to be recorded, or to have them in a... Yeah, in a recorded format, whatever that would look like. Whether it's a one-shot 45 minute, 60 minute clip, or a three or four by 15 minutes clip that's all fine now. But you still would like a prerecorded version.

Okay. So how do you y'all feel about, let's say if we can all come back to face-to-face environment. This whole format of where we allow you to... So those who like to come for class in a face-to-face setting for your interactive lectures, y'all can come. Those who don't like to come or who can't come for any reason, the session will be recorded, and you can view it on your own time.

But then it's compulsory, then, to come back for a face-to-face workshop. And then it is similar for CTL where for those who can come, you come for your face-to-face CTL. For those who cannot come, obviously the session will be recorded, and you can access it on your own time. What do y'all think about that? Would you like it, or would you think, no, that's not fair. If everyone has to come for the IL and CTL, everybody must do that.

Student B (Focus Group 2):

It's a little bit not fair.

Lecturer A:

It's not fair? Why do you think that is?

Student B (Focus Group 2):

Because... I want to go physically, but I cannot go. So it's not fair.

Lecturer A:

I see, okay. Understood. All right. And was there anything else that contributes to this feeling of not-fairness, Student B?

Student B (Focus Group 2):

Nothing I can think of now.

Lecturer A:

Okay, yeah. No problem. Thank you. And the other two, any thoughts to that?

Student A (Focus Group 2):

I think it's fine for me. I mean, I can go to campus. I can just stay in [inaudible 00:53:41] then I can attend the class live. I mean, let's say if the lecturer says we will have our physical class, then I think I will go because I want- because I say I want the physical lecture.

So in terms of fairness, I think it is okay for me, because some people, they have their own concerns of life, and also they have their own concerns of not going onto campus, face-to-face for class. So I think I don't really mind about that. Yeah.

Lecturer A:

Okay.

Student A (Focus Group 2):

Yeah. It depends on [inaudible 00:54:23] depends on yourself, whether you want to go or not, yeah.

Lecturer A:

Thank you. Thanks, Student A. Student C, anything similar or anything different? Or anything else to add?

Student C (Focus Group 2):

I agree with Student A. So [inaudible 00:54:42] is compulsory, so everyone should join. And for IL and CTL, [inaudible 00:54:50] ready to go or not is good.

Lecturer A:

Is not good, or?

Student C (Focus Group 2):

It's good.

Lecturer A:

Oh, it's good. Okay.

Student C (Focus Group 2):

Depends on what the learning style we want.

Lecturer A:

Okay. All right, thank you. So I think that's- yeah, we're almost towards the end already, unless there's any other comments, or any thoughts. I guess over the time you've- you know, some parts of the questions you felt that you wanted to think a little bit more. But I think Dr. Lecturer B- that you can obviously share now, but I think Dr. Ron also may have one question that he just wants you to check.

Lecturer B:

Yeah. I [inaudible 00:55:34] and I just wanted to go a bit further. So you all had your two years online, right? Do you think this will affect your career in anyway? The fact that you had basically half your classes online, and half of it may be a mix of online/offline. Do you?

I say, do you all think in any way that this would affect your future as a pharmacist or your career in any way? Did it upset you, or you actually was [inaudible 00:56:05]. I just was wondering whether you have some concerns on this. Probably it's the first time in a long time that this will ever happen to anyone [inaudible 00:56:49] two years like this again, the other two years. [inaudible 00:56:49].

Student A (Focus Group 2):

I feel that should we pass, I think it's okay. Just the [inaudible 00:56:49]. I'm wondering if we have some placement, or something, if possible. Yeah.

Lecturer B:

Okay. So at the end of it, I'll explain- actually, we will have a few things for that, I think. In fact, in year four, [inaudible 00:57:05] and there will be more things. But anyway, I guess your concern is practical skill. So these two years might have been a bit weaker for learning practical skills, the things that you need to tangibly touch something. Medical devices, extemporaneous compounding. I guess OSCE's a little something also that's not tangible or face-to-face to a real human being.

Student B (Focus Group 2):

So maybe the placement. Because when we go on placement, we can- I'm not sure what we're doing for placement in year two and year one, but I think it's time for us to apply and observe the real life

setting of our future workplace. And the fact that we cannot do it now is [inaudible 00:58:29], because, in fact, to me [inaudible 00:58:35].

Student C (Focus Group 2):

Yeah, I agree, Student B. Because I think placement will help me to now understand which field I want to be in. Maybe in a community or in a hospital, or even in the pharmaceutical. I'm not sure that- I mean, [inaudible 00:58:52] in a hospital, community, and pharmacy, something like that. So... I'm still quite lost on whether I want to be in the pharmaceutical part, or in the hospital, or at a community pharmacy. So maybe with placements we might get some insight on which type we want, what we love [inaudible 00:59:18].

Lecturer B:

Okay, thanks. So that's my only question, so I'll pass it back to you, Wei.

Lecturer A:

Yep, okay. All right, thank you. So we're at the end already of our session already. We've gone a bit, a few minutes past time. But was there any last closing comments, or any feedback, or any thoughts that y'all... Any answers that you want to add to what we talked about this morning already, from anyone?

Student B (Focus Group 2):

[inaudible 00:59:55].

Lecturer A:

So none from- okay, Student B?

Student B (Focus Group 2):

The placement. Now I think a placement is hospital, community, and industry [inaudible 01:00:06].

Lecturer A:

For our placements, yeah. Generally, it will be those three aspects. Yeah.

Student B (Focus Group 2):

I'm not sure it's possible, but I would like to have a placement in academia, because I don't know what is that, like R&D, and those type of stuff. Because it sounds interesting, but I'm not sure if that is the type of work that I would like. Not academia, but R&D that type of-

Lecturer A:

Oh, okay.

Student B (Focus Group 2):

I'm not sure which one.

Lecturer A:

Okay. Sure, sure. Okay, I think I know what you're asking. Okay, I'll talk about it after I finish this recording. But just again, coming back to what we've talked about this morning about your education experiences so far, whether if there was anything else that you all- to whatever we've talked about the past hour if there was anything else that you would like to add.

So I think there was none from Student C. Student A, anything else to add? Nope? Okay. And Student B, nothing else to add? Okay. All right. Thank you so much for your thoughts. I'm just going to stop the recording right now.

Deidentified FG8Y4 - Oct 2021

Lecturer A:

All right. This is the following focus group that is accompanying our education research project. As I mentioned earlier as an introduction, the reality is that the way we teach pharmacy in [inaudible 00:00:22] is a bit different than other universities. I think you have heard of ... you of all students have heard of this [inaudible 00:00:27], so I don't have to explain so much in terms of what those differences are.

Lecturer A:

Last year, when we conducted ... I guess last year and this year, when we shared with you a survey on asking about some of your feelings towards this style of learning, so I don't know whether if you took part or some of you may have elected to took part and some of you didn't. But essentially, the survey asked you about your attitudes and your preferences towards this style of learning here in [inaudible 00:01:01].

Lecturer A:

Dr. Lecturer B's just now sharing on the screen a screenshot or a sample of what some of those questions that we asked you. Hopefully, this can re-jog your memory as well if you took part in the survey. What this survey was actually also doing was that they were asking you about your attitudes and your preferences towards this style of learning. Very interestingly, and across two cohorts, we found that there was a subsequent drop when you progressed from years two to years three on your attitudes and preferences. If I can just repeat that again, what we found that no students, in years one, they were at this level.

Lecturer A:

Years two, they liked it a bit more, and then at years three, we thought that they would like it more, but no, they liked it less, so there was a drop in the transition from years two to years three in terms of their scoring and where they sit.

Lecturer A:

We were just interested or curious why that was so. Anybody would like to start from your perspective, why do you think that was so?

Lecturer B:

I've been shifting this slide, so they might be taking a bit of time to digest. This [inaudible 00:02:28] you don't actually need to look at every question and answer based on that or anything. It's just to give you an idea of what ... just to refresh your memory on what you actually did for this survey. But the main thing is this drop.

Lecturer B:

It's quite consistent. If you take a year two, generally, the cohort of year two and you follow them to year three, you will notice that generally the mean scores are across almost every domain drops from year two to year three in terms of attitudes, preferences to what the [inaudible 00:03:03] classroom. We're curious as to why this is happening and we want your thoughts on why do you think this is, based on your experiences.

Student D (Focus Group 4):

When you mean a drop, what do you mean? Is it [crosstalk 00:03:28]-

Lecturer B:

Yeah. They are tending more towards disagreeing versus ... In general, when you do a year two student, they would be maybe more towards the agree side of things, and then when they transition to year three, it drops to maybe more towards neutral or more somewhat agree, something like that. It's actually just trending downward. Which is ... yeah, we actually just don't know why and we're wondering why basically.Maybe the main thing is what's so different about year two to year three to you that would make you perhaps feel that you prefer it less or anything along those lines.

Student D (Focus Group 4):

Can I go first?

Lecturer B:

Yeah. Sure.

Student D (Focus Group 4):

I think that for my year two and year three, it's basically a transition from physical to online. When I was in an online situation, I don't really have physical experiences where I have to go to class, stay in class, so I actually have ... I would say I have more time in terms of when I wake up or my freedom. I'm kind of my own discipline teacher that I must tell myself what to do.

Student D (Focus Group 4):

That's why maybe for my case, I moved away from uni quite a lot. Like my priorities change. Yeah. If you want to talk in terms of like if I did my second year physically, I would still say the same. It's because maybe when you're freshman, you're first year, you want to be super good in grades and education. That you give 100% to it. And then maybe along the way, you meet more friends from other courses, and then you join some core curriculum, and then you tend to still go and do other things and have new priorities.

Student D (Focus Group 4):

I guess whether or not it's online or not, my results will still remain quite similiar. Because eventually, I know that I will let uni just be my priority. Not education-wide, I'll just do more other things. I think that can explain some of the things that I disagree.

Lecturer A:

Thank you. Thanks Student C. What about Student B?

Student B (Focus Group 4):

Yeah. I actually agree with Student C as well. I feel like as you progress to year three and year four, your priorities actually start to change. You're already having like the right momentum for your education, so you tend to focus on other things as well.

Lecturer A:

Thank you. And Student A, any thoughts?

Student A (Focus Group 4):

No. I basically agree with them. The transition, it is the same for me as well.

Lecturer A:

Okay. Thanks for that. I guess Student C did share interesting point. Because when you're [inaudible 00:07:07] for some of you, it coincided with this flip to the online learning environment. But we also sampled the other cohort of students, which were online in year two and also online in year three. They also had the same pattern where they dropped, when they progressed from years two to years three. That part about changing from offline to an online environment may not be so applicable to them, but we also noticed the same results.

Lecturer A:

One of their comments that is when we asked them was that they felt that the years three units was ... and I quote and unquote one of the words that was used was chaotic from the students' experience.

Student A (Focus Group 4):

Agree. Agree.

Lecturer A:

Okay. So agree as well?

Student A (Focus Group 4):

I agree. Yeah. Because I'm in that cohort right now, so I can also say that it was very chaotic.

Lecturer A:

Okay. What about the other two, Student C and Student B? Did you all felt the same or not?

Student D (Focus Group 4):

I personally [inaudible 00:08:13].

Lecturer A:

Okay. Student B is nodding her head as well. I assume that's the same feeling as well.

Student B (Focus Group 4):

Yeah.

Lecturer A:

Okay. All right. Thank you. I guess in line with that, or connected with that as well, we also found that ...

Lecturer A:

Oh, sorry, Lecturer B. Yeah.

Lecturer B:

Yes. [inaudible 00:08:38] just because chaotic, confusing, that's the main thing. [inaudible 00:08:42].

Student A (Focus Group 4):

I think- Oh, bless you, whoever that was. For me, it was more of ... like in year two [inaudible 00:08:54] because I only have one unit, I did not really feel it. But when it came to [inaudible 00:08:58] two and then all the units are there, I would say some units don't have a very well thought

out planning. In turn, it kind of messed up a lot of people's schedules as when do you practice this, because it's no schedule sessions, you have to schedule it yourselves. And also first tend to be very unavailable. In that sense, it's quite clear I think.

Student D (Focus Group 4):

I didn't know that [inaudible 00:09:21] only has unit. Did they change?

Student A (Focus Group 4):

No, for me, because I took intermission.

Student D (Focus Group 4):

Oh. Okay. I think year three somehow was the hardest for me. It's like so bad. I feel like there were too many knowledge and it's super crowded in my brain. There's like exams every week, and we constantly have to deal with cases and then upload them. It's like in uni, when the class ends, it ends. You go and do your own thing. But online, for year three [crosstalk 00:10:06]-

Student A (Focus Group 4):

You tend to think there's something at the back of your brain that you missed.

Student D (Focus Group 4):

Yeah. Also, I have to work till nighttime to submit something, a case study for the following day. It's like I don't have a break. Yeah.

Lecturer B:

Thanks. I think I got the answer. Back to you Lecturer A.

Lecturer A:

Thanks. Thank you. Thanks for sharing as opening comment, and as a first question to explore. In line with that as well, now just reflecting on some of the data that we collected from this survey as well. We also found another interesting pattern that we noticed was that across all the cohorts that we sampled, compare to, contrast to- Sorry, Lecturer B.

Lecturer B:

No, yeah, your connection. You were cutting out a little. Do you want me to do the questions?

Lecturer A:

Am I back? Hello?

Lecturer B:

I think you're back.

Lecturer A:

Yeah. Go for it.

Lecturer B:

Okay. Essentially, maybe just look at the screen. We ask this question ... basically, we ask you the same questions here for both [inaudible 00:11:26] settings in workshops, the workshops helped me. These are the questions. The lectures helped me, these are the questions. We noticed throughout all four years, if you take the workshop questions, generally most of them tend to agree that the workshops are helping them to clarify what you learned in [inaudible 00:11:46] activities to develop problem solving skills [inaudible 00:11:49] all these points.

Lecturer B:

We noticed that most [inaudible 00:11:52] is more applicable to workshops and less applicable to lectures. And again, what's your opinion on this? Why do you think this is the case? Or is it true for you? And that's kind of the question.

Student D (Focus Group 4):

I think I agree with that, because lectures is a pre-study thing. We need to explore ourself, find information ourself, and even that's the case, it does not actually mean that we will 100% understand it. We will still need some guidance. When we actually go into a workshop, we are given a case to solve with the knowledge that we have studied. We have something to apply on, and then when it's a discussion, there's a discussion going on with your friends, you can share knowledge, and you can help to clear some doubts that you have, and then the lecturers will come in and explain to you why is this the case, and then you will have more of [inaudible 00:12:50] understanding of the topic.

Lecturer B:

Anyone else has anything to add on this?

Student A (Focus Group 4):

I think it's also more in lectures, people tend to be more shy, and they don't want to approach the lecturers. Whereas, in the workshops, you're broken down into smaller groups and then your lecturer is just focused more on that group so you feel more confident to ask them questions. That's why you feel like you understand better.

Lecturer B:

All right. Yeah. [inaudible 00:13:35] you have anything to add, if not [inaudible 00:13:37].

Student B (Focus Group 4):

Yeah. No. I agree with Student C. I think that doing discovery before lecture, I think it helps. But when you study a discovery, it's not 100% ... you no 100% understand the content fully. But during workshop, it's when you can actually test your understanding, and see if you really understand the concept.

Lecturer B:

All right. [inaudible 00:14:08]. I'm going to start sharing [inaudible 00:14:09] remaining questions [inaudible 00:14:12]. Yeah. Back to you Lecturer A.

Lecturer A:

All right. Thanks Lecturer B. I hope I'm coming across clearly. We're just moving onto the next question now. Taking a pause now and looking back at the virtual learning experiences that you've had so far since we all went virtual, was there anything that you all felt that was hard to learn virtually?

Lecturer A:

Or any learning activity that you all felt that was ... it was just hard [crosstalk 00:14:48]- Yeah. Student B.

Student B (Focus Group 4):

I think it was okay, but I feel like for me personally, the hardest challenge was to stay focused. Because even before COVID, we did most of our study in discovery, they were all online anyway. Yeah.

Lecturer A:

Yeah. True. Thank you. And Student C?

Student D (Focus Group 4):

I find it very hard to do group work.

Lecturer A:

To do group work.

Student D (Focus Group 4):

Yeah. Because it's we had to find time. I mean I understand everyone's in a COVID situation, like where else can you go? But everyone has their own personal methods. When you're a uni setting, it's kind of like okay, I see you face to face. You can see from my expression that this is really important, we need to get this done. It's harder for people to avoid that responsibility when compared to online, people can just not reply your text, or just not contribute to the work at all. I think that's one of the hardest challenges as well for me.

Lecturer A:

Thanks Student C. That's a really good point. Thanks for sharing that. Student A, what about yourself?

Student A (Focus Group 4):

I think mine is more of accessibility to lectures, because if you're face to face, you can just drop by the office, and then they will be in and then you can ask all your questions. And then whereas online, you have to send them an email, and then you have to wait for them to reply back. Yeah.

Lecturer A:

Yeah. Understood. Yeah. Thank you. Thanks for that. Any thoughts as well on the aspect? I don't know whether if you all had the opportunities to explore this [inaudible 00:16:37]? But some of the groups of students had issues with learning medical devices and lab activities and doing this virtually. They commented that this was something that they found that to be quite difficult to follow.

Lecturer A:

Do you all have any thoughts to that?

Student D (Focus Group 4):

This Thursday, we're actually going to uni to do lab work, so I think that can be resolved. But in the case of the medical device, we actually got that done year two in the physical setting, so I don't think [crosstalk 00:17:15]-

Student A (Focus Group 4):

I don't think it's applicable. Yeah.

Lecturer A:

Yeah. All right. Thank you. But I guess the point on the lab, yes, there is a session happening this week. But even if I take like today onwards and I just look back in the past 18 months, so ever since we went virtual, you had some lab sessions [crosstalk 00:17:38] which were-

Lecturer B:

They got to do lab. The year two [crosstalk 00:17:40].

Student A (Focus Group 4):

Yeah. We got to do.

Lecturer B:

They did it live, so I guess fair enough, you're right that you did get a bulk of the lab experiences I think in that sense.

Lecturer A:

Okay. All right. Thanks. Thank you for that. Now, I guess we'll over onto the next question then. The next question, so on this idea of flexibility, so in something of the earlier focus groups that we conducted, students commented that the virtual learning environment has afforded them a lot of flexibility, and they liked it very merchant, or they seem to enjoy this degree of flexibility. We're just curious now from your perspectives, how important is flexibility to you for your overall learning experience?

Student A (Focus Group 4):

I would say for me, as moderate importance to me. Because if I'm too flexile, I tend to just ignore my work. If I'm too constrained, I get too stressed to the point I can't think properly. For me, it's moderately important flexibility.

Student B (Focus Group 4):

But for me, I feel having flexibility is good, because it gives us the freedom to focus on other stuff as well, rather than just education. You have the free time to explore other aspects of your life, because we will have to prepare for life even after our degree. Having flexibility allows us to do that, which is good.

Student D (Focus Group 4):

For my case, I need very high flexibility, because I know that I can [inaudible 00:19:37] in what I need to do, like I need to get my uni work done. But at the same time, there are a lot of things that I need to be flexible with in terms of my personal issues, personal commitments. I really need flexibility, whether or not how I deal with it, I will know that I will get things done. But I just ... on my own pace I would say.

Lecturer A:

Sure. Okay. Thank you. I think that answers the question for us really as well. The next question that we have essentially ... I don't know whether okay ... some of you have the opportunity to come back to campus just to stay. Some of you may have some more future opportunities later on, and then we

were just wondering what are your feelings right now towards coming back to the campus post-COVID or in the middle of this pandemic right now?

Lecturer A:

Any feelings or what do you feel about it? Were there any concerns already or you're excited? Or no feelings at all? Let's just get on with it, kind of thing, or ... Yeah.

Student D (Focus Group 4):

Definitely excited, because we do get a chance to not go and just watch it virtually, but I'm not actually excited for that. I'm just excited to be with my friends and be in the university setting maybe one last time. Yeah.

Student B (Focus Group 4):

Yeah. I agree with her.

Student A (Focus Group 4):

Yeah. [inaudible 00:21:19].

Lecturer A:

Okay. Thank you. Any concerns though? Any feelings of anxiety or nervousness or anything of that sort of or not? Nah? Okay.

Student D (Focus Group 4):

Nah.

Lecturer A:

Fantastic. All right. Thank you. No, definitely from our end as well. We're also looking forward, and we do hope that everything can just play out smoothly and fine as well. The next question that we have, we're just wondering whether if there's ... because you've had quite a bit of substantial virtual learning over this COVID pandemic, just wondering whether do you think that this has any impact on your future career prospects or for your life beyond uni?

Lecturer A:

Student C's nodding her head. Do you care to leverage a bit more?

Student D (Focus Group 4):

Yes, definitely, I think it shows everyone that you don't actually have to be physically present to work. You can always use online. It made me see how much this internet has impacted everyone globally. I believe that this is the future. Maybe in the past, everyone's just using online shopping, but now it's so profound. Everyone just does it. You can say you can just go restaurant next door and they say, "Oh, no, because I get vouchers online." And people would prefer to do that.

Student D (Focus Group 4):

In a way that I would actually like to explore more online careers, or any job that would eventually incorporate that, because if a company constantly just stays where they are, just physical, physical, then I feel like eventually, they will just get terminated. Yeah. You got to keep up. Yeah.

Lecturer A:

Thanks Student C. Student B?

Student B (Focus Group 4):

Yeah. Most companies are also starting to introduce hybrid working, so they'll combine virtual working and physical, so I think yeah, the pandemic has really changed a lot of things in terms of working environment.

Lecturer A:

And Student A, any thoughts?

Student A (Focus Group 4):

I think for me, it would just show that some things can be actually done from home, where you don't really need to be present somewhere to do it. Hopefully, moving forward, places that potentially I will work at can see this, and then maybe have a blended environment.

Lecturer A:

All right. Thank you. If I can just briefly summarize as well. It seems that the vibe that I'm getting is that you seem to have realized that there are other opportunities to explore, because of what you've gone through. You are fairly optimistic about your future career prospects or not?

Lecturer A:

In terms of this feeling of optimism.

Student D (Focus Group 4):

Yeah. I'm quite optimistic about it.

Student A (Focus Group 4):

Yeah.

Lecturer A:

All right. Yeah.

Student B (Focus Group 4):

I think there's a mix between optimism and anxiety. As much as I am optimistic about it, I can still sometimes get quite anxious about thinking about the future. Yeah.

Lecturer A:

Okay. I guess Student B, do you mind elaborating a little bit more? This sense of anxiety, what were you anxious about?

Student B (Focus Group 4):

I used to be a lot more anxious, but recently, I've grown to be a lot more optimistic. Because recently, I've joined a lot of talks where people from different fields ... pharmacy graduates from different fields, they just come in and they give talks about their career progression. It made me realize that everyone has to start somewhere. Yeah. I'm a lot more optimistic now rather than just anxious, but sometimes I just can't help it, because what if it doesn't work out? I guess it's human nature to worry.

Lecturer A:

Okay. All right. Thank you. Thanks for sharing. Sorry, Lecturer B, did you wanted to say something? Yeah.

Lecturer B:

Yeah. Your views are good. They're quite different from some of the views of some of your juniors. You see your two years of online education as some sort of advantage in a sense, like the fact that you have two years offline, three years online, do you think that this is kind of some sort of advantage to you?

Lecturer B:

I think that, that was kind of one of the cores of the question that was the two years online versus the two years offline, were you afraid whether the online part was going to affect your working life in any way or not? But I'm so glad to know that you actually kind of saw it in a more positive light then. It change our perspective a little of how world is going to be post-pandemic in that sense. But back to the original thing, were you ... But in general, in the two years of online education, did you feel that this would have affected your work in any way? Or not really?

Student B (Focus Group 4):

I actually think it's an advantage, because not only did it give me more flexibility. It also made me more used to working online, to prep myself for working online in the future, so I guess that's a good thing. Yeah.

Lecturer B:

Cool. Sorry. Yeah.

Student D (Focus Group 4):

For me, I think it's good, because I get to learn things besides pharmacy, which I think ... I was doing out of interest. I was studying other books and being a nerd, and then I realized how beneficial it became when I started going for interviews for jobs. They were just asking me, "What have you been doing during pandemic?" Then I was just sharing them some of the extra things that I did besides uni. It actually did help me to ... it can show that I have some kind of diversity in terms of my life, which more knowledge is never not beneficial.

Student D (Focus Group 4):

I think it still all comes down to more freedom and flexibility, which allows me to do that.

Lecturer B:

Yeah. I think that's good. Unless you have anything to add Student A, or [crosstalk 00:28:51]-

Student A (Focus Group 4):

Not really, no, I agree with them.

Lecturer B:

All right. Fantastic. Back to you Lecturer A.

Lecturer A:

Thank you. Just few more questions, then we'll be done for the afternoon [inaudible 00:29:03].

Lecturer A:

This is a bit specific and this is about OSCE and it can be commercial question as well. But we just wanted your frank thoughts on it. We've gone through a few rounds of virtual OSCE already, and you've also had the privilege of going through a face to face OSCE as well. Thinking about your virtual OSCEs, over the course of all the virtual OSCEs that you've gone through already, do you notice whether if there's any big changes? Whether in a positive direction or in a negative direction or even just neutral across all the virtual OSCEs that you already take part in? This is just thinking first about your virtual OSCEs.

Student D (Focus Group 4):

Were there any changes?

Lecturer A:

Yeah. Did you notice any changes or not?

Student D (Focus Group 4):

No.

Student A (Focus Group 4):

I didn't notice as [crosstalk 00:30:06].

Lecturer A:

No problem. Sure. That's fine as well. Yeah. And Student B?

Student B (Focus Group 4):

Yeah. No. No changes.

Lecturer A:

Okay. All right. No problem. It's just a constant feeling towards it, overall the virtual OSCEs if I can summarize that. Yeah. Okay. All right.

Lecturer A:

And then thinking now also on your virtual OSCEs that you've really take part in, do you think that it helps prepare you for your life beyond uni or for your future practice?

Student D (Focus Group 4):

Honestly, no. I feel like it brings a lot of anxiety.

Student A (Focus Group 4):

Yeah.

Lecturer A:

Okay. I would like to hear your thoughts now. Maybe we'll start with Student A first. Why do you think that is? Why do you say so?

Student A (Focus Group 4):

I think it's because a virtual OSCE, I can always just [inaudible 00:31:04] of the Zoom screen and then look through my references, and I can be confident while giving out counseling. But whereas if put me in a situation like face to face OSCE, I don't have access to that, and I have to maintain eye contact as well. It's like you tend to get more and more anxious. Yeah.

Lecturer A:

Okay. Thank you. Student C, any thoughts?

Student D (Focus Group 4):

Okay. Understand that this time limit thing, where it trains us to give out a recommendation ASAP, but sometimes the case is super long, and then the patient is required to speak very fast, and they give us all the knowledge, and things like that. I feel like there needs to be some alteration in that. I'm not sure about everyone, but for me when I have this OSCE exam, my mind isn't really much about doing it for my future. It's more like I need to pass this. I think that's the wrong mindset, but that's the kind of feeling that I get from OSCE. Yeah. It's not natural to me and I feel like working part-time in a pharmacy gives me a better environment to learn how to do it in real life, rather than OSCE itself.

Lecturer A:

Thank you. Those are some really good points there Student C. Student B, any thoughts?

Student B (Focus Group 4):

Yeah. I completely agree with her. I feel like what I gained from OSCE is basically just how to work well under pressure. Yeah.

Lecturer A:

Okay. Thank you. Do you think that's a good skill to learn, working well under pressure?

Student B (Focus Group 4):

Yeah. Definitely.

Lecturer A:

Sure. All right. Cool. Thank you. Thanks for that. I think we're onto our last question already, unless, Lecturer B, do you have anything else that you want to ask or clarify?

Lecturer B:

I'm good. Thanks.

Lecturer A:

Okay. All right. The last question, I don't know how you want to ... I would still be interested to hear your thoughts, but then I guess some of your juniors had a lot of things to say about this. But essentially what we were just curious if for those who have gone through an online learning environment, and let's say if you're going to transition back to an offline or a face to face learning environment, was there anything that is good that happened in the virtual learning? Or in the online learning environment that would like to be carried through in an offline learning environment?

Lecturer A:

I know I guess most of you all here are towards the later part of your program already. But I guess if you had a chance to contribute back to the future of how pharmacy could look like, what do you think ... was there any good things that happened in the virtual learning format that we could carry through into an offline or a face to face learning format?

Lecturer A:

I guess while you're thinking about it as well, I guess you could also imagine yourself that, okay, let's say if you are going to become a student next year, or year in the future, again, what do you think a nice pharmacy graduate program would look like? What could it have? Are there elements that from the online learning, which we can continue on and incorporate into our offline or our face to face learning settings? That's really what the question was asking about.

Student D (Focus Group 4):

Honestly, I think the adaptation for online learning is only flexibility and being five seconds away from my laptop. Beside that, I don't really see much of an advantage to it, because I think it brings more disadvantage than advantage. Yeah.

Lecturer A:

Sorry. Student C, could you repeat that last sentence? It was [crosstalk 00:35:21]-

Student D (Focus Group 4):

I think online learning brings more disadvantage than advantage for me.

Lecturer A:

Okay. All right. Thank you.

Lecturer A:

The other two, any thoughts?

Student B (Focus Group 4):

Yeah. I agree with Student C. I feel like if possible, most of us would really like to return to physical learning as soon as possible.

Student A (Focus Group 4):

Yeah. I feel like just offline content was just open to make it online, so whether when we go back to offline, it doesn't really change, nothing can be brought for me.

Lecturer A:

Okay. All right. Thank you. I think that's pretty much what we have for the three of you, or for us this afternoon already. Just some closing remarks or some comments from the three of you. I guess was there anything that we've talked about in the past half an hour that stood out to you this afternoon? Or anything that you just wanted to highlight or you just wanted to focus again or shine the light and say that this was a really important thing that we talked about this afternoon? Was there anything?

Student D (Focus Group 4):

I think they were all equally important.

Lecturer A:

Thanks Student C. Student B? Yeah.

Student B (Focus Group 4):

I think that it made me realize how most of us actually have other priorities rather than just focusing on education. Yeah. The importance of virtual learning is actually highlighted there.

Lecturer A:

Thank you. And Student A, anything?

Student A (Focus Group 4):

I think mine it was the last question on how maybe some elements of online learning can be integrated into offline, I actually never thought about that. That one actually just did get me thinking. I think that was the more important part for me.

Lecturer A:

Fantastic. All right. Thank you. Lecturer B, any closing comments? As I need to just quickly check, I think there's a cab driver outside my house. I'm sorry. Lecturer B, can you continue?

Lecturer B:

Oh, okay. Yeah. No worries. I'll take over. Actually, that more or less is the end of the session.

Lecturer B:

Actually, I just had one question that's not exactly related. But do you all find [inaudible 00:38:35] harder than [inaudible 00:38:35] or easier? Because I think Student C you said that's the hardest time you had. I always thought year four [inaudible 00:38:45] was tough, because you have exit OSCEs, you have [inaudible 00:38:49].

Lecturer B:

Yeah.

Student D (Focus Group 4):

Maybe I haven't finished my second term this term, so I don't know. Maybe when the OSCE comes, then I might change my mind. But in terms of if we're going to disregard OSCE, because I think both have OSCE for both years, so in terms of the course-wise, I was thinking that distance is easier.

Lecturer B:

[inaudible 00:39:12]. Sorry. I asked them whether they thought year four [inaudible 00:39:14] or year [inaudible 00:39:22] was harder. I think Student C year three [inaudible 00:39:23] was harder. For Student A and Student B, you feel the same?

Student B (Focus Group 4):

Yeah. Because I feel like for this semester, most of the knowledge we already have anyway, so I definitely think year three was harder.

Student A (Focus Group 4):

Yeah. Currently, because I'm in year three [inaudible 00:39:42] two, I think like the anxiety of year three was the hardest year.

Lecturer B:

Okay. I was curious to know how your experiences ... I guess in my case, [inaudible 00:39:56] was the toughest and year four [inaudible 00:39:58] was quite light. Yeah.

Lecturer B:

Okay. I'm done on my side. And the final question was not really part of the interview. I guess with that, we're kind of all done with it, unless you have anything else to add Lecturer A. If not, I guess we can dismiss the session. Thanks a lot for joining us for this. Have a good summer break and good session this Thursday. All three of you are going this Thursday?

Student A (Focus Group 4):

I'm one year behind. I'm one year behind.

Lecturer B:

Oh, sorry, sorry. Yeah. For Student B and Student C, both of you are heading over?

Student D (Focus Group 4):

Yes.
