# Supplementary material for: Pharmacy students’ perspective on remote flipped classrooms in Malaysia: a qualitative study
Source: J Educ Eval Health Prof. 2025 Jan 14;22:2. doi: 10.3352/jeehp.2025.22.2 (PMC12055608; doi:10.3352/jeehp.2025.22.2)
Supplement: Supplementary file 2 [file jeehp-22-2-dataset2.pdf]

Dataset 2

Deidentified Field Notes FG1Y2 - Sept 2020

What do you feel about the online experience?

Student A – online experience quite ok cause quite flexible – can see videos multiple times

Workshop hard cause members can be unresponsive, and

Student B – can self record lectures too, not so good – hard to reach lecturers, can be difficult

Feels that it can be weird in workshops cause can't see a person there, the hand gestures and all.

Speed of responses not good, no access to exam answers (feedback), during online less accessible

Student C – online more time, cause no need to travel – flexibility. Taking pictures of slides easier, can screenshot

Student D – lectures online is pretty cool, but workshops online is not good, very difficult, and if line drops its difficult, then line drops its hard cause you'll miss a part and then hard to ask for repeating.

Difference offline (yr1) and online (yr2)

Student B – can get immediate feedback from an offline class, exams are not very useful, cause all the answers are in front of us, might not benefit us. Can have 50 other tabs for exam questions

Student D – online exams especially OSCE not good, feels its not exactly how OSCE should be done, in sem exams , should evaluate how well we learn, open book exams are not so useful, don't really evaluate what we learn through the year. Online assessments are a contest on how fast to find answers.

Student A – assessments not fair, cause they can work together and corroborate

Student C – osce online not good experience, cause patients connection got disconnected, and was stuck in room for 8 minutes alone, and 2nd time time also half disconnected patient. Our assessments, not fair, most people can just search for all answers, so not fair.

Comparison – online offline IL, CTL, Workshops

Student A – online interactive lecture, actually can ask questions quickly and anonymously, more efficient than a lecture hall. For pre-recorded, using Australian ones or last years are not good, cause they may say quiet down or random things.

Student B – sometimes lecturer mentions your name on anonymous asking, not so fun. Hard to record lectures.

Student C – flexibility of doing whenever you want, pre-recorded one lose the element of collective answers from friends.

Student D – prefers online lectures

Workshops

Student B – breakout rooms wrong can cause panic, the links can be an issue if it changes. Sometimes I may leave the session during workshop

Student C – most students don't wanna open the camera and mike, and prefer to type in google drive. In physical we can discuss it together well. Workshops can exceed time often during online implementation. Sometimes the actual workshop not being done can cause issues with parents.

Student A – communication issues in workshops.

Student D – online workshop hard to learn practical devices (inhalers, insulin pens), placement places can be well equipped or not, this can affect performance in OSCE. CTL sessions are quite similar to IL, and she feels CTL is the most useful session, it might be better to do CTL online, as its easier to ask questions.

Organization of the online flipped in sem 1, 2020

Student B – sudden lectures, too sudden there is a lecture, in terms of timing, the timetable changes too often,

Student D –

Student C - Zoom links are all clearly stated, last sem lots of different emails.

Student A – sem 2 was way more organized, timetabling, and all is way better, sem 1 was bits and pieces being revealed suddenly.

Offline to online – what were your main concerns

Student B – internet issues, connection is a big problem that can't be taken lightly, still persists

Student A – extempt labs replacement.

Student C – technology barrier, using zoom, now used to zoom its quite ok liao. Even lecturers could not record or use, wasting time, and now its good, resolved.

Student D – mainly schedule, zoom resolved. But the exams are not resolved, its not fair.

Assessments online

Student B – can drink, or eat during assessments, can use machine learning to search answers

Student A – the home use of hardware is nice, at home temp etc is good. Could be less fair, cause cursor at start of document, and students don't play the role, in real life that won't happen.

Student C – benefit of online assessments – using own laptop easier to type, more stress in the MMDAC is more stressful. May not be fair on online assessments workshops

Student D – when typing assessments essay, connection issue and a red bar came out, and worried that answer may not be updated in system. She likes the MMDAC environment, online exam at home the environment is too comfortable, not good, less stress but not so good for me, dislikes online exam.

Assessments are they a good measure of knowledge and skills

Student B – online learning teaches new ways to abuse tech, but makes us learns less

- No need drawing online so can't do that

Student C – helps cause marks up and teaches how to search

Student A – not an accurate representation of our marks, depends a lot on trust, for her she takes it serious cause she know its for her own good. Worries on what the CGPA really means cause it was all done during the online era.

What do you think of the pre-class activities

Student A – discovery can be overwhelming, but good to have something before the lecture proper, overall pros more than cons. If we don't do discovery can't get anything from lecture. Optional readings should be highlighted, cause may read unnecessarily things that can be confusing

Student C – like discovery, last time don't study before class, now with discovery as we study it gives us a baseline so it helps to link back during lecture. If directly go into lecture, it will be harder. Sometimes the link has too much info and its too detailed, and they dunno what they need to know. Should embed it into the discovery narrow down what is important.

Student B – sometimes lecture can be too easy and don't need to read

Student D – length of discovery is different, but given same time frame, sometimes too much things, too little time. May not know what the content we should focus on during discovery only gets it during IL, so she thinks that its best to have IL first before discovery (to identify important points to know)

What do you think of in class activities

Student A – Conclusion after workshop is better, during CTL may not always address the workshop cases. If during workshop not enough time, sometimes lecturers just say ok time to leave and go off.

Student D -

Student C – workshop briefing too short, some don't even go through the case. Should have discussion on workshop right after workshop instead of during CTL. Feels IL is quite good, cause directly link into the discovery, but would like more examples that link to reality.

Student B – like close the loop sessions – cause it's a recap of the topic,

How does flipped compare to traditional classroom

Student B – feels that flipped is okay, but feels that for example lecturer not answering, then no point giving good discovery material but never answer. Quite indifferent

Student A – last time was study by the book, traditional into flipped was very hard, cause very confusing, but slowly got used to it, good cause get to work in groups and can discuss. I would prefer if the syllabus has more Malaysian context.

Student C – a lot of presentation skills things, and a lot of on the spot learning things, do a lot of interactive and communication skills, more interactive she likes it.

Initial feelings compared to now (why)

Student C – in the beginning, didn't know what all the things are, in the discovery and all. Did not like clicking next next in discovery,

Student A – have to accept so she adapted and

Student B – originally very pessimistic, had a flipped experience last time, and still pessimistic. Was expected.

Student D – same with Student A adapt cause no choice

Flipped learning on units – useful for all units? [think they didn't get the question]

Student B – we have too little lab, haven't done lab at all, felt that pharmacy would have a lot more practical

Student A – might

Would you choose flipped or traditional if you had a choice

Student D – no opinion

Student A – would prefer flipped

Student B -

Student C – only experienced flipped, so hard to comment on others

Compared to pre u and high school

Student D – compared year 1 its good, compared year 2, not so good.

Student B – year 2 more interesting to year 1, pre- university was more chill, and easier. (did A levels).

Student C – Science was in Malay SPM, so pre-u had to learn all in English, then book to online. Overall online is ok, cause more convenient, handwriting not good so can use autocorrect is good.

Student A – can learn all from books, and can get all in books, now in degree, its more application, due to learning style, more fun, less boring, if can do this in high school, would be more beneficial.

How many hours for discovery materials?

Student B – 3 rounds of discovery, 1 hour first round, 2 hours for 2nd round, 1.5 hours for 3rd round (5 hours is good, 8 hours 2 subjects).

Student D – depends on how someone studies, depends on topic and content, can vary from 3 hours, to 3 days.

Student A – depends a lot on materials for PP sometimes its very easy, sometimes very technical so might have to read word by word, and attention span matters.

Introduce flipped in the beginning or better to introduce later in uni?

Student D –

Student B – the online is a bit of a letdown,

Student C – better to start in year 1, if start later, then you need to spend time later to get used to it, so might as well start in the beginning.

Student A – ok to intro in yr 1, but a bit overwhelming immediately, especially moodle.

Does it increase adaptability

Student A – yes, also being the fact that can't change. Initially really disliked, and out of the comfort zone, and now adapt, shifts mindset to adaptation, become more positive in that sense.

Student D – Yes, we can't change anything so adapt

Student C – yes

Student B – neutral, personally doesn't actually changed much.

Recommendations for the flipped program

Student A – placements - allowance for placements cause some are very far, if can't swap its very difficult

Student B – placements can be very far,

Student C – hard copies of APF not enough copies (better access to resources).

Many more STEPS – is this good or bad?

Student D – like, but depends on location, or the pharmacy branch

Student B – neutral

Student A – like good early exposure, see how pharmacy role in real life setting

Student C – like, most important can experience

Most important thing we discussed

Student B – CTLs are happening long time ago

Student C – Recommendations are quite good

Student A – not really

Student D – nothing really surprised me

Flipped learning on units – useful for all units? [think they didn't get the question]

Student B – we have too little lab, haven't done lab at all, felt that pharmacy would have a lot more practical

Student A – might

Student B – flipped learning

Student A – maybe the workshops could be more

Student C – CC can have more IL (cause not enough initial explanation), for PP maybe can have less workshop, lectures, some workshops are useless

PLP

Student A – concern on PLP, assessment not fair, cause lecturer decides what is good or not good, its not fair, fairness of interpretation.

Deidentified Field Notes FG2Y2, Sept 2020

UCSI teaching ourselves, search and then research and present in class.

ONLINE PART

How has online learning experience been so far

Student A – prefer physical classes – cause can ask questions on the spot, easy to clarify, and can see person talked to, also feels that its good for lecturer can't see students. But acaddemically not much difference .

Student B – generally agree that asking face to face is easier, but online has more time, save travel time, can utilize the time in between well when online cause gap times are not 'lost'

Student C – quite similar to Student D and Student B, saves time on travel, online classes encourage questioning, feels lecturers more active in answering questions. But still prefers physical classes.

Student D – online has one benefit, can take screenshots instead of pictures, now online can take screenshot instead of blurry pictures, can see every single page its less overwhelming. But easier to ask face to face questions.

How Online compare to offline

Student A – Physical class easier to ask questions, cause the ILs are recorded, so if got question need to wait till the end of recording, then go email or something to ask

Student B – Easier to explain a question, can point at a specific thing, and also act on the answer immediate. But online when asked questions can use chat to answer.

Student C – During workshop its easier to ask the person on the spot, but in online mode, need to keep switching breakouts its more messy. Prefer some parts of online cause convenient to ask questions via email.

Student D – For me, I take time to think of questions, so online is good, but to clarify concepts its easier to do physical class.

Online IL, CTL, Workshop

Student A – After physical workshop, IL can stay back and ask and people do this, online mode everyone just goes off after session ends. Prefers live ONLINE one vs. any recording.

Student B – Some pre-recorded ILs can be too short and not much content, as a disadvantage.

Student C – Workshop online more messy, breakout sometimes they will monopolize the lecturer. Hard to know when to ask. Prefers a pre-recorded and also wants a live session to ask questions.

Student D – online workshop can be hard to discuss, cause of line issue can also be nervewrecking on assessment workshop. Last sem CTL can be quite long, but improved this sem. Prefer pre-recorded IL but wants a CTL type one to ask questions. Prefers broken down into sections not as a whole.

Improvement Sem 1 to Sem 2

Student A – Significant improvement in terms of organization.

Student B – Zoom much better in sem 2, last time very confusing cause was via emails, now its all centralized in a google docs.

Student C – Now lecturers also know how to maneuver around zoom which helps.

Student D – Improvement in terms of organization, and also now focus on forum discussions, its useful

Initial concerns when initially switching offline to online

Student A – very disappointed initially, but also happy that it was going to be offline cause can study from home (sabah,Sarawak). Also worried on cost and hostel accommodation. Also worried about workshops will be done, cause knows a lot of students will be shy to on the webcam. Overall, now feels that some of these have been overcome not as bad as expected, but still prefers physical.

Student B – Concerts on labs, workshop, placements. By now can see that workshops overall ended up not bad, and placement and extra sessions help. For OSCE can be a technical challenge, like construction, or issues with disturbance. This can't be predicted sometimes, cause it disturbs psychologically can be nerve wrecking

Student C – Cost was a big issue, cause got coursera or any other online modalities, so why pay this much for online class. Worried on labs and OSCE, may be disadvantage in hospital or anything. Feels that F2F OSCE is the best. Workshops also an issue cause not everyone responds, can affect teamwork and communication. OSCE must see the camera, feels its an issue, feels that lecturer may suspect cheating based on behavior.

Student D – main concerns, how labs/lectures would be carried out, cost issues, and also likes physical notes a lot so hard to annotate. So for classes its rather ok, cost no change too bad, now using electronic notes so more organized. Since OSCE has very strict timing doesn't allow for buffering time for connection or technical issue.

Feeling on assessments

Student A – no isolated space to do exams/assessments, so it is a bit issue with assessments OSCE. Connection is another issue, esp OSCE, will be more nervous cause of the panic of connection. Another thing is that it may not measure your head knowledge, but may be good cause it does measure your ability to search and find good info. Prefers written assessments and F2F OSCE. Zoom OSCE is less intimidating. Main unfairness is environmental, but feels that its fair to be able to utilize resources.

Student B – In terms of marks its good cause can refer, but in terms of disturbance it's a disadvantage. Doesn't measure knowledge well but measures how effective at finding resources. Not much time to refer to things during the open books exams.

Student C – good that can refer for quizzes, but time limit makes it hard to refer. Also harder to do group study during the online thing. Most

Student D – same points as Student A and Student B. Maybe some unfairness due to references.

Technological challenge

Student A – sometimes webcam issues, or mic issues, which can be an issue.

Student B – In case of hardware failure, no backup, can be risky.

Student C – hard to ask online on certain things, especially if there are many

Student D – can have issues with hardware redundancy. Attendance can be lower online cause can refer to recordings, maybe did not get full benefit of lectures. Sometimes hard to put questions acStudent Cs, an example of a rubric for a CC unit, the implementation can be an issue. Cause offline one could just fill and exchange, but online each must submit individual rubric and did know know as a patient or pharmacist.

Pre-class materials

Student A – Like cause can do beforehand. Don't like cause can be incomplete, or confusing, and teacher may not teach it clearly. Very much concerned with the confusion of discovery and the fact that its not clarified even after the next year

Student B – Also concerned with the confusion, gave examples of fiber on IBS, it looked like its bad

Student C – Likes the general format of discovery, IL, workshop, CTL. Feels that discovery has too many links. Sometimes notes can be too confusing, for example diabetes, in clinical practice guidelines its organized, but in discovery its confusing.

Student D – Discovery can be good, but to improve cause too many links, sometimes not sure what to read, should try to clarify what needs to be read, and what is extra. Also discovery can be misleading, and sometimes its downloaded beforehand and then did not correct. Sometimes video content has issues with accent, or understanding the content. Best to have both notes and transcript.

In class activities

Student A -

Student B – Access to different workshop scenarios can be an issue, if can't see other questions.

Student C – Sometimes workshops don't give the answers. Prefers having scenario and answer for both sessions. Feel can put all the workshop materials in moodle link. Can consider giving feedback on the student answers if don't want to give answers directly.

Student D – Likes how workshop tests understanding, but the wrap up session can be improved. Prefers shortwrap up directly after workshop, then can do the follow up at proper later CTL session. Sometimes the students answers are all different but not explained what is correct.

Contrasting IL from traditional lecture

Student A – prefers live IL, but online IL not as interactive. Can't see face hard to guage level of understanding.

Student B – IL may be too hard in terms of answers to questions. Initially not very used to IL, but what she likes it that it tests them on it early so we need to understand it well, or at least tells us what we don't understand.

Student C – Likes zoom polls instead of poll EV.

Student D – Most previous experience is 1 way type, helps focus studies, prefers interactive lecture to one way traditional. Likes Zoom polls.

Initial feeling on Flipped and how long to adjust

Student A – She is used to the lecture type system from pre-U. In Monash its more interactive, and workshop is more applied scenario, may be more applicable to real life.

Student B -

Student C – had a bit of issue with adjusting to online initially, but also now adapted.

Student D – Initially took one time to adjust 1 sem. Year 1 is discovery IL, break, workshop CTL. Prefers year 2 system

Flipped useful for all kinds of units?

Student A – Feels that this system is useful across all units.

Student B – Also feels that the system its useful,

Student C – Feels that workshop for PP it may not be that useful, in our workshop format.

Student D – Generaly useful for all units. For specific subjects like systematic review, can be a bit confusing in implementation.

All 4 prefer to use flipped style for undergrad.

If this style continued next year, what would you think should be done

Student A – can't really think what to improve.

Student B -

Student C – Usually can ask directly to lecturer on campus, and sometimes hard to type questions, suggests to have 1-1 zoom sessions with lecturers.

Student D – Some uni, can do office hour Zoom where students can come in and ask questions.

If next year offline is re-introduced, what would you keep or hybrid from the current system? Anonymous forum is too messy to study, cause not organized. If streamlined, may use anonymous forums even more than zoom. Needs guide for year 2 placements

If offline returns next year, would you keep anything from now?

Student A – Should have all the recorded lectures there,

Student B – Can continue extra sessions online via Zoom

Student C – Sending slides via zoom, which are clearer as compared to offline pictures

Student D –

Does flipped make you more adaptable?

Student A – improved time management, especially since online classes start. Learned to speak up more during lectures. Especially online must speak up more often.

Student B – makes us more independent to study ourselves first.

Student C – Time to read discovery makes her more detailed and focus, cause the day dedicated to discovery makes it enforced to study.

Student D –

Most important thing talked about today for you

Student A – Interaction and communication is important, should speak up more

Student B – more or less said.

Student C – highlights issues students are facing and what measures to improve the studies, and difference between online and physical. How to prep for next year.

Student D – See point of view of lectures and

Feedback on the fact that we don't give feedback directly on assessments. Given more generic feedback.

If everyone did wrong, or most people did wrong should go and correct those parts.

Deidentified Field Notes FG3Y3 - Sept 2020

What do you feel on the online experience?

Student D – Sem 1 was better, sem 2 very blur for online. Needs a lot of discipline. Exam wise, a lot of issues, technical issues, very chaotic, only way to retake exam, and basically that was kinda bad.

Cons – confusion, emailing/forming is easier said than done, hard to phrase through text, forums very confusing, not organized. Sometimes forums must wait for response. Very confusing, overall, sometimes they just give up and do it on their own.

Pros – flexible timing, being with family, no need to go out, have more time,

Student C – a lot of unnecessary assessments, iRAD and tRAD

Student A – technical issues, issues to ask questions (understanding what the question is), F2F questioning is easier.

Student B – in sem 1, was implemented better (convenience), in sem 2, worse implementation

How do you feel about IL, CTL, workshop

Student D – sometimes people like to stay muted instead of contributing. And hard to tell them to contribute. Sometimes workshops drag a long time, time management poorer. Easier to ask questions in offline IL.

Student C – iRAD and tRAD redundant. Sem 1 IL are pre-recorded with sections. Sem 2 ILs are Live more hectic.

Student A – Shy people don't contribute in discussion, and this is worse online. Maybe if assessed ones can get everyone to on video may be better.

Student B – main downside is the breakout room, cause monopolized by others. But otherwise being with your own group online is actually more focused, less distraction. Clearer to hear questions. For the groupwork part, sometimes people are shy, even if they stay muted and do work. Timing can be quite tough online.

Organization of the online experience

Student D – sem 1 more hectic, but in sem 2 its hectic cause confused not cause workload. Discovery this sem, has key concept tasks which are not fully discussed, CYL is enough to check our understanding, iRAD and tRAD

Student C – IL in sem 1, better than sem 2.

Student A – iRAD tRAD too soon to be done after discovery, cause didn't understand and immediately tested. ILs now a bit too narrow, only covers 7 questions, doesn't cover the discovery well enough.

Student B – sem 2 timetable more organized than sem 1, and being a class rep its hard.

Initial concerns online and now

Student D – Cost issues is there discount on course, economic difficulty, coping with online was a worry. Realized that uni is quite self driven, so learned to self regulate. But financially its still hard. Was very active in uni, so now that its online can't do these and its an important escape from stress. So now alone in room, was very hard to cope mentally and physically. Also time management is worse, cause now too much time, hard to regulate. Took a toll on general health. In sem 1, worked super hard and super stress, and results did not reflect the hard work put in. In sem 2, started to do other things besides studying, and put other activities actually helped to appreciate and can manage time better.

Student C – financial concerns, what about classes initially, resigned to adapt to zoom type classes. Learned that finances are a bit more complex and also learned to accept.

Student A – missed out a bit -

Student B – Felt stressed cause she is the middle person, so felt that timetable not clear, so she has to communicate a lot in between, and organize. In sem 2 fixed time tabling helped a lot.

Research online, how does it feel?

Student D – Initially, everyone was very blur. Feels its useful for those who are interested in research initially. Does some surveys, on the side, and after placement in caring pharmacy found that Caring does a lot of evidence based things, and feels that's why research is important. Main point not everyone is meant for research.

Student C -

Student A – Feels that its useful in the long run – feels that it came out of nowhere. Not used to it cause different from previous units, feels a disconnect. Feels its 2 units going in separate ways. A lot of miscommunication on the questioning.

Student B – very confusing. Miscommunication makes it difficult to learn.

Assessments online - feelings

Student D – technical difficulties – caused a lot of mental stress – feel that should have a backup question set to do it next day, instead of doing at sem 2 cause its worse. Open book issues with multiple tabs. Workshop assessments no answers given, so in offline its better sometimes answers given.

Student C – cause its online assessment, doesn't measure their ability to understand discovery. Cause open book may not test discovery.

Student A – stressed in assessments cause if anything wrong, can't ask invigilator, but offline its very stressed youre in your room alone, and panic, can't call tech support, and its, Also issues with only got 1 laptop and router, tech limitations in equipment.

Student B – retook in sem 2, the paper, and felt not worth.

What do you feel about discovery materials

Student D – lots of optional activities, and if assessed on, its unfair. Sometimes different resources say different things, and so which to use? And during exams which resource is correct?, if they use one ref how am I sure it'll be marked or I'll get someone saying you should use updated resource.

Student C – too many links in discovery

Student A – Initially discovery very in depth, but now (year 3), its all links and not defined what needs to be read. Discovery year 3, sem 2, is vague its all links.

Student B – same, don't know what to read, too many links, easy to get sidetracked.

Do you like or dislike flipped learning as compared to last time

Student D – A-levels, helped her self regulate, she quite likes active learning, find answer yourself is good. She likes active learning and interaction. She did her own study in an ACTIVE LEARNING style cause during A-levels, and feels that how uni should be anyway cause it's the way it should be. Monash system is good cause that's how it should be. Useful for coming out to work, helps you to think.

Student C – Enjoys interactive designs, can self learning. A-levels, is very traditional type, she originally doesn't like flipped, but after got used to it, now feels that active type learning is good for medical professional type, cause teaches you HOW to learn, so overall in long term its good. Got used to it during year 1,

Student A – Like assessments done before workshop, less time pressure cause can do first then only go workshop. A-levels – had past year QNS, and marking scheme. In Monash this doesn't exist.

Student B – likes workshops where given cases, cause prompts us to think about it, can discuss why and can implement what we have learned, MUFY. MUFY is a very active learning based thing, so the coping was easier.

Given a choice would you like to learn Pharmacy in this style

Student D – Prefers this style

Student C -

Student A -

Student B – prefers this style

Does it work for all units

Student D - doesn’t work for research, doesn’t work for PP cause not contextualize. To do it better, maybe just need a short IL, not a whole unit.

Student C - doesn’t work for research

Student A – doesn’t work for research

Student B – doesn’t work for research, cant self study research, cause its too new, cause no basics so even if search yourself can’t really get it. Contextualization is important , should do it a lot more.

Does flipped classrooms help you grow?

Student D – learn to be independent, originally hard to breahe, very lost. Helps them to learn how to learn, builds personality, learn how to search and plan own stuff.

Student C – learn out of syllabus can be good.

Student A – adaptability.

Student B – don’t know the limits, can be a disadvantage cause dunno where to stop. But also sometimes can be good.

Online improvements if it continues

Student D – lecturers should be more prepared, some may not even know what needs to be done, or even haven’t went through the case. Prefer sem 1, cause the Aussie lecturers seems better prepared, in sem 2 the Msian lectuers seemed lesser prepared. Need to make discovery clearer.

Student C -

Student A – Forget to send zoom link, was an issue. Backup assessments to overcome technical issues. Lecturers have technical issues in using software, should also be overcome, poll EV, Zoom.

Student B – better communication between lecturers in terms of planning for classes, since she continue year 3, want it more well planned,

Anything online you want to bring to offline classes.

Student D – Pre-recorded lectures – gives a more comprehensive understanding

Student C -

Student A – pre-recorded – clearer explanation,

Student B – likes the part by part pre-recorded lecture, for CTL, they would want a live session, but maybe either online or live in person.

Ranking (best….worse) 15X4 pre-record, 1 hour pre-record, 1 hour live (in person), 1 hour live (online)

Main reasons is cause of flexibility.

Deidentified Field Notes FG4Y4 - Sept 2020

Note: Though PL is the acronym of a student name - we did not replace it with student D, because PL is found in many words (e.g. reply, complex, people), so using "find and replace" would have been difficult. The acronym PL should be deidentified enough.

What do you feel about the online experience

Student A – Initially hard, but slowly improving, but still prefers live. In workshops can be totally silent, very different than live. In F2F easier to get lecturer attention, in breakout rooms workshops takes time. Virtual workshop less staff it's a bit understaffed.

Student C – feels weird at first, cause used to seeing F2F, especially workshops are weird, but slowly getting used to it, online more confidence in asking questions than offline. Overall, still like F2F, feels interaction in lecture hall is better.

PL – Likes virtual way of learning, cause a lot of flexibility, can arrange timings. And agrees that its easier to ask questions, online. Campus life may not matter much to me, so online in generally quite good. But initially, there was not so good organization, now its better.

Student B – Workshop is the most troublesome part of the online experience, cause hard to communicate, hard to get immediate response compared to F2F.

How does offline contrast to offline

Student A – Hard time, looking at timetable, zoom links a bit confusing. Some are hit and miss. Technical difficulties accessing zoom lectures. A bit case to case, for 4042 a lot of effort on keeping us updated. Physical lectures/workshop no barrier on asking questions, can stay back, immediate feedback. For virtual must use email/forum its an extra step which is an extra effort. Limitations of virtual. PA recordings available year 1-3, now in yr4 no more PA lectures, it's a drawback.

Student C – Feels that online way, lecturers are putting a lot more effort on making it work. Now a lot of documentation on questions and answers. This sem lecturers putting in a lot more effort compared to past 3 years.

PL -

Student B – E-lectures, easy to miss out deadlines for assessments, in F2F can easily remind, and online easy to forget the deadlines.

IL, CTL, workshop online vs. offline

Student A – Feels workshops experience less ideal, blank screen, webcam off, so online vs. offline workshop makes a big difference. CTL and IL offline pros – can immediately clarify doubts, and can ask friend

Pros of online – can take screenshots, and screens are close to us, no need to squint to find things.

Student C – IL CTL documenting is good, but workshops its way better to do it live, cause of communication mainly. Partly related to how workshops work this sem, now it's a series of workshops that are related, it's a bit more complex so harder to communicate. If got F2F workshop can force people to talk, can be an issue. Advantage of online is can share screen to show things. Overall pros and cons but communication and teamwork via F2F is better. In virtual workshops cant read expressions, so don't know what issues happening,

PL – Previously in workshop offline, sometimes questions asked they cant hear, but now in online, more people ask questions and I learn from them. Communication wise, she thinks less of an issue with online, mainly cause whatsapp, google docs can still do the normal communication style. Also feels the online experience is more efficient cause everyone just focus on their own things.

Student B – IL, CTL – can do online, no problems, its easier to follow, can do own self later. For workshops prefers F2F time, for bonding important.

Importance of bonding (scale of 10)

Student A – 11 (very important) – its about network, part of the on campus experience

Student C – 7-8

PL - 6

Student B – 8

Organization of online

Student A – Sem 1, too free, too busy, should have balanced out both sems. No classes sem 1, only classes sem 2. Compiling all the questions for the week and putting in forums was very good. For 5252, if told to contrast, sem 2 has no time to do analysis, need to prep poster, oral presentation, too much things in 5252. Should communicate the requirements earlier.

Student C – Feels that assignments are ok, but too repetitive, 2 diff health promotion, 2 diff practice innovation. Deadlines still manageable, putting more time on OSCE will stress more. Feels that having a balance is better. 5252 has a lot of things that if 5151 was done well could have helped. This group did all their analysis in sem 1, so in sem 2 had much less things.

PL – agrees in general with the workload, a lot of assignments should be done in sem 1. Now in sem 2, everyone anxious in exit OSCE, should have more time to self learn, and do practice exams, now very little prep time. Too many assignments

Student B – Too little work sem 1, sem 2 was too busy, should try to balance the workload over the sems. Assessment details released way too late for all units except 5252. For example, don't know final exam assessment details for final exams. A lot of uncertainty, OSCE, SOAP notes all not released, they are very worried,. E.g. video piStudent Bh should release details before hand earlier.

Transition offline to online, initial concerns?

Student A – One of the major concerns, was it's a brand new unit, and its gonna be done online, worried on communication barriers organization, and worried on getting assessments details. Now as time goes by concerns diminish over time. Communication with peers. One big issue is attendance, cause in physical class if someone is absent its obvious, and in online its hard to see.

Student C – initial concern, was communication barrier, its still there, felt it was hard to organize and gets responses during IL/CTL, but in reality responses questioning are better during online IL CTL. So actually advantage for virtual was the IL/CTL. Not existent in physical classroom. And also extracting all the questions and answering compiled form is a very good thing that probably would't be done in physical class.

PL – Asking questions was the main advantage now, much braver to ask questions, can type faster and clearer, cause when verbal can be worse. And sometimes peers are helping answer questions. And now can ask questions without interrupting others. And can see questions in chat whilst listening lecture. And save a lot of time for travelling. And can screenshot replay. Communication barrier in terms of don't meet people in class, can't read emotions, can also cause miscommunication. Organization of units wise, it's a bit individual.

Student B – Communication was the top concern, and is still there, now desensitized to it. Technical issues another concern, feels that tech issues a bit lesser now. Now less in terms of tech issues online

Why online forum, PollEV, now so responsive, before this was quiet

Student A – 2 major reasons, in physical class can ask and get reply immediately. And this sem, not much choice. And no replies even after post. Once lecturer replies, more motivated to reply and ask more. Now PollEV can compile questions early few days before lecture, so got time.

Student C – Also cause shy, and also no reply no asking

PL – Now in zoom chat got instant reply. In forums need to wait sometimes need for other peers to answer first. Speed of responses is quite important. Now lecturers are more repetitive in virtual learning, the lecturer more caring overall. There is a strange change in virtual classrooms where there is a big encouragement in asking questions.

Student B – Last time no enthusiasm to ask, cause last time no reply, now a lot of replies.

Do you think research can be thought online fully with no lectures?

Student A – No. 5151 – doesn't transition well to sem 2 (5252). Not effective in terms of time management, should not do this. Should have the information on 5252, then only go back 5151 and do that part.

Student C – No, cause things can't be explained easily, different interpretation of things, and choices of answers are subjective. Lots of ambiguity. Felt quite lost on 5052. There are things that are not easy to understand e.g. statistics, ethics. Also for 5252, there is also confusion, on how to do certain things, its not easy to do via moodle just self reading. Feels the method 5151-5252 is quite good, cause you do, then reflect and rethink through the project. Helps to make it clearer and adjust the whole flow. Feels that each supervisor had a clear aim in proj, if everyone had clearly known what they need to do before they choose the proj. Very important to stress that you need to see the supervisor first, so you know what is expected, then things will be better.

PL - No

Student B – No

Assessments feelings by online?

Student A – Concern with online e-exams may have tech issues, contrasted to physical e-exams if tech issue no probs, but in home it might be a lot more stressed.

Student C – likes open book assessments cause its easier for memory,

PL -

Student B –

Assessments online do they measure skills?

Student A – Easy to collude, its open book, less stress. Does not duplicate the physical e-exams as done from home. Online exams should be made more challenging.

Student C – Does not truly measure, cause its open book. In closed book you need to actually know the details. Based on OSCE, verbal, online actually still measures the actual performance. But still can collude, mostly no and a bit yes.

PL – Open book may not be that easy to score, cause many questions are more application or case based. Feels memorizing is quite redundant, so open book is actually more useful. Only thing is don't collude, so if can find info fast its quite good,

Student B -

Flipped learning section

What do you all think about discovery materials

Student A – Prefers discovery material over slides, cause its more text book like. Sometimes feel its too heavy with a lot of links. Feels that links should tell what to read and be specific. Should not include so many external links, and just incorporate what is needed.

Student C – initially feared discovery materials, initially the IL was a bit of a repeat of discovery. In workshop totally different, given a case and asked to solve. And told to be independent, CTL was presentation. After 5 weeks, they changed CTL was used then as clarifying doubts. Cause in year 1, what you don't know no CTL to clarify, so that's a very good thing. Initially discovery felt to be rather useless, cause can't see application. In year 2, since its clinical it helps. For PP not sure if the

discovery is useful, may not link to IL. For research 5052, 5252 discoveries were almost all they had. Alignment table is useful when tailored to our project.

PL -

Student B -

Thoughts on in class activities

Student A – Adapted to it, but overall when ask questions, no spoonfeeding. In PA its quite different, in terms of delays of class. In terms of answering questions, you ask questions and then they ask you to find, but may not come back and reply. Student A don't like y1,y2 in class, but likes y3 y4. Does not like the 10 minute discussion types so a bit weird. And some questions too simple, so don't need to discuss.

Student C – Overall, it depends on individual lecturers, perhaps when syllabus started, it was quite difficult, and throughout the years more and more interactions are seen, overall. IL are only IL cause of the interactivity. Likes it in general. Initially did not know what to expect, and now they start to feel that interactivity helps to gain more knowledge, and they now start to see the value, so they start to like it more. Starting year 3, sem 2 got this feeling that there is a good exchange.

PL – PL, don't like y1,y2 in class, but likes y3 y4. Feels that its changes now online to more interactive

Student B – Not supposed to spoonfeed, should give a plate of good food. Should open the door, and tell me where. And across semesters eventually started to get more constructive feedback. So feel that CTL now to cover concepts, instead of. Student B don't like y1,y2 in class, but likes y3 y4. Don't like year 1,2 but like final sem cause lecturers itself are more enthusiastic in discussing questions, what you get is what you give, positive vibes from lecturers.

Would you redo this if you had a choice?

Student A – would prefer interactive thingy, cause more flexibility of time. Traditional way of teaching relies a lot on. Made her more resourceful, can look up info.

Student C – feels like would still go through it, feels more confident, last time won't approach lecturer. Content delivered through interactive lectures. This system gives time to think what is learned.

PL – Initially, the quality of discovery was not there, so the IL was challenging, no cohesivity from one activity to another. Case sharing more useful. Having discovery is more useful, cause in traditional classroom, I'd just wait, but here I must read discovery and it compels students to work on studies.

Student B – If got choice, I would like IL more, cause timing wise its really a lot more flexible in timing, Comparatively other traditional ones that

Usefulness across different types of units?

Student A – Year 1 discovery too heavy and IL workshop no real relation. For PP not so useful to do flipped learning. For research needs more time to clarify.

Student C – good for CC, not so useful for PP. Lots of things need explanation before apply, not particularly useful for HMW type units. HMW was not very well built, its difficult.

PL -

Student B – PP type or moral units, might be better to talk with lecturers. Some things not explained whats the correct answers, and don't know whats the issue or why?

Student A – how to improve

How it helps improvement personal

Student A – resourcefulness, If don't develop discovery properly will affect the IL workshop so its important to getting discovery done.

Student C – helps to think of what learned, self reflection, more confident, and learn faster, voice opinions more, and can take charge of learning, learn value of peer learning, personal learning. Drawbacks would be too breadth of study.

PL –

Student B -

Improving program

Student A – IL online is better, compiling lectures and answer all together. Having Poll EV link 1-2 days before IL, instead of having them open in the lecture. Disovery can be better developed, research structure can be developed. Can try to contextualize better.

Student C – For year 1, can refine discovery scope to see the applications. Introduce CTL for year 1. Questions and answer sessions in virtual classrooms if could be translated to physical classrooms that would be good.

PL – if can maintain IL online would be good, lecture consolidation. Introducing optional lectures to add content.

Student B – improving, must have the sequence correct. Sometimes, there are some sessions where workshops come first then discovery, best if discovery day is Friday. Sequence is the most important thing.

Overall general consensus is like the program,

Deidentified Field Notes FG5Y1 - Sept 2020

Pre- U backgound

Student A - UEC

Student B: UEC

****

1. Online learning experience

   Student B: quite OK

   Student A: happy, like the flexibility, living interstate, although, no friends, struggling to connect and make friends

   Student B: Lack of friends, struggle

2. Organization of studies

   Student A: ok, feel like is organized ok, very little problems
   Student B: initial confused but unsure of existence of video recordings,
   Student A: Sem 2 improved better – live lectures,
   Student B: Sem 2 more organized
3. Initial concerns:
   Student A: had imagination that university life was more than studying..feeling a bit disappointed as it seems that uni is just studyings..
   Student B: expected uni life to be more interesting but due to online challenges, felt online learning not too bad though. (Flexibilitiy)
   Student B: technical issues and administration of lectures. Clarity and audio of presentation
   Student A: concerned about communication with lecturers but it does seems to be better.
   - Struggle to make friend and connection with people
   - Me

4. Assessments
   Student B: initially liked the idea of open book assesments but struggled with difficulty
   Student A: In semester exam was alright, hard to do presentation
   Student A: internet problems
   Student A: time management in issues
5. Assessments being fair and true measurement?
   a. Student A: unsure, open book exams easier?
   b. Student B: easy to find answers so unsure if it will be a measure of competency
   c. Student A + Student B: fair
6. Technology challenges
   a. Student A: Yes! Bad internet

    b. Student B: unstable internet
    c. Student B + Student A : could struggle during assessment
    d. Student B: feel frustrated
    e. Student A: would probably panic and freak out but haven't had it during assessments yet
7. Use of new softwares – moodle, zoom
    a. Student A: No problem so far
    b. Student B: initial struggle
8. Student A: prefer sem 2 IL vs Sem 1 – likes to ask question immediately and getting a response
   Student B: No preference for Sem 2 or Sem 1 lecture (recorded or live)
   Student A: Workshop online
   -enjoys Prof practice
   Student A: struggle with presentation and public speaking skills
   Student A: Workshops – people don't switch on camera and struggle to discuss and talk.
   Student B + Student A: prefer talking vs typing
   Student A: good idea to switch on camera and audio

CTL
Student B: ok with it
Student A: same

PART 2: flipped learning

1. Discovery materials
   Student A: likes to print discovery materials and read it – prefers words than clips
   Student B: opposite

   Student A: more IL's + explain discovery materials better
   Student A: past year papers
   Student B: having discovery beforehand is good and likes the ability to study a bit first
   Student A: did not have much oral exam in UEC
   Student A+ Student B: does enjoy asking questions and interaction, being invited to ask question

2. Student A: learn more about Malaysia context
   Student B
3. Student A: Monash Pharm more organized than other uni so far
   Use whatsapp group chat?

   Student B: email

   Student A: harder to ask f2f question in lecture but ok in workshop

   Other skills to develop
   Student B: team working skills (compared wo working with old schools friends and new strangers)

   Improvements:

Student A: More feedback

Student B: PASS sessions (real life vs zoom) – capacity

Student A + Student B: general ok with flipped learning

Student A + Student B: making friends has been biggest challenge so far in 2020, forcing Web cams switched on.

Student A + Student B: some interaction with seniors but did not ask for help .
