# Supplementary material for: Pharmacy students’ perspective on remote flipped classrooms in Malaysia: a qualitative study
Source: J Educ Eval Health Prof. 2025 Jan 14;22:2. doi: 10.3352/jeehp.2025.22.2 (PMC12055608; doi:10.3352/jeehp.2025.22.2)
Supplement: Supplementary file 3 [file jeehp-22-2-dataset3.pdf]

Dataset 3

Emergent themes in the study

Themes	Sub-themes
A. Flexibility	A1. Timing
	A2. Revisiting content
B. Communication	B1. Advantage – Increased engagement
	B2. Advantage - anonymity
	B3. Advantage – content & questions/answers recorded
	B4. Nuanced comprehension
	B5. Disadvantage – Absent non-verbal communication
	B6. Disadvantage – Limited peer interactions/unresponsiveness
	B7. Disadvantage – Slow assistance
C. Technological challenges	C1. Assessment anxiety
	C2. Limited access to activities
D. Skills-based learning challenges	D1. Extemporaneous compounding
	D2. Medical devices
	D3. Objective structured clinical examinations (OSCE)
E. Time based effects	E1. Initial Virtual Uncertainty
	E2. Longing for Campus Life
	E3. Remote learning fatigue
