# Supplementary material for: Pharmacy students’ perspective on remote flipped classrooms in Malaysia: a qualitative study
Source: J Educ Eval Health Prof. 2025 Jan 14;22:2. doi: 10.3352/jeehp.2025.22.2 (PMC12055608; doi:10.3352/jeehp.2025.22.2)
Supplement: Supplementary file 4 — Supplement 1. Themes, sub-themes, and example quotes from focus groups with pharmacy students. [file jeehp-22-02-suppl1.docx]

**Supplement 1.** Themes, sub-themes, and example quotes from focus groups with Pharmacy students. FG = focus group, Y = year

| **Themes** | **Sub-themes** | **Example quotes** |
| --- | --- | --- |
| 1. Flexibility | A1. Timing | We are allowed to complete our interactive lecture, anytime, anywhere, according to our own pace. (FG1, Y2) |
|  | A2. Revisiting content | If I miss anything, I just play back later to check. And everything can be screenshot perfectly clearly. (FG4, Y4) |
| 1. Communication | B1. Advantage – Increased engagement | But the pros of having virtual classes, like what Friend A and Friend B have said, is that people tend to ask more questions. (FG4, Y4)  And I feel each of us has more confidence in asking questions in the online session, compared to the [face to face] lecture. (FG4, Y4)  For online interactive lectures the biggest advantage is that you can ask questions quickly. You can just search and then the question is there. (FG1, Y2)  I could actually formulate my question before I actually send it up [via chat] so I could read through, I think is an advantage. (FG2, Y2) |
|  | B2. Advantage - anonymity | You can go anonymous. You can go non-anonymous, either way the lecturer will respond. (FG1, Y2) |
|  | B3. Advantage – content & questions/answers recorded | Because it's an online session, you can just type out whatever you want and you get the answer and it's documented in a way. In the lecture hall, I need to write down every single thing or record it on my phone, but now everything is recorded. (FG4, Y4)  And we do not need to take photos. We are so close to the screen that we do not even need to zoom in. (FG4, Y4) |
|  | B4. Nuanced comprehension | But to clarify concepts, especially on a process or a specific diagram, I prefer physical [face to face]. But for small questions, it's easier if it's typing out. And if it's a huge diagram and concept to understand then I prefer physical. (FG2, Y2) |
|  | B5. Disadvantage – Absent non-verbal communication | I also have a point to add that, through virtual workshops, I do not know what challenges or what difficulties my teammates are facing. Usually in the [face to face] workshop, I can see their faces as they're frowning, or they don't understand what you said. They frown at you, they go like... They look puzzled, but in a virtual workshop, I really don't know whether they understand me or whether they need help or they just didn't tell me. (FG4, Y4) |
|  | B6. Disadvantage – Limited peer interactions/unresponsiveness | The pros of the physical [face to face] classes is that we get [to] ask our friends outside, "Hey, what did the lecturers just say?" If we miss out on some of the points, we can get that clarified right on the spot [with lecturers], right after classes. (FG4, Y4)  But another thing I feel is that if we had a workshop face-to-face, it's easier to talk to the group members. At least if they don't talk to you, you can look at them and force them to talk to you. But then through the virtual classroom, I'm just talking to a blank screen. No one's opening their webcams, (FG4, Y4)  So the workshop could be a bit awkward when it is conducted online because some members might be unresponsive. They might be MIA [missing in action]. They can just leave their computers on. So it has happened before and it's a bit troublesome. (FG1, Y2)  I find trouble focusing on the workshop session, as we cannot interact with other groups, that's what we usually do. We have some chit chat, we have some gathering of information, but we are not allowed to do that because we are only in our breakout rooms, so we can only discuss among ourselves. (FG4, Y4) |
|  | B7. Disadvantage – Slow assistance | But in breakout rooms, it's quite difficult I would say, because even if we call for help, the lecturer normally takes some time. (FG4, Y4)  During case-based discussions, the lecturer can only focus on one group at a time. This contributed to some of the communication challenges we face during breakout sessions. (FG4, Y4)  And also asking questions is a bit troublesome because you have to invite the lecturer in and out from the Zoom [breakout] room. (FG1, Y2) |
| 1. Technological challenges | C1. Assessment anxiety | If the line drops during an assessment workshop, it is quite nerve-wracking. (FG2, Y2) |
|  | C2. Limited access to activities | Sometimes my line [internet connection] drops. I’m worried if the lecturers are saying something very important and my line just happens to not be working that time. (FG1, Y2)  Last few months there was a specific week where my internet connection was very weak. Out of 4 workshops that week and I got disconnected out of 3. (FG5, Y1) |
| 1. Skills-based learning challenges | D1. Extemporaneous compounding | For the extemporaneous [compounding] session after I finished my product, I'm not sure I did it correctly. And there's no one to physically check. They just can look at the picture. (FG7, Y2) |
|  | D2. Medical devices | And for the device counselling, I don't have all the devices at home. So even though I just watch the video, I cannot familiarise myself with all the devices. (FG7, Y2) |
|  | D3. Objective structured clinical examinations (OSCE) | For online OSCE is that I feel more confident and less nervous when doing it, I don't know why. (FG 6, Y3)  I think the other concern is interpersonal skills, what you just mentioned, because everything is online so some introverts or shy people may feel more comfortable doing it online, without switching on their cameras, and just having a chat. But it may become a problem if they go out and speak to future customers or future patients because even our OSCE [objective structured clinical examinations] is done online. (FG 6, Y3)  Because we're too reliant on our references, and I actually make simple notes on what to give for each disease. So I feel like I'll be less confident if someone just came in person and we all right away, and I have to think on the spot, it might be tough for me. (FG 6, Y3) |
| 1. Time based effects | E1. Initial Virtual Uncertainty | At the start of year one, because everything was transferred online, I’m quite lost. I’m not familiar and I don’t know where to find the information. (FG7 Y2)  The first one month, for me, in the first year, my first semester, I had to figure out everything. I feel very lost at times, and also very helpless. Because I don’t have any friends studying the same course with me [they couldn’t meet face to face]. And I got no one to ask about this, yeah. (FG7 Y2) |
|  | E2. Longing for Campus Life | I would like to go back to campus as well because we have been losing the physical touch for some time and I feel like the communication and the human interaction is also a very important part of being a pharmacist, especially the working experience. (FG6 Y3)  I’m super excited to go back because my house is not made for studying, it’s super noisy and the Wi-Fi is not that good. And I don’t even have a proper study table to study. The school is a conducive environment for us to study and learn, so I think the best places is to go back to the campus. (FG6 Y3) |
|  | E3. Remote learning fatigue | I could attend two to three events back-to-back. The downside would be it made me lose focus and I don’t feel like I’m fully participating in the events. (FG6 Y3)  I think it makes life easier and then we become lazier. But if you do it every day, you’ll feel your study life is super bland [boring] and then you’ll get tired of it, especially after doing this for two years. So I think it’s not really that good. (FG 6 Y3) |
